# Elucidating the mechanism of Buyang Huanwu Decoction in the treatment of ischemic stroke: A network pharmacology and molecular docking study

**DOI:** 10.1097/MD.0000000000049736

**Published:** 2026-07-17

**Authors:** Tailai Shi, Zhaoqi Chen, Mengmeng Wang, Danna Chen

**Affiliations:** a Department of Rehabilitation, Ningbo Zhenhai Longsai Hospital, Ningbo, Zhejiang, China.

**Keywords:** Buyang Huanwu Decoction, ischemic stroke, mechanism, molecular docking, network pharmacology

## Abstract

A large number of functional disorders and uncomfortable symptoms often remain following ischemic stroke (IS). Existing drug therapy is not ideal for the direct improvement of symptoms, which often leads to poor patient compliance with physical rehabilitation therapy. Buyang Huanwu Decoction (BYHWD) is a famous prescription that is effective in treating IS, especially during the sequela stage of IS. We analyzed the therapeutic mechanism of BYHWD through network pharmacology. This study aims to investigate the potential active ingredients, targets, and signaling pathways of BYHWD for the treatment of IS, utilizing network pharmacology and molecular docking technology. The active ingredients of 7 Chinese herbs in BYHWD were obtained from the Traditional Chinese Medicine Systems Pharmacology and HERB databases, and IS-related disease targets were searched in the DisGeNET, GeneCards, and OMIM databases. The protein–protein interaction network was constructed using the STRING database and analyzed by Cytoscape 3.10.2 software. Additionally, the target genes were uploaded to the Database for Annotation, Visualization, and Integrated Discovery website for Gene Ontology alongside Kyoto Encyclopedia of Genes and Genomes analyses. With the assistance of AutoDockTools and PyMOL software (Schrödinger, Inc.), a validation of molecular docking results and a visualization of the results were performed. The results showed that there were 190 intersection targets between the active drug components and IS, corresponding to 61 active components, among which the top 5 target genes were tumor suppressor protein 53, Jun proto-oncogene, AKT serine/threonine kinase 1, mitogen-activated protein kinase 1, and estrogen receptor alpha. The PI3K-Akt signaling pathway is one of the top 10 pathways. The molecular docking results indicated that most of the top 5 targets had good affinities for the 8 core compounds. This computational analysis suggests that BYHWD may treat IS through multiple targets and pathways. It may play a neuroprotective role by regulating the inflammatory response, oxidative stress, apoptosis, autophagy, and vascular endothelial homeostasis. The identification of core effective components provides a theoretical foundation and candidate compounds for further investigation into new drugs for the treatment of sequelae after IS.

## 1. Introduction

The World Stroke Organization’s Global Stroke Statistics Report 2025 shows that stroke remains the second leading cause of death among noncommunicable diseases worldwide, with ischemic stroke (IS) accounting for the highest proportion of new stroke cases.^[1]^ Although treatment technologies for IS in the acute stage have developed rapidly, treatment of the sequelae stage currently focuses on secondary prevention with drugs and physical rehabilitation, and pharmacological approaches remain relatively limited.

Buyang Huanwu Decoction (BYHWD) is composed of huang qi (Hedysarum Multijugum Maxim), dang gui (Angelicae Sinensis Radix Astragalus), chi shao (Radix Paeoniae Rubra), di long (Earthworm), chuan xiong (Chuanxiong Rhizoma), tao ren (Persicae Semen), and hong hua (Carthami Flos).^[2]^ Within these compounds, properties of qi invigoration, the facilitation of hematologic circulation, and collateral duct cleansing are enshrined. The prescription, which garners renown for alleviating symptomatic maladies post-IS episodes, originates from Wang Qingren, a distinguished physician of the illustrious Qing Dynasty era.^[3]^ A systematic review of 39 clinical randomized controlled trials conducted by Wang et al proved the safety of BYHWD in the treatment of patients with convalescent IS and its ability to improve National Institutes of Health Stroke Scale, critical size spectrum, and activities of daily living scores.^[4]^

Traditional Chinese medicine (TCM) syndrome differentiation of IS at the recovery and sequelae stages is mainly divided into 3 types: phlegm stasis blocking collaterals, deficiency in vitality (Qi) combined with stagnation of the blood, and insufficiencies of hepatic and renal origin. The medical formulation BYHWD primarily finds its therapeutic application among individuals exhibiting characteristics of such a deficiency and stagnation typology; notable amelioration is observed in symptoms manifesting as limb weakness, pallor with yellowish undertones on the facial epidermis, tongues bearing pale purple hues marked by ecchymosis, and pulses indicating diminished vigor.^[5]^ Clinical observations demonstrate that BYHWD plays an auxiliary role in facilitating patient recuperation during stroke convalescence.^[4,6]^

**Figure 1.**
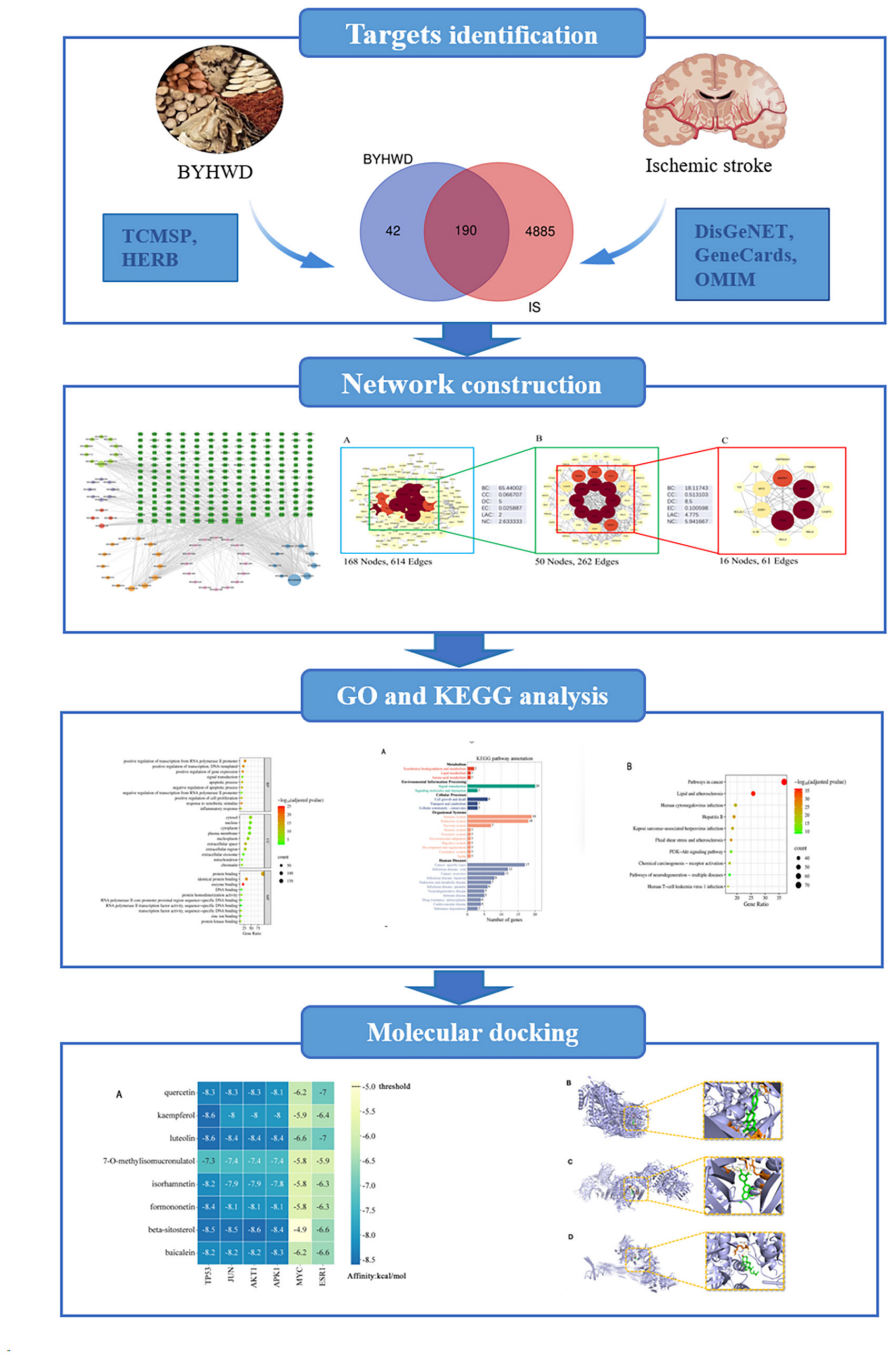
Analysis flowchart of Buyang Huanwu Decoction in treating the sequelae of ischemic stroke. BYHWD = Buyang Huanwu Decoction, GO = Gene Ontology, IS = ischemic stroke, KEGG = Kyoto Encyclopedia of Genes and Genomes, OMIM = Online Mendelian Inheritance in Man, TCMSP = Traditional Chinese Medicine Systems Pharmacology.

Therefore, this study attempts to further explore the potential core targets and pathways of the overall therapeutic effect of BYHWD on IS through network pharmacology (Fig. 1), providing a reliable reference value for subsequent experimental verification, new drug development, and new clinical applications.

## 2. Materials and methods

### 2.1. Screening of active components and relative targets in BYHWD

The active ingredients of 7 Chinese herbs in BYHWD were obtained from the Traditional Chinese Medicine Systems Pharmacology (TCMSP, https://www.tcmsp-e.com/, version 2.3, accessed on July 6, 2025) and HERB (http://herb.ac.cn, version 2.0, accessed on July 6, 2025) databases. Using TCMSP’s optimal ADME (Absorption, Distribution, Metabolism, and Excretion) criteria, candidates among Chinese herbal constituents were selected with oral bioavailability (OB) > 30% and drug-likeness > 0.18. Compounds that did not meet these thresholds were excluded. Registered targets were annotated using the Search Tool for the Retrieval of Interacting Genes/Proteins (STRING, https://cn.string-db.org/, version 12.0, accessed on July 8, 2025) or UniProt (https://www.uniprot.org, release 2025-04, accessed on July 8, 2025) databases for gene name annotation.

### 2.2. Retrieving the relative disease targets of IS and capturing the overlapping targets

The Disease Gene Network (DisGeNET, https://disgenet.com/, version 25.2, accessed on July 6, 2025), GeneCards (https://www.genecards.org/, version 5.24, accessed on July 6, 2025), and Online Mendelian Inheritance in Man (OMIM, https://omim.org/, updated July 2025, accessed on July 6, 2025) databases were searched to identify disease-associated targets using the keyword “ischemic stroke.” These targets were then integrated for further analysis. Ultimately, by integrating and removing redundancies among the targets unique to each disease database, we obtained the combined set of IS targets.

### 2.3. Constructing the database of intersecting drug-disease target genes

After uploading, the active pharmacological constituents and pathological entities of interest were submitted to the Bioinformatics & Evolutionary Genomics portal (https://bioinformatics.ugent.be/webtools/Venn/), where Venn diagrams were created. The intersection gene set from this operation was obtained, and the target gene library of the active drug ingredients was subsequently constructed. Using Cytoscape 3.10.2 software, a network illustrating the relationships between these active ingredients and their targets was generated.

### 2.4. Constructing the protein–protein interaction (PPI) network

STRING (https://string-db.org/), a platform that enables dynamic exploration of gene data, offers insights into the intricacies of PPIs. We input the intersecting gene arrays into STRING version 12.0 to delineate the complex network of protein interactions, utilizing the “multiple proteins” analysis module, specifying “Homo sapiens” as the species, and setting the interaction confidence threshold to “highest (0.9).” This approach provides insights into PPIs and is further refined using Cytoscape software, version 3.10.2 (The Cytoscape Consortium), which plays an instrumental role in improving the visualization and functional interpretation of the PPI network map, thereby facilitating the identification of pivotal hub genes within the studied milieu.

### 2.5. Functional enrichment analyses of GO and KEGG

The Gene Ontology (GO) consortium developed an up-to-date and comprehensive computational framework of biological systems. We used GO enrichment analysis to understand the molecular functions (MFs), biological processes (BPs), and cellular components (CCs) of a gene set in a species and how they contribute to biological mechanisms. The Kyoto Encyclopedia of Genes and Genomes (KEGG) database helps elucidate biological system functions based on molecular information. The Database for Annotation, Visualization, and Integrated Discovery (DAVID, https://davidbioinformatics.nih.gov/tools.jsp) is a repository of functional annotation tools. The compiled intersection gene target list was submitted to DAVID (released December 2021) for GO and KEGG analyses, yielding datasets on CC, MF, BP, and KEGG pathways to provide genomic insights.

### 2.6. Verification of ingredient-target interaction via molecular docking

In drug design, molecular docking is based on receptor characteristics and receptor-drug molecule interactions. Theoretical simulation clarifies molecular interactions and predicts binding. It has become a key tool in computer-aided pharmacological research.

The top 6 hub genes in the PPI network were identified as core targets. Gene IDs (entry IDs) were found via UniProt. In the Protein Data Bank (PDB, https://www.rcsb.org/), entry IDs were entered, suitable receptor proteins were selected, and PDB files were downloaded as receptors. PyMOL was used to remove water and organic molecules from the PDB files. In the ingredient-target network, the 8 high-degree active ingredients were ligands. PubChem IDs were obtained from TCMSP, and 2D SDF files were downloaded from PubChem (https://pubchem.ncbi.nlm.nih.gov/). Chemical Draw 3D was used to modify the ligands to low-energy conformations and save them as mol2 files.

AutoDock, widely recognized in molecular biochemistry for docking and virtual screening, was used to add hydrogen atoms to receptor proteins and save them as pdbqt files. We used AutoDockTools to add hydrogen atoms to receptors and ligands and save them as pdbqt files, add charges, adjust the grid box size (spacing [angstrom] = 1), perform molecular docking via the Command Prompt to determine the minimum binding energy, and construct the binding energy heatmap on the Wei Sheng Xin platform (http://bioinformatics.com.cn).

### 2.7. Ethical approval

This study does not involve any studies with human participants or animals performed by any of the authors.

## 3. Results

### 3.1. Screening active ingredients and obtaining targets of BYHWD

Queries to the TCMSP and HERB databases were used to extract BYHWD’s compounds and their predictive targets. ADME screening with parameters of OB ≥ 30 and drug-likeness ≥ 0.18 identified 104 bioactive constituents associated with the primary pharmaceuticals within this prescription. Among them, huang qi included 20 types, dang gui 2 types, chi shao 29 types, di long 1 type, chuan xiong 7 types, tao ren 23 types, and hong hua 22 types. Table 1 shows only the top 5 active ingredients by OB value for each herb. By combining and removing duplicate primary screening ingredients and their corresponding targets, 91 active ingredients and 233 drug targets were obtained. The raw data for the active ingredients of BYHWD and their corresponding target genes are listed in Supplementary Table S1, Supplemental Digital Content 1.

**Table 1:** The top 5 active compounds from each Chinese herb in the Buyang Huanwu Decoction.

Drug	Mol ID	Molecule name	OB (%)	DL
Huang qi	MOL000398	isoflavanone	109.98	0.29
Huang qi	MOL000378	7-O-methylisomucronulatol	74.68	0.29
Huang qi	MOL000392	formononetin	69.67	0.21
Huang qi	MOL000433	FA	68.96	0.7
Huang qi	MOL000438	(3R)-3-(2-hydroxy-3,4-dimethoxyphenyl)chroman-7-ol	67.66	0.26
Dang gui	MOL000449	stigmasterol	43.82	0.75
Dang gui	MOL000358	beta-sitosterol	36.91	0.75
Chi shao	MOL001918	paeoniflorgenone	87.59	0.36
Chi shao	MOL001925	paeoniflorin_qt	68.17	0.39
Chi shao	MOL000358	beta-sitosterol	65.33	0.75
Chi shao	MOL007016	paeoniflorigenone	65.08	0.36
Chi shao	MOL000449	stigmasterol	64.73	0.75
Di long	MOL000953	cholesterol	37.87	0.67
Chuan xiong	MOL000433	FA	68.96	0.7
Chuan xiong	MOL002140	perlolyrine	65.94	0.27
Chuan xiong	MOL002151	senkyunone	47.66	0.24
Chuan xiong	MOL002157	wallichilide	42.31	0.7
Chuan xiong	MOL001494	mandenol	41.99	0.19
Tao ren	MOL001371	populoside_qt	108.88	0.2
Tao ren	MOL001351	gibberellin A44	101.61	0.54
Tao ren	MOL001348	gibberellin 17	94.64	0.49
Tao ren	MOL001353	GA60	93.16	0.53
Tao ren	MOL001349	4a-formyl-7alpha-hydroxy-1-methyl-8-methylidene-4aalpha,4bbeta-gibbane-1alpha,10beta-dicarboxylic acid	88.59	0.46
Hong hua	MOL002712	6-hydroxykaempferol	62.13	0.27
Hong hua	MOL002680	flavoxanthin	60.41	0.55
Hong hua	MOL002717	qt_carthamone	51.02	0.2
Hong hua	MOL002694	4-[(E)-4-(3,5-dimethoxy-4-oxo-1-cyclohexa-2,5-dienylidene)but-2-enylidene]-2,6-dimethoxycyclohexa-2,5-dien-1-one	48.46	0.36
Hong hua	MOL002710	pyrethrin II	48.35	0.35

DL = drug-likeness, OB = oral bioavailability.

### 3.2. Prediction of IS targets

From the 3 disease repositories – DisGeNET, GeneCards, and OMIM – a total of 1159 targets from DisGeNET, 4789 targets from GeneCards, and 20 targets from OMIM were compiled. This aggregation yielded a total of 5968 distinct pathological targets. After removing duplicates, a total of 5075 targets were obtained (Supplementary Table S2, Supplemental Digital Content 2).

### 3.3. BYHWD active ingredient-target network diagram

**Figure 2.**
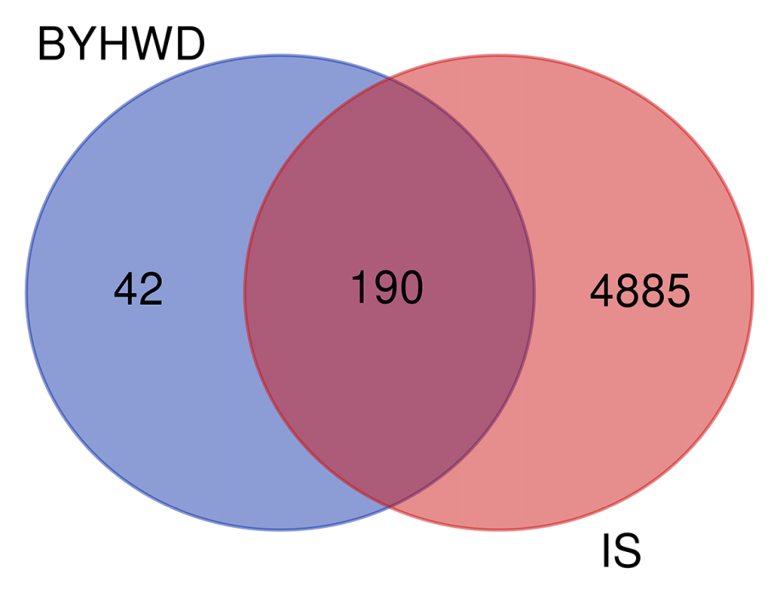
Venn diagram of BYHWD for IS treatment. BYHWD = Buyang Huanwu Decoction, IS = ischemic stroke.

**Figure 3.**
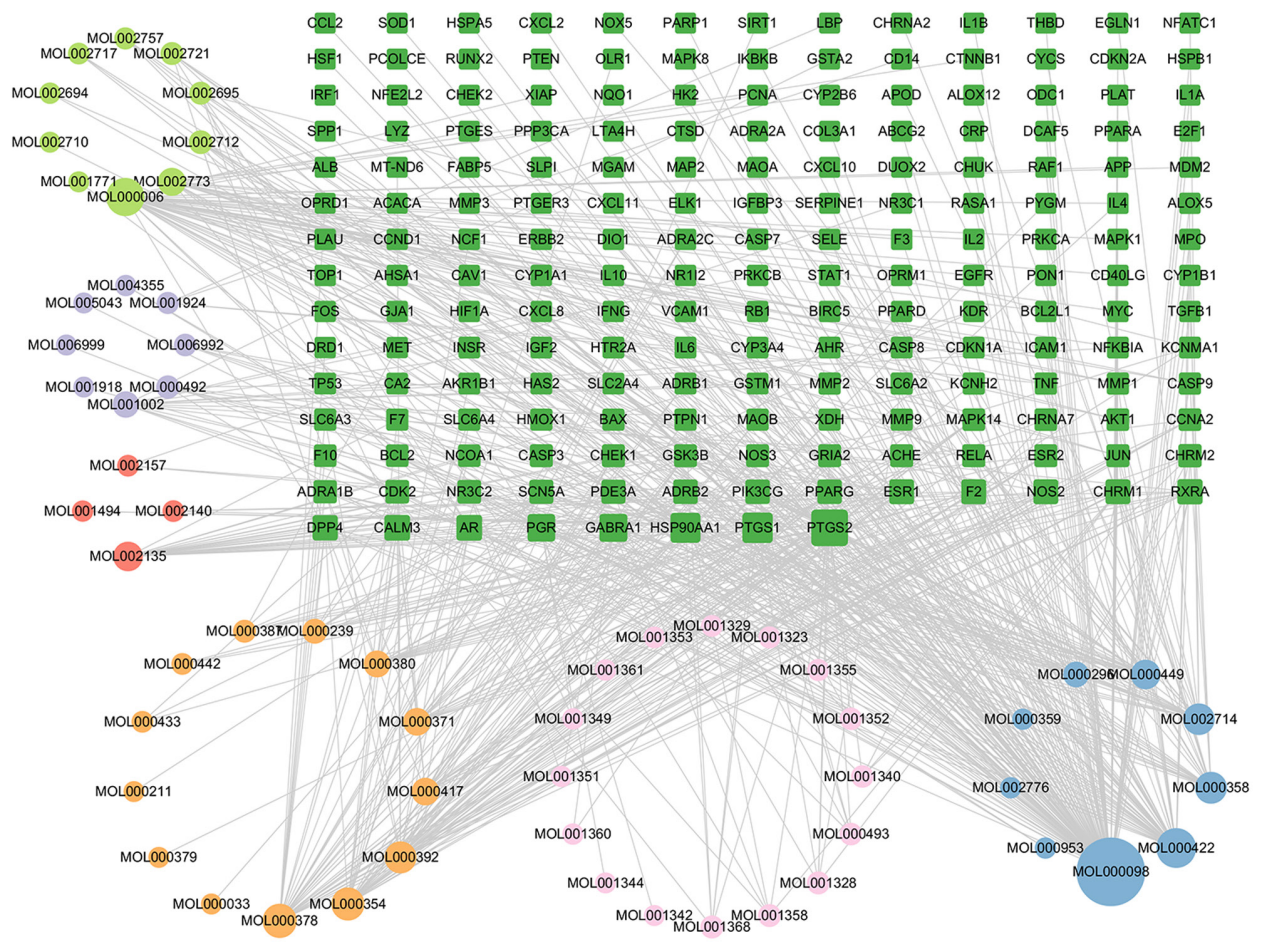
Active ingredient-target network of BYHWD. This network shows the targeted relationship between the active ingredients of TCM and the intersection of genes. Squares represent targets. Circles represent ingredients. Light green represents hong hua; purple, chi shao; red, chuan xiong; orange, huang qi; pink, tao ren; and blue, co-active ingredients. The following compounds belong to multiple herbs: MOL000098 and MOL000422 to huang qi and hong hua; MOL000296 to huang qi and tao ren; MOL000358 to di long, chi shao, tao ren, and hong hua; MOL000359 to chi shao and chuan xiong; MOL000449 to di long, chi shao, and hong hua; MOL000953 to di long and hong hua; MOL002714 and MOL002776 to chi shao and hong hua. BYHWD = Buyang Huanwu Decoction, TCM = traditional Chinese medicine.

Drug therapeutic targets and disease targets were input into the Bioinformatics & Evolutionary Genomics platform to generate the Venn diagram (Fig. 2), and 190 intersection targets were obtained (Table 2). Supplementary Table S3, Supplemental Digital Content 3, presents the relationship between the 190 target genes and the functional proteins. Sixty active ingredients in BYHWD were associated with the 190 intersection targets, and the relationship between these drugs, ingredients, and targets is shown in Figure 3. Supplementary Table S4, Supplemental Digital Content 4, details the correspondence among Chinese medicinal herbs, components, target proteins, and target genes. The relationships among TCM drugs, active ingredients, and their corresponding degree are shown in Supplementary Table S5, Supplemental Digital Content 5. The top 8 ingredients by degree are: MOL000098 (quercetin, degree = 123), MOL000422 (kaempferol, degree = 49), MOL000006 (luteolin, degree = 45), MOL000378 (7-O-methylisomucronulatol, degree = 35), MOL000354 (isorhamnetin, degree = 32), MOL000392 (formononetin [FMN], degree = 29), MOL000358 (beta-sitosterol, degree = 29), and MOL002714 (baicalein, degree = 28). The top 5 intersection targets by degree are: PTGS2, PTGS1, HSP90AA1, GABRA1, PGR.

**Table 2:** The intersection targets of Buyang Huanwu Decoction and ischemic stroke.

No.	Gene	No.	Gene	No.	Gene	No.	Gene	No.	Gene
1	MMP2	39	CXCL10	77	HIF1A	115	PDE3A	153	DUOX2
2	KCNMA1	40	CDKN1A	78	RELA	116	TGFB1	154	CASP8
3	XDH	41	DIO1	79	SLC2A4	117	STAT1	155	PPARG
4	NOS2	42	RASA1	80	MAP2	118	GABRA1	156	CXCL11
5	MET	43	NFKBIA	81	NOS3	119	CD14	157	CXCL8
6	PRKCB	44	IGFBP3	82	INSR	120	NR3C2	158	NR3C1
7	GSTA2	45	ALB	83	**AKT1**	121	HMOX1	159	RAF1
8	MAOA	46	CHRNA7	84	CYP2B6	122	GSTM1	160	ADRA1B
9	BCL2	47	PTGER3	85	F3	123	**MAPK1**	161	PCNA
10	F7	48	SLC6A2	86	NR1I2	124	PLAT	162	CHUK
11	CYP1A1	49	IRF1	87	PYGM	125	IKBKB	163	SLPI
12	CHRM1	50	RXRA	88	CDKN2A	126	IL4	164	ADRB2
13	AHR	51	ACACA	89	**JUN**	127	APOD	165	CTSD
14	NFE2L2	52	NFATC1	90	AR	128	NOX5	166	CCL2
15	XIAP	53	PON1	91	CD40LG	129	MAPK8	167	HSP90AA1
16	OLR1	54	CHEK1	92	CYP3A4	130	AKR1B1	168	PARP1
17	NCF1	55	SELE	93	APP	131	RB1	169	KDR
18	**MYC**	56	THBD	94	HSPB1	132	**TP53**	170	PPARA
19	CCNA2	57	MAPK14	95	DCAF5	133	CASP9	171	IGF2
20	MT-ND6	58	MPO	96	CYCS	134	LYZ	172	IL2
21	GSK3B	59	EGLN1	97	PCOLCE	135	SIRT1	173	HSPA5
22	MMP1	60	CASP7	98	BIRC5	136	IL1B	174	HK2
23	PPARD	61	ACHE	99	PPP3CA	137	HTR2A	175	ICAM1
24	ALOX12	62	IL6	100	FABP5	138	ESR2	176	BCL2L1
25	MMP3	63	CASP3	101	ADRA2A	139	CYP1B1	177	CA2
26	IL10	64	COL3A1	102	PTGES	140	SLC6A3	178	HSF1
27	MDM2	65	MGAM	103	CRP	141	LTA4H	179	CHRM2
28	CXCL2	66	ABCG2	104	CHRNA2	142	DRD1	180	OPRM1
29	EGFR	67	PTPN1	105	ADRB1	143	ADRA2C	181	CALM3
30	SOD1	68	MAOB	106	TNF	144	PLAU	182	SERPINE1
31	ERBB2	69	ELK1	107	IL1A	145	PTEN	183	VCAM1
32	F10	70	AHSA1	108	SPP1	146	SLC6A4	184	PTGS1
33	NCOA1	71	PIK3CG	109	F2	147	ALOX5	185	BAX
34	CHEK2	72	NQO1	110	PTGS2	148	GJA1	186	PRKCA
35	CDK2	73	IFNG	111	CTNNB1	149	DPP4	187	SCN5A
36	RUNX2	74	FOS	112	HAS2	150	LBP	188	GRIA2
37	PGR	75	OPRD1	113	CCND1	151	KCNH2	189	MMP9
38	ODC1	76	CAV1	114	**ESR1**	152	E2F1	190	TOP1

GO and KEGG enrichment analyses were performed, and the functional proteins corresponding to 190 target genes, as well as the relationships among Chinese medicinal materials, components, target proteins, and target genes, are recorded in the Supplementary Table S4, Supplemental Digital Content 4. Genes shown in bold are the top 6 key targets in the PPI network.

AKT1 = AKT serine/threonine kinase 1, BAX = Bcl-2-associated X protein, ESR1 = estrogen receptor alpha, GO = Gene Ontology, JUN = Jun proto-oncogene, KEGG = Kyoto Encyclopedia of Genes and Genomes, MAPK1 = mitogen-activated protein kinase 1, MPO = myeloperoxidase, PPI = protein–protein interaction, SIRT1 = sirtuin 1, STAT1 = signal transducer and activator of transcription 1, TP53 = tumor suppressor protein 53.

### 3.4. PPI network and topological analysis

**Figure 4.**
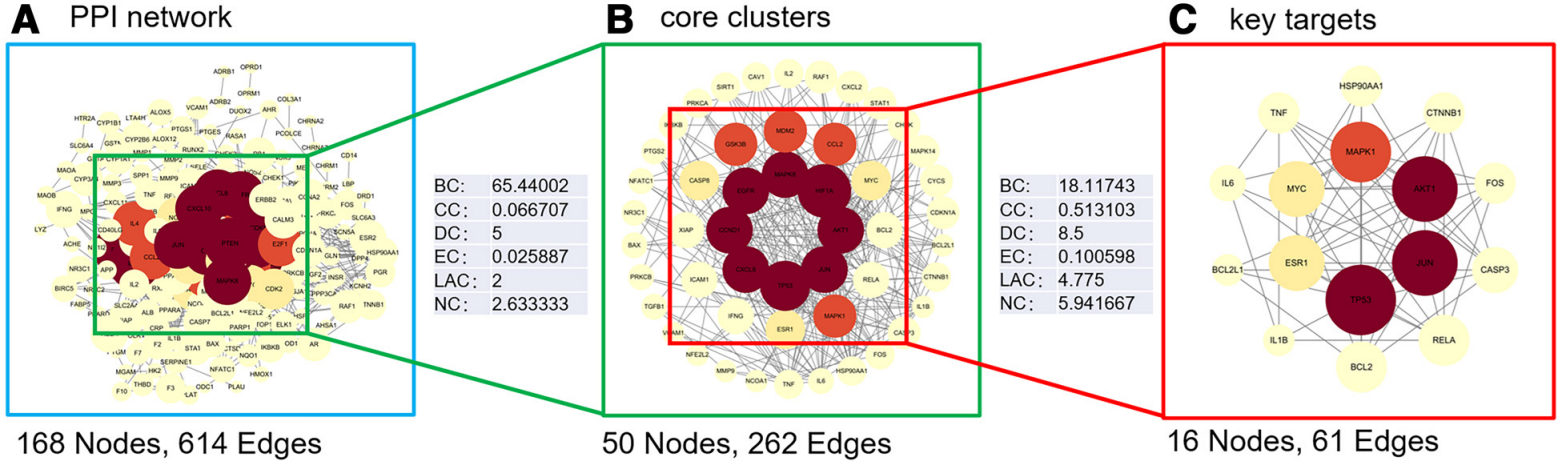
Network diagram of the PPI network, core cluster, and key targets: (A) PPI network, (B) core cluster, (C) key targets. A node with a larger size and deeper color possesses a higher degree value, such as TP53, JUN, AKT1, MAPK1, MYC, and ESR1. AKT1 = AKT serine/threonine kinase 1, BC = betweenness centrality, CC = closeness centrality, DC = degree centrality, EC = eigenvector centrality, ESR1 = estrogen receptor alpha, JUN = Jun proto-oncogene, LAC = local average connectivity-based method, MAPK1 = mitogen-activated protein kinase 1, NC = network centrality, PPI = protein–protein interaction, TP53 = tumor suppressor protein 53.

A total of 190 intersection targets were input into STRING 12.0 with a minimum interaction score >0.9 (Supplementary Table S6, Supplemental Digital Content 6, presents the results). We obtained 168 targets and constructed a BYHWD PPI network for IS using Cytoscape 3.10.2 (Fig. 4A, Supplementary Table S7, Supplemental Digital Content 7, presents the data), with 168 nodes and 614 edges. The average node degree was 7.2958. The CytoNCA (version 2.1.6; Central South University) extension was used to find key targets. By applying thresholds of betweenness >65.44002, closeness >0.066707, degree >5, eigenvector >0.025887, local average connectivity >2, and network >2.633333, a core cluster consisting of 50 nodes and 262 edges was formed (Fig. 4B). A more stringent filtering with betweenness >18.11743, closeness >0.513103, degree >8.5, eigenvector >0.100598, local average connectivity >4.775, and network >5.941667 yielded a cluster of 16 nodes (key targets) and 61 edges (Fig. 4C). Darker colors indicate higher node degrees. The top 6 degree nodes are tumor suppressor protein 53 (TP53), Jun proto-oncogene (JUN), AKT serine/threonine kinase 1 (AKT1), mitogen-activated protein kinase 1 (MAPK1), MYC, and estrogen receptor alpha (ESR1) (Table 3). The core targets were screened using the median (50% quantile) as the threshold, and the 75% quantile was used for sensitivity analysis. The overlap rate of the 2 groups of targets was 81.25%, indicating that the screening method based on the median is highly stable. The results do not rely on a single threshold, and the method has strong robustness (Supplementary Table S8, Supplemental Digital Content 8).

**Table 3:** The top 6 key targets in the protein–protein interaction network.

Target name	Betweenness	Closeness	Degree	Eigenvector	LAC	Network
TP53	291.3434	0.662162	28	0.317405	8.285714	21.86923
JUN	341.2218	0.671233	25	0.280185	7.36	17.7461
AKT1	202.1073	0.662162	24	0.280907	7.666667	17.24157
TNF	249.2422	0.620253	20	0.162422	5.8	14.32009
IL6	164.029	0.583333	18	0.145766	5.888889	13.2535
ESR1	66.43971	0.590361	18	0.237017	7.666667	12.26222

AKT1 = AKT serine/threonine kinase 1, ESR1 = estrogen receptor alpha, JUN = Jun proto-oncogene, LAC = local average connectivity, TP53 = tumor suppressor protein 53.

### 3.5. GO BPs of common target genes and KEGG pathway analysis

**Figure 5.**
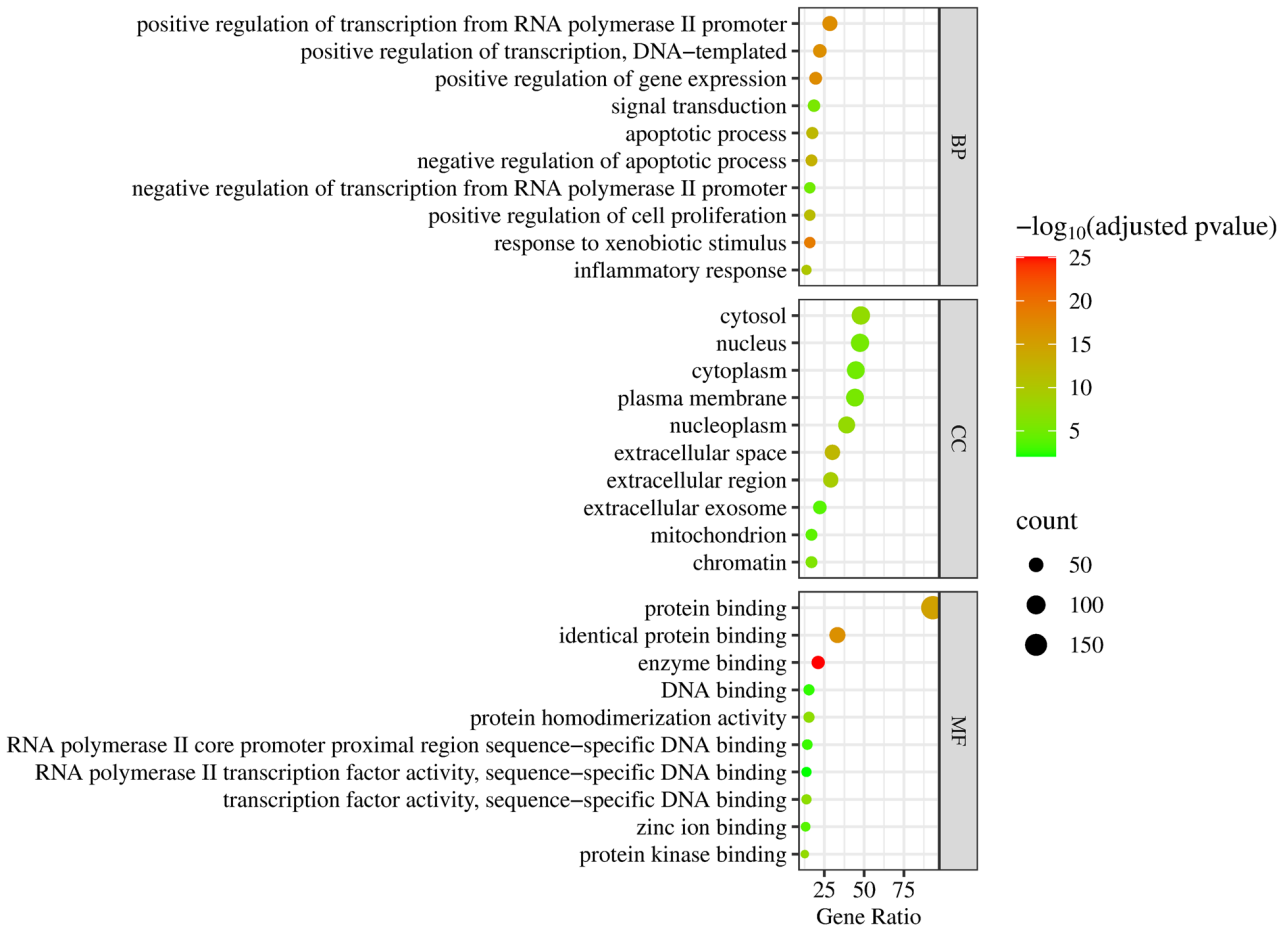
Each top 10 terms of GO functional enrichment analysis of BP, CC, and MF. BP = biological process, CC = cellular component, GO = Gene Ontology, MF = molecular function.

**Figure 6.**
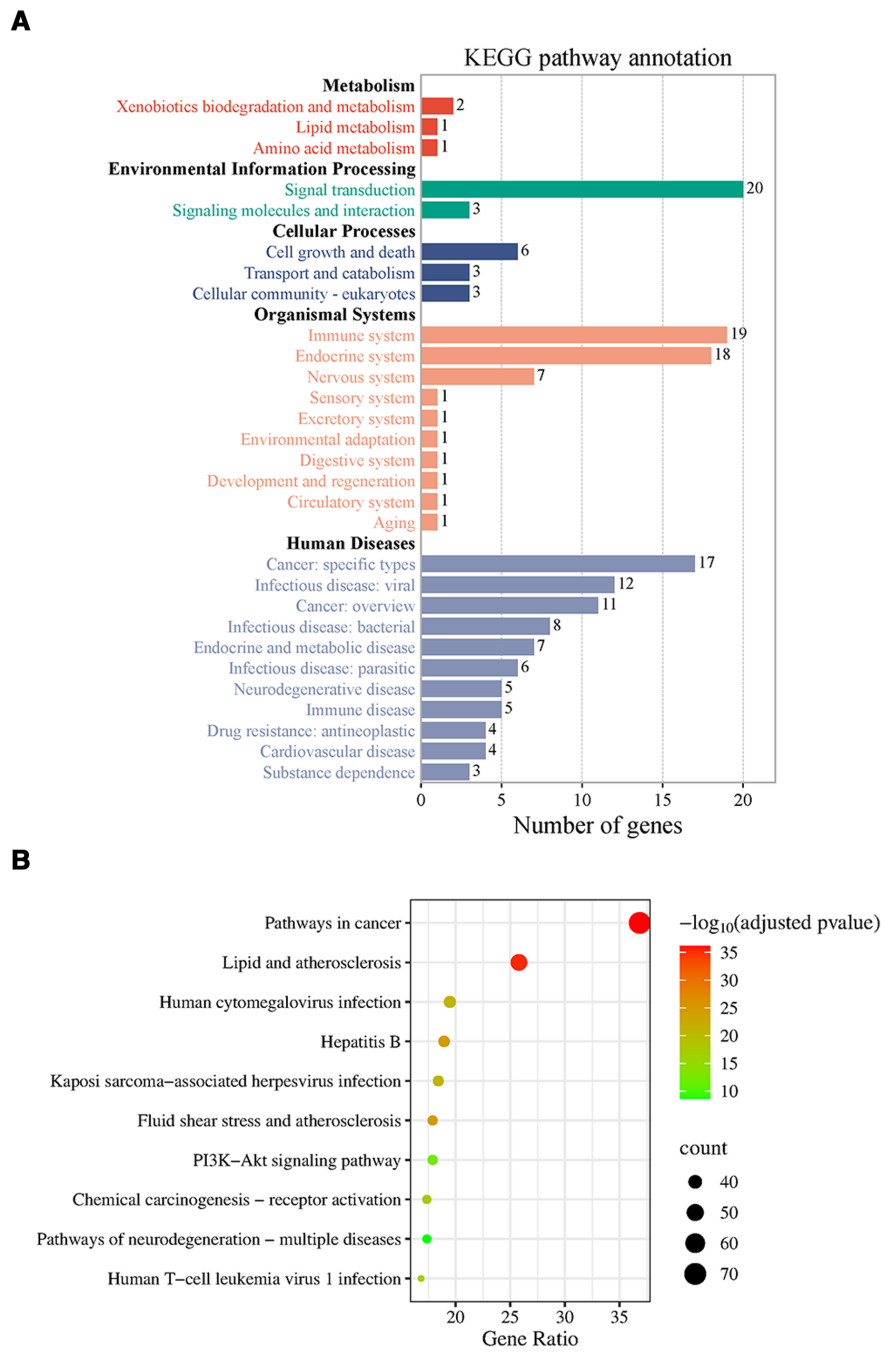
KEGG analysis: (A) secondary classification annotation of KEGG pathways; (B) top 10 pathways of KEGG analysis. KEGG = Kyoto Encyclopedia of Genes and Genomes.

Genes at the intersection of BYHWD’s pharmacological agent targets and IS-related genes were uploaded to DAVID for GO and KEGG analyses. The initial analysis results are presented in Supplementary Tables S9 and S10, Supplemental Digital Content 9 and 10, respectively. Multiple testing correction was performed using the Benjamini–Hochberg method, and terms with adjusted *P* values <.05 were considered statistically significant. The numbers of CCs, MFs, and BPs were 49, 77, and 349, respectively (adjusted *P* < .05). A bubble chart in Figure 5 shows the top 10 GO terms for CC, MF, and BP based on gene ratios. The top 5 CCs included cytosol, nucleus, cytoplasm, plasma membrane, and nucleoplasm. Prevalent MFs were protein binding, identical protein binding, enzyme binding, DNA binding, and protein homodimerization activity. BPs involved positive regulation of transcription from RNA polymerase II promoter, positive regulation of transcription (DNA-templated), positive regulation of gene expression, signal transduction, and apoptotic process. KEGG analysis revealed 172 significant signaling pathways (adjusted *P* < .05). KEGG classified results at 3 levels. The top 5 secondary classifications were signal transduction, cancer: overview, endocrine system, immune system, and cardiovascular disease (Fig. 6A). The top 10 pathways are shown by gene ratio in Figure 6B, with details in Supplementary Table S11, Supplemental Digital Content 11.

### 3.6. Molecular docking analysis

**Figure 7.**
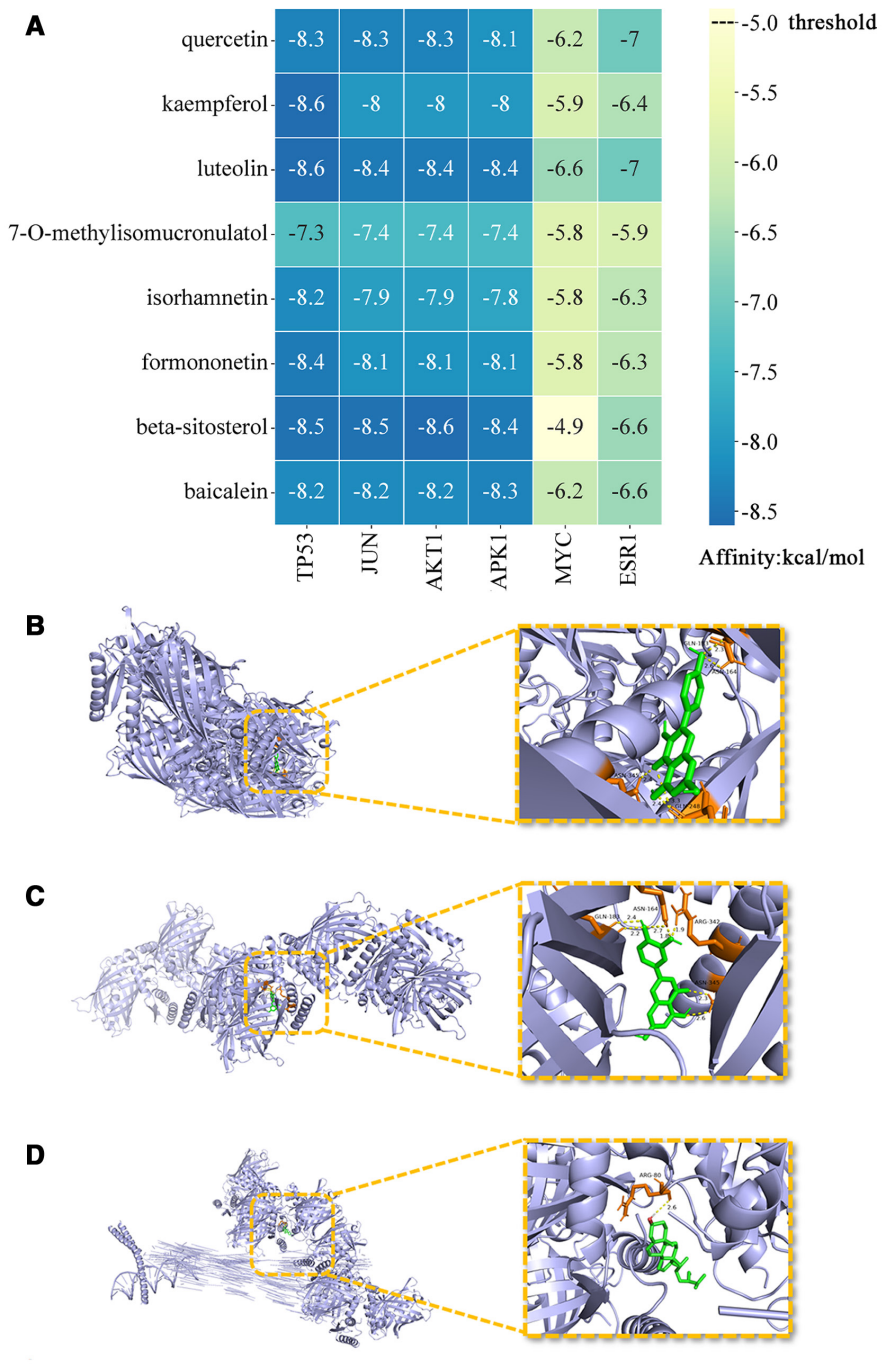
Molecular docking simulation of interactions between bioactive compounds and key targets: (A) Heatmap depicting binding energy interactions between proteins and bioactive compounds in Buyang Huanwu Decoction. Binding energy <−5.0 kcal/mol is used as a screening criterion, indicating a high binding affinity between the ligand and protein. (B) TP53-kaempferol, (C) TP53-luteolin, (D) AKT1-beta-sitosterol. Sticks represent ligands, and the cartoon representation depicts the hub target protein. TP53 = tumor suppressor protein 53.

The core targets (TP53, JUN, AKT1, MAPK1, MYC, and ESR1) in the PPI network were subjected to molecular docking with the core active ingredients (quercetin, kaempferol, luteolin, 7-O-methylisomucronulatol, isorhamnetin, FMN, beta-sitosterol, and baicalein) in the active ingredient-target network. The binding energy was <−5.0 kcal/mol, indicating efficient ligand-protein binding and high conformational stability. This value serves as a screening criterion rather than an absolute indicator of biological activity. Our research showed that binding energies ranged from −8.6 to −4.90 kcal/mol. Heatmap analysis showed the lowest binding energies (Fig. 7A), and the docking results of low-energy molecular pairs (TP53-kaempferol, etc) are shown in Figure 7B to D. The identification information for ligands and receptors across each experimental platform is recorded in Supplementary Table S12, Supplemental Digital Content 12. The actual grid box dimensions and center coordinates of each receptor are also provided. We used the same protocol to dock a known active inhibitor or activator into the active site of the target protein. The results (Supplementary Table S13, Supplemental Digital Content 13) confirmed that our docking procedure successfully identified positive controls, thus validating the reliability of our docking method.

## 4. Discussion

The post-acute phase of IS causes many sequelae, such as hemiplegia, sensory loss, cognitive and mental issues, and speech and swallowing problems. Progress has been made in stroke detection and treatment. However, post-acute management and sequelae mitigation mainly rely on physiotherapy, lifestyle changes, and etiological treatment. Drug treatment is limited to antiplatelet and anticoagulant therapies. There is a lack of effective comprehensive drugs for the psychosomatic symptoms resulting from functional disorders.

In TCM theory, IS is classified as “stroke disease.” Different symptoms in stroke convalescence and sequelae are classified with corresponding treatments and prescriptions. The “Qi deficiency and blood stagnation” syndrome manifests as hemiplegia, weak limbs, and other symptoms. Treatment includes qi supplementation, blood enrichment, stasis resolution, and collateral clearing. Wang Qingren’s YiLin GaiCuo in the Qing Dynasty presented BYHWD. Huang qi augments Qi, dang gui nourishes blood, and the other ingredients enhance circulation and dispel stasis. A meta-analysis by Shao et al on 32 randomized controlled trials of BYHWD for stroke sequelae showed that BYHWD improved clinical effectiveness and neurological rehabilitation with no adverse events.^[6]^ BYHWD can improve stroke patients’ functional status in the convalescent and sequelae stages and treat various complications.^[4,6–8]^ Following the common treatment principle of “replenishing qi and activating blood circulation,” BYHWD can be used to treat various diseases, such as hemorrhagic stroke, atherosclerosis, acute attack of chronic pulmonary heart disease, pulmonary fibrosis, and diabetic nephropathy.^[2,9–11]^ Its multi-target effects may involve improving microcirculation, exerting antioxidant and anti-inflammatory effects, and promoting angiogenesis.^[9–11]^ However, the molecular pharmacological mechanisms and underlying material basis of specific diseases remain to be fully understood.

### 4.1. Top 8 ingredients

Through the synthesis of all BYHWD objectives and disease markers, a total of 190 related targets were identified. Among these, a group of 8 key ingredients – quercetin, kaempferol, luteolin, 7-O-methylisomucronulatol, isorhamnetin, FMN, beta-sitosterol, and baicalein – was identified and highlighted in the active ingredient-target compendium.

Quercetin promotes neuroprotection against ischemic brain injury via various downstream signaling pathways, such as PI3K/Akt, mitogen-activated protein kinase (MAPK), and sirtuin 1. Its mechanisms may include inhibiting immune cell activation and inflammatory thrombosis and exerting antioxidative, anti-inflammatory, and anti-apoptotic effects. It also involves protecting the blood-brain barrier, regulating ion channels, reducing cellular excitatory glutamate toxicity, and ameliorating cognitive impairment.^[12,13]^

Kaempferol may play an anti-apoptotic role by upregulating the brain-derived neurotrophic factor-TrkB-PI3K/Akt pathway.^[14]^ It can protect against oxygen-glucose deprivation/R-induced ferroptosis via activating the nuclear factor erythroid 2-related factor 2 (Nrf2)/SLC7A11/GPX4 axis.^[15]^ Evidence shows that kaempferol mitigates neuronal attrition and glial cell activation in IS tissues, reduces COX-2 inflammatory protein expression, and suppresses the toll-like receptor 4/MyD88/nuclear factor kappa-light-chain-enhancer of activated B cells (NF-κB) pathway. It also decreases myeloperoxidase activity and peripheral neutrophil counts, curtailing neutrophilic aggregation and infiltration in compromised cerebral regions.^[14]^

Luteolin enhances cell viability and suppresses apoptosis through dual pathways: MMP9 inhibition coupled with PI3K/Akt signaling pathway activation.^[16]^

Isorhamnetin reduces infarct volume and caspase-3 activity; it also promotes neurological recovery. In experimental stroke models, it attenuates cerebral edema and improves blood-brain barrier function by increasing tight-junction protein expression. It activates Nrf2/heme oxygenase-1, suppresses inducible nitric oxide synthase/nitric oxide, and reduces malondialdehyde and 3-nitrotyrosine formation in the ipsilateral cortex. It inhibits myeloperoxidase activity and significantly decreases the protein concentrations of inflammatory cytokines (interleukin-1 Beta, interleukin-6, and tumor necrosis factor alpha) in the ipsilateral cortex.^[17]^

FMN provides IS resistance by attenuating the B-cell lymphoma 2-associated X protein/B-cell lymphoma 2 ratio and activating the PI3K/Akt pathway, with anti-apoptotic effects and reduced tumor necrosis factor alpha production. It upregulates βIII-tubulin and GAP43, inducing neuronal differentiation, and significantly augments nerve growth factor and brain-derived neurotrophic factor expression, enhancing synaptic plasticity for neurological restoration.^[18,19]^

Multiple studies confirm that β-sitosterol has strong antioxidative, antihyperlipidemic, anti-inflammatory, and antitumoral properties, especially in the central nervous system (CNS).^[20]^ One study found that it can boost hypoxia-injured endothelial cell activity by reducing oxidative stress.^[21]^ It may treat IS by inhibiting neuronal cholesterol overload, endoplasmic reticulum stress, and apoptosis pathways.^[22]^

Baicalein has multifaceted roles, including antioxidant, anti-apoptotic, and anti-inflammatory properties, as well as protective functions against excitotoxicity.^[23]^ It protects mitochondrial integrity, enhances neuroprotective agents in neurons, and promotes adult neurogenesis. It affects cerebral insults, indirectly modulates microglia polarization in stroke, and promotes an anti-inflammatory state by attenuating toll-like receptor 4/nuclear factor kappa-light-chain-enhancer of activated B cells and reducing signal transducer and activator of transcription 1 phosphorylation.^[24]^ It has also been found that baicalein reduced Nrf2 and adenosine monophosphate-activated protein kinase levels and protected the rat brain from ischemia/reperfusion injury, showing neuroprotection via downregulating NF-κB, lectin-like oxidized low-density lipoprotein receptor 1, and the adenosine monophosphate-activated protein kinase/Nrf2 pathway.^[25]^

Although many identified compounds (e.g., quercetin and kaempferol) pass the OB filter, poor OB and the presence of the blood-brain barrier limit their therapeutic efficacy. Some scholars have proposed that compounds can be delivered to the injury site through targeted nanosystems to enhance the therapeutic effect. Alternatively, the basic structure of a compound can be altered to improve its pharmacokinetics and neuroprotective capabilities.^[12]^ Furthermore, the neuroprotective effects of these flavonoids may be mediated through indirect mechanisms, such as modulation of peripheral inflammation, oxidative stress, and gut-brain axis communication, which can influence CNS pathology without requiring the compounds to cross the BBB in significant amounts.

### 4.2. PPI network, GO, and KEGG analysis

Top targets (TP53, JUN, AKT1, MAPK1, MYC, and ESR1) in the PPI network were hub genes. Combining GO and KEGG analyses and a literature search, BYHWD may protect IS patients through the predicted regulation of processes such as apoptosis, proliferation, differentiation, cell survival, growth, angiogenesis, metabolism, regulation of astrocyte-associated water-sodium balance, anti-inflammatory actions, and others.

TP53, a regulator of cellular processes, is involved in cell cycle arrest, DNA repair, apoptosis, and senescence. Posttranslational modifications of p53 under cellular stress determine its location, duration, and apoptotic ability. After IS, p53 quickly accumulates in affected neurons, promoting neuronal programmed cell death via transcriptional-dependent and -independent mechanisms.^[26]^

In the activator protein 1 complex, JUN is involved in various cell activities – proliferative factors, apoptotic processes, survival mechanisms, neoplastic transformations, and architectonic tissue structuring.^[27]^ In intracellular signaling, c-Jun N-terminal kinase downregulates hypoxia-inducible factor-1α, accelerating neuronal senescence under hypoxia.^[28]^ c-Jun N-terminal kinase activation and c-Jun phosphorylation enhance SF-1 transcriptional activity via cyclic adenosine monophosphate signaling, promoting CYP11A1 gene expression and exerting anti-inflammatory effects.^[29]^

AKT kinases (AKT1, AKT2, and AKT3) play a key role in processes such as metabolism, cell proliferation, intracellular survival mechanisms, cell growth, and angiogenesis-related activities. Neuroligin 3 activates Gαi1/3-Akt signaling, protecting neurons from ischemic-reperfusion injury.^[30]^ The PI3K/Akt pathway, a major cell-survival pathway, is related to IS. Previous studies have shown that AKT1 phosphorylation at Ser473 is linked to neuronal survival in CNS ischemic pathologies.^[31]^

MAPK1, a constituent of the MAP kinase family, is encoded as part of this collective. MAPKs (serine-threonine kinases) are key mediators of cellular responses to external stimuli through signal transduction. Also called ERKs, they act as focal points for biochemical signaling in various cellular processes such as proliferation, differentiation, transcriptional regulation, apoptosis, and development.^[32]^

MYC activates growth-related genes and associates with the VEGFA promoter for angiogenesis.^[33,34]^ N-myc downstream regulated gene 2 is an endogenous neuroprotector and a target for ischemic brain edema, regulating astrocyte water-sodium balance.^[35]^

ESR1 is also known as estrogen receptor alpha. Studies have shown that 17β-estradiol (E2) protects the brain from stroke by reducing neuronal apoptosis, exerting anti-inflammatory effects, and promoting repair of the ischemic brain.^[36]^

Although evidence in the literature supports the neuroprotective potential of each of these compounds, the actual bioavailability, pharmacokinetics, and synergistic effects of these compounds as part of a complete BYHWD formulation still need to be determined through in vitro and in vivo studies.

Concomitantly, GO BP analysis showed that transcriptional regulation, signal transduction, apoptosis, cell proliferation, and response to xenobiotic stimuli were significantly implicated in IS pathology. KEGG secondary enrichment analysis revealed that target genes were mainly enriched in the top 5 categories: signal transduction, cancer overview, endocrine system, immune system, and cardiovascular disease. The classical top 10 third-level pathway is PI3K-Akt, which regulates inflammation, oxidative stress, apoptosis, autophagy, and vascular endothelial homeostasis after IS.^[37]^

Due to the multi-target nature of BYHWD in treating diseases, the sources and time nodes for obtaining ingredients and disease target data are different. Additionally, the methods and quantities used for screening core targets vary, so the key targets finally obtained are often not identical. However, there is considerable overlap in the metabolic pathways involved in the research, such as the PI3K-Akt signaling pathway, MAPK signaling pathway, NF-κB signaling pathway, TNF signaling pathway, P53 signaling pathway, HIF-1 signaling pathway, and nucleotide-binding oligomerization domain-like receptor signaling pathway.^[9,11,38,39]^ This is consistent with the pathways involved in the top 10 pathway diagrams we obtained through KEGG analysis.

### 4.3. Molecular docking

By carefully observing the molecular docking results, it can be seen that, in addition to the lower binding between MYC and beta-sitosterol, TP53, JUN, AKT1, MAPK1, MYC, and ESR1 have better binding capabilities with the 8 core compounds: quercetin, kaempferol, luteolin, 7-O-methylisomucronulatol, isorhamnetin, FMN, beta-sitosterol, and baicalein. This finding supports the hypothesis that the sequelae of IS may be improved by regulating inflammation, oxidative stress, apoptosis, proliferation, differentiation, autophagy, cell survival, growth, angiogenesis, and vascular endothelial homeostasis. These core effective components are expected to provide valuable references for the development of new drugs for the treatment of sequelae following IS.

### 4.4. Limitations

We explored the key components, targets, and pathways of BYHWD in the treatment of IS through network pharmacology analysis. However, there are still several shortcomings. First, database-driven target predictions rely on similarity algorithms and may generate false-positive associations, particularly for compounds with promiscuous binding profiles or targets with broad substrate specificities. Second, the data are derived from databases, so some components or targets may remain undiscovered and thus were not included in the analysis. Compared with analysis based on high-performance liquid chromatography quadrupole orbitrap mass spectrometry, the obtained drug components may better reflect real-world scenarios.^[40]^ Third, although we have made conjectures about the functions of key targets and core active ingredients by reviewing the literature, as a TCM compound, the true mechanism of action of BYHWD needs to be further verified through in vivo and in vitro experiments. Fourth, we compared the central genes identified by CytoNCA with those identified by cytoHubba (using the MCC method, version 0.1) and the MCODE plug-in (version 2.0.3; Bader Lab, University of Toronto) in Cytoscape. We found that the overlap rate was low (Supplementary Table S14, Supplemental Digital Content 14). This may be related to our excessive filtering conditions and differences in the evaluation criteria of node importance among different topological centrality algorithms. These findings also reflect the complexity of the PPI network structure. Although the robustness of the overlap is moderate, there is partial overlap among the top-ranked genes, and most of the ligands we selected for molecular docking verification are overlapping target proteins. Finally, further studies on individual compound combinations and pathway inhibition experiments are needed to clarify synergy and quantitative contribution claims.

## 5. Conclusion

In summary, the multifaceted bioactive constituents inherent in BYHWD have the potential to improve the clinical manifestations observed during both the recovery and residual stages of IS. This may be accomplished through mechanisms characterized by multi-target interactions and multi-pathway processes, as shown in relevant case studies and empirical data.

## Acknowledgments

We thank all authors for their contributions and support.

## Author contributions

**Conceptualization:** Tailai Shi.

**Methodology:** Tailai Shi.

**Funding acquisition:** Danna Chen.

**Formal analysis:** Zhaoqi Chen, Mengmeng Wang.

**Writing – review & editing:** Tailai Shi, Danna Chen.

**Writing – original draft:** Zhaoqi Chen, Mengmeng Wang.




























