# Supplementary material for: Elucidating the mechanism of Buyang Huanwu Decoction in the treatment of ischemic stroke: A network pharmacology and molecular docking study
Source: Medicine (Baltimore). 2026 Jul 17;105(29):e49736. doi: 10.1097/MD.0000000000049736 (PMC13384647; doi:10.1097/MD.0000000000049736)
Supplement: Supplementary file 2 [file medi-105-e49736-s002.docx]

**S 2.** Summary of Disease Targets in Ischemic Stroke: Raw Data.

| **Gene name** | **Gene name** | **Gene name** | **Gene name** | **Gene name** |
| --- | --- | --- | --- | --- |
| GLA | APP | COL3A1 | ITM2B | F2 |
| F5 | NOTCH3 | ADAMTS13 | HDAC9 | CDKN2B-AS1 |
| LDLR | ALDH2 | ABO | ZFHX3 | ACTA2 |
| PROCR | PDE3A | SLCO1B1 | COL4A1 | LOX |
| SMAD3 | ABCC1 | PRKG1 | TGFBR2 | C20orf181 |
| SH2B3 | PMF1-BGLAP | PATJ | CDK6 | CDKN1A |
| CDKN2C | CERNA3 | PRPF8 | DINOL | CHD3 |
| FAF1 | PMF1 | NLRP3 | APOA5 | AGBL1 |
| CRP | CYP2C19 | DAB1 | ACE | AGT |
| AGTR1 | EDN1 | ELN | ENG | FBN1 |
| FGB | FOXE3 | LRCH1 | TSPAN15 | ALOX5 |
| ALOX5AP | NAALADL2 | PCSK9 | SLC26A11 | F11-AS1 |
| APOB | TSPAN33 | APOE | IL1B | IL1RN |
| IL6 | IL10 | ILF3 | KCNK1 | KCNK3 |
| C10orf143 | LPA | LPL | MIR146A | MAT2A |
| MMP9 | MTHFR | MYH11 | MYLK | NINJ2 |
| NOS3 | SERPINE1 | PCDH7 | PCYT1A | PDE4D |
| PIK3CA | PIK3CB | PIK3CD | PIK3CG | PLAT |
| PLG | PON1 | PPARG | CASZ1 | KIF26B |
| AVP | PROC | SLC44A2 | PTGS2 | PTPRD |
| PTPRG | NTN4 | BDNF | ATXN2 | SMARCA4 |
| FUNDC2 | SON | STAT3 | TGFB2 | TGFB3 |
| TGFBR1 | TLR4 | TNF | VEGFA | VWF |
| MFAP5 | SLC2A10 | QRICH2 | RUNX1 | ALDH1A2 |
| HPS4 | COX7A2L | ADIPOQ | PLAA | ACVRL1 |
| CDC5L | EPO | HMGB1 | MMP3 | MIR499A |
| SELP | PLA2G7 | CYP4F2 | IGF1 | CXCL8 |
| IL17A | AQP4 | LTA | NFE2L2 | P2RY12 |
| EPHX2 | ESR1 | FGFR1 | MMRN1 | GABPA |
| HIF1A | IL1A | MIR126 | MIR145 | OLR1 |
| CCL2 | CXCL12 | SELE | CDKN2A | CETP |
| CYP11B2 | AGER | SIRT1 | ICAM1 | IL18 |
| ITGA2 | JAK2 | MIR223 | TNFRSF11B | TREM2 |
| PTGS1 | TGFB1 | CASP3 | PPIG | SIGMAR1 |
| CPB2 | ABCA1 | MTOR | GPX3 | IL4 |
| LGALS3 | MMP1 | MMP2 | NGF | GP6 |
| ABCB1 | PRKCH | MAPK3 | RETN | BRCA1 |
| THBD | TP53 | APLN | KALRN | KL |
| HDAC6 | CDKN2B | MTCO2P12 | COL4A2 | COX8A |
| PARP1 | CSF3 | CST3 | CYP4A11 | ENO2 |
| F3 | FABP4 | SIRT3 | ALOX15 | ANGPT1 |
| NRG1 | HMOX1 | HSPA4 | APOC3 | IFNB1 |
| ITGA2B | ITGB3 | MALAT1 | KLK1 | KNG1 |
| LCN2 | LEP | MIR122 | MIR143 | MIR149 |
| MMP12 | MPO | COX2 | SERPINC1 | NEFL |
| NOS2 | NPY | PON2 | PPARA | CHDH |
| MAPK1 | REN | SPP1 | TRAF6 | UCP2 |
| VKORC1 | CAV1 | CBS | IL33 | GDF15 |
| CD40 | DNM1L | CDK5 | TRIB1 | KLF2 |
| ADAMTS7 | SERPINA3 | CX3CR1 | CYBA | CYP2C9 |
| CYP2J2 | DPP4 | EDNRB | F2R | F11 |
| F13A1 | FGA | SIRT2 | POLDIP2 | GRIN2A |
| GSK3B | HFE | HLA-DRB1 | APOA1 | IL6R |
| IL13 | LGALS1 | LGALS2 | LIF | LTC4S |
| MIR130A | MIR137 | MIR155 | MIR19A | MIR21 |
| MIR210 | MIR30A | MAP3K5 | MIF | MMP8 |
| MMP10 | COX1 | NOS1 | P2RY1 | ANGPTL4 |
| TNFRSF12A | RNLS | MEG3 | SELENOS | PTX3 |
| BCL2 | S100B | ACSM3 | CCL11 | SELPLG |
| SLC1A2 | SLC25A1 | BSG | CCR2 | XBP1 |
| XRCC1 | CAD | SNHG12 | TP63 | SOCS1 |
| KCNK17 | NTN1 | ROCK2 | PTGES | CD40LG |
| CELSR1 | ARHGEF10 | MIR874 | OCLN | MIR499B |
| PPIF | PGR-AS1 | NAMPT | CBSL | AHSA1 |
| SLC17A3 | HPSE | RUVBL2 | CHI3L1 | HSPA12B |
| ADD1 | RBM45 | ADM | CPOX | CRK |
| MAPK14 | CTLA4 | ADRA2B | ADRB2 | CYP1A1 |
| CYP2B6 | CYP2C8 | CYP3A4 | CYP3A5 | NQO1 |
| AGTR2 | APLNR | ECE1 | EDNRA | EGR1 |
| AHSG | MLKL | EPHA4 | EPHB2 | ESR2 |
| ALB | EZH2 | F12 | FCGR2A | FGFR4 |
| FGG | DKK1 | RCOR1 | SNHG1 | ALOX12 |
| POU2F3 | RNF19A | GCG | GFAP | GJA4 |
| IL37 | ANGPTL3 | GLP1R | GPER1 | GRIA2 |
| GRIN1 | NR3C1 | CXCL2 | HABP2 | HDAC2 |
| CFH | HGF | HSPA1A | HSPA1B | HSPB1 |
| HSPB2 | IFNG | APOH | IL9 | IL15 |
| IMPA2 | AQP9 | IRF4 | AR | JUN |
| KCNJ13 | KCNQ1 | ARG1 | FADS1 | LLGL2 |
| LTA4H | MIR106B | MIR107 | MIR125A | MIR132 |
| MIR134 | MIR150 | MIR15A | MIR181C | MIR27B |
| MIR29B1 | MIR29B2 | MIR17HG | TNFSF12-TNFSF13 | ARSA |
| MIP | ACHE | MMP7 | MIR335 | MTR |
| MTRR | NUBP1 | ATM | NGFR | NOTCH1 |
| NPPA | NPR3 | NTF3 | DUOX2 | CKLF |
| CLEC1B | PDE4A | SIRT6 | SERPINF1 | PIN1 |
| PLA2G1B | PLA2G2A | PLAU | SERPINF2 | DUOX1 |
| PPARD | PPID | TUG1 | MARCHF1 | MAPK8 |
| PROS1 | PSMA6 | ACKR3 | PTEN | PTGIS |
| MIR494 | NGB | CXCL16 | RAC1 | MOK |
| ACE2 | RBP4 | BCL2A1 | RELA | HPSE2 |
| GAS5 | SGK1 | RTN4R | SLC5A2 | SLC9A1 |
| SLC12A2 | SST | STC1 | SULT1E1 | SYK |
| TBXAS1 | HNF1A | TGFA | TSPO | TIMP2 |
| TLR2 | TNFSF4 | TXN | VCAM1 | TRPV1 |
| CACNA1A | CXCR4 | AIMP2 | NEIL1 | NR4A3 |
| PDGFD | COASY | COL18A1 | PLA2G6 | ULK1 |
| TSLP | BECN1 | RIPK1 | TNFSF12 | HSPB3 |
| MCU | TIMD4 | SLC33A1 | CD14 | COX5A |
| GRAP2 | ABCG2 | GSTO1 | AIM2 | EIF2AK3 |
| CARTPT | ABCG1 | CD69 | SEMA3E | MAFB |
| CDC42 | ZGLP1 | MIR298 | MIR922 | KIR2DS2 |
| DEFB4B | MIR1306 | MIR1203 | MIR664A | HOTTIP |
| MIR4437 | MIR4669 | MIR4656 | NR1H3 | EDIL3 |
| PTCSC3 | CDH13 | EBI3 | EMSLR | LOC101929707 |
| CDKN3 | IRF9 | ERICD | ATG7 | AGR2 |
| GNLY | SORBS1 | CAMKK2 | CXCR6 | MASP2 |
| TRAF3IP2 | NES | PLK2 | ALDH1L1 | LILRB1 |
| RNPS1 | FASTK | PDIA5 | STIP1 | COPS5 |
| RIPK3 | LOC110673971 | DUSP14 | CAPN10 | CHIT1 |
| TREX1 | PARK7 | VSIG4 | ACOT7 | MGLL |
| CHRNA4 | TRIM9 | CIRBP | H3P7 | ADCYAP1 |
| LRG1 | CYP2R1 | ACOT4 | CCR5 | FOPNL |
| TAF8 | UBR3 | COMT | CRHR1 | CRMP1 |
| DEFB104A | TRPM6 | SRXN1 | CRYZ | CSF2 |
| TTC7B | ADRA1A | CILP2 | NKX2-5 | HJV |
| CTNNB1 | ADRA2A | ARL6IP6 | RMDN2 | ADRA2C |
| ADRB1 | C5orf38 | CYP2B7P | CYP2D6 | CYP2E1 |
| CYP24A1 | DECR1 | DEFB1 | DEFB4A | DLD |
| DLG2 | DVL1 | EDN2 | EDN3 | EEF1A1 |
| EFNB2 | CELSR2 | EGR2 | EGR4 | RMST |
| A2M | ELK1 | EPAS1 | ERCC4 | ETS1 |
| ETV3 | ABCD1 | F7 | F8 | F9 |
| FBLN1 | FCGR3A | FCGR3B | FCN1 | FGF1 |
| FGF2 | ABCD2 | FGF13 | VEGFD | MRAS |
| VASH1 | NTNG1 | CARD8 | ATF6 | FOXF2 |
| FAIM2 | FOXO3 | MCF2L2 | FLG | TBC1D9 |
| MLC1 | ARC | CLEC16A | FMO3 | CRTC1 |
| NCS1 | TRS-AGA2-3 | FOLH1 | SPIDR | FOS |
| MMD | MAPK8IP2 | PADI4 | SMUG1 | DAPK2 |
| PLA2G15 | BCL2L13 | ABCA4 | PANX1 | G6PD |
| PRDX5 | HECTD1 | GALNT2 | HSPA12A | FAM98A |
| GAP43 | IS1 | COPD | GATA1 | GATA3 |
| GCH1 | GCKR | SLC17A5 | NPAS4 | GEM |
| HAVCR1 | GGCX | GHRH | GJA1 | NAAA |
| IL17B | GCLC | STK39 | GLI2 | HTRA2 |
| GNAS | GNB3 | GOT2 | GPI | H19 |
| ANGPT2 | SNHG15 | ANK1 | XKR6 | GPR37 |
| GPR42 | GPT | GPX1 | REM1 | GRN |
| GRIK2 | ANPEP | GRIN2B | GRIN2C | GRINA |
| SETD2 | GRM1 | GRM2 | CXCL1 | GSTM1 |
| GSTT1 | UBQLN1 | NOP53 | ANXA1 | HADHA |
| HCRT | HDLBP | HK2 | ACACA | HLA-B |
| HLA-C | HLA-DQB1 | HLA-G | AOC2 | NR4A1 |
| HP | MMAB | SERPINA9 | HES1 | BIRC5 |
| HSPG2 | HTR1A | HTR2A | FFAR4 | C1QTNF9 |
| ID2 | IFIT3 | ACTBL2 | APOD | MAGEB5 |
| IGF1R | IGFBP3 | IKBKB | IL2 | IL4R |
| IL5 | IL7R | AQP1 | IL10RA | IL10RB |
| IL16 | TNFRSF9 | INSRR | IRAK1 | IRS1 |
| ABCC6 | ITGB2 | JAK3 | JUNB | AREG |
| PEAR1 | KDR | KIR2DS1 | KIR3DL1 | KIR3DL2 |
| KRT18 | C11orf96 | TSPYL6 | LAD1 | LAMA5 |
| LAMC2 | LAMP2 | RPSA | LCP1 | LEPR |
| LGALS9 | LIG4 | LIMK1 | LOXL1 | LRP1 |
| LRP6 | LINC01194 | CIMT | MIRLET7B | MIRLET7E |
| MIRLET7I | MIR106A | MIR125B2 | MIR129-2 | MIR130B |
| MIR140 | MIR144 | MIR152 | MIR17 | MIR185 |
| MIR192 | MIR195 | MIR199B | MIR200C | MIR206 |
| MIR212 | MIR216A | MIR219A1 | MIR22 | MIR221 |
| MIR30D | MIR31 | MIR34A | MIR93 | ARRB2 |
| MAPT | MBL2 | MDK | MDM2 | CIITA |
| NR3C2 | MOG | MRC1 | MIAT | POTEKP |
| ZFAS1 | MIR326 | MSD | MSR1 | PLF |
| MTAP | CYTB | MUSK | MMUT | MVK |
| ATF3 | ATF4 | NDUFC2 | NFKB1 | NM |
| NNMT | NPC1 | NPPB | NTF4 | MIR422A |
| MIR424 | OATP1 | OGG1 | OPRK1 | OPRM1 |
| P2RX4 | P2RX7 | P2RY2 | DEFB104B | PAEP |
| NOX4 | SERPINB2 | IL20 | PAPPA | AK3 |
| PC | PDE11A | FOXP3 | SERPINA5 | GAL |
| MLXIPL | RMDN1 | IRAK4 | PCSK1 | MZB1 |
| TLR7 | TLR8 | SPTBN5 | MS4A4A | ZC3HC1 |
| PDGFRB | PECAM1 | RTEL1 | STK26 | ASIC5 |
| SERPINE2 | SERPINI1 | PIK3R1 | PITX2 | PKM |
| PLIN1 | PLP1 | ACP5 | PNN | NANS |
| POR | POU2F1 | TOMM7 | DLL4 | EGLN1 |
| UGT1A1 | PPBP | ROPN1 | PPIA | TRPM7 |
| RASIP1 | TMEM132A | ANO1 | RMDN3 | IMPACT |
| MIR449A | PRH1 | PRH2 | KIF16B | SYBU |
| PRKAR1A | VPS35 | PRKCA | PRKCD | RNF130 |
| PRKD1 | ACSS2 | APOM | LRRC8A | MASP1 |
| MMP26 | B2M | SPHK2 | ANKS1B | PSMB9 |
| PDXP | RTN4 | PTGDR | PTGER2 | MRS2 |
| PTGIR | MIR363 | PTHLH | MIR451A | MIR410 |
| MIR497 | NDRG3 | NDRG2 | MIR503 | NLN |
| TAOK1 | SEMA6A | PTMA | PTMAP4 | RNF213 |
| PRX | HAMP | BARD1 | NLRC4 | IL21 |
| RAPSN | RARRES2 | TRPV4 | RENBP | ACTB |
| RGS7 | RNASE3 | PROK2 | RPS20 | RTN1 |
| SORT1 | S100A9 | S100A11 | SAA1 | SAA2 |
| TSPAN31 | CEACAM1 | BHMT | CCL5 | CCL19 |
| CCL23 | CX3CL1 | SDC1 | SRR | NEUROG2 |
| SELL | CRLF2 | MOAP1 | POTEM | MAP2K4 |
| SNHG6 | ABCG8 | ITSN1 | SHC1 | GORASP1 |
| SHH | SHMT1 | SMURF2 | NMNAT1 | MARCHF7 |
| AGXT2 | PINK1 | ACD | NBEAL1 | MARCKSL1 |
| WNK1 | WNK3 | SLC6A4 | SLC6A11 | SLC6A13 |
| SLC12A3 | SLC19A1 | SLC20A2 | SLCO1A2 | CYP4F12 |
| SMN1 | SMN2 | SNCA | SNRNP70 | MIR544A |
| MIR487B | SOD2 | SOD3 | BNIP3L | SOX2 |
| SREBF2 | SRF | BRCA2 | SSTR4 | STAR |
| STAT4 | STAT5A | STAT5B | SCARNA6 | STIM1 |
| BRS3 | ABCC8 | SYT1 | TAC1 | ADAM17 |
| BTF3P11 | MIR532 | MIR605 | MIR608 | MIR618 |
| MIR638 | ZEB1 | TCN2 | TEK | TERC |
| TERT | TFDP1 | TFPI | TGM2 | THBS1 |
| TIMP1 | TLR3 | ACTG1 | SERPING1 | TLR5 |
| TM7SF2 | CLDN5 | TNFAIP3 | TNFRSF1A | TNNI3 |
| TRC-GCA24-1 | TRAF3 | ACTG2 | TRH | TRPC6 |
| TRPM2 | TRPS1 | C5 | TTR | UBE2L3 |
| UCHL1 | UGCG | UGT2B4 | UMOD | VPS51 |
| USF1 | UTRN | VASP | VDR | VEGFB |
| VIP | VSNL1 | VTN | WRN | CACNA1C |
| ZNF208 | MANF | REEP5 | MUL1 | ADIPOR2 |
| TNFAIP8L2 | DHX40 | TFPI2 | EHMT1 | ARHGEF5 |
| MAP9 | ERMP1 | WLS | NAA25 | CALM1 |
| ZC3H12A | FLAD1 | KCNIP4 | CALR | CALU |
| AKAP1 | RNF146 | QTRT1 | BRAP | CASP1 |
| PLVAP | BCO2 | TSPAN10 | CASP7 | CASP8 |
| MINDY4 | KLF11 | NTNG2 | KDM2B | ACCS |
| CAT | PSRC1 | GPR65 | IL17RC | ORAI1 |
| APOL1 | DOCK7 | PIAS1 | DENR | MADD |
| TNFSF11 | PLPP3 | PDE5A | TNKS | ABCC3 |
| TNFSF14 | TNFSF10 | TNFRSF11A | IL18RAP | IL18R1 |
| NR1I2 | SQSTM1 | OTULIN | USP14 | LPAR2 |
| IL1RL1 | XPR1 | METTL18 | NOG | MSC |
| SPECC1 | DNER | ASIC3 | CD163 | ZFYVE9 |
| CD28 | MUC16 | FADS2 | HOMER2 | HOMER1 |
| FHL5 | CD34 | CD36 | ADAMTS4 | ADAMTS3 |
| GTPBP1 | SNCAIP | PDE4DIP | CD74 | HDAC4 |
| ACYP2 | XYLB | SLC23A2 | CCS | MT-TL1 |
| GP1BA | BDNF-AS | ADA2 | MT-CYB | MT-ND1 |
| LOC132090228 | MT-TK | MT-TS1 | MT-ND5 | HTRA1 |
| ACSL4 | MT-CO3 | POLG | MT-TI | MT-TL2 |
| MT-CO1 | POLGARF | MT-TP | MT-ND6 | MT-ND4 |
| MT-ATP6 | MT-CO2 | INS | ATRIP | ATRIP-TREX1 |
| HOTAIR | RPL36A-HNRNPH2 | GUCY1A1 | SCN5A | KAT6B |
| LMNA | DIAPH1 | HBB | SLC2A1 | SOD2-OT1 |
| TNNT2 | PF4 | RNF213-AS1 | PSEN1 | SOD1 |
| KCNJ5 | ATP1A2 | PRNP | CREB1 | MAP2 |
| GBA1 | PROZ | PIK3C2A | CKB | SCN1A |
| THPO | CYCS | NR4A2 | MECP2 | TTN |
| HMGCR | KRIT1 | ENPP1 | AKT1 | F10 |
| FLNA | DARS2 | CTSA | SULT1A3 | MIR29A |
| GDNF | MIR146B | TMX2-CTNND1 | MIR142 | MPL |
| MIR98 | MIR9-1 | MIR124-1 | MYBPC3 | CKM |
| ATP1A3 | CCM2 | PKD1 | SCN8A | MIR199A1 |
| PIGQ | WDR37 | GHRL | LIPC | MIR342 |
| LOC106627981 | TET2 | LINC01672 | DEPDC5 | PVALB |
| MIR133B | UBE4A | GJA5 | SMAD5-AS1 | GATA4 |
| ABCC9 | GSR | SLC1A3 | MBP | JAG1 |
| SETD1B | MYH7 | PWAR1 | TSHR | APTX |
| WWOX | CORIN | HEY2 | MB | SMAD4 |
| ADORA1 | ARMS2 | MYMY1 | CPA6 | KCNT1 |
| KMT2E | NBEA | TTC21B | OTUD6B | TANC2 |
| CEP128 | ARFGEF1-DT | AIF1 | IL1R1 | ENTPD1 |
| KCNE2 | FABP2 | TF | COL5A1 | ANXA5 |
| BMP7 | LINC02605 | MYH9 | FLT1 | SNHG14 |
| BAX | INSL6 | LOC106099062 | LOC107133510 | CSF1 |
| XDH | DNAH8 | GATA6 | PMM2 | MYL4 |
| CNTF | MIR455 | TH | PDCD10 | CCL3 |
| PVT1 | SERPINA1 | ACTC1 | PRL | MIR125B1 |
| CP | GAPDH | MYH6 | MFN2 | FAS |
| PXDN | CAMK2G | TCF7L2 | RBFOX3 | EPOR |
| KCNA5 | RPS27A | ADA | SMAD2 | IRX2-DT |
| SCN2B | SCN1B | CHAT | COL4A5 | POMC |
| C3 | MEN1 | NEAT1 | COG2 | SCN3B |
| COL4A4 | CELA2A | SDHB | ELP1 | SCN4B |
| MIR338 | MTX2 | MIR27A | HTR3A | MIR103A1 |
| SERPIND1 | IDO1 | SDHA | SRC | MGP |
| CD63 | HNRNPH2 | TJP1 | CS | MIR188 |
| PLOD1 | ITIH4 | CPS1 | KCNJ2 | HSPA8 |
| SCNN1A | NF1 | ATG9B | MC4R | TIMP3 |
| CSF1R | PRTN3 | CBL | DCX | MIR574 |
| PKD2 | KCNJ11 | CLU | KLKB1 | KRAS |
| FLVCR2 | BGLAP | FOXP2 | CALCA | COL1A2 |
| MIR181A1 | SNORD15A | CASP9 | TAB2 | LOC129992813 |
| SHBG | DFFA | PGF | F13B | SLC6A3 |
| KCNQ1OT1 | POLG2 | PPARGC1A | LINC01394 | MT-ATP8 |
| MIR204 | SMARCAL1 | DMD | KCNH2 | BACE1 |
| MAOB | PHACTR1 | COL1A1 | HULC | BRCC3 |
| IL5RA | LIPA | EPHB4 | INF2 | EMX2OS |
| PKD1-AS1 | LINC01227 | ADRB3 | LTF | ITGB1 |
| PCNT | CNR1 | SLC25A24 | MIR224 | TRA-TGC7-1 |
| AARS2 | SAMHD1 | NDE1 | MIR24-1 | PALLD |
| RYR1 | NUP155 | VDAC1 | MIR25 | ACTA2-AS1 |
| MIR34C | MIR15B | XIST | ACAN | ELANE |
| PXDNL | ZMPSTE24 | CASP2 | HBA1 | COL2A1 |
| KCNJ3 | BCL2L1 | FN1 | GNB2 | CKMT2 |
| NTS | NCF1 | MIR23A | GH1 | MIR486-1 |
| PDGFB | ITGAM | GAA | PSEN2 | IFIH1 |
| DLG4 | CHGA | LOC126859827 | FGF23 | FABP3 |
| TRE-TTC3-1 | IGFBP1 | KCNE1 | NAGA | MIR320A |
| BRAF | DNTT | GPX7 | GPX2 | GPX5 |
| GPX8 | GPX6 | NTRK2 | ITLN1 | GDF2 |
| RAB4B-EGLN2 | MIR208A | IL1F10 | IFNA2 | UCN |
| AMPD1 | RBPJ | PRKN | MIR383 | MIR4793 |
| ADAMTSL1 | MIRLET7C | EGF | NRP1 | CAPN3 |
| SCNN1B | PPOX | RAF1 | CKMT1B | THSD4 |
| MTERF1 | PTH | KCNMA1 | GRIA1 | TSPAN2 |
| MT-ND2 | CDH5 | WFDC21P | CSRP3 | TPM1 |
| PRKAG2 | CYP17A1 | LOC110006319 | NPPA-AS1 | ASIC1 |
| TBX20 | KITLG | JAK1 | CYBB | ASTN2 |
| LOC130060044 | ODC1 | SCARNA5 | ST2 | NDUFS4 |
| ATFB5 | MAOA | KMO | GATA5 | PDGFRA |
| LOC110806262 | UCP3 | MROS | FMR1 | VHL |
| YY1AP1 | ALDH7A1 | MT-RNR1 | PTPN22 | C4B |
| GGT1 | MMP13 | PDGFA | DNMT3A | THSD1 |
| AHDC1 | MIR128-2 | DSP | AOC3 | COL5A2 |
| TTN-AS1 | DNAH5 | MIR222 | TMEM126B | ADAMTS2 |
| MIR30B | FTO | CYP1B1 | NT5E | KIF6 |
| SCGB1A1 | MEFV | STN1 | MIR196A2 | SHANK3 |
| CSF3R | CCR3 | BAD | SRRT | DRD1 |
| GRM7 | CRYAB | MYL3 | ZCCHC14 | BMP6 |
| PWAR4 | ATP12A | ATP4A | POU4F1 | CYP11B1 |
| FGF21 | GCK | IL2RA | OPN4 | LINC-ROR |
| MIR423 | GC | EPRS1 | SCN2A | PRKCH-AS1 |
| TBX5 | DES | TLX1NB | IL3 | TNFRSF1B |
| SNHG16 | BLVRB | BCHE | MIR483 | KYNU |
| MIR30E | ITGAL | ADORA2A | NSD1 | IRF1 |
| MTHFD1 | TARDBP | TWIST1 | SREBF1 | CCL4 |
| SFTA3 | DGUOK | MTTP | GRM8 | MYL2 |
| ATRX | TBXA2R | FOXC1 | TNXB | PRDM16 |
| TUBB3 | LOC110121269 | TGFBR3 | RYR2 | NPHS2 |
| CLCN1 | LOC126862864 | MGR1 | RBM20 | MIR26B |
| SLC1A1 | MIR211 | GSTP1 | APOA4 | SLC25A44 |
| MYOC | INSR | MAML3 | ADK | MS4A2 |
| STX1A | LOC126862663 | HCCAT5 | GRM5 | SLCO1C1 |
| FBN2 | IL11 | MIR668 | SRFBP1 | GABRG2 |
| CTSD | MIR200B | SCN9A | FUS | PTPN11 |
| CALB1 | LOC126805877 | COLGALT1 | SLC22A5 | TCF7 |
| IFNA1 | CD79A | TFAM | MIR99A | VAMP8 |
| SGCD | RECK | HECTD4 | CRYBG1 | SYP |
| VIM | FNDC5 | FLNC | SLC22A4 | STING1 |
| MIR208B | DAOA-AS1 | CCN2 | JPH3 | MIR19B1 |
| LEPQTL1 | IRF5 | SPTB | SLC6A1 | CLCN6 |
| SOS1 | CRH | MIR182 | MT-RNR2 | IL1RAPL2 |
| LTB | TSC2 | STIN2-VNTR | KCNMB1 | CFLAR |
| BDKRB2 | LTBP1 | COX4I1 | MIR133A1 | CDKAL1 |
| TYMP | OIP5-AS1 | SUOX | WARS2 | DBH |
| MMACHC | DLL1 | DAPK1 | TPO | VCP |
| ACADS | CCR1 | TFRC | SLC2A4 | MYC |
| NRXN1 | IKBKG | AIFM1 | HSPD1 | AKR1B1 |
| S1PR1 | MIR148B | BMP2 | MIR16-1 | DNASE1 |
| MIR214 | GCDH | MIR590 | CMA1 | NPC2 |
| ALPL | MIRLET7G | KCNN4 | MIR124-3 | CLCN2 |
| TFB1M | PNP | SOST | TIGAR | SOCS3 |
| HEY1 | HSD11B2 | SP1 | DPAGT1 | IGF2-AS |
| ROS1 | CITED2 | DEFA1 | MAML1 | MAML2 |
| HPX | OTC | SLC6A2 | APOC1 | PON3 |
| IGF2BP2 | HTR2B | NHERF2 | AMD1 | MYH15 |
| SNX19 | CD177 | ICAM4 | HNF4A | HK1 |
| HCG27 | SLC66A2 | AHCY | DSG2 | ITGA4 |
| VIPR2 | GAD2 | C4A | LOC101448202 | SNHG8 |
| KIT | GJB1 | MIR193A | LDB3 | IL36RN |
| MIR320E | NTRK1 | IGF2 | MIRLET7F2 | FABP12 |
| USP8 | SCNN1G | SLC8A1 | ROCK1 | MIR365A |
| MIR4306 | CHUK | ARIH1 | LINC01438 | IL12A |
| SNCB | GLUL | DRD2 | SERPINA12 | PTGDS |
| HRES1 | MIR320D1 | PMM1 | NAGLU | LOC106799833 |
| MIR495 | CCK | RHOA | SAA4 | CHKA |
| IAPP | MEF2D | CD47 | HTR4 | LOC107988032 |
| ADAMTS1 | MYD88 | GAS6 | LCAT | ITGB6 |
| CYBRD1 | CXCL10 | SGCE | GRIA4 | EMILIN1 |
| MYH14 | DCN | HPS1 | TIE1 | BMP10 |
| UCA1 | POLR3F | NF2 | TMEM106B | C1QA |
| LOC654780 | APEX1 | NPR1 | FASLG | ALOX12B |
| CPT2 | CYP1A2 | SUN1 | SH3BP5-AS1 | ENSG00000260337 |
| TLL1 | RPL3L | MGAT5 | BCAN | SPATA13 |
| ABCB7 | PCCA | STRN | XK | DPM3 |
| DOCK8 | WNT2B | DENND11 | ALDH5A1 | GHR |
| KIAA0319L | MIR18A | EGFR | ANK3 | AP3B1 |
| HBG2 | FABP1 | MCF2L | VCL | THUMPD3-AS1 |
| DDAH1 | C1QTNF3-AMACR | ACP1 | SUPT3H | MIR382 |
| HCN4 | CLN3 | CD59 | GUSB | FGF5 |
| KLC1 | PNOC | NEXN | GYS1 | KCNQ1-AS1 |
| CACNA1H | CHD4 | MIR381 | IDS | GATM |
| PRRC2A | GSTO2 | FURIN | DNMT1 | ITPR1 |
| MIR10A | SMPD1 | CHRNB2 | SNORD118 | IGFBP2 |
| GRIK1 | NORAD | PKP2 | ADAR | KLF4 |
| LZTR1 | GSN | SETBP1 | FIP1L1 | NOD2 |
| CTSB | CACNA1D | PRCP | MYMY4 | PLA2G10 |
| CXCR2 | HLA-DRA | TNC | ABCB11 | CD274 |
| RNY3 | MAP1B | PLAUR | NEDD4L | ADORA3 |
| CHMP2B | SPSB4 | CSTB | HSPA1L | RNF217-AS1 |
| CHRNA7 | LTB4R | GAD1 | GRIA3 | H2AX |
| TYK2 | ANXA2 | MIR215 | CRY1 | RNASEH2C |
| HAND2 | BMPR2 | PRKAA2 | GABBR1 | GATAD1 |
| HBG1 | PCDH19 | CHRNA3 | CCL26 | CTF1 |
| RAI1 | HSPA5 | CACNA2D1 | DNAH11 | FHOD3 |
| HTR2C | GOSR2 | CFI | SLC22A3 | ANGPTL6 |
| ULK4 | RMRP | GAMT | SCN10A | HSD11B1 |
| MALT1 | SHOC2 | CYGB | DUSP22 | APOC2 |
| MAS1 | S100A8 | MPDZ | ADSL | ABAT |
| MKI67 | BGN | CHRNB4 | WDR12 | IL1R2 |
| RASA1 | MIR379 | ASPM | TBK1 | EPHA3 |
| TCF21 | NPHS1 | SCN3A | PRKACA | SOX17 |
| CCNH | MLYCD | IDH1 | ABCC5 | SLC24A3 |
| AKT2 | FIRRE | FOXF1 | COL6A3 | HNF1B |
| FST | SNHG5 | MME | TYRO3 | GLDC |
| SCGN | LOC126861856 | DPH1 | ARSB | MAPK10 |
| PLN | NRGN | PIGA | SLC12A1 | ITGAE |
| MIR181A2 | HRH3 | CALB2 | ORM1 | CFHR5 |
| EFNA1 | OXT | WNT3 | MIR5100 | SCARB1 |
| MIR100 | ITGAV | FHIP1A | SPATA31F1 | APCS |
| IDUA | CARD14 | MON1B | ICAM3 | CYP19A1 |
| MIR659 | BAG3 | KCNA3 | HIF3A | CALCRL |
| GALNT3 | SOAT1 | TOX | PSMC3 | MIR485 |
| LMX1B | LTBP2 | STAT1 | SERPINA7 | PRRT2 |
| FCAR | NPTX1 | DAXX | SLC39A7 | AQP10 |
| TAS2R50 | ENDOV | PTPRF | MIR665 | NAGS |
| CACNB4 | WT1 | MCCC2 | METTL3 | TPP1 |
| SLC12A4 | EIF2AK2 | DCC | KRT5 | SERPINB8 |
| SLC26A8 | P2RX6 | MLF1 | MCM10 | KRT74 |
| VTI1A | DMXL2 | PGLYRP2 | SGIP1 | HEATR5B |
| MYOM3 | TAF3 | FSTL4 | TMPRSS11B | WDR31 |
| FCRLB | OR2A25 | WDR55 | ZNF132 | LY6G5B |
| OR13G1 | LGALS14 | RPLP0P4 | MYPN | ITGA3 |
| SLPI | BTK | LPXN | MIR33A | HNRNPA1 |
| HNRNPA2B1 | LPAL2 | POLR3B | MIR181D | ASS1 |
| CDK5R1 | ANK2 | SIL1 | GCLM | SCN4A |
| TG | LBP | FALEC | PENK | NCAM1 |
| ITPR3 | MIRLET7F1 | PPT1 | SCIN | LAMA2 |
| TACR1 | GAS5-AS1 | SCN11A | MIR184 | MMP14 |
| RUNX2 | TRIM31 | SNHG7 | RCAN1 | ENOX1 |
| CD4 | WDR45 | INTS5 | CACNB2 | MAP4K4 |
| WNT9B | LRRC37A2 | BMPER | CTSK | ABL1 |
| SLC7A11 | SRSF3 | CFHR4 | MAPK8IP1 | CACNA1B |
| ADCY10 | TNFAIP6 | TNFSF9 | PGK1 | C9orf72 |
| ST7-OT3 | CYP11A1 | MAP1LC3A | DDC | ENPP3 |
| TRADD | RB1 | NT5C2 | IFNGR1 | CTNNA1 |
| ADAMTS9-AS2 | PDYN | HRAS | E2F1 | GP9 |
| PAFAH1B1 | PEMT | MIR139 | EYA1 | TCAP |
| SMAD9 | C3AR1 | CAPN1 | GALNS | MIRLET7D |
| GAS6-AS1 | POLR3A | SLC12A5 | DLX6-AS1 | SCN7A |
| ZPR1 | RASD1 | ALKBH8 | ERRFI1 | BCR |
| MCM3AP-AS1 | ITPK1-AS1 | C1QTNF3 | LOC102723566 | LOC126806446 |
| CXCR3 | LAMP1 | SFTPA1 | TFEB | MIR375 |
| MIR92B | HTT | SPAST | PAWR | PCNA |
| NKX2-1 | ARNT | CGAS | CDKL5 | PRKCE |
| ACTN2 | DHFR | MIR10B | PTPRC | DNAJC30 |
| KCNQ2 | BCL2L11 | MIR191 | PSAP | IVD |
| MVP-DT | FLT3 | TTF2 | RNASE2 | GZMB |
| LOC100507006 | TBX4 | ETFA | MIR374A | FOXO1 |
| CFTR | MAPK11 | MIR641 | BMP4 | SEMA3A |
| RAMP2 | FADD | ABCC2 | LAMA4 | IDE |
| PRDM8 | ALAS2 | ADAMTS18 | SNORD75 | KANK2 |
| NRIP2 | SUSD5 | MIR320B2 | EIF2B2 | RNF216 |
| MIR501 | OSM | ASXL1 | HNF1A-AS1 | MIR488 |
| ATP13A2 | SH3PXD2A | LIPG | BLK | ERN1 |
| MUC1 | PARP12 | GPATCH8 | TMCO6 | NLGN1 |
| P4HB | CNNM2 | MIR411 | COMP | MIR505 |
| MIR20A | ENPEP | PDCD1 | RET | MIR6796 |
| ITGA5 | DDX31 | SKI | SI | MGAM |
| MIR502 | ADCYAP1R1 | CACNA1S | SNAP25 | MIR7-1 |
| NOD1 | NEFH | GP5 | NLRP1 | USP48 |
| ASAH1 | HMBS | lnc-NTRK3-4 | VEGFC | NEU1 |
| CDH4 | CACNA1G-AS1 | MIRLET7A1 | MIR190B | GALC |
| H6PD | MIR885 | PDP1 | PGAM5 | P2RX3 |
| REG3A | UBE3A | CCL17 | TTLL5 | TMEM163 |
| SHISA6 | OBI1 | ZDHHC22 | LINC02113 | MIR361 |
| GLB1 | MIR3615 | CDH11 | RHO | CASQ1 |
| KCNA2 | EMD | SNORA68 | SNORD44 | HS3ST1 |
| LTB4R2 | MIR676 | MIR4446 | MIR345 | PTPN1 |
| KCNJ6 | DIO3 | HYOU1 | PTGER4 | F2RL3 |
| EPM2A | NHLRC1 | CD86 | TNIP1 | FABP5 |
| APC | DUSP13B | GBE1 | RPS15 | MRPS27 |
| THY1 | KCNQ4 | GABRA1 | LCMT1-AS2 | LINC01002 |
| CCND1 | DOCK8-AS1 | LOC126860552 | LOC130001437 | MIR99B |
| ATL1 | MIR127 | CCDC40 | IFNAR1 | ACADVL |
| CAMK4 | IL34 | HAVCR2 | CANT1 | CD68 |
| S100A1 | RAMP1 | ERCC6 | CNR2 | DLGAP2 |
| ADIPOQ-AS1 | AGA | SLC25A38 | FSTL1 | PCCB |
| IL23R | NCOR2 | ITPK1 | COL23A1 | OSGIN1 |
| PROM1 | GPT2 | ATP13A3 | LINC01001 | EPHX1 |
| ID3 | CYTOR | FGFR2 | MCEE | M6PR |
| TFAP2A | UTS2 | LINC01554 | PRKCB | PLA2G2E |
| EIF1AY | SERPINB9P1 | SGSH | RABEP2 | GABRB3 |
| IL2RG | PCSK2 | ATP10A | ACSS1 | CMC4 |
| PTPRB | ADH1C | NPPC | FBLN5 | ENO1 |
| FAS-AS1 | WNK4 | HHT4 | MET | CPT1B |
| LOC126860124 | CLN6 | PRKAA1 | TBX3 | BDKRB1 |
| ATP6AP2 | DOHH | ZBTB41 | LINC02145 | HBA2 |
| SNORD7 | ZEB2 | FBXW7-AS1 | SURF6 | FAM13A |
| MIR148A | SLC4A1 | SOCS2 | ADPGK | TAF1 |
| ADH1B | OGA | APOA2 | TAB1 | CCL7 |
| LRP5 | PTPRN2 | ZNF143 | lnc-TWIST1-1 | BAMBI |
| MAGI2 | CR2 | BACH1 | F2RL2 | HDAC1 |
| MIR199A2 | CTC1 | TNR | SVIL | SLC2A3 |
| SOX9 | MIR29C | lnc-ABO-33 | MIR181B1 | MIR602 |
| TREM1 | GRK2 | MAN2B1 | HLA-DQA1 | GPX4 |
| IL21R | RBP3 | DDIT3 | SLC16A1 | PDGFC |
| DKK3 | CNOT6L | FUCA1 | A4GALT | MCOLN1 |
| GBA2 | MIR766 | MED12 | LOC102724058 | LRRK2 |
| PLA2G3 | XIAP | PYGB | KCNA1 | MIR1283-2 |
| EP300 | PYGM | LTBP4 | EFEMP2 | SAP30 |
| SERBP1 | C14orf119 | LINC02908 | LINC02913 | MIR3161 |
| GUCY1B1 | SLC2A12 | TBX18 | ROCR | SLC49A3 |
| MIR3928 | CXCL13 | ST3GAL4 | LAMA1 | KIF5A |
| BMPR1A | RHD | ERAP1 | UBAC2 | KLRC4 |
| IL12A-AS1 | CACNA1F | AHSP | WRAP53 | BCL11A |
| MIR1226 | BIN1 | TEX41 | HDAC3 | PYY |
| PMFBP1 | FOXF2-DT | RPL21P81 | ENSG00000201451 | RF00017-8149 |
| SERPINA6 | TYMS | TLK1 | MIR4271 | ACTA1 |
| SYNGAP1 | FGF14 | PSMB7 | DNAJC6 | PTCSC1 |
| CCN1 | G6PC1 | OSMR | LOC113939944 | LOC130057019 |
| LOC126862124 | LOC130057352 | PLTP | FOXQ1 | LOC110973015 |
| WNT7A | ADGRA2 | SOX5 | CLN8 | NFKBIA |
| KLF3-AS1 | PNPLA2 | TOP1 | CAST | MIR498 |
| SORL1 | ACVR2A | CD55 | MLXIP | BMPR1B |
| RNASEH2B | APAF1 | GSDMD | MIR301B | FLNB |
| SLC17A7 | SLC17A6 | SAG | PTK2 | MIR302A |
| ENDOG | MOCS2 | GABBR2 | LGI1 | C1orf167 |
| TECRL | MIR340 | MIR1246 | STXBP5 | RGS5 |
| ANXA3 | CYP4V2 | ASXL2 | MIR551A | MIRLET7BHG |
| BABAM2-AS1 | MIR1263 | LRPAP1 | FMR1-AS1 | NBAT1 |
| PNKY | IGFBP4 | CNTNAP2 | FUT2 | GRM3 |
| MIR616 | OBP2B | NFIA | SMTN | CST2 |
| VEZF1 | IL20RB | SNHG11 | MYB | GYPA |
| KLF1 | MIPEP | HBS1L | DLX4 | HBD |
| HBE1 | SELENOI | GYPE | MIR3200 | HBB-LCR |
| LOC106099065 | FCP1 | ALCAM | SCUBE1 | ARMC5 |
| GLUD1 | SPARCL1 | S100P | TIPARP | KIF20A |
| IKZF2 | HSP90AA1 | RELN | CACNA1G | KCNQ3 |
| SV2A | CDH2 | AMBP | PPP1R1B | SPRED1 |
| ANKS1A | IGFBP7 | SLC39A8 | CA4 | MIR519D |
| MIR1258 | MIR299 | MIR576 | SMARCA2 | HPR |
| DUSP2 | TERF2IP | LINC00520 | MIR296 | HVCN1 |
| ITGAX | CDH23 | MIR3123 | CES1 | FGD5-AS1 |
| MIR330 | RAPGEF3 | SV2C | FLNC-AS1 | NPL |
| MIR496 | LEF1 | POMT2 | LAMA3 | SEMA3F |
| ACADM | SMAD1 | CD8A | KLHL3 | RPTOR |
| XKR9 | RNU6-1252P | IDH2 | KCNJ16 | MIR599 |
| ATP5F1A | MX1 | MIR24-2 | HADHB | PAX6 |
| MIR323A | MIR124-2 | CALM3 | IRAG1 | ACAA1 |
| NCSTN | APH1A | JMJD1C | CAV2 | MAPK9 |
| HCN2 | TAP2 | KCNK2 | TIMP4 | CHD8 |
| B3GLCT | SORD | CAV3 | TBP | CD46 |
| SMG6 | DNM1P37 | TAGLN | FES | PRANCR |
| HMGCL | STUB1 | USP36 | TLN1 | MOV10 |
| NEUROG3 | NOX1 | SEMA4A | CACFD1 | piR-59907-030 |
| ERVW-1 | NEURL1 | SLC25A13 | SORCS2 | LACTB |
| NSF | H3-3A | PHETA1 | SLIT2 | DYNC2H1 |
| OBSCN | MIR6165 | ALDH1L1-AS2 | RNU6-1 | SLC6A8 |
| KCNQ5 | SLC13A5 | PRKCZ | NCEH1 | TSC1 |
| SLC38A2 | KCNMB4 | KCNMB2 | FHL2 | SLC27A4 |
| PANX2 | AKAP7 | MIR431 | MIR1299 | MIR1182 |
| TSPAN9 | PSPH | GNMT | AKAP9 | RFC1 |
| MAP3K11 | DDIT4 | CAPN2 | HCP5 | KDM1A |
| SUV39H1 | CBX3 | CBX5 | CBX1 | H3-3B |
| H3C12 | H3C13 | H3C14 | APIP | LOC106029312 |
| DENND2B | ACTN1 | SCG2 | NID1 | TRIM29 |
| CSN1S1 | DPYSL2 | MIR151B | BAZ1A | TOLLIP |
| AP3D1 | CCT2 | UHRF1 | SLC7A8 | ZNF618 |
| OPRD1 | CLDN1 | MEF2A | MAPK7 | NFAT5 |
| MIR561 | FABP7 | DIO1 | SLC22A7 | STXBP1 |
| KCNJ10 | ADARB1 | SLC32A1 | IL2RB | MIR4443 |
| PRKCQ | ROR2 | CTBP1 | PML | PSMB4 |
| DOCK1 | EIF2AK1 | TP73 | PSMA4 | TFF1 |
| CDH15 | MCC | NDST1 | SBF1 | COLEC11 |
| MAPKAP1 | POLR1D | SEPTIN9 | USP20 | COX5B |
| PITRM1 | CAMTA1 | COL13A1 | NADSYN1 | NUP210 |
| SIX5 | TAF4 | TBCD | TRAPPC9 | CPLX2 |
| FUT7 | OGDHL | PACS2 | PNLDC1 | SPTBN4 |
| AKAP12 | ARVCF | ERGIC1 | LARGE2 | PIGG |
| UGGT2 | CUL9 | DIDO1 | GAS7 | RGS12 |
| SS18L1 | ITM2C | MYO7B | NVL | PPP1R12B |
| UBE2R2 | USP37 | WNK2 | ADAMTSL5 | DIP2C |
| RABGAP1L | SDK1 | TCF25 | MEGF11 | ZFP30 |
| ZNF79 | ZNF607 | EMC8 | GPR160 | MARCHF10 |
| TMEM39A | CCDC86 | CDYL2 | RAB6C | VOPP1 |
| ZC3H3 | ZSWIM2 | TAFA4 | NUDT6 | COMMD5 |
| IGF2R | TPPP3 | ESM1 | ACVR1 | PRPS1 |
| XPO1 | LMNB1 | SPG7 | FYN | OPA1 |
| MIR22HG | DANCR | CSN3 | SLC9A3 | LINC01370 |
| IL12B | NFATC1 | MIR181B2 | PCDHGA3 | MMP25 |
| ACAD8 | MMAA | MSTN | ARHGAP42 | MAPKAPK5 |
| ADAM1A | RF00017-1278 | MIR186 | SUPT16H | PCBP2-OT1 |
| PKHD1 | SWAP70 | SLC35F6 | CIB4 | UBE2L2 |
| ENSG00000225378 | MIR378G-002 | lnc-CIB4-2 | HSALNG0013736 | HSALNG0089737 |
| HSALNG0146421 | PLCB2 | CRABP1 | ARHGEF17 | GPS1 |
| ZXDC | PTTG2 | RNF222 | CCER2 | DEFB133 |
| LINC00316 | MYMY3 | NPY1R | BNIP3 | HSF1 |
| G6PC3 | SLC8A3 | ZFPM2 | MICA | GOSR1 |
| CHRNA2 | ST3GAL5 | HCN1 | CACNA1I | GABRA4 |
| GRM4 | CACNG2 | DLX5 | ME2 | ADAM22 |
| ARX | EMP2 | GALR3 | LRP8 | OPHN1 |
| PHOX2A | SRD5A1 | CSNK1G1 | GALR1 | NDP |
| CLN5 | EFHC1 | MVP | DHRS9 | PCDH10 |
| PPP1R9B | GALR2 | OTX1 | SV2B | KCNMB3 |
| EPM2AIP1 | CSRNP3 | ZACN | CASTOR3P | SCN1A-AS1 |
| TTC21B-AS1 | LOC109610631 | EJM2 | SLX1A-SULT1A3 | LY96 |
| CAVIN2 | AUTS2 | CRLF3 | SIK2 | SLC37A4 |
| LOC100506472 | NEDD4 | MIR151A | P2RX2 | BBS9 |
| GIMAP5 | MIR138-1 | LINC01116 | MIR1275 | LINC00630 |
| MIR339 | ISL1 | MIR4516 | CUBN | TCN1 |
| NBPF3 | PLCG1 | SLC14A2 | SUCNR1 | DELEC1 |
| PLEC | HGFAC | SPPL2A | FARP2 | PSMG1 |
| CHD9 | DIP2B | MS4A6A | CARINH | ZIC3 |
| MPP1 | MHRT | IFNA5 | IFNA17 | PRKACB |
| PRKACG | CALML3 | CALML5 | CALML4 | CALML6 |
| MERTK | NFU1 | MYBPC1 | MIR183 | IL6ST |
| LOC126861898 | TSTD2 | GPBAR1 | CHKB | STK11 |
| PDPK1 | SCT | RAD51 | FANCM | HLTF |
| RECQL | MUS81 | FBH1 | PRIMPOL | ZRANB3 |
| RADX | NXF5 | TBL3 | GPR135 | SRSF2 |
| C4BPA | PRKDC | RBBP4 | RAN | LMNB2 |
| LPAR3 | TWIST2 | MTA3 | RBBP7 | UBA7 |
| H3C1 | NAT10 | H3C3 | H3C6 | H3C10 |
| H3C15 | UBE2I | PLA2G4A | MYLK2 | MYT1L |
| GIMAP4 | C16orf95 | TMEM116 | PCNPP1 | RNU7-159P |
| HSALNG0094037 | HSALNG0094038 | HSALNG0143727 | MN297386 | lnc-FAM109A-1 |
| piR-36455 | piR-38259 | piR-48007 | piR-50346 | piR-51327 |
| piR-51449 | piR-56480-015 | piR-59149 | LOC124903019 | GIP |
| SLC25A11 | ZNF318 | LNCOC1 | DMRTA1 | MIR9-3 |
| CPT1A | SLC25A14 | IFNA6 | IFNA14 | IFNA21 |
| IFNA8 | IFNA10 | IFNA4 | IFNA13 | IFNA7 |
| IFNA16 | ANKRD1 | TAF1A | OPCML | B3GAT3 |
| PAPSS1 | ANKH | ARSJ | KCNJ12 | KCNJ4 |
| CCL20 | KAT2B | ATP2B2 | PEX1 | FUT8 |
| VPS33B | AP1S1 | GALNT17 | ALG12 | TRAPPC11 |
| CEP89 | CEP20 | SLC4A4 | MIR23B | IPW |
| PTGER3 | MIR520G | MIR524 | PDIA2 | INPP5D |
| ALG13 | ATP2B1 | GAST | TET1 | TNNI3K |
| MIR4739 | CREBBP | PICALM | CARS2 | PBK |
| ANO2 | EPG5 | DEFA5 | SCARA5 | CRTAC1 |
| BARHL1 | USP46 | DEFA3 | CCDC83 | DEFA4 |
| SPATA18 | DEFA1B | LINC02932 | OR7E154P | LINC02426 |
| DEFA11P | DEFT1P2 | ENSG00000235450 | MRPS5P4 | RNU4ATAC9P |
| RPL13AP13 | SNORA120 | HNRNPA1P67 | HSALNG0099362 | HSALNG0099364 |
| LOC124903211 | HSALNG0088814 | HSALNG0150129 | piR-52079-196 | HSALNG0063178 |
| lnc-DEFA1B-1-002 | LOC124901874 | LOC124901875 | CDK18 | TRPC3 |
| SLC5A6 | ATF2 | ADAM12 | REL | CYP46A1 |
| ANGPTL8 | GRK5 | LOC111365141 | TCF19 | HLCS |
| ILK | MICB | GLMN | RHOD | PDCD4 |
| CCL15-CCL14 | FLOT1 | MIR135B | MIR708 | LINC01550 |
| LINC00649 | IRX4 | MIR136 | GIPR | CA2 |
| SNORA66 | MS4A1 | U2AF1 | LARP4 | ZRSR2 |
| ABT1 | MIR490 | DMP1 | SLC2A13 | SMAD6 |
| PPP1CA | PDE3B | RAD50 | STX2 | VGLL4 |
| PIEZO2 | DNAAF3 | DNAAF3-AS1 | EIF2AK4 | PTPN6 |
| GPR17 | LBR | TXNIP | SNORD24 | CUL3 |
| MIR1181 | KEAP1 | GJC1 | OFCC1 | DDX6 |
| LRAT | TSHB | TTBK1 | TMEM184C | C1orf127 |
| ZNF474 | NGF-AS1 | ZNF475 | HRG-AS1 | LINC01765 |
| MIR297 | ZCCHC14-DT | LINC02305 | ENSG00000277653 | HSALNG0000800 |
| HSALNG0070392 | MTND4P33 | RF00951-004 | lnc-JPH3-6 | HSALNG0050175 |
| HSALNG0144814 | LOC124903748 | piR-31534-613 | piR-41525-616 | piR-58308-245 |
| HSALNG0006259 | HSALNG0006261 | RELB | DYRK1A | MIR34B |
| CASC15 | LINC-PINT | MIR518B | MIR767 | MIR1305 |
| SCAI | PSMD9 | MIR7-3HG | SLC29A3 | HBEGF |
| PFKM | MACROD2 | REST | UTS2R | KLF15 |
| DUSP19 | ABCD4 | MCEMP1 | UQCRFS1 | PAH |
| GNB1 | ATP2B1-AS1 | NR2F1 | PTH1R | ADCY1 |
| RPL7P45 | TNKS2 | PANK2 | CTNNA3 | PLCL2 |
| GNRH1 | JUND | MIR205 | LINC00115 | MIR651 |
| LOC110596866 | LOC110599585 | CASQ2 | CHKB-CPT1B | INPPL1 |
| NOL3 | AFP | RPL18P1 | PEA15 | DHODH |
| ADH4 | GYS2 | PLOD3 | IL1RL2 | SFRP4 |
| HTR3B | CXCR5 | DDX39B | PEX10 | CLEC4M |
| SRSF6 | TTC8 | ASIP | STK19 | RBM12 |
| PSORS1C1 | MEIKIN | HCG26 | CD200 | CYP27A1 |
| RETREG1 | MIR3944 | TEAD1 | MIR92A1 | ADD3 |
| POMK | PPP3CA | SLK | FAM20C | PAPSS2 |
| PHEX | SLC34A3 | SLC2A6 | PHOSPHO1 | SLC2A11 |
| SLC2A14 | GNA11 | CCL18 | EGLN2 | MBD4 |
| PDE9A | BRD1 | OR2AG1 | GREM1 | CBR1 |
| KCNJ8 | SLC25A4 | MMADHC | LMBRD1 | TSC22D3 |
| NR1H2 | APELA | DRD4 | ADD2 | GRK4 |
| DNM2 | LAMC1 | CXCL14 | NT5C1B | IFIT2 |
| HIF1A-AS1 | WNT3A | U2AF2 | NALT1 | CYSLTR1 |
| EPX | MTF1 | TAC3 | PEE1 | EVA1A |
| CXCL11 | KCNN3 | PNPO | FIS1 | TRDMT1 |
| HTR1E | AAR2 | QARS1 | PEPD | DDR2 |
| TPM4 | RP1 | TMEM132D | MYH7B | FLRT2 |
| MTRFR | RPL15P15 | TERF1P3 | NR1H4 | HYAL1 |
| BLM | HAAO | CEP164 | RNF8 | RXFP2 |
| CEP250 | LPCAT2 | EML4 | IL36G | PI16 |
| STK38 | ETV7 | IL36B | KCNG3 | RBM39 |
| RILP | ARMC12 | BRPF3 | CMTR1 | SRRM4 |
| TMEM14C | DMRTA2 | RAP1GAP2 | FAM133B | ADTRP |
| C6orf89 | TMEM170B | RAB44 | GTSCR1 | C1orf185 |
| TRA | POC1B-GALNT4 | CDK6-AS1 | TBX3-AS1 | DISC1FP1 |
| LINC01541 | LINC02955 | LINC02511 | VPS33B-DT | LINC01148 |
| LINC02884 | ENSG00000258302 | LINC02309 | RNU1-88P | RNU6-1180P |
| ENSG00000266993 | ENSG00000271897 | RNU6-229P | RPL7AP30 | UBA52P7 |
| ICE2P2 | LOC107985936 | RN7SL363P | ENSG00000237719 | ENSG00000287593 |
| BOLA3P1 | CM034959-043 | ENSG00000286742 | MTCO1P25 | RN7SL292P |
| lnc-ALDH1A2-9 | ENSG00000290102 | GAPDHP50 | HSALNG0049754 | LOC124904176 |
| MN298114-175 | NONHSAG020469.2 | OA985837 | RF00017-5320 | RF00017-5535 |
| RF00017-5538 | RF00017-5541 | SGO1P2 | lnc-CDKN2C-1 | lnc-FAF1-3 |
| lnc-FURIN-3 | lnc-MTAP-3 | lnc-RAB44-3 | lnc-TMEM170B-5 | CM034956-128 |
| ENSG00000243831 | HSALNG0048081 | HSALNG0092956 | HSALNG0106222 | HSALNG0108163 |
| HSALNG0108164 | RF00017-6504 | RF00026-552 | lnc-AQP9-4 | lnc-COX7A2L-1 |
| lnc-CTTNBP2NL-4 | lnc-EML4-10 | lnc-FAM133B-2 | piR-32285-085 | piR-59515-001 |
| HSALNG0123985 | lnc-LPA-4 | lnc-RILP-2 | piR-51980 | piR-58579 |
| BLZF1 | MIR377 | PPP2R2B | CSPG4 | KRT8 |
| LINC01094 | HSD17B10 | KIAA0586 | ATXN3 | SARS2 |
| HYT1 | ITGA1 | HRH1 | GNAI1 | HSPA14 |
| ADRA1B | UBE2G2 | KCNK9 | KCNK5 | ERVFRD-1 |
| TRPA1 | LYZ | TBX1 | CRELD1 | CFB |
| SNX10 | MTRNR2L8 | EIF4A2 | BTD | MED23 |
| HGS | ATP8B4 | CACNA2D3 | PLD2 | MEG8 |
| UBQLN2 | MIR511 | MIR1-1 | HTR1B | THBS2 |
| IL32 | NMT1 | MELTF | ADAMTSL2 | ARTN |
| LRRFIP1 | CYP27B1 | SP3 | KIR2DL1 | CD226 |
| RNF43 | ADM2 | MICAL2 | C5AR1 | MYOCD |
| MYDGF | COQ7 | BBS2 | TMEM67 | DYNLT1 |
| LOC106799834 | PRDX2 | FAAH | BRD4 | FZD7 |
| ADGRL3 | TRA2B | COL14A1 | MIR1248 | PTPN3 |
| RORA | CDC7 | ARNT2 | NDUFV3 | NPLOC4 |
| SEPTIN2 | PF4V1 | CHORDC1 | IRX1 | PPP2R5E |
| DCHS2 | NAALAD2 | KAZN | AJAP1 | STARD3NL |
| TMX1 | FBXO33 | TC2N | TSC22D4 | TP53TG1 |
| MIR4492 | RPS19P6 | ABCB4 | DCDC2 | MIR486-2 |
| CASR | APRT | FLVCR1 | SIGLEC8 | ADIPOR1 |
| MEF2C | HLX | FAM107B | OXTR | CPQ |
| MT2A | ADGRL4 | SPTAN1 | IL18BP | MIR433 |
| MESP2 | KCNIP2 | SUMF1 | TLR6 | AIP |
| BCKDHB | ABCA7 | ANXA11 | TYROBP | SNCG |
| PCSK1N | DRD3 | SEC61A1 | TRIM32 | SDCCAG8 |
| IFT172 | BBS7 | BOLA3 | IBA57 | MEX3C |
| HYT3 | HYT4 | HYT8 | LOC117600004 | RFH1 |
| ABCB8 | MIR33B | ING5 | DPYD | PLXND1 |
| RPL34-DT | MIR328 | CD44 | TNFRSF25 | CAPN5 |
| ERV3-1 | FOXC2 | CYSLTR2 | HCRTR2 | ID1 |
| S1PR2 | TRPM4 | MIR1297 | AFF2 | KCP |
| TNXA | LOC114827851 | KCNK18 | STMN2 | TPK1 |
| GOT1 | LPIN1 | SHANK2 | PARVA | P2RX5-TAX1BP3 |
| GSTA4 | MSBP1 | KDM4C | MYOM2 | NOS1AP |
| CHRM2 | RNF214 | NTRK3 | CDK2 | STIM2 |
| PHOX2B | SLC7A7 | MIR103A2 | EZR | TPH1 |
| ACTN4 | E2F3 | TRPM8 | ADORA2B | NAIP |
| HTATIP2 | TCF4 | ATG5 | FFAR3 | SEMA7A |
| KMT2A | WEE1 | ADH5 | PLCB4 | CELF2 |
| EIF4A3 | EPHB1 | FXR1 | FZD1 | HINT1 |
| EED | GDA | GRIP1 | NDUFS6 | RFC4 |
| SEMA3C | VPS11 | CNTN6 | CYTH1 | DHX38 |
| DNAJC19 | ETS2 | HMGN1 | IFT122 | KIRREL3 |
| LNX1 | PTPRT | AFF4 | NCOA6 | PSMB3 |
| RAB8A | STAG1 | TNFRSF21 | ACR | ATXN2L |
| CPD | DCPS | DNAJB11 | KTN1 | NLRP5 |
| RGS10 | SLC14A1 | SLC36A2 | SORBS2 | SYNGR1 |
| TRAPPC4 | TUBGCP6 | AMBN | ARCN1 | ENAM |
| FCRL5 | HNRNPA3 | MGAT3 | PLEKHA1 | SBF2 |
| UAP1 | USP28 | CADPS2 | CCHCR1 | COPS8 |
| CTR9 | CXCL6 | EIF2S2 | EVI5 | FOXP4 |
| GALNT1 | KCTD13 | NCAPH2 | POP1 | PPP6R2 |
| PRCC | RHPN2 | SDR16C5 | TMED1 | UPK2 |
| WDR4 | ZFP36L1 | ACER3 | ADAMTSL3 | AKAP8 |
| DYNC2I1 | FCHO2 | FEZ1 | FNIP1 | FRMD6 |
| GDI2 | HOXD8 | IFT52 | KCNS3 | L3MBTL1 |
| MPHOSPH9 | NCAM2 | PCOLCE | PHLDB1 | PIK3C2G |
| PLCH2 | PLRG1 | STAB2 | ANO7 | ARHGAP21 |
| CDK2AP1 | CEP131 | FAAP24 | FBXO38 | GALNT13 |
| GIGYF1 | OSBPL1A | PIGU | PLXNB2 | RPS25 |
| TMOD4 | ZNHIT6 | BRMS1L | FAAP100 | FITM2 |
| KBTBD8 | LINGO2 | NRIP3 | PELI2 | PGLYRP3 |
| RER1 | SHKBP1 | SIGLEC15 | SNX13 | TBCCD1 |
| TTC17 | ZNHIT1 | ADGRF1 | AGMO | ASPHD1 |
| CCL16 | GALNT4 | GTF3C4 | HINFP | HOXD1 |
| IRX2 | LMF2 | PRDM15 | RNF17 | TRIM56 |
| TSKU | CENPQ | DCAF5 | DPY19L3 | ELAPOR2 |
| FGD6 | FOXN3 | INO80E | LRCH4 | NIPAL2 |
| PDZRN4 | PPP2R3A | PRTFDC1 | R3HCC1L | RAPGEF6 |
| RASAL3 | RPAP2 | SPINK7 | ZBED4 | ARRDC4 |
| B3GNT4 | CCDC59 | DTWD1 | EML5 | MORN1 |
| NRBF2 | PCNX1 | PSORS1C2 | RASEF | REEP3 |
| RPUSD4 | WIZ | ZNF507 | ZNF516 | C2CD2L |
| EQTN | FAM118B | FOXR1 | GLTP | LCA5L |
| LRRC43 | MEPCE | PDCD11 | PILRB | PPP1R35 |
| TMEM74 | ZNF333 | ZNF431 | ZNF77 | ANKRD18A |
| BCL9L | CENATAC | LY6G6C | METTL17 | NYNRIN |
| PRMT9 | RNF26 | THNSL1 | TMEM123 | TMEM171 |
| TTPAL | USP38 | VAT1L | C7orf50 | DENND6B |
| GPATCH1 | STPG2 | TBPL2 | TEPSIN | TMEM51 |
| ZFAND2A | CIMAP2 | FAAP20 | H1-10 | LRRC31 |
| RERGL | VWDE | WDR88 | ZCCHC10 | EFCAB12 |
| LYZL6 | METTL25B | OR2B11 | ZNF691 | OR52B6 |
| SPINK9 | SYCE3 | TMEM14A | TMEM243 | CFAP77 |
| DRC12 | MCCD1 | PLSCR5 | SYCE1L | NAT16 |
| ZNF626 | RPRML | SPDYE3 | TRIQK | LINC03042 |
| ZNF737 | MS4A4E | CLDN25 | ANP32CP | MAPKAPK5-AS1 |
| PSORS1C3 | HAGLR | NPIPA5 | CYP1B1-AS1 | SBF2-AS1 |
| CCDC179 | LINC00474 | SNORA4 | MCPH1-AS1 | MIR3936HG |
| OR4E1 | SNORA63 | SUZ12P1 | KTN1-AS1 | LINC00640 |
| LINC02906 | PPP1R3B-DT | SNORD117 | SNORD2 | COX7A2P2 |
| PKD1P1 | SNORA81 | LINC01620 | OSER1-DT | PCOLCE-AS1 |
| PRKCZ-AS1 | TIRAP-AS1 | CALCRL-AS1 | LINC00343 | LINC00923 |
| LINC02577 | LOC100131635 | RPL32P3 | TMEM9B-AS1 | B4GALT4-AS1 |
| HNF4A-AS1 | LINC00924 | LINC01376 | LINC01750 | LINC02240 |
| LINC02709 | MOCS2-DT | RPL21P44 | SDR16C6P | SNORA63B |
| LINC01422 | LINC01522 | LINC02882 | LOC100507053 | NALCN-AS1 |
| TRAV2 | ADM-DT | CELF2-DT | CENATAC-DT | CHKB-DT |
| DEPDC1-AS1 | FBXO38-DT | IL1R1-AS1 | LINC01430 | LINC01854 |
| LINC02267 | LINC02566 | LINC02940 | LINC02997 | LINC03062 |
| MIR3646 | MIR4277 | MIR8084 | RPL23AP64 | TRAM2-AS1 |
| TSKU-AS1 | UNC5C-AS1 | ZFAND2A-DT | LINC01377 | LINC01707 |
| LINC01893 | LINC02010 | LINC02315 | LINC02349 | LINC02399 |
| LINC02501 | LINC02863 | LINC02885 | LINC03018 | MICB-DT |
| MIR4290HG | MIR4497 | NPIPA6 | NXT1-AS1 | SBNO1-AS1 |
| SIPA1L1-AS1 | EIF3IP1 | ENSG00000222022 | ENSG00000261707 | ENSG00000262633 |
| LINC02151 | LINC02400 | LINC02497 | LINC02718 | LINC02772 |
| LOC101928855 | LOC101928882 | MIR1470 | PDCD6P1 | PPIAP9 |
| SELENOO-AS1 | STK19B | ATF4P4 | DBF4P1 | ENSG00000224079 |
| ENSG00000227698 | ENSG00000231724 | ENSG00000233754 | ENSG00000254428 | ENSG00000254632 |
| ENSG00000258216 | ENSG00000258942 | ENSG00000261578 | ENSG00000263826 | ENSG00000271716 |
| LINC01643 | LOC124902827 | LOC157273 | MRPL35P2 | RNU2-3P |
| RNU6-1066P | RNU6-380P | RPL15P4 | RPL21P97 | RPL5P30 |
| RPL7AP10 | RPS20P14 | STAG3L5P | ENSG00000145075 | ENSG00000202533 |
| ENSG00000231236 | ENSG00000231421 | ENSG00000242798 | ENSG00000243107 | ENSG00000250421 |
| ENSG00000251330 | ENSG00000253496 | ENSG00000253821 | ENSG00000254397 | ENSG00000254694 |
| ENSG00000255317 | ENSG00000256250 | ENSG00000259560 | ENSG00000261063 | ENSG00000262049 |
| ENSG00000262879 | ENSG00000283782 | PRDX1P1 | RN7SL688P | RNA5SP478 |
| RNF13P1 | RNU6-440P | RPL10P1 | RPL17P35 | RPL23AP12 |
| RPL30P10 | RPL30P6 | RPSAP64 | VN2R10P | ARL2BPP4 |
| ENSG00000222529 | ENSG00000235023 | ENSG00000245869 | ENSG00000249678 | ENSG00000272836 |
| ENSG00000273218 | ENSG00000284977 | ENSG00000286508 | ENSG00000288545 | ISCA1P6 |
| LCN1P2 | MAGOH3P | MKRN8P | NRBF2P1 | ORMDL1P1 |
| RNA5SP107 | RNU6-284P | RNU6-560P | RNU7-165P | RPL21P41 |
| RPL31P5 | RPP40P2 | RPS20P25 | RPS2P1 | RPSAP37 |
| TPT1P9 | TRPC6P3 | ZBED1P1 | BRWD1P2 | ENSG00000249894 |
| ENSG00000258751 | ENSG00000269110 | ENSG00000289728 | ENSG00000290395 | KRT18P43 |
| MPPE1P1 | MTCYBP43 | NCSTNP1 | POLR3KP1 | RN7SKP242 |
| RN7SL474P | RNA5SP430 | RNA5SP63 | RNU4-64P | RNU6-1060P |
| RNU6-297P | ANKRD11P1 | COX6A1P4 | ENSG00000224431 | ENSG00000254909 |
| ENSG00000267611 | ENSG00000269779 | ENSG00000284946 | ENSG00000288587 | ENSG00000289764 |
| HNRNPA1P53 | ILF2P2 | LOC101928255 | LOC124901794 | MRM3P2 |
| MTND1P24 | MTND4LP18 | RNA5SP360 | RNU6-694P | RNU7-92P |
| SDHBP1 | SPTLC1P2 | TRMT112P7 | USP32P4 | hsa-miR-5096-153 |
| lnc-EED-4 | lnc-IRF1-1 | lnc-RPS25-6 | lnc-SBF2-3 | lnc-SETSIP-3 |
| lnc-VIPR2-1 | BUB3P1 | CHMP4AP1 | CM034953-349 | CM034955-349 |
| CM034961-053 | CM034961-054 | CM034964-116 | ENSG00000233902 | ENSG00000256350 |
| ENSG00000269896 | ENSG00000285637 | ENSG00000289328 | HSALNG0020226-023 | HSALNG0020226-431 |
| HSALNG0020226-444 | HSALNG0020226-445 | HSALNG0020226-483 | HSALNG0049245-002 | HSALNG0080729 |
| HSALNG0087578 | HSALNG0088030 | HSALNG0094715 | HSALNG0125404 | HSALNG0143222 |
| HSALNG0149400 | HSALNG0149699 | LARP1BP1 | LOC107984869 | LOC124902802 |
| LOC124904075 | LOC124905050 | MK280269-006 | MN298114-173 | NIPAL2-AS1 |
| NONHSAG036923.2 | NONHSAG046618.2 | NONHSAG048398.2 | RF00004-004 | RF00017-2598 |
| RF00017-6567 | RF00017-6571 | RF00017-6572 | lnc-ACTN1-2 | lnc-C7orf50-1 |
| lnc-CHORDC1-1 | lnc-CXCR5-2 | lnc-EGR2-6 | lnc-FITM2-5 | lnc-HDAC9-10 |
| lnc-IL1F10-2 | lnc-MOCS2-5 | lnc-MS4A6A-1 | lnc-NEURL1-4 | lnc-POP1-1 |
| lnc-RAB17-7 | lnc-RSPH1-4 | lnc-SWAP70-4 | lnc-TAMM41-4 | lnc-TIMM29-1 |
| lnc-TMEM105-1 | lnc-TRIM49D1-2 | lnc-USP28-3 | lnc-VGF-4 | lnc-ZNF66-3 |
| piR-43325-005 | piR-44098 | piR-48209-507 | piR-57460-243 | AB372791 |
| CM021582-026 | CM034953-362 | CM034954-114 | CM034955-350 | CM034964-105 |
| CM034967-342 | FJ601684-376 | HSALNG0004020 | HSALNG0012260 | HSALNG0035985 |
| HSALNG0039707 | HSALNG0044846 | HSALNG0055618 | HSALNG0059486 | HSALNG0059492 |
| HSALNG0074936 | HSALNG0076746-001 | HSALNG0076749 | HSALNG0080735 | HSALNG0087588 |
| HSALNG0094630 | HSALNG0099558 | HSALNG0099559 | HSALNG0102123-001 | HSALNG0102123-002 |
| HSALNG0102125 | HSALNG0108471 | HSALNG0112528 | HSALNG0112598 | HSALNG0119064 |
| HSALNG0121262-004 | HSALNG0124827 | HSALNG0129694 | HSALNG0129696 | HSALNG0129805 |
| HSALNG0130581 | HSALNG0132447-002 | HSALNG0133099 | HSALNG0133110 | HSALNG0133334 |
| HSALNG0135506 | HSALNG0136319 | HSALNG0145259 | HSALNG0146572 | HSALNG0147184 |
| HSALNG0148361 | HSALNG0148535 | HSALNG0149861 | LOC105372762 | LOC105378490 |
| LOC124902821 | LOC124904659 | MK280144-536 | MN298214 | NONHSAG020037.2 |
| RF00017-1051 | RF00017-6336 | RF00026-195 | RF00066-040 | RF00994-505 |
| SNODB1980 | hsa-miR-5095-132 | lnc-ACER3-1 | lnc-ADAMTS18-12 | lnc-ADAMTS5-5 |
| lnc-ATP6V1G2-DDX39B-3 | lnc-BNC1-16 | lnc-CCKAR-20 | lnc-CDK2AP1-1 | lnc-CXCL6-3 |
| lnc-DDR2-3 | lnc-DDX31-2 | lnc-EGR2-5 | lnc-FBXO34-1 | lnc-FBXO38-1 |
| lnc-FST-6 | lnc-NCOA6-4 | lnc-NRBF2-2 | lnc-PASK-4 | lnc-PRC1-3 |
| lnc-R3HDML-1 | lnc-SGCB-1 | lnc-SPATA18-4 | lnc-SRPRA-7 | lnc-TTC14-3 |
| piR-30695 | piR-31432-181 | piR-31541 | piR-33614-122 | piR-34154 |
| piR-37824 | piR-38580-046 | piR-38829 | piR-43106-156 | piR-43222 |
| piR-48325-111 | piR-49732-058 | piR-50444-564 | piR-56320 | piR-57337-010 |
| piR-58712 | piR-60051-065 | HE855921 | HSALNG0039132 | HSALNG0071141-002 |
| HSALNG0074935 | HSALNG0076128 | HSALNG0078466-517 | HSALNG0090516 | HSALNG0094629 |
| HSALNG0094717 | HSALNG0107907 | HSALNG0119063 | HSALNG0123961 | HSALNG0124379 |
| HSALNG0132452 | HSALNG0146769 | HSALNG0148469 | HSALNG0148994 | HSALNG0150742 |
| HSALNG0150813 | LOC105370935 | LOC105374240 | LOC105375855 | LOC107986462 |
| LOC124902956 | LOC124903726 | LOC124904805 | MN309174-262 | NONHSAG024805.2 |
| RF00026-1064 | RF00998-050 | lnc-LEPR-1 | lnc-NR3C2-8 | lnc-PLCB2-1-001 |
| lnc-PLCB2-1-003 | lnc-SIGLEC15-1 | lnc-SLC1A1-7 | lnc-TERF2IP-6 | piR-30791-025 |
| piR-31199-235 | piR-32023-046 | piR-32214-308 | piR-33101-001 | piR-33103-021 |
| piR-33945 | piR-34822-091 | piR-35581 | piR-36756-007 | piR-38562-028 |
| piR-42806-013 | piR-55985-066 | piR-56120 | HSALNG0020602 | HSALNG0110658 |
| HSALNG0147056 | lnc-SEZ6L2-1 | piR-31432-165 | piR-38351-348 | piR-39341-170 |
| piR-43099-154 | piR-43583-142 | piR-47086-217 | piR-53431-166 | piR-54764-163 |
| piR-55650-161 | piR-58156-028 | piR-61240-147 | SEPT5-GP1BB | MOCS1 |
| TRPC1 | ARG2 | PTK6 | TBX2 | SLC35A3 |
| TRIM8 | ATF6B | BRK1 | IL4I1 | HES7 |
| LAX1 | PCDH18 | TMEM11 | SYNC | GBP4 |
| SPIN4 | SPIN3 | MIR6082 | ATP1A1 | ASIC2 |
| NINJ1 | ACADSB | HIBCH | CA1 | FOXO4 |
| HAS2 | MIR369 | CALHM1 | PTGER1 | CAMK2A |
| GPR35 | KIRREL1 | CAMK2D | CHRM1 | GLRX |
| CXCL9 | LIPN | LMAN1 | PLK4 | HLA-S |
| MIR96 | MAP3K7 | ZDHHC17 | MAP2K3 | TRL-TAG1-1 |
| CIB1 | SLC1A7 | MIR373 | LMO4 | CEBPA |
| TRD-GTC9-1 | MYOZ2 | BAG6 | TMEM107 | SUGCT |
| PIGM | THORLNC | TRN-GTT2-1 | WWP1 | SEC11A |
| YAP1 | SMPD2 | SUCLA2 | SUCLG1 | ACSF3 |
| BCKDHA | TMLHE | ELOC | ELOB | LDHA |
| ALOX15B | MIR1260B | FXN | FOXM1 | OLIG2 |
| STEAP4 | MIR939 | ATXN1 | OPTN | DNAJC5 |
| SERPINA10 | SLC2A9 | CHEK2 | TCFL5 | TKT |
| CLCN7 | TAT | MIR302D | MIR663B | MIR196B |
| CSK | OGN | SLC39A2 | AKT1S1 | IFRD1 |
| TRIM45 | SNORD73A | MIR1291 | ATP2B4 | PTAFR |
| CDR1-AS | NOTCH2 | LINC00336 | ZEB1-AS1 | ARF1 |
| ERBB2 | SLC11A2 | NRF1 | FAH | AVPR2 |
| DLK1 | SLC1A6 | TIAL1 | EXT1 | SESN2 |
| FGF10 | SRA1 | SENP3 | CLOCK | IL23A |
| BSCL2 | INO80 | AOC1 | TGFBI | RGS4 |
| MIR454 | FKBP1A | SLC29A1 | POSTN | CAAP1 |
| RNF32-DT | SNORD50A | PRDX1 | HTR7 | KCNK4 |
| TRPV2 | LOC130002910 | CALCR | VIPR1 | HSPE1 |
| ZBTB7C | MS | EGLN3 | DLGAP1-AS2 | LINC02249 |
| KIF12 | SLC6A9 | NISCH | NRAS | PSMB8 |
| SMAD5 | DKC1 | SCARB2 | HTR1D | ARRB1 |
| COL4A6 | MIR28 | STC2 | GPHA2 | SNAI2 |
| MIR429 | CTH | HOXA11-AS | MIR302C | MIR92A2 |
| MIR1197 | AQP3 | CYLD | APBB1 | ATN1 |
| TIA1 | CCNF | CHCHD10 | SPG21 | MFSD8 |
| MOBP | DIPK1C | EPCAM | NEGR1 | PPP1R15A |
| LCK | IGFBP5 | TERF2 | CIDEA | LAMB2 |
| CDH1 | TRO | DSC2 | YY1 | DAP3 |
| HLA-A | MTM1 | EIF4E | PLEKHG1 | PRKAB1 |
| RAP1A | POLR1F | TMEM196 | TERF1 | LEF1-AS1 |
| MSI1 | CTSL | PER2 | CSNK2A1 | GPR68 |
| FUNDC1 | LINC01619 | MIR4647 | IMPA1 | PARP2 |
| PARP4 | PARP3 | SLC9A2 | GBA3 | POT1 |
| TEP1 | ZBTB46 | PPP3R1 | KIR2DL3 | KIR2DL2 |
| NPC1L1 | FCGR1A | PTPA | SDC4 | CSNK1D |
| CACNA1E | DRD5 | TRPV3 | HTR1F | HTR5A |
| RAMP3 | AGAP1 | KCNK10 | CALCB | LAPTM4A |
| ZBTB7A | KRT76 | ANKDD1B | MTRNR2L5 | MGR6 |
| MGR12 | MGR2 | MGR3 | MGR5 | MGR8 |
| MGR10 | MGR11 | LDLRAP1 | ESRRA | CHRNA5 |
| SCFD1 | NAB2 | S100A12 | DIABLO | PTK2B |
| PHF6 | DROSHA | PMS1 | AIRE | DICER1 |
| PACC1 | IL7 | MIR378A | CARD16 | CD320 |
| GGACT | LOC124946329 | APOA1-AS | OXA1L | SPARC |
| PTPRJ | MSX2 | AGO2 | PCSK6 | CCT3 |
| MAP4K2 | PAPPA2 | PIGF | TPI1 | MCAM |
| TRAF2 | MRPL44 | RLN2 | DKK2 | PPP2CA |
| GYG1 | ZNF98 | SLC5A1 | BIRC3 | YWHAZ |
| TRIM28 | AKAP13 | CISD2 | PDLIM1 | FKBP4 |
| SLC4A7 | MYOG | RXRA | HIF1AN | PKLR |
| GSS | ATP2A3 | PPP3CB | MIR3679 | PNPLA3 |
| LGALS13 | ADAMTS5 | UCN2 | SNORD47 | ITGA8 |
| ALDH9A1 | UBA1 | FDFT1 | TRV-AAC1-4 | LOC106050102 |
| SERPINH1 | APEH | CNMD | LIM2-AS1 | MAP2K1 |
| MIR200A | CYP2C18 | PPARGC1B | SGCB | SENP2 |
| HIF1A-AS2 | NPY5R | CD58 | VLDLR | CLEC4A |
| PTPN2 | CGB3 | CASC3 | HRH2 | MIR101-1 |
| SNORD43 | LRP2 | CTSG | STAT6 | GRK3 |
| SPI1 | TEAD4 | PLSCR3 | USP34 | TATDN1 |
| EMILIN3 | MRPL52 | LINC00472 | LINC01234 | PAK1 |
| VNN1 | HOTAIRM1 | CA3 | LCT | FUT3 |
| MEPE | APOBEC2 | CLCN3 | HCRTR1 | MFN1 |
| FLT4 | FAM167A | CNTN1 | GLO1 | GNRHR |
| PGM1 | KRT19 | CFL2 | PDIA3 | ST6GAL1 |
| GNE | PPIC | ACADL | MAZ | SHOX |
| PEG3 | PWAR6 | SCNN1D | PLEK | SCG5 |
| RAB3GAP2 | GPRC6A | GUCY2C | OLIG1 | IFNE |
| MIR194-2 | AARS1 | PTPRU | CRLS1 | IL3RA |
| RPS6KB1 | DCTN1 | CHRNE | MAP2K5 | PRKRA |
| MIR409 | MIR517A | HSP90AB1 | PRLR | CR1 |
| IL12RB2 | SLC22A8 | TIMM8A | ACOX3 | HLA-DRB4 |
| CXCL5 | FRMD4B | GATD3 | MIR217 | TTTY15 |
| PICART1 | JUP | ACKR2 | MYOD1 | ALMS1 |
| LUM | FCN3 | CMYA5 | NBR2 | RN7SL1 |
| BANCR | GJD2-DT | F11R | ITGA6 | NPR2 |
| MIR1-2 | PRG4 | EFNB1 | APOC4-APOC2 | ITGA7 |
| VTRNA1-1 | MAP3K14 | AKT3 | DUSP1 | GJB2 |
| AOX1 | RORC | RNY1 | TNFRSF10B | ITGB4 |
| MMP16 | SLC8A2 | RGS2 | PEBP1 | CCAT1 |
| MIR194-1 | ITPKB | GPC3 | WWP2 | PLPP1 |
| MDFI | FLJ16779 | EIF4G1 | ITGB5 | RARA |
| LAMB1 | BMP1 | CCND2 | PBX1 | NFATC2 |
| NCF2 | CCR7 | CEBPB | COL6A1 | NCL |
| NCOA1 | SLC22A6 | SNAI1 | MCFD2 | PHYH |
| CRHR2 | NPY2R | ACAA2 | BPI | AEBP1 |
| MKKS | PPM1K | SYN2 | ADAMTS9 | DCAF17 |
| ILF2 | TAAR1 | RO60 | DGCR5 | MIR129-1 |
| MIR3609 | HMOX2 | PPM1A | EGFL7 | TRIM65 |
| TRIM47 | SIRT4 | HMCN1 | FTH1 | ANXA6 |
| SPTBN2 | GDF3 | CKMT1A | SKAP1-AS2 | CASP6 |
| ITGA9 | ITGB7 | ITGA11 | ITGAD | ITGB8 |
| ITGA10 | MIR20B | MIR4508 | PPP2CB | MIR203A |
| GATA2 | DBNL | SUMO2 | C11orf65 | MIR141 |
| CSF2RA | ANKRD2 | SP8 | HTT-AS | RPPH1 |
| HTR2A-AS1 | TMCC2-AS1 | ENSG00000254664 | ENSG00000232828 | ENSG00000255143 |
| ENSG00000272631 | LONP1 | MARS1 | SLC1A4 | SLC1A5 |
| DHX9 | SGCA | VAV2 | DAB2IP | PAFAH2 |
| CEBPA-DT | PCBP1-AS1 | MIR506 | MIR624 | LOC117125594 |
| MAP3K1 | MAP2K7 | FAP | HELLS | S1PR3 |
| FRZB | FCER1G | SLC24A1 | EIF4G2 | AQP8 |
| CREG1 | MIR744 | MIR653 | MIR450B | MIR3149 |
| FHL1 | ESRRG | TJP2 | MEIS1 | CD93 |
| MIR484 | MIR3146 | CHEK1 | EIF2S1 | LINGO1 |
| CUL4A | MAVS | DIP2A | NOX5 | EFS |
| GSTA2 | SIVA1 | MIR376C | JPX | MIR539 |
| SMILR | TRP-AGG2-6 | MIR3171 | MIR892A | ADAM10 |
| HSPA9 | AHR | CCNA2 | POLR1C | PPP1R12A |
| DBT | AZIN2 | PARG | SLC16A4 | H2BC3 |
| NR2F1-AS1 | MIR147B | LINC00539 | MIR3189 | MIR1279 |
| MIR1264 | TRG-GCC3-1 | PGR | PRMT1 | VRK1 |
| CANX | FLRT3 | PLCD1 | PRKCI | COL6A2 |
| CSF2RB | E2F4 | SIRPA | RHOT1 | SLC26A2 |
| CYP2U1 | DYNLL1 | SLC6A6 | LGALS3BP | BBC3 |
| HACE1 | RAB1A | CCT4 | HAT1 | KLF10 |
| MUC2 | OXSR1 | SLC38A1 | SLCO2B1 | MLLT1 |
| CMKLR1 | DLGAP4 | ESAM | BIK | FHOD1 |
| HIGD1A | LY86 | APOO | CEMIP2 | NRDC |
| BCL2L14 | DCTN6 | LANCL1 | OSR1 | SLC38A5 |
| WBP1L | WT1-AS | MIR26A1 | DNM3OS | HCG11 |
| HOXA-AS2 | MIR95 | LINC00963 | MIR627 | CASC9 |
| CCAT2 | MIR942 | ABALON | MIR216B | HOXB-AS2 |
| MIR4284 | MIR3158-2 | MIR448 | MIR1202 | MIR3148 |
| TRK-TTT3-5 | STRK1 | THPH2 | RPRGL1 | THPH1 |
| FLAP | BSS | DCP1 | RPRGL2 | BDPLT1 |
| ACE1 | VWDP | MVCD3 | BDPLT3 | ICH |
