# Supplementary material for: Elucidating the mechanism of Buyang Huanwu Decoction in the treatment of ischemic stroke: A network pharmacology and molecular docking study
Source: Medicine (Baltimore). 2026 Jul 17;105(29):e49736. doi: 10.1097/MD.0000000000049736 (PMC13384647; doi:10.1097/MD.0000000000049736)
Supplement: Supplementary file 3 [file medi-105-e49736-s003.docx]

**S 3.** Functional Proteins Associated with the 190 Target Genes.

|  | **Gene name** | **Protein name** |  | **Gene name** | **Protein name** |
| --- | --- | --- | --- | --- | --- |
| 1 | MMP2 | 72 kDa type IV collagenase | 96 | CYCS | Cytochrome c |
| 2 | KCNMA1 | Calcium-activated potassium channel subunit alpha 1 | 97 | PCOLCE | Procollagen C-endopeptidase enhancer 1 |
| 3 | XDH | Xanthine dehydrogenase/oxidase | 98 | BIRC5 | Baculoviral IAP repeat-containing protein 5 |
| 4 | NOS2 | Nitric oxide synthase, inducible | 99 | PPP3CA | Serine/threonine-protein phosphatase 2B catalytic subunit alpha isoform |
| 5 | MET | Hepatocyte growth factor receptor | 100 | FABP5 | Fatty acid-binding protein, epidermal |
| 6 | PRKCB | Protein kinase C beta type | 101 | ADRA2A | Alpha-2A adrenergic receptor |
| 7 | GSTA2 | Glutathione S-transferase A2 | 102 | PTGES | Prostaglandin E synthase |
| 8 | MAOA | Amine oxidase [flavin-containing] A | 103 | CRP | C-reactive protein |
| 9 | BCL2 | Apoptosis regulator Bcl-2 | 104 | CHRNA2 | Neuronal acetylcholine receptor subunit alpha-2 |
| 10 | F7 | Coagulation factor VII | 105 | ADRB1 | Beta-1 adrenergic receptor |
| 11 | CYP1A1 | Cytochrome P450 1A1 | 106 | TNF | Tumor necrosis factor |
| 12 | CHRM1 | Muscarinic acetylcholine receptor M1 | 107 | IL1A | Interleukin-1 alpha |
| 13 | AHR | Aryl hydrocarbon receptor | 108 | SPP1 | Osteopontin |
| 14 | NFE2L2 | Nuclear factor erythroid 2-related factor 2 | 109 | F2 | Prothrombin |
| 15 | XIAP | Baculoviral IAP repeat-containing protein 4 | 110 | PTGS2 | Prostaglandin G/H synthase 2 |
| 16 | OLR1 | Oxidized low-density lipoprotein receptor 1 | 111 | CTNNB1 | Catenin beta-1 |
| 17 | NCF1 | Neutrophil cytosol factor 1 | 112 | HAS2 | Hyaluronan synthase 2 |
| 18 | MYC | Myc proto-oncogene protein | 113 | CCND1 | G1/S-specific cyclin-D1 |
| 19 | CCNA2 | Cyclin-A2 | 114 | ESR1 | Estrogen receptor |
| 20 | MT-ND6 | NADH-ubiquinone oxidoreductase chain 6 | 115 | PDE3A | cGMP-inhibited 3',5'-cyclic phosphodiesterase A |
| 21 | GSK3B | Glycogen synthase kinase-3 beta | 116 | TGFB1 | Transforming growth factor beta-1 |
| 22 | MMP1 | Interstitial collagenase | 117 | STAT1 | Signal transducer and activator of transcription 1-alpha/beta |
| 23 | PPARD | Peroxisome proliferator-activated receptor delta | 118 | GABRA1 | Gamma-aminobutyric-acid receptor subunit alpha-1 |
| 24 | ALOX12 | Arachidonate 12-lipoxygenase, 12S-type | 119 | CD14 | Monocyte differentiation antigen CD14 |
| 25 | MMP3 | Stromelysin-1 | 120 | NR3C2 | Mineralocorticoid receptor |
| 26 | IL10 | Interleukin-10 | 121 | HMOX1 | Heme oxygenase 1 |
| 27 | MDM2 | E3 ubiquitin-protein ligase Mdm2 | 122 | GSTM1 | Glutathione S-transferase Mu 1 |
| 28 | CXCL2 | C-X-C motif chemokine 2 | 123 | MAPK1 | Mitogen-activated protein kinase 1 |
| 29 | EGFR | Epidermal growth factor receptor | 124 | PLAT | Tissue-type plasminogen activator |
| 30 | SOD1 | Superoxide dismutase [Cu-Zn] | 125 | IKBKB | Inhibitor of nuclear factor kappa-B kinase subunit beta |
| 31 | ERBB2 | Receptor tyrosine-protein kinase erbB-2 | 126 | IL4 | Interleukin-4 |
| 32 | F10 | Coagulation factor X | 127 | APOD | Apolipoprotein D |
| 33 | NCOA1 | Nuclear receptor coactivator 1 | 128 | NOX5 | NADPH oxidase 5 |
| 34 | CHEK2 | Serine/threonine-protein kinase Chk2 | 129 | MAPK8 | Mitogen-activated protein kinase 8 |
| 35 | CDK2 | Cell division protein kinase 2 | 130 | AKR1B1 | Aldose reductase |
| 36 | RUNX2 | Runt-related transcription factor 2 | 131 | RB1 | Retinoblastoma-associated protein |
| 37 | PGR | Progesterone receptor | 132 | TP53 | Cellular tumor antigen p53 |
| 38 | ODC1 | Ornithine decarboxylase | 133 | CASP9 | Caspase-9 |
| 39 | CXCL10 | C-X-C motif chemokine 10 | 134 | LYZ | Lysozyme |
| 40 | CDKN1A | Cyclin-dependent kinase inhibitor 1 | 135 | SIRT1 | NAD-dependent deacetylase sirtuin-1 |
| 41 | DIO1 | Type I iodothyronine deiodinase | 136 | IL1B | Interleukin-1 beta |
| 42 | RASA1 | Ras GTPase-activating protein 1 | 137 | HTR2A | 5-hydroxytryptamine 2A receptor |
| 43 | NFKBIA | NF-kappa-B inhibitor alpha | 138 | ESR2 | Estrogen receptor beta |
| 44 | IGFBP3 | Insulin-like growth factor-binding protein 3 | 139 | CYP1B1 | Cytochrome P450 1B1 |
| 45 | ALB | Serum albumin | 140 | SLC6A3 | Sodium-dependent dopamine transporter |
| 46 | CHRNA7 | Neuronal acetylcholine receptor subunit alpha-7 | 141 | LTA4H | Leukotriene A-4 hydrolase |
| 47 | PTGER3 | Prostaglandin E2 receptor, EP3 subtype | 142 | DRD1 | D(1A) dopamine receptor |
| 48 | SLC6A2 | Sodium-dependent noradrenaline transporter | 143 | ADRA2C | Alpha-2C adrenergic receptor |
| 49 | IRF1 | Interferon regulatory factor 1 | 144 | PLAU | Urokinase-type plasminogen activator |
| 50 | RXRA | Retinoic acid receptor RXR-alpha | 145 | PTEN | Phosphatidylinositol-3,4,5-trisphosphate 3-phosphatase and dual-specificity protein phosphatase PTEN |
| 51 | ACACA | Acetyl-CoA carboxylase 1 | 146 | SLC6A4 | Sodium-dependent serotonin transporter |
| 52 | NFATC1 | Nuclear factor of activated T-cells, cytoplasmic 1 | 147 | ALOX5 | Arachidonate 5-lipoxygenase |
| 53 | PON1 | Serum paraoxonase/arylesterase 1 | 148 | GJA1 | Gap junction alpha-1 protein |
| 54 | CHEK1 | Serine/threonine-protein kinase Chk1 | 149 | DPP4 | Dipeptidyl peptidase 4 |
| 55 | SELE | E-selectin | 150 | LBP | Lipopolysaccharide-binding protein |
| 56 | THBD | Thrombomodulin | 151 | KCNH2 | Potassium voltage-gated channel subfamily H member 2 |
| 57 | MAPK14 | Mitogen-activated protein kinase 14 | 152 | E2F1 | Transcription factor E2F1 |
| 58 | MPO | Myeloperoxidase | 153 | DUOX2 | Dual oxidase 2 |
| 59 | EGLN1 | Egl nine homolog 1 | 154 | CASP8 | Caspase-8 |
| 60 | CASP7 | Caspase-7 | 155 | PPARG | Peroxisome proliferator-activated receptor gamma |
| 61 | ACHE | Acetylcholinesterase | 156 | CXCL11 | C-X-C motif chemokine 11 |
| 62 | IL6 | Interleukin-6 | 157 | CXCL8 | Interleukin-8 |
| 63 | CASP3 | Caspase-3 | 158 | NR3C1 | Glucocorticoid receptor |
| 64 | COL3A1 | Collagen alpha-1(III) chain | 159 | RAF1 | RAF proto-oncogene serine/threonine-protein kinase |
| 65 | MGAM | Maltase-glucoamylase, intestinal | 160 | ADRA1B | Alpha-1B adrenergic receptor |
| 66 | ABCG2 | ATP-binding cassette sub-family G member 2 | 161 | PCNA | Proliferating cell nuclear antigen |
| 67 | PTPN1 | Tyrosine-protein phosphatase non-receptor type 1 | 162 | CHUK | Inhibitor of nuclear factor kappa-B kinase subunit alpha |
| 68 | MAOB | Amine oxidase [flavin-containing] B | 163 | SLPI | Antileukoproteinase |
| 69 | ELK1 | ETS domain-containing protein Elk-1 | 164 | ADRB2 | Beta-2 adrenergic receptor |
| 70 | AHSA1 | Activator of 90 kDa heat shock protein ATPase homolog 1 | 165 | CTSD | Cathepsin D |
| 71 | PIK3CG | Phosphatidylinositol-4,5-bisphosphate 3-kinase catalytic subunit gamma isoform | 166 | CCL2 | Small inducible cytokine A2 |
| 72 | NQO1 | NAD(P)H dehydrogenase [quinone] 1 | 167 | HSP90AA1 | Heat shock protein HSP 90-alpha |
| 73 | IFNG | Interferon gamma | 168 | PARP1 | Poly [ADP-ribose] polymerase 1 |
| 74 | FOS | Proto-oncogene c-Fos | 169 | KDR | Vascular endothelial growth factor receptor 2 |
| 75 | OPRD1 | Delta-type opioid receptor | 170 | PPARA | Peroxisome proliferator-activated receptor alpha |
| 76 | CAV1 | Caveolin-1 | 171 | IGF2 | Insulin-like growth factor II |
| 77 | HIF1A | Hypoxia-inducible factor 1-alpha | 172 | IL2 | Interleukin-2 |
| 78 | RELA | Transcription factor p65 | 173 | HSPA5 | 78 kDa glucose-regulated protein |
| 79 | SLC2A4 | Solute carrier family 2, facilitated glucose transporter member 4 | 174 | HK2 | Hexokinase-2 |
| 80 | MAP2 | Microtubule-associated protein 2 | 175 | ICAM1 | Intercellular adhesion molecule 1 |
| 81 | NOS3 | Nitric-oxide synthase, endothelial | 176 | BCL2L1 | Bcl-2-like protein 1 |
| 82 | INSR | Insulin receptor | 177 | CA2 | Carbonic anhydrase 2 |
| 83 | AKT1 | RAC-alpha serine/threonine-protein kinase | 178 | HSF1 | Heat shock factor protein 1 |
| 84 | CYP2B6 | Cytochrome P450 2B1 | 179 | CHRM2 | Muscarinic acetylcholine receptor M2 |
| 85 | F3 | Tissue factor | 180 | OPRM1 | Mu-type opioid receptor |
| 86 | NR1I2 | Nuclear receptor subfamily 1 group I member 2 | 181 | CALM3 | Calmodulin |
| 87 | PYGM | Glycogen phosphorylase, muscle form | 182 | SERPINE1 | Plasminogen activator inhibitor 1 |
| 88 | CDKN2A | Cyclin-dependent kinase inhibitor 2A, isoforms 1/2/3 | 183 | VCAM1 | Vascular cell adhesion protein 1 |
| 89 | JUN | Transcription factor AP-1 | 184 | PTGS1 | Prostaglandin G/H synthase 1 |
| 90 | AR | Androgen receptor | 185 | BAX | Apoptosis regulator BAX |
| 91 | CD40LG | CD40 ligand | 186 | PRKCA | Protein kinase C alpha type |
| 92 | CYP3A4 | Cytochrome P450 3A4 | 187 | SCN5A | Sodium channel protein type 5 subunit alpha |
| 93 | APP | Amyloid beta A4 protein | 188 | GRIA2 | Glutamate receptor 2 |
| 94 | HSPB1 | Heat shock protein beta-1 | 189 | MMP9 | Matrix metalloproteinase-9 |
| 95 | DCAF5 | DDB1- and CUL4-associated factor 5 | 190 | TOP1 | DNA topoisomerase 1 |
