# Supplementary material for: Elucidating the mechanism of Buyang Huanwu Decoction in the treatment of ischemic stroke: A network pharmacology and molecular docking study
Source: Medicine (Baltimore). 2026 Jul 17;105(29):e49736. doi: 10.1097/MD.0000000000049736 (PMC13384647; doi:10.1097/MD.0000000000049736)
Supplement: Supplementary file 4 [file medi-105-e49736-s004.docx]

**S 4.** Correspondence among Chinese medicinal herbs, components, target proteins, and target genes.

| **Chinese medicinal herbs** | **Mol ID** | **Component name** | **Protein name** | **Gene name** |
| --- | --- | --- | --- | --- |
| huangqi | MOL000033 | (3S,8S,9S,10R,13R,14S,17R)-10,13-dimethyl-17-[(2R,5S)-5-propan-2-yloctan-2-yl]-2,3,4,7,8,9,11,12,14,15,16,17-dodecahydro-1H-cyclopenta[a]phenanthren-3-ol | Progesterone receptor | PGR |
| huangqi | MOL000098 | quercetin | Prostaglandin G/H synthase 1 | PTGS1 |
| huangqi | MOL000098 | quercetin | Insulin receptor | INSR |
| huangqi | MOL000098 | quercetin | Prothrombin | F2 |
| huangqi | MOL000098 | quercetin | Potassium voltage-gated channel subfamily H member 2 | KCNH2 |
| huangqi | MOL000098 | quercetin | Androgen receptor | AR |
| huangqi | MOL000098 | quercetin | Sodium channel protein type 5 subunit alpha | SCN5A |
| huangqi | MOL000098 | quercetin | Peroxisome proliferator-activated receptor gamma | PPARG |
| huangqi | MOL000098 | quercetin | Coagulation factor X | F10 |
| huangqi | MOL000098 | quercetin | Apoptosis regulator Bcl-2 | BCL2 |
| huangqi | MOL000098 | quercetin | Arachidonate 5-lipoxygenase | ALOX5 |
| huangqi | MOL000098 | quercetin | Prostaglandin G/H synthase 2 | PTGS2 |
| huangqi | MOL000098 | quercetin | Nitric-oxide synthase, endothelial | NOS3 |
| huangqi | MOL000098 | quercetin | Coagulation factor VII | F7 |
| huangqi | MOL000098 | quercetin | Ornithine decarboxylase | ODC1 |
| huangqi | MOL000098 | quercetin | Retinoic acid receptor RXR-alpha | RXRA |
| huangqi | MOL000098 | quercetin | Acetylcholinesterase | ACHE |
| huangqi | MOL000098 | quercetin | Acetyl-CoA carboxylase 1 | ACACA |
| huangqi | MOL000098 | quercetin | 72 kDa type IV collagenase | MMP2 |
| huangqi | MOL000098 | quercetin | Beta-2 adrenergic receptor | ADRB2 |
| huangqi | MOL000098 | quercetin | Tumor necrosis factor | TNF |
| huangqi | MOL000098 | quercetin | Aldose reductase | AKR1B1 |
| huangqi | MOL000098 | quercetin | Epidermal growth factor receptor | EGFR |
| huangqi | MOL000098 | quercetin | Gamma-aminobutyric-acid receptor subunit alpha-1 | GABRA1 |
| huangqi | MOL000098 | quercetin | Maltase-glucoamylase, intestinal | MGAM |
| huangqi | MOL000098 | quercetin | Dipeptidyl peptidase 4 | DPP4 |
| huangqi | MOL000098 | quercetin | Urokinase-type plasminogen activator | PLAU |
| huangqi | MOL000098 | quercetin | Interleukin-6 | IL6 |
| huangqi | MOL000098 | quercetin | Interstitial collagenase | MMP1 |
| huangqi | MOL000098 | quercetin | Mitogen-activated protein kinase 1 | MAPK1 |
| huangqi | MOL000098 | quercetin | Serum paraoxonase/arylesterase 1 | PON1 |
| huangqi | MOL000098 | quercetin | Cathepsin D | CTSD |
| huangqi | MOL000098 | quercetin | Interferon gamma | IFNG |
| huangqi | MOL000098 | quercetin | Transcription factor AP-1 | JUN |
| huangqi | MOL000098 | quercetin | Small inducible cytokine A2 | CCL2 |
| huangqi | MOL000098 | quercetin | Interleukin-1 beta | IL1B |
| huangqi | MOL000098 | quercetin | E-selectin | SELE |
| huangqi | MOL000098 | quercetin | Myeloperoxidase | MPO |
| huangqi | MOL000098 | quercetin | Tissue-type plasminogen activator | PLAT |
| huangqi | MOL000098 | quercetin | Gap junction alpha-1 protein | GJA1 |
| huangqi | MOL000098 | quercetin | Vascular cell adhesion protein 1 | VCAM1 |
| huangqi | MOL000098 | quercetin | Stromelysin-1 | MMP3 |
| huangqi | MOL000098 | quercetin | Heat shock protein HSP 90-alpha | HSP90AA1 |
| huangqi | MOL000098 | quercetin | Thrombomodulin | THBD |
| huangqi | MOL000098 | quercetin | Tissue factor | F3 |
| huangqi | MOL000098 | quercetin | NAD(P)H dehydrogenase [quinone] 1 | NQO1 |
| huangqi | MOL000098 | quercetin | Phosphatidylinositol-4,5-bisphosphate 3-kinase catalytic subunit gamma isoform | PIK3CG |
| huangqi | MOL000098 | quercetin | Amine oxidase [flavin-containing] B | MAOB |
| huangqi | MOL000098 | quercetin | Xanthine dehydrogenase/oxidase | XDH |
| huangqi | MOL000098 | quercetin | Prostaglandin E2 receptor, EP3 subtype | PTGER3 |
| huangqi | MOL000098 | quercetin | Superoxide dismutase [Cu-Zn] | SOD1 |
| huangqi | MOL000098 | quercetin | Cytochrome P450 3A4 | CYP3A4 |
| huangqi | MOL000098 | quercetin | Cellular tumor antigen p53 | TP53 |
| huangqi | MOL000098 | quercetin | DNA topoisomerase 1 | TOP1 |
| huangqi | MOL000098 | quercetin | Heme oxygenase 1 | HMOX1 |
| huangqi | MOL000098 | quercetin | Collagen alpha-1(III) chain | COL3A1 |
| huangqi | MOL000098 | quercetin | Retinoblastoma-associated protein | RB1 |
| huangqi | MOL000098 | quercetin | Glutathione S-transferase Mu 1 | GSTM1 |
| huangqi | MOL000098 | quercetin | 78 kDa glucose-regulated protein | HSPA5 |
| huangqi | MOL000098 | quercetin | Aryl hydrocarbon receptor | AHR |
| huangqi | MOL000098 | quercetin | Interleukin-2 | IL2 |
| huangqi | MOL000098 | quercetin | Activator of 90 kDa heat shock protein ATPase homolog 1 | AHSA1 |
| huangqi | MOL000098 | quercetin | Apoptosis regulator BAX | BAX |
| huangqi | MOL000098 | quercetin | ATP-binding cassette sub-family G member 2 | ABCG2 |
| huangqi | MOL000098 | quercetin | Baculoviral IAP repeat-containing protein 5 | BIRC5 |
| huangqi | MOL000098 | quercetin | Bcl-2-like protein 1 | BCL2L1 |
| huangqi | MOL000098 | quercetin | Caspase-3 | CASP3 |
| huangqi | MOL000098 | quercetin | Caspase-8 | CASP8 |
| huangqi | MOL000098 | quercetin | Caspase-9 | CASP9 |
| huangqi | MOL000098 | quercetin | Caveolin-1 | CAV1 |
| huangqi | MOL000098 | quercetin | CD40 ligand | CD40LG |
| huangqi | MOL000098 | quercetin | C-reactive protein | CRP |
| huangqi | MOL000098 | quercetin | C-X-C motif chemokine 10 | CXCL10 |
| huangqi | MOL000098 | quercetin | C-X-C motif chemokine 11 | CXCL11 |
| huangqi | MOL000098 | quercetin | C-X-C motif chemokine 2 | CXCL2 |
| huangqi | MOL000098 | quercetin | Cyclin-dependent kinase inhibitor 1 | CDKN1A |
| huangqi | MOL000098 | quercetin | Cyclin-dependent kinase inhibitor 2A, isoforms 1/2/3 | CDKN2A |
| huangqi | MOL000098 | quercetin | Cytochrome P450 1A1 | CYP1A1 |
| huangqi | MOL000098 | quercetin | Cytochrome P450 1B1 | CYP1B1 |
| huangqi | MOL000098 | quercetin | DDB1- and CUL4-associated factor 5 | DCAF5 |
| huangqi | MOL000098 | quercetin | Dual oxidase 2 | DUOX2 |
| huangqi | MOL000098 | quercetin | ETS domain-containing protein Elk-1 | ELK1 |
| huangqi | MOL000098 | quercetin | G1/S-specific cyclin-D1 | CCND1 |
| huangqi | MOL000098 | quercetin | Heat shock factor protein 1 | HSF1 |
| huangqi | MOL000098 | quercetin | Heat shock protein beta-1 | HSPB1 |
| huangqi | MOL000098 | quercetin | Hexokinase-2 | HK2 |
| huangqi | MOL000098 | quercetin | Hyaluronan synthase 2 | HAS2 |
| huangqi | MOL000098 | quercetin | Hypoxia-inducible factor 1-alpha | HIF1A |
| huangqi | MOL000098 | quercetin | Inhibitor of nuclear factor kappa-B kinase subunit alpha | CHUK |
| huangqi | MOL000098 | quercetin | Insulin-like growth factor-binding protein 3 | IGFBP3 |
| huangqi | MOL000098 | quercetin | Insulin-like growth factor II | IGF2 |
| huangqi | MOL000098 | quercetin | Intercellular adhesion molecule 1 | ICAM1 |
| huangqi | MOL000098 | quercetin | Interferon regulatory factor 1 | IRF1 |
| huangqi | MOL000098 | quercetin | Interleukin-10 | IL10 |
| huangqi | MOL000098 | quercetin | Interleukin-1 alpha | IL1A |
| huangqi | MOL000098 | quercetin | Interleukin-8 | CXCL8 |
| huangqi | MOL000098 | quercetin | Matrix metalloproteinase-9 | MMP9 |
| huangqi | MOL000098 | quercetin | Myc proto-oncogene protein | MYC |
| huangqi | MOL000098 | quercetin | Neutrophil cytosol factor 1 | NCF1 |
| huangqi | MOL000098 | quercetin | NF-kappa-B inhibitor alpha | NFKBIA |
| huangqi | MOL000098 | quercetin | Nuclear factor erythroid 2-related factor 2 | NFE2L2 |
| huangqi | MOL000098 | quercetin | Nuclear receptor subfamily 1 group I member 2 | NR1I2 |
| huangqi | MOL000098 | quercetin | Osteopontin | SPP1 |
| huangqi | MOL000098 | quercetin | Peroxisome proliferator-activated receptor alpha | PPARA |
| huangqi | MOL000098 | quercetin | Peroxisome proliferator-activated receptor delta | PPARD |
| huangqi | MOL000098 | quercetin | Peroxisome proliferator-activated receptor gamma | PPARG |
| huangqi | MOL000098 | quercetin | Plasminogen activator inhibitor 1 | SERPINE1 |
| huangqi | MOL000098 | quercetin | Poly [ADP-ribose] polymerase 1 | PARP1 |
| huangqi | MOL000098 | quercetin | Procollagen C-endopeptidase enhancer 1 | PCOLCE |
| huangqi | MOL000098 | quercetin | Protein kinase C alpha type | PRKCA |
| huangqi | MOL000098 | quercetin | Protein kinase C beta type | PRKCB |
| huangqi | MOL000098 | quercetin | Proto-oncogene c-Fos | FOS |
| huangqi | MOL000098 | quercetin | RAC-alpha serine/threonine-protein kinase | AKT1 |
| huangqi | MOL000098 | quercetin | RAF proto-oncogene serine/threonine-protein kinase | RAF1 |
| huangqi | MOL000098 | quercetin | Ras GTPase-activating protein 1 | RASA1 |
| huangqi | MOL000098 | quercetin | Receptor tyrosine-protein kinase erbB-2 | ERBB2 |
| huangqi | MOL000098 | quercetin | Runt-related transcription factor 2 | RUNX2 |
| huangqi | MOL000098 | quercetin | Serine/threonine-protein kinase Chk2 | CHEK2 |
| huangqi | MOL000098 | quercetin | Signal transducer and activator of transcription 1-alpha/beta | STAT1 |
| huangqi | MOL000098 | quercetin | Solute carrier family 2, facilitated glucose transporter member 4 | SLC2A4 |
| huangqi | MOL000098 | quercetin | Transcription factor E2F1 | E2F1 |
| huangqi | MOL000098 | quercetin | Transcription factor p65 | RELA |
| huangqi | MOL000098 | quercetin | Transforming growth factor beta-1 | TGFB1 |
| huangqi | MOL000098 | quercetin | Type I iodothyronine deiodinase | DIO1 |
| huangqi | MOL000098 | quercetin | Phosphatidylinositol-3,4,5-trisphosphate 3-phosphatase and dual-specificity protein phosphatase PTEN | PTEN |
| huangqi | MOL000211 | Mairin | Progesterone receptor | PGR |
| huangqi | MOL000239 | Jaranol | Nitric oxide synthase, inducible | NOS2 |
| huangqi | MOL000239 | Jaranol | Prostaglandin G/H synthase 1 | PTGS1 |
| huangqi | MOL000239 | Jaranol | Androgen receptor | AR |
| huangqi | MOL000239 | Jaranol | Sodium channel protein type 5 subunit alpha | SCN5A |
| huangqi | MOL000239 | Jaranol | Prostaglandin G/H synthase 2 | PTGS2 |
| huangqi | MOL000239 | Jaranol | Estrogen receptor beta | ESR2 |
| huangqi | MOL000239 | Jaranol | Dipeptidyl peptidase 4 | DPP4 |
| huangqi | MOL000239 | Jaranol | Heat shock protein HSP 90-alpha | HSP90AA1 |
| huangqi | MOL000239 | Jaranol | Cell division protein kinase 2 | CDK2 |
| huangqi | MOL000239 | Jaranol | Serine/threonine-protein kinase Chk1 | CHEK1 |
| huangqi | MOL000239 | Jaranol | Calmodulin | CALM3 |
| huangqi | MOL000296 | hederagenin | Prostaglandin G/H synthase 1 | PTGS1 |
| huangqi | MOL000296 | hederagenin | Muscarinic acetylcholine receptor M1 | CHRM1 |
| huangqi | MOL000296 | hederagenin | Sodium channel protein type 5 subunit alpha | SCN5A |
| huangqi | MOL000296 | hederagenin | Prostaglandin G/H synthase 2 | PTGS2 |
| huangqi | MOL000296 | hederagenin | Retinoic acid receptor RXR-alpha | RXRA |
| huangqi | MOL000296 | hederagenin | cGMP-inhibited 3',5'-cyclic phosphodiesterase A | PDE3A |
| huangqi | MOL000296 | hederagenin | Sodium-dependent noradrenaline transporter | SLC6A2 |
| huangqi | MOL000296 | hederagenin | Progesterone receptor | PGR |
| huangqi | MOL000296 | hederagenin | Muscarinic acetylcholine receptor M2 | CHRM2 |
| huangqi | MOL000296 | hederagenin | Alpha-1B adrenergic receptor | ADRA1B |
| huangqi | MOL000296 | hederagenin | Gamma-aminobutyric-acid receptor subunit alpha-1 | GABRA1 |
| huangqi | MOL000296 | hederagenin | Lysozyme | LYZ |
| huangqi | MOL000296 | hederagenin | Glutamate receptor 2 | GRIA2 |
| huangqi | MOL000354 | isorhamnetin | Nitric oxide synthase, inducible | NOS2 |
| huangqi | MOL000354 | isorhamnetin | Prostaglandin G/H synthase 1 | PTGS1 |
| huangqi | MOL000354 | isorhamnetin | Prothrombin | F2 |
| huangqi | MOL000354 | isorhamnetin | Estrogen receptor | ESR1 |
| huangqi | MOL000354 | isorhamnetin | Androgen receptor | AR |
| huangqi | MOL000354 | isorhamnetin | Peroxisome proliferator-activated receptor gamma | PPARG |
| huangqi | MOL000354 | isorhamnetin | Prostaglandin G/H synthase 2 | PTGS2 |
| huangqi | MOL000354 | isorhamnetin | Nitric-oxide synthase, endothelial | NOS3 |
| huangqi | MOL000354 | isorhamnetin | Coagulation factor VII | F7 |
| huangqi | MOL000354 | isorhamnetin | Acetylcholinesterase | ACHE |
| huangqi | MOL000354 | isorhamnetin | Tyrosine-protein phosphatase non-receptor type 1 | PTPN1 |
| huangqi | MOL000354 | isorhamnetin | Aldose reductase | AKR1B1 |
| huangqi | MOL000354 | isorhamnetin | Estrogen receptor beta | ESR2 |
| huangqi | MOL000354 | isorhamnetin | Gamma-aminobutyric-acid receptor subunit alpha-1 | GABRA1 |
| huangqi | MOL000354 | isorhamnetin | Dipeptidyl peptidase 4 | DPP4 |
| huangqi | MOL000354 | isorhamnetin | Glycogen phosphorylase, muscle form | PYGM |
| huangqi | MOL000354 | isorhamnetin | Peroxisome proliferator-activated receptor delta | PPARD |
| huangqi | MOL000354 | isorhamnetin | Mitogen-activated protein kinase 14 | MAPK14 |
| huangqi | MOL000354 | isorhamnetin | Glycogen synthase kinase-3 beta | GSK3B |
| huangqi | MOL000354 | isorhamnetin | Heat shock protein HSP 90-alpha | HSP90AA1 |
| huangqi | MOL000354 | isorhamnetin | Cell division protein kinase 2 | CDK2 |
| huangqi | MOL000354 | isorhamnetin | Phosphatidylinositol-4,5-bisphosphate 3-kinase catalytic subunit gamma isoform | PIK3CG |
| huangqi | MOL000354 | isorhamnetin | Amine oxidase [flavin-containing] B | MAOB |
| huangqi | MOL000354 | isorhamnetin | Xanthine dehydrogenase/oxidase | XDH |
| huangqi | MOL000354 | isorhamnetin | Serine/threonine-protein kinase Chk1 | CHEK1 |
| huangqi | MOL000354 | isorhamnetin | Cyclin-A2 | CCNA2 |
| huangqi | MOL000354 | isorhamnetin | Glutamate receptor 2 | GRIA2 |
| huangqi | MOL000354 | isorhamnetin | Nuclear receptor coactivator 1 | NCOA1 |
| huangqi | MOL000354 | isorhamnetin | Calmodulin | CALM3 |
| huangqi | MOL000354 | isorhamnetin | Neutrophil cytosol factor 1 | NCF1 |
| huangqi | MOL000354 | isorhamnetin | Oxidized low-density lipoprotein receptor 1 | OLR1 |
| huangqi | MOL000354 | isorhamnetin | Transcription factor p65 | RELA |
| huangqi | MOL000371 | 3,9-di-O-methylnissolin | Nitric oxide synthase, inducible | NOS2 |
| huangqi | MOL000371 | 3,9-di-O-methylnissolin | Prostaglandin G/H synthase 1 | PTGS1 |
| huangqi | MOL000371 | 3,9-di-O-methylnissolin | Prothrombin | F2 |
| huangqi | MOL000371 | 3,9-di-O-methylnissolin | Muscarinic acetylcholine receptor M1 | CHRM1 |
| huangqi | MOL000371 | 3,9-di-O-methylnissolin | Estrogen receptor | ESR1 |
| huangqi | MOL000371 | 3,9-di-O-methylnissolin | Beta-1 adrenergic receptor | ADRB1 |
| huangqi | MOL000371 | 3,9-di-O-methylnissolin | Sodium channel protein type 5 subunit alpha | SCN5A |
| huangqi | MOL000371 | 3,9-di-O-methylnissolin | Prostaglandin G/H synthase 2 | PTGS2 |
| huangqi | MOL000371 | 3,9-di-O-methylnissolin | Nitric-oxide synthase, endothelial | NOS3 |
| huangqi | MOL000371 | 3,9-di-O-methylnissolin | Alpha-2C adrenergic receptor | ADRA2C |
| huangqi | MOL000371 | 3,9-di-O-methylnissolin | Retinoic acid receptor RXR-alpha | RXRA |
| huangqi | MOL000371 | 3,9-di-O-methylnissolin | Acetylcholinesterase | ACHE |
| huangqi | MOL000371 | 3,9-di-O-methylnissolin | cGMP-inhibited 3',5'-cyclic phosphodiesterase A | PDE3A |
| huangqi | MOL000371 | 3,9-di-O-methylnissolin | Alpha-1B adrenergic receptor | ADRA1B |
| huangqi | MOL000371 | 3,9-di-O-methylnissolin | Beta-2 adrenergic receptor | ADRB2 |
| huangqi | MOL000371 | 3,9-di-O-methylnissolin | Mu-type opioid receptor | OPRM1 |
| huangqi | MOL000371 | 3,9-di-O-methylnissolin | Gamma-aminobutyric-acid receptor subunit alpha-1 | GABRA1 |
| huangqi | MOL000371 | 3,9-di-O-methylnissolin | Calmodulin | CALM3 |
| huangqi | MOL000378 | 7-O-methylisomucronulatol | Nitric oxide synthase, inducible | NOS2 |
| huangqi | MOL000378 | 7-O-methylisomucronulatol | Prostaglandin G/H synthase 1 | PTGS1 |
| huangqi | MOL000378 | 7-O-methylisomucronulatol | D(1A) dopamine receptor | DRD1 |
| huangqi | MOL000378 | 7-O-methylisomucronulatol | Prothrombin | F2 |
| huangqi | MOL000378 | 7-O-methylisomucronulatol | Potassium voltage-gated channel subfamily H member 2 | KCNH2 |
| huangqi | MOL000378 | 7-O-methylisomucronulatol | Muscarinic acetylcholine receptor M1 | CHRM1 |
| huangqi | MOL000378 | 7-O-methylisomucronulatol | Estrogen receptor | ESR1 |
| huangqi | MOL000378 | 7-O-methylisomucronulatol | Androgen receptor | AR |
| huangqi | MOL000378 | 7-O-methylisomucronulatol | Beta-1 adrenergic receptor | ADRB1 |
| huangqi | MOL000378 | 7-O-methylisomucronulatol | Sodium channel protein type 5 subunit alpha | SCN5A |
| huangqi | MOL000378 | 7-O-methylisomucronulatol | Peroxisome proliferator-activated receptor gamma | PPARG |
| huangqi | MOL000378 | 7-O-methylisomucronulatol | Coagulation factor X | F10 |
| huangqi | MOL000378 | 7-O-methylisomucronulatol | Prostaglandin G/H synthase 2 | PTGS2 |
| huangqi | MOL000378 | 7-O-methylisomucronulatol | Nitric-oxide synthase, endothelial | NOS3 |
| huangqi | MOL000378 | 7-O-methylisomucronulatol | Alpha-2C adrenergic receptor | ADRA2C |
| huangqi | MOL000378 | 7-O-methylisomucronulatol | Retinoic acid receptor RXR-alpha | RXRA |
| huangqi | MOL000378 | 7-O-methylisomucronulatol | Delta-type opioid receptor | OPRD1 |
| huangqi | MOL000378 | 7-O-methylisomucronulatol | cGMP-inhibited 3',5'-cyclic phosphodiesterase A | PDE3A |
| huangqi | MOL000378 | 7-O-methylisomucronulatol | 5-hydroxytryptamine 2A receptor | HTR2A |
| huangqi | MOL000378 | 7-O-methylisomucronulatol | Muscarinic acetylcholine receptor M2 | CHRM2 |
| huangqi | MOL000378 | 7-O-methylisomucronulatol | Alpha-1B adrenergic receptor | ADRA1B |
| huangqi | MOL000378 | 7-O-methylisomucronulatol | Sodium-dependent dopamine transporter | SLC6A3 |
| huangqi | MOL000378 | 7-O-methylisomucronulatol | Beta-2 adrenergic receptor | ADRB2 |
| huangqi | MOL000378 | 7-O-methylisomucronulatol | Sodium-dependent serotonin transporter | SLC6A4 |
| huangqi | MOL000378 | 7-O-methylisomucronulatol | Estrogen receptor beta | ESR2 |
| huangqi | MOL000378 | 7-O-methylisomucronulatol | Gamma-aminobutyric-acid receptor subunit alpha-1 | GABRA1 |
| huangqi | MOL000378 | 7-O-methylisomucronulatol | Dipeptidyl peptidase 4 | DPP4 |
| huangqi | MOL000378 | 7-O-methylisomucronulatol | Mitogen-activated protein kinase 14 | MAPK14 |
| huangqi | MOL000378 | 7-O-methylisomucronulatol | Glycogen synthase kinase-3 beta | GSK3B |
| huangqi | MOL000378 | 7-O-methylisomucronulatol | Heat shock protein HSP 90-alpha | HSP90AA1 |
| huangqi | MOL000378 | 7-O-methylisomucronulatol | Cell division protein kinase 2 | CDK2 |
| huangqi | MOL000378 | 7-O-methylisomucronulatol | Serine/threonine-protein kinase Chk1 | CHEK1 |
| huangqi | MOL000378 | 7-O-methylisomucronulatol | Cyclin-A2 | CCNA2 |
| huangqi | MOL000378 | 7-O-methylisomucronulatol | Calcium-activated potassium channel subunit alpha 1 | KCNMA1 |
| huangqi | MOL000378 | 7-O-methylisomucronulatol | Calmodulin | CALM3 |
| huangqi | MOL000379 | 9,10-dimethoxypterocarpan-3-O-β-D-glucoside | Prostaglandin G/H synthase 2 | PTGS2 |
| huangqi | MOL000380 | (6aR,11aR)-9,10-dimethoxy-6a,11a-dihydro-6H-benzofurano[3,2-c]chromen-3-ol | Nitric oxide synthase, inducible | NOS2 |
| huangqi | MOL000380 | (6aR,11aR)-9,10-dimethoxy-6a,11a-dihydro-6H-benzofurano[3,2-c]chromen-3-ol | Prostaglandin G/H synthase 1 | PTGS1 |
| huangqi | MOL000380 | (6aR,11aR)-9,10-dimethoxy-6a,11a-dihydro-6H-benzofurano[3,2-c]chromen-3-ol | Prothrombin | F2 |
| huangqi | MOL000380 | (6aR,11aR)-9,10-dimethoxy-6a,11a-dihydro-6H-benzofurano[3,2-c]chromen-3-ol | Muscarinic acetylcholine receptor M1 | CHRM1 |
| huangqi | MOL000380 | (6aR,11aR)-9,10-dimethoxy-6a,11a-dihydro-6H-benzofurano[3,2-c]chromen-3-ol | Estrogen receptor | ESR1 |
| huangqi | MOL000380 | (6aR,11aR)-9,10-dimethoxy-6a,11a-dihydro-6H-benzofurano[3,2-c]chromen-3-ol | Sodium channel protein type 5 subunit alpha | SCN5A |
| huangqi | MOL000380 | (6aR,11aR)-9,10-dimethoxy-6a,11a-dihydro-6H-benzofurano[3,2-c]chromen-3-ol | Prostaglandin G/H synthase 2 | PTGS2 |
| huangqi | MOL000380 | (6aR,11aR)-9,10-dimethoxy-6a,11a-dihydro-6H-benzofurano[3,2-c]chromen-3-ol | Retinoic acid receptor RXR-alpha | RXRA |
| huangqi | MOL000380 | (6aR,11aR)-9,10-dimethoxy-6a,11a-dihydro-6H-benzofurano[3,2-c]chromen-3-ol | Acetylcholinesterase | ACHE |
| huangqi | MOL000380 | (6aR,11aR)-9,10-dimethoxy-6a,11a-dihydro-6H-benzofurano[3,2-c]chromen-3-ol | Alpha-1B adrenergic receptor | ADRA1B |
| huangqi | MOL000380 | (6aR,11aR)-9,10-dimethoxy-6a,11a-dihydro-6H-benzofurano[3,2-c]chromen-3-ol | Beta-2 adrenergic receptor | ADRB2 |
| huangqi | MOL000380 | (6aR,11aR)-9,10-dimethoxy-6a,11a-dihydro-6H-benzofurano[3,2-c]chromen-3-ol | Gamma-aminobutyric-acid receptor subunit alpha-1 | GABRA1 |
| huangqi | MOL000380 | (6aR,11aR)-9,10-dimethoxy-6a,11a-dihydro-6H-benzofurano[3,2-c]chromen-3-ol | Heat shock protein HSP 90-alpha | HSP90AA1 |
| huangqi | MOL000380 | (6aR,11aR)-9,10-dimethoxy-6a,11a-dihydro-6H-benzofurano[3,2-c]chromen-3-ol | Neuronal acetylcholine receptor subunit alpha-7 | CHRNA7 |
| huangqi | MOL000380 | (6aR,11aR)-9,10-dimethoxy-6a,11a-dihydro-6H-benzofurano[3,2-c]chromen-3-ol | Nuclear receptor coactivator 1 | NCOA1 |
| huangqi | MOL000380 | (6aR,11aR)-9,10-dimethoxy-6a,11a-dihydro-6H-benzofurano[3,2-c]chromen-3-ol | Calmodulin | CALM3 |
| huangqi | MOL000387 | Bifendate | Prostaglandin G/H synthase 1 | PTGS1 |
| huangqi | MOL000387 | Bifendate | Prostaglandin G/H synthase 2 | PTGS2 |
| huangqi | MOL000387 | Bifendate | Vascular endothelial growth factor receptor 2 | KDR |
| huangqi | MOL000387 | Bifendate | Hepatocyte growth factor receptor | MET |
| huangqi | MOL000387 | Bifendate | Heat shock protein HSP 90-alpha | HSP90AA1 |
| huangqi | MOL000387 | Bifendate | Calcium-activated potassium channel subunit alpha 1 | KCNMA1 |
| huangqi | MOL000392 | formononetin | Nitric oxide synthase, inducible | NOS2 |
| huangqi | MOL000392 | formononetin | Prostaglandin G/H synthase 1 | PTGS1 |
| huangqi | MOL000392 | formononetin | Prothrombin | F2 |
| huangqi | MOL000392 | formononetin | Muscarinic acetylcholine receptor M1 | CHRM1 |
| huangqi | MOL000392 | formononetin | Estrogen receptor | ESR1 |
| huangqi | MOL000392 | formononetin | Androgen receptor | AR |
| huangqi | MOL000392 | formononetin | Peroxisome proliferator-activated receptor gamma | PPARG |
| huangqi | MOL000392 | formononetin | Prostaglandin G/H synthase 2 | PTGS2 |
| huangqi | MOL000392 | formononetin | Nitric-oxide synthase, endothelial | NOS3 |
| huangqi | MOL000392 | formononetin | Retinoic acid receptor RXR-alpha | RXRA |
| huangqi | MOL000392 | formononetin | Acetylcholinesterase | ACHE |
| huangqi | MOL000392 | formononetin | cGMP-inhibited 3',5'-cyclic phosphodiesterase A | PDE3A |
| huangqi | MOL000392 | formononetin | Sodium-dependent dopamine transporter | SLC6A3 |
| huangqi | MOL000392 | formononetin | Beta-2 adrenergic receptor | ADRB2 |
| huangqi | MOL000392 | formononetin | Sodium-dependent serotonin transporter | SLC6A4 |
| huangqi | MOL000392 | formononetin | Estrogen receptor beta | ESR2 |
| huangqi | MOL000392 | formononetin | Dipeptidyl peptidase 4 | DPP4 |
| huangqi | MOL000392 | formononetin | Mitogen-activated protein kinase 14 | MAPK14 |
| huangqi | MOL000392 | formononetin | Transcription factor AP-1 | JUN |
| huangqi | MOL000392 | formononetin | Glycogen synthase kinase-3 beta | GSK3B |
| huangqi | MOL000392 | formononetin | Heat shock protein HSP 90-alpha | HSP90AA1 |
| huangqi | MOL000392 | formononetin | Cell division protein kinase 2 | CDK2 |
| huangqi | MOL000392 | formononetin | Amine oxidase [flavin-containing] B | MAOB |
| huangqi | MOL000392 | formononetin | Serine/threonine-protein kinase Chk1 | CHEK1 |
| huangqi | MOL000392 | formononetin | NADH-ubiquinone oxidoreductase chain 6 | MT-ND6 |
| huangqi | MOL000392 | formononetin | Cyclin-A2 | CCNA2 |
| huangqi | MOL000392 | formononetin | Calmodulin | CALM3 |
| huangqi | MOL000392 | formononetin | Interleukin-4 | IL4 |
| huangqi | MOL000392 | formononetin | NAD-dependent deacetylase sirtuin-1 | SIRT1 |
| huangqi | MOL000392 | formononetin | Peroxisome proliferator-activated receptor gamma | PPARG |
| huangqi | MOL000417 | Calycosin | Nitric oxide synthase, inducible | NOS2 |
| huangqi | MOL000417 | Calycosin | Prostaglandin G/H synthase 1 | PTGS1 |
| huangqi | MOL000417 | Calycosin | Estrogen receptor | ESR1 |
| huangqi | MOL000417 | Calycosin | Androgen receptor | AR |
| huangqi | MOL000417 | Calycosin | Peroxisome proliferator-activated receptor gamma | PPARG |
| huangqi | MOL000417 | Calycosin | Prostaglandin G/H synthase 2 | PTGS2 |
| huangqi | MOL000417 | Calycosin | Retinoic acid receptor RXR-alpha | RXRA |
| huangqi | MOL000417 | Calycosin | cGMP-inhibited 3',5'-cyclic phosphodiesterase A | PDE3A |
| huangqi | MOL000417 | Calycosin | Beta-2 adrenergic receptor | ADRB2 |
| huangqi | MOL000417 | Calycosin | Estrogen receptor beta | ESR2 |
| huangqi | MOL000417 | Calycosin | Dipeptidyl peptidase 4 | DPP4 |
| huangqi | MOL000417 | Calycosin | Mitogen-activated protein kinase 14 | MAPK14 |
| huangqi | MOL000417 | Calycosin | Glycogen synthase kinase-3 beta | GSK3B |
| huangqi | MOL000417 | Calycosin | Heat shock protein HSP 90-alpha | HSP90AA1 |
| huangqi | MOL000417 | Calycosin | Cell division protein kinase 2 | CDK2 |
| huangqi | MOL000417 | Calycosin | Serine/threonine-protein kinase Chk1 | CHEK1 |
| huangqi | MOL000417 | Calycosin | Cyclin-A2 | CCNA2 |
| huangqi | MOL000417 | Calycosin | Calmodulin | CALM3 |
| huangqi | MOL000422 | kaempferol | Nitric oxide synthase, inducible | NOS2 |
| huangqi | MOL000422 | kaempferol | Prostaglandin G/H synthase 1 | PTGS1 |
| huangqi | MOL000422 | kaempferol | Insulin receptor | INSR |
| huangqi | MOL000422 | kaempferol | Prothrombin | F2 |
| huangqi | MOL000422 | kaempferol | Muscarinic acetylcholine receptor M1 | CHRM1 |
| huangqi | MOL000422 | kaempferol | Androgen receptor | AR |
| huangqi | MOL000422 | kaempferol | Peroxisome proliferator-activated receptor gamma | PPARG |
| huangqi | MOL000422 | kaempferol | Apoptosis regulator Bcl-2 | BCL2 |
| huangqi | MOL000422 | kaempferol | Arachidonate 5-lipoxygenase | ALOX5 |
| huangqi | MOL000422 | kaempferol | Prostaglandin G/H synthase 2 | PTGS2 |
| huangqi | MOL000422 | kaempferol | Nitric-oxide synthase, endothelial | NOS3 |
| huangqi | MOL000422 | kaempferol | Coagulation factor VII | F7 |
| huangqi | MOL000422 | kaempferol | Acetylcholinesterase | ACHE |
| huangqi | MOL000422 | kaempferol | Sodium-dependent noradrenaline transporter | SLC6A2 |
| huangqi | MOL000422 | kaempferol | Progesterone receptor | PGR |
| huangqi | MOL000422 | kaempferol | Muscarinic acetylcholine receptor M2 | CHRM2 |
| huangqi | MOL000422 | kaempferol | Alpha-1B adrenergic receptor | ADRA1B |
| huangqi | MOL000422 | kaempferol | Tumor necrosis factor | TNF |
| huangqi | MOL000422 | kaempferol | Gamma-aminobutyric-acid receptor subunit alpha-1 | GABRA1 |
| huangqi | MOL000422 | kaempferol | Dipeptidyl peptidase 4 | DPP4 |
| huangqi | MOL000422 | kaempferol | Interstitial collagenase | MMP1 |
| huangqi | MOL000422 | kaempferol | Transcription factor AP-1 | JUN |
| huangqi | MOL000422 | kaempferol | E-selectin | SELE |
| huangqi | MOL000422 | kaempferol | Vascular cell adhesion protein 1 | VCAM1 |
| huangqi | MOL000422 | kaempferol | Heat shock protein HSP 90-alpha | HSP90AA1 |
| huangqi | MOL000422 | kaempferol | Phosphatidylinositol-4,5-bisphosphate 3-kinase catalytic subunit gamma isoform | PIK3CG |
| huangqi | MOL000422 | kaempferol | Xanthine dehydrogenase/oxidase | XDH |
| huangqi | MOL000422 | kaempferol | Cytochrome P450 3A4 | CYP3A4 |
| huangqi | MOL000422 | kaempferol | Mitogen-activated protein kinase 8 | MAPK8 |
| huangqi | MOL000422 | kaempferol | Heme oxygenase 1 | HMOX1 |
| huangqi | MOL000422 | kaempferol | Glutathione S-transferase Mu 1 | GSTM1 |
| huangqi | MOL000422 | kaempferol | Aryl hydrocarbon receptor | AHR |
| huangqi | MOL000422 | kaempferol | Calmodulin | CALM3 |
| huangqi | MOL000422 | kaempferol | Serine/threonine-protein phosphatase 2B catalytic subunit alpha isoform | PPP3CA |
| huangqi | MOL000422 | kaempferol | Activator of 90 kDa heat shock protein ATPase homolog 1 | AHSA1 |
| huangqi | MOL000422 | kaempferol | Antileukoproteinase | SLPI |
| huangqi | MOL000422 | kaempferol | Apoptosis regulator BAX | BAX |
| huangqi | MOL000422 | kaempferol | Caspase-3 | CASP3 |
| huangqi | MOL000422 | kaempferol | Cytochrome P450 1A1 | CYP1A1 |
| huangqi | MOL000422 | kaempferol | Cytochrome P450 1B1 | CYP1B1 |
| huangqi | MOL000422 | kaempferol | Hyaluronan synthase 2 | HAS2 |
| huangqi | MOL000422 | kaempferol | Inhibitor of nuclear factor kappa-B kinase subunit beta | IKBKB |
| huangqi | MOL000422 | kaempferol | Intercellular adhesion molecule 1 | ICAM1 |
| huangqi | MOL000422 | kaempferol | Nuclear receptor subfamily 1 group I member 2 | NR1I2 |
| huangqi | MOL000422 | kaempferol | Peroxisome proliferator-activated receptor gamma | PPARG |
| huangqi | MOL000422 | kaempferol | RAC-alpha serine/threonine-protein kinase | AKT1 |
| huangqi | MOL000422 | kaempferol | Signal transducer and activator of transcription 1-alpha/beta | STAT1 |
| huangqi | MOL000422 | kaempferol | Solute carrier family 2, facilitated glucose transporter member 4 | SLC2A4 |
| huangqi | MOL000422 | kaempferol | Transcription factor p65 | RELA |
| huangqi | MOL000422 | kaempferol | Type I iodothyronine deiodinase | DIO1 |
| huangqi | MOL000433 | FA | Prothrombin | F2 |
| huangqi | MOL000433 | FA | Glycogen synthase kinase-3 beta | GSK3B |
| huangqi | MOL000433 | FA | Cell division protein kinase 2 | CDK2 |
| huangqi | MOL000442 | 1,7-Dihydroxy-3,9-dimethoxy pterocarpene | Prostaglandin G/H synthase 2 | PTGS2 |
| huangqi | MOL000442 | 1,7-Dihydroxy-3,9-dimethoxy pterocarpene | Retinoic acid receptor RXR-alpha | RXRA |
| huangqi | MOL000442 | 1,7-Dihydroxy-3,9-dimethoxy pterocarpene | Heat shock protein HSP 90-alpha | HSP90AA1 |
| danggui | MOL000358 | beta-sitosterol | Prostaglandin G/H synthase 1 | PTGS1 |
| danggui | MOL000358 | beta-sitosterol | D(1A) dopamine receptor | DRD1 |
| danggui | MOL000358 | beta-sitosterol | Potassium voltage-gated channel subfamily H member 2 | KCNH2 |
| danggui | MOL000358 | beta-sitosterol | Muscarinic acetylcholine receptor M1 | CHRM1 |
| danggui | MOL000358 | beta-sitosterol | Sodium channel protein type 5 subunit alpha | SCN5A |
| danggui | MOL000358 | beta-sitosterol | Apoptosis regulator Bcl-2 | BCL2 |
| danggui | MOL000358 | beta-sitosterol | Prostaglandin G/H synthase 2 | PTGS2 |
| danggui | MOL000358 | beta-sitosterol | cGMP-inhibited 3',5'-cyclic phosphodiesterase A | PDE3A |
| danggui | MOL000358 | beta-sitosterol | 5-hydroxytryptamine 2A receptor | HTR2A |
| danggui | MOL000358 | beta-sitosterol | Progesterone receptor | PGR |
| danggui | MOL000358 | beta-sitosterol | Muscarinic acetylcholine receptor M2 | CHRM2 |
| danggui | MOL000358 | beta-sitosterol | Alpha-1B adrenergic receptor | ADRA1B |
| danggui | MOL000358 | beta-sitosterol | Beta-2 adrenergic receptor | ADRB2 |
| danggui | MOL000358 | beta-sitosterol | Neuronal acetylcholine receptor subunit alpha-2 | CHRNA2 |
| danggui | MOL000358 | beta-sitosterol | Sodium-dependent serotonin transporter | SLC6A4 |
| danggui | MOL000358 | beta-sitosterol | Mu-type opioid receptor | OPRM1 |
| danggui | MOL000358 | beta-sitosterol | Gamma-aminobutyric-acid receptor subunit alpha-1 | GABRA1 |
| danggui | MOL000358 | beta-sitosterol | Serum paraoxonase/arylesterase 1 | PON1 |
| danggui | MOL000358 | beta-sitosterol | Transcription factor AP-1 | JUN |
| danggui | MOL000358 | beta-sitosterol | Heat shock protein HSP 90-alpha | HSP90AA1 |
| danggui | MOL000358 | beta-sitosterol | Phosphatidylinositol-4,5-bisphosphate 3-kinase catalytic subunit gamma isoform | PIK3CG |
| danggui | MOL000358 | beta-sitosterol | Neuronal acetylcholine receptor subunit alpha-7 | CHRNA7 |
| danggui | MOL000358 | beta-sitosterol | Microtubule-associated protein 2 | MAP2 |
| danggui | MOL000358 | beta-sitosterol | Apoptosis regulator BAX | BAX |
| danggui | MOL000358 | beta-sitosterol | Caspase-3 | CASP3 |
| danggui | MOL000358 | beta-sitosterol | Caspase-8 | CASP8 |
| danggui | MOL000358 | beta-sitosterol | Caspase-9 | CASP9 |
| danggui | MOL000358 | beta-sitosterol | Protein kinase C alpha type | PRKCA |
| danggui | MOL000358 | beta-sitosterol | Transforming growth factor beta-1 | TGFB1 |
| danggui | MOL000449 | Stigmasterol | Prostaglandin G/H synthase 1 | PTGS1 |
| danggui | MOL000449 | Stigmasterol | Muscarinic acetylcholine receptor M1 | CHRM1 |
| danggui | MOL000449 | Stigmasterol | Beta-1 adrenergic receptor | ADRB1 |
| danggui | MOL000449 | Stigmasterol | Sodium channel protein type 5 subunit alpha | SCN5A |
| danggui | MOL000449 | Stigmasterol | Prostaglandin G/H synthase 2 | PTGS2 |
| danggui | MOL000449 | Stigmasterol | Alpha-2A adrenergic receptor | ADRA2A |
| danggui | MOL000449 | Stigmasterol | Retinoic acid receptor RXR-alpha | RXRA |
| danggui | MOL000449 | Stigmasterol | 5-hydroxytryptamine 2A receptor | HTR2A |
| danggui | MOL000449 | Stigmasterol | Sodium-dependent noradrenaline transporter | SLC6A2 |
| danggui | MOL000449 | Stigmasterol | Progesterone receptor | PGR |
| danggui | MOL000449 | Stigmasterol | Muscarinic acetylcholine receptor M2 | CHRM2 |
| danggui | MOL000449 | Stigmasterol | Alpha-1B adrenergic receptor | ADRA1B |
| danggui | MOL000449 | Stigmasterol | Sodium-dependent dopamine transporter | SLC6A3 |
| danggui | MOL000449 | Stigmasterol | Mineralocorticoid receptor | NR3C2 |
| danggui | MOL000449 | Stigmasterol | Beta-2 adrenergic receptor | ADRB2 |
| danggui | MOL000449 | Stigmasterol | Aldose reductase | AKR1B1 |
| danggui | MOL000449 | Stigmasterol | Gamma-aminobutyric-acid receptor subunit alpha-1 | GABRA1 |
| danggui | MOL000449 | Stigmasterol | Urokinase-type plasminogen activator | PLAU |
| danggui | MOL000449 | Stigmasterol | Leukotriene A-4 hydrolase | LTA4H |
| danggui | MOL000449 | Stigmasterol | Amine oxidase [flavin-containing] B | MAOB |
| danggui | MOL000449 | Stigmasterol | Amine oxidase [flavin-containing] A | MAOA |
| danggui | MOL000449 | Stigmasterol | Neuronal acetylcholine receptor subunit alpha-7 | CHRNA7 |
| danggui | MOL000449 | Stigmasterol | Nuclear receptor coactivator 1 | NCOA1 |
| chishao | MOL000358 | beta-sitosterol | Prostaglandin G/H synthase 1 | PTGS1 |
| chishao | MOL000358 | beta-sitosterol | D(1A) dopamine receptor | DRD1 |
| chishao | MOL000358 | beta-sitosterol | Potassium voltage-gated channel subfamily H member 2 | KCNH2 |
| chishao | MOL000358 | beta-sitosterol | Muscarinic acetylcholine receptor M1 | CHRM1 |
| chishao | MOL000358 | beta-sitosterol | Sodium channel protein type 5 subunit alpha | SCN5A |
| chishao | MOL000358 | beta-sitosterol | Apoptosis regulator Bcl-2 | BCL2 |
| chishao | MOL000358 | beta-sitosterol | Prostaglandin G/H synthase 2 | PTGS2 |
| chishao | MOL000358 | beta-sitosterol | cGMP-inhibited 3',5'-cyclic phosphodiesterase A | PDE3A |
| chishao | MOL000358 | beta-sitosterol | 5-hydroxytryptamine 2A receptor | HTR2A |
| chishao | MOL000358 | beta-sitosterol | Progesterone receptor | PGR |
| chishao | MOL000358 | beta-sitosterol | Muscarinic acetylcholine receptor M2 | CHRM2 |
| chishao | MOL000358 | beta-sitosterol | Alpha-1B adrenergic receptor | ADRA1B |
| chishao | MOL000358 | beta-sitosterol | Beta-2 adrenergic receptor | ADRB2 |
| chishao | MOL000358 | beta-sitosterol | Neuronal acetylcholine receptor subunit alpha-2 | CHRNA2 |
| chishao | MOL000358 | beta-sitosterol | Sodium-dependent serotonin transporter | SLC6A4 |
| chishao | MOL000358 | beta-sitosterol | Mu-type opioid receptor | OPRM1 |
| chishao | MOL000358 | beta-sitosterol | Gamma-aminobutyric-acid receptor subunit alpha-1 | GABRA1 |
| chishao | MOL000358 | beta-sitosterol | Serum paraoxonase/arylesterase 1 | PON1 |
| chishao | MOL000358 | beta-sitosterol | Transcription factor AP-1 | JUN |
| chishao | MOL000358 | beta-sitosterol | Heat shock protein HSP 90-alpha | HSP90AA1 |
| chishao | MOL000358 | beta-sitosterol | Phosphatidylinositol-4,5-bisphosphate 3-kinase catalytic subunit gamma isoform | PIK3CG |
| chishao | MOL000358 | beta-sitosterol | Neuronal acetylcholine receptor subunit alpha-7 | CHRNA7 |
| chishao | MOL000358 | beta-sitosterol | Microtubule-associated protein 2 | MAP2 |
| chishao | MOL000358 | beta-sitosterol | Apoptosis regulator BAX | BAX |
| chishao | MOL000358 | beta-sitosterol | Caspase-3 | CASP3 |
| chishao | MOL000358 | beta-sitosterol | Caspase-8 | CASP8 |
| chishao | MOL000358 | beta-sitosterol | Caspase-9 | CASP9 |
| chishao | MOL000358 | beta-sitosterol | Protein kinase C alpha type | PRKCA |
| chishao | MOL000358 | beta-sitosterol | Transforming growth factor beta-1 | TGFB1 |
| chishao | MOL000359 | sitosterol | Progesterone receptor | PGR |
| chishao | MOL000359 | sitosterol | Mineralocorticoid receptor | NR3C2 |
| chishao | MOL000449 | Stigmasterol | Prostaglandin G/H synthase 1 | PTGS1 |
| chishao | MOL000449 | Stigmasterol | Muscarinic acetylcholine receptor M1 | CHRM1 |
| chishao | MOL000449 | Stigmasterol | Beta-1 adrenergic receptor | ADRB1 |
| chishao | MOL000449 | Stigmasterol | Sodium channel protein type 5 subunit alpha | SCN5A |
| chishao | MOL000449 | Stigmasterol | Prostaglandin G/H synthase 2 | PTGS2 |
| chishao | MOL000449 | Stigmasterol | Alpha-2A adrenergic receptor | ADRA2A |
| chishao | MOL000449 | Stigmasterol | Retinoic acid receptor RXR-alpha | RXRA |
| chishao | MOL000449 | Stigmasterol | 5-hydroxytryptamine 2A receptor | HTR2A |
| chishao | MOL000449 | Stigmasterol | Sodium-dependent noradrenaline transporter | SLC6A2 |
| chishao | MOL000449 | Stigmasterol | Progesterone receptor | PGR |
| chishao | MOL000449 | Stigmasterol | Muscarinic acetylcholine receptor M2 | CHRM2 |
| chishao | MOL000449 | Stigmasterol | Alpha-1B adrenergic receptor | ADRA1B |
| chishao | MOL000449 | Stigmasterol | Sodium-dependent dopamine transporter | SLC6A3 |
| chishao | MOL000449 | Stigmasterol | Mineralocorticoid receptor | NR3C2 |
| chishao | MOL000449 | Stigmasterol | Beta-2 adrenergic receptor | ADRB2 |
| chishao | MOL000449 | Stigmasterol | Aldose reductase | AKR1B1 |
| chishao | MOL000449 | Stigmasterol | Gamma-aminobutyric-acid receptor subunit alpha-1 | GABRA1 |
| chishao | MOL000449 | Stigmasterol | Urokinase-type plasminogen activator | PLAU |
| chishao | MOL000449 | Stigmasterol | Leukotriene A-4 hydrolase | LTA4H |
| chishao | MOL000449 | Stigmasterol | Amine oxidase [flavin-containing] B | MAOB |
| chishao | MOL000449 | Stigmasterol | Amine oxidase [flavin-containing] A | MAOA |
| chishao | MOL000449 | Stigmasterol | Neuronal acetylcholine receptor subunit alpha-7 | CHRNA7 |
| chishao | MOL000449 | Stigmasterol | Nuclear receptor coactivator 1 | NCOA1 |
| chishao | MOL000492 | (+)-catechin | Prostaglandin G/H synthase 1 | PTGS1 |
| chishao | MOL000492 | (+)-catechin | Estrogen receptor | ESR1 |
| chishao | MOL000492 | (+)-catechin | Prostaglandin G/H synthase 2 | PTGS2 |
| chishao | MOL000492 | (+)-catechin | Retinoic acid receptor RXR-alpha | RXRA |
| chishao | MOL000492 | (+)-catechin | Heat shock protein HSP 90-alpha | HSP90AA1 |
| chishao | MOL000492 | (+)-catechin | Calmodulin | CALM3 |
| chishao | MOL000492 | (+)-catechin | Hyaluronan synthase 2 | HAS2 |
| chishao | MOL001002 | ellagic acid | Estrogen receptor | ESR1 |
| chishao | MOL001002 | ellagic acid | Androgen receptor | AR |
| chishao | MOL001002 | ellagic acid | Progesterone receptor | PGR |
| chishao | MOL001002 | ellagic acid | 72 kDa type IV collagenase | MMP2 |
| chishao | MOL001002 | ellagic acid | Heat shock protein HSP 90-alpha | HSP90AA1 |
| chishao | MOL001002 | ellagic acid | Cell division protein kinase 2 | CDK2 |
| chishao | MOL001002 | ellagic acid | Glutathione S-transferase Mu 1 | GSTM1 |
| chishao | MOL001002 | ellagic acid | Glutathione S-transferase A2 | GSTA2 |
| chishao | MOL001002 | ellagic acid | Cyclin-dependent kinase inhibitor 1 | CDKN1A |
| chishao | MOL001002 | ellagic acid | Insulin-like growth factor II | IGF2 |
| chishao | MOL001002 | ellagic acid | Interleukin-8 | CXCL8 |
| chishao | MOL001002 | ellagic acid | Matrix metalloproteinase-9 | MMP9 |
| chishao | MOL001002 | ellagic acid | NF-kappa-B inhibitor alpha | NFKBIA |
| chishao | MOL001002 | ellagic acid | Protein kinase C beta type | PRKCB |
| chishao | MOL001002 | ellagic acid | Transcription factor p65 | RELA |
| chishao | MOL001918 | paeoniflorgenone | Gamma-aminobutyric-acid receptor subunit alpha-1 | GABRA1 |
| chishao | MOL001924 | paeoniflorin | Tumor necrosis factor | TNF |
| chishao | MOL001924 | paeoniflorin | Interleukin-6 | IL6 |
| chishao | MOL001924 | paeoniflorin | Lipopolysaccharide-binding protein | LBP |
| chishao | MOL001924 | paeoniflorin | Monocyte differentiation antigen CD14 | CD14 |
| chishao | MOL002714 | baicalein | Prostaglandin G/H synthase 1 | PTGS1 |
| chishao | MOL002714 | baicalein | Androgen receptor | AR |
| chishao | MOL002714 | baicalein | Apoptosis regulator Bcl-2 | BCL2 |
| chishao | MOL002714 | baicalein | Prostaglandin G/H synthase 2 | PTGS2 |
| chishao | MOL002714 | baicalein | cGMP-inhibited 3',5'-cyclic phosphodiesterase A | PDE3A |
| chishao | MOL002714 | baicalein | Dipeptidyl peptidase 4 | DPP4 |
| chishao | MOL002714 | baicalein | Myeloperoxidase | MPO |
| chishao | MOL002714 | baicalein | Heat shock protein HSP 90-alpha | HSP90AA1 |
| chishao | MOL002714 | baicalein | Phosphatidylinositol-4,5-bisphosphate 3-kinase catalytic subunit gamma isoform | PIK3CG |
| chishao | MOL002714 | baicalein | Cellular tumor antigen p53 | TP53 |
| chishao | MOL002714 | baicalein | Aryl hydrocarbon receptor | AHR |
| chishao | MOL002714 | baicalein | Nuclear receptor coactivator 1 | NCOA1 |
| chishao | MOL002714 | baicalein | Egl nine homolog 1 | EGLN1 |
| chishao | MOL002714 | baicalein | Cytochrome c | CYCS |
| chishao | MOL002714 | baicalein | Calmodulin | CALM3 |
| chishao | MOL002714 | baicalein | Apolipoprotein D | APOD |
| chishao | MOL002714 | baicalein | Apoptosis regulator BAX | BAX |
| chishao | MOL002714 | baicalein | Caspase-3 | CASP3 |
| chishao | MOL002714 | baicalein | Fatty acid-binding protein, epidermal | FABP5 |
| chishao | MOL002714 | baicalein | Hypoxia-inducible factor 1-alpha | HIF1A |
| chishao | MOL002714 | baicalein | Insulin-like growth factor II | IGF2 |
| chishao | MOL002714 | baicalein | Matrix metalloproteinase-9 | MMP9 |
| chishao | MOL002714 | baicalein | NADPH oxidase 5 | NOX5 |
| chishao | MOL002714 | baicalein | Nuclear factor of activated T-cells, cytoplasmic 1 | NFATC1 |
| chishao | MOL002714 | baicalein | Proto-oncogene c-Fos | FOS |
| chishao | MOL002714 | baicalein | RAC-alpha serine/threonine-protein kinase | AKT1 |
| chishao | MOL002714 | baicalein | Transcription factor p65 | RELA |
| chishao | MOL002714 | baicalein | Arachidonate 12-lipoxygenase, 12S-type | ALOX12 |
| chishao | MOL002776 | Baicalin | Coagulation factor X | F10 |
| chishao | MOL002776 | Baicalin | Tyrosine-protein phosphatase non-receptor type 1 | PTPN1 |
| chishao | MOL004355 | Spinasterol | Progesterone receptor | PGR |
| chishao | MOL004355 | Spinasterol | Mineralocorticoid receptor | NR3C2 |
| chishao | MOL005043 | campest-5-en-3beta-ol | Progesterone receptor | PGR |
| chishao | MOL006992 | (2R,3R)-4-methoxyl-distylin | Nitric oxide synthase, inducible | NOS2 |
| chishao | MOL006992 | (2R,3R)-4-methoxyl-distylin | Prostaglandin G/H synthase 1 | PTGS1 |
| chishao | MOL006992 | (2R,3R)-4-methoxyl-distylin | Estrogen receptor | ESR1 |
| chishao | MOL006992 | (2R,3R)-4-methoxyl-distylin | Prostaglandin G/H synthase 2 | PTGS2 |
| chishao | MOL006992 | (2R,3R)-4-methoxyl-distylin | Heat shock protein HSP 90-alpha | HSP90AA1 |
| chishao | MOL006999 | stigmast-7-en-3-ol | Progesterone receptor | PGR |
| dilong | MOL000953 | cholesterol | Progesterone receptor | PGR |
| dilong | MOL000953 | cholesterol | Mineralocorticoid receptor | NR3C2 |
| chuanxiong | MOL000359 | sitosterol | Progesterone receptor | PGR |
| chuanxiong | MOL000359 | sitosterol | Mineralocorticoid receptor | NR3C2 |
| chuanxiong | MOL000433 | FA | Prothrombin | F2 |
| chuanxiong | MOL000433 | FA | Glycogen synthase kinase-3 beta | GSK3B |
| chuanxiong | MOL000433 | FA | Cell division protein kinase 2 | CDK2 |
| chuanxiong | MOL001494 | Mandenol | Prostaglandin G/H synthase 1 | PTGS1 |
| chuanxiong | MOL001494 | Mandenol | Prostaglandin G/H synthase 2 | PTGS2 |
| chuanxiong | MOL002135 | Myricanone | Nitric oxide synthase, inducible | NOS2 |
| chuanxiong | MOL002135 | Myricanone | Prostaglandin G/H synthase 1 | PTGS1 |
| chuanxiong | MOL002135 | Myricanone | Prothrombin | F2 |
| chuanxiong | MOL002135 | Myricanone | Potassium voltage-gated channel subfamily H member 2 | KCNH2 |
| chuanxiong | MOL002135 | Myricanone | Estrogen receptor | ESR1 |
| chuanxiong | MOL002135 | Myricanone | Androgen receptor | AR |
| chuanxiong | MOL002135 | Myricanone | Sodium channel protein type 5 subunit alpha | SCN5A |
| chuanxiong | MOL002135 | Myricanone | Peroxisome proliferator-activated receptor gamma | PPARG |
| chuanxiong | MOL002135 | Myricanone | Prostaglandin G/H synthase 2 | PTGS2 |
| chuanxiong | MOL002135 | Myricanone | Coagulation factor VII | F7 |
| chuanxiong | MOL002135 | Myricanone | Vascular endothelial growth factor receptor 2 | KDR |
| chuanxiong | MOL002135 | Myricanone | Retinoic acid receptor RXR-alpha | RXRA |
| chuanxiong | MOL002135 | Myricanone | cGMP-inhibited 3',5'-cyclic phosphodiesterase A | PDE3A |
| chuanxiong | MOL002135 | Myricanone | Beta-2 adrenergic receptor | ADRB2 |
| chuanxiong | MOL002135 | Myricanone | Estrogen receptor beta | ESR2 |
| chuanxiong | MOL002135 | Myricanone | Dipeptidyl peptidase 4 | DPP4 |
| chuanxiong | MOL002135 | Myricanone | Mitogen-activated protein kinase 14 | MAPK14 |
| chuanxiong | MOL002135 | Myricanone | Glycogen synthase kinase-3 beta | GSK3B |
| chuanxiong | MOL002135 | Myricanone | Heat shock protein HSP 90-alpha | HSP90AA1 |
| chuanxiong | MOL002135 | Myricanone | Cell division protein kinase 2 | CDK2 |
| chuanxiong | MOL002135 | Myricanone | Serine/threonine-protein kinase Chk1 | CHEK1 |
| chuanxiong | MOL002135 | Myricanone | Cyclin-A2 | CCNA2 |
| chuanxiong | MOL002135 | Myricanone | Nuclear receptor coactivator 1 | NCOA1 |
| chuanxiong | MOL002140 | Perlolyrine | Prothrombin | F2 |
| chuanxiong | MOL002140 | Perlolyrine | Prostaglandin G/H synthase 2 | PTGS2 |
| chuanxiong | MOL002140 | Perlolyrine | Retinoic acid receptor RXR-alpha | RXRA |
| chuanxiong | MOL002157 | wallichilide | Prostaglandin G/H synthase 2 | PTGS2 |
| chuanxiong | MOL002157 | wallichilide | Mineralocorticoid receptor | NR3C2 |
| chuanxiong | MOL002157 | wallichilide | Glucocorticoid receptor | NR3C1 |
| taoren | MOL000296 | hederagenin | Prostaglandin G/H synthase 1 | PTGS1 |
| taoren | MOL000296 | hederagenin | Muscarinic acetylcholine receptor M1 | CHRM1 |
| taoren | MOL000296 | hederagenin | Sodium channel protein type 5 subunit alpha | SCN5A |
| taoren | MOL000296 | hederagenin | Prostaglandin G/H synthase 2 | PTGS2 |
| taoren | MOL000296 | hederagenin | Retinoic acid receptor RXR-alpha | RXRA |
| taoren | MOL000296 | hederagenin | cGMP-inhibited 3',5'-cyclic phosphodiesterase A | PDE3A |
| taoren | MOL000296 | hederagenin | Sodium-dependent noradrenaline transporter | SLC6A2 |
| taoren | MOL000296 | hederagenin | Progesterone receptor | PGR |
| taoren | MOL000296 | hederagenin | Muscarinic acetylcholine receptor M2 | CHRM2 |
| taoren | MOL000296 | hederagenin | Alpha-1B adrenergic receptor | ADRA1B |
| taoren | MOL000296 | hederagenin | Gamma-aminobutyric-acid receptor subunit alpha-1 | GABRA1 |
| taoren | MOL000296 | hederagenin | Lysozyme | LYZ |
| taoren | MOL000296 | hederagenin | Glutamate receptor 2 | GRIA2 |
| taoren | MOL000358 | beta-sitosterol | Prostaglandin G/H synthase 1 | PTGS1 |
| taoren | MOL000358 | beta-sitosterol | D(1A) dopamine receptor | DRD1 |
| taoren | MOL000358 | beta-sitosterol | Potassium voltage-gated channel subfamily H member 2 | KCNH2 |
| taoren | MOL000358 | beta-sitosterol | Muscarinic acetylcholine receptor M1 | CHRM1 |
| taoren | MOL000358 | beta-sitosterol | Sodium channel protein type 5 subunit alpha | SCN5A |
| taoren | MOL000358 | beta-sitosterol | Apoptosis regulator Bcl-2 | BCL2 |
| taoren | MOL000358 | beta-sitosterol | Prostaglandin G/H synthase 2 | PTGS2 |
| taoren | MOL000358 | beta-sitosterol | cGMP-inhibited 3',5'-cyclic phosphodiesterase A | PDE3A |
| taoren | MOL000358 | beta-sitosterol | 5-hydroxytryptamine 2A receptor | HTR2A |
| taoren | MOL000358 | beta-sitosterol | Progesterone receptor | PGR |
| taoren | MOL000358 | beta-sitosterol | Muscarinic acetylcholine receptor M2 | CHRM2 |
| taoren | MOL000358 | beta-sitosterol | Alpha-1B adrenergic receptor | ADRA1B |
| taoren | MOL000358 | beta-sitosterol | Beta-2 adrenergic receptor | ADRB2 |
| taoren | MOL000358 | beta-sitosterol | Neuronal acetylcholine receptor subunit alpha-2 | CHRNA2 |
| taoren | MOL000358 | beta-sitosterol | Sodium-dependent serotonin transporter | SLC6A4 |
| taoren | MOL000358 | beta-sitosterol | Mu-type opioid receptor | OPRM1 |
| taoren | MOL000358 | beta-sitosterol | Gamma-aminobutyric-acid receptor subunit alpha-1 | GABRA1 |
| taoren | MOL000358 | beta-sitosterol | Serum paraoxonase/arylesterase 1 | PON1 |
| taoren | MOL000358 | beta-sitosterol | Transcription factor AP-1 | JUN |
| taoren | MOL000358 | beta-sitosterol | Heat shock protein HSP 90-alpha | HSP90AA1 |
| taoren | MOL000358 | beta-sitosterol | Phosphatidylinositol-4,5-bisphosphate 3-kinase catalytic subunit gamma isoform | PIK3CG |
| taoren | MOL000358 | beta-sitosterol | Neuronal acetylcholine receptor subunit alpha-7 | CHRNA7 |
| taoren | MOL000358 | beta-sitosterol | Microtubule-associated protein 2 | MAP2 |
| taoren | MOL000358 | beta-sitosterol | Apoptosis regulator BAX | BAX |
| taoren | MOL000358 | beta-sitosterol | Caspase-3 | CASP3 |
| taoren | MOL000358 | beta-sitosterol | Caspase-8 | CASP8 |
| taoren | MOL000358 | beta-sitosterol | Caspase-9 | CASP9 |
| taoren | MOL000358 | beta-sitosterol | Protein kinase C alpha type | PRKCA |
| taoren | MOL000358 | beta-sitosterol | Transforming growth factor beta-1 | TGFB1 |
| taoren | MOL000493 | campesterol | Prostaglandin G/H synthase 1 | PTGS1 |
| taoren | MOL000493 | campesterol | Prostaglandin G/H synthase 2 | PTGS2 |
| taoren | MOL000493 | campesterol | Progesterone receptor | PGR |
| taoren | MOL000493 | campesterol | Heat shock protein HSP 90-alpha | HSP90AA1 |
| taoren | MOL000493 | campesterol | Phosphatidylinositol-4,5-bisphosphate 3-kinase catalytic subunit gamma isoform | PIK3CG |
| taoren | MOL001323 | Sitosterol alpha1 | Prostaglandin G/H synthase 2 | PTGS2 |
| taoren | MOL001323 | Sitosterol alpha1 | Progesterone receptor | PGR |
| taoren | MOL001323 | Sitosterol alpha1 | Mineralocorticoid receptor | NR3C2 |
| taoren | MOL001323 | Sitosterol alpha1 | Gamma-aminobutyric-acid receptor subunit alpha-1 | GABRA1 |
| taoren | MOL001328 | 2,3-didehydro GA70 | Prostaglandin G/H synthase 1 | PTGS1 |
| taoren | MOL001328 | 2,3-didehydro GA70 | Muscarinic acetylcholine receptor M1 | CHRM1 |
| taoren | MOL001328 | 2,3-didehydro GA70 | Prostaglandin G/H synthase 2 | PTGS2 |
| taoren | MOL001328 | 2,3-didehydro GA70 | Sodium-dependent noradrenaline transporter | SLC6A2 |
| taoren | MOL001328 | 2,3-didehydro GA70 | Gamma-aminobutyric-acid receptor subunit alpha-1 | GABRA1 |
| taoren | MOL001328 | 2,3-didehydro GA70 | Glutamate receptor 2 | GRIA2 |
| taoren | MOL001329 | 2,3-didehydro GA77 | Prostaglandin G/H synthase 2 | PTGS2 |
| taoren | MOL001329 | 2,3-didehydro GA77 | Carbonic anhydrase 2 | CA2 |
| taoren | MOL001329 | 2,3-didehydro GA77 | Gamma-aminobutyric-acid receptor subunit alpha-1 | GABRA1 |
| taoren | MOL001340 | GA120 | Muscarinic acetylcholine receptor M1 | CHRM1 |
| taoren | MOL001340 | GA120 | Prostaglandin G/H synthase 2 | PTGS2 |
| taoren | MOL001340 | GA120 | Muscarinic acetylcholine receptor M2 | CHRM2 |
| taoren | MOL001340 | GA120 | Gamma-aminobutyric-acid receptor subunit alpha-1 | GABRA1 |
| taoren | MOL001340 | GA120 | Neuronal acetylcholine receptor subunit alpha-7 | CHRNA7 |
| taoren | MOL001342 | GA121-isolactone | Progesterone receptor | PGR |
| taoren | MOL001344 | GA122-isolactone | Progesterone receptor | PGR |
| taoren | MOL001349 | 4a-formyl-7alpha-hydroxy-1-methyl-8-methylidene-4aalpha,4bbeta-gibbane-1alpha,10beta-dicarboxylic acid | Progesterone receptor | PGR |
| taoren | MOL001349 | 4a-formyl-7alpha-hydroxy-1-methyl-8-methylidene-4aalpha,4bbeta-gibbane-1alpha,10beta-dicarboxylic acid | Mineralocorticoid receptor | NR3C2 |
| taoren | MOL001351 | Gibberellin A44 | Mineralocorticoid receptor | NR3C2 |
| taoren | MOL001351 | Gibberellin A44 | Gamma-aminobutyric-acid receptor subunit alpha-1 | GABRA1 |
| taoren | MOL001352 | GA54 | Coagulation factor X | F10 |
| taoren | MOL001352 | GA54 | Prostaglandin G/H synthase 2 | PTGS2 |
| taoren | MOL001352 | GA54 | Tyrosine-protein phosphatase non-receptor type 1 | PTPN1 |
| taoren | MOL001352 | GA54 | Heat shock protein HSP 90-alpha | HSP90AA1 |
| taoren | MOL001352 | GA54 | Calmodulin | CALM3 |
| taoren | MOL001353 | GA60 | Muscarinic acetylcholine receptor M2 | CHRM2 |
| taoren | MOL001353 | GA60 | Gamma-aminobutyric-acid receptor subunit alpha-1 | GABRA1 |
| taoren | MOL001353 | GA60 | Glutamate receptor 2 | GRIA2 |
| taoren | MOL001355 | GA63 | Prostaglandin G/H synthase 2 | PTGS2 |
| taoren | MOL001355 | GA63 | Gamma-aminobutyric-acid receptor subunit alpha-1 | GABRA1 |
| taoren | MOL001355 | GA63 | Neuronal acetylcholine receptor subunit alpha-7 | CHRNA7 |
| taoren | MOL001355 | GA63 | Glutamate receptor 2 | GRIA2 |
| taoren | MOL001358 | gibberellin 7 | Muscarinic acetylcholine receptor M1 | CHRM1 |
| taoren | MOL001358 | gibberellin 7 | Prostaglandin G/H synthase 2 | PTGS2 |
| taoren | MOL001358 | gibberellin 7 | cGMP-inhibited 3',5'-cyclic phosphodiesterase A | PDE3A |
| taoren | MOL001358 | gibberellin 7 | Sodium-dependent dopamine transporter | SLC6A3 |
| taoren | MOL001358 | gibberellin 7 | Beta-2 adrenergic receptor | ADRB2 |
| taoren | MOL001358 | gibberellin 7 | Sodium-dependent serotonin transporter | SLC6A4 |
| taoren | MOL001360 | GA77 | Gamma-aminobutyric-acid receptor subunit alpha-1 | GABRA1 |
| taoren | MOL001360 | GA77 | Glutamate receptor 2 | GRIA2 |
| taoren | MOL001361 | GA87 | Prostaglandin G/H synthase 2 | PTGS2 |
| taoren | MOL001361 | GA87 | Carbonic anhydrase 2 | CA2 |
| taoren | MOL001368 | 3-O-p-coumaroylquinic acid | Prostaglandin G/H synthase 1 | PTGS1 |
| taoren | MOL001368 | 3-O-p-coumaroylquinic acid | Prostaglandin G/H synthase 2 | PTGS2 |
| taoren | MOL001368 | 3-O-p-coumaroylquinic acid | Tyrosine-protein phosphatase non-receptor type 1 | PTPN1 |
| taoren | MOL001368 | 3-O-p-coumaroylquinic acid | Heat shock protein HSP 90-alpha | HSP90AA1 |
| taoren | MOL001368 | 3-O-p-coumaroylquinic acid | Phosphatidylinositol-4,5-bisphosphate 3-kinase catalytic subunit gamma isoform | PIK3CG |
| taoren | MOL001368 | 3-O-p-coumaroylquinic acid | Calmodulin | CALM3 |
| honghua | MOL000006 | luteolin | Prostaglandin G/H synthase 1 | PTGS1 |
| honghua | MOL000006 | luteolin | Insulin receptor | INSR |
| honghua | MOL000006 | luteolin | Androgen receptor | AR |
| honghua | MOL000006 | luteolin | Prostaglandin G/H synthase 2 | PTGS2 |
| honghua | MOL000006 | luteolin | 72 kDa type IV collagenase | MMP2 |
| honghua | MOL000006 | luteolin | Tumor necrosis factor | TNF |
| honghua | MOL000006 | luteolin | Epidermal growth factor receptor | EGFR |
| honghua | MOL000006 | luteolin | Dipeptidyl peptidase 4 | DPP4 |
| honghua | MOL000006 | luteolin | Hepatocyte growth factor receptor | MET |
| honghua | MOL000006 | luteolin | Interleukin-6 | IL6 |
| honghua | MOL000006 | luteolin | Interstitial collagenase | MMP1 |
| honghua | MOL000006 | luteolin | Mitogen-activated protein kinase 1 | MAPK1 |
| honghua | MOL000006 | luteolin | Interferon gamma | IFNG |
| honghua | MOL000006 | luteolin | Transcription factor AP-1 | JUN |
| honghua | MOL000006 | luteolin | Heat shock protein HSP 90-alpha | HSP90AA1 |
| honghua | MOL000006 | luteolin | Phosphatidylinositol-4,5-bisphosphate 3-kinase catalytic subunit gamma isoform | PIK3CG |
| honghua | MOL000006 | luteolin | Xanthine dehydrogenase/oxidase | XDH |
| honghua | MOL000006 | luteolin | Cellular tumor antigen p53 | TP53 |
| honghua | MOL000006 | luteolin | Amyloid beta A4 protein | APP |
| honghua | MOL000006 | luteolin | DNA topoisomerase 1 | TOP1 |
| honghua | MOL000006 | luteolin | Heme oxygenase 1 | HMOX1 |
| honghua | MOL000006 | luteolin | Retinoblastoma-associated protein | RB1 |
| honghua | MOL000006 | luteolin | Caspase-7 | CASP7 |
| honghua | MOL000006 | luteolin | Interleukin-2 | IL2 |
| honghua | MOL000006 | luteolin | Baculoviral IAP repeat-containing protein 4 | XIAP |
| honghua | MOL000006 | luteolin | Baculoviral IAP repeat-containing protein 5 | BIRC5 |
| honghua | MOL000006 | luteolin | Bcl-2-like protein 1 | BCL2L1 |
| honghua | MOL000006 | luteolin | Caspase-3 | CASP3 |
| honghua | MOL000006 | luteolin | Caspase-9 | CASP9 |
| honghua | MOL000006 | luteolin | CD40 ligand | CD40LG |
| honghua | MOL000006 | luteolin | Cyclin-dependent kinase inhibitor 1 | CDKN1A |
| honghua | MOL000006 | luteolin | E3 ubiquitin-protein ligase Mdm2 | MDM2 |
| honghua | MOL000006 | luteolin | G1/S-specific cyclin-D1 | CCND1 |
| honghua | MOL000006 | luteolin | Intercellular adhesion molecule 1 | ICAM1 |
| honghua | MOL000006 | luteolin | Interleukin-10 | IL10 |
| honghua | MOL000006 | luteolin | Interleukin-4 | IL4 |
| honghua | MOL000006 | luteolin | Matrix metalloproteinase-9 | MMP9 |
| honghua | MOL000006 | luteolin | NF-kappa-B inhibitor alpha | NFKBIA |
| honghua | MOL000006 | luteolin | Peroxisome proliferator-activated receptor gamma | PPARG |
| honghua | MOL000006 | luteolin | Proliferating cell nuclear antigen | PCNA |
| honghua | MOL000006 | luteolin | Prostaglandin E synthase | PTGES |
| honghua | MOL000006 | luteolin | RAC-alpha serine/threonine-protein kinase | AKT1 |
| honghua | MOL000006 | luteolin | Receptor tyrosine-protein kinase erbB-2 | ERBB2 |
| honghua | MOL000006 | luteolin | Solute carrier family 2, facilitated glucose transporter member 4 | SLC2A4 |
| honghua | MOL000006 | luteolin | Transcription factor p65 | RELA |
| honghua | MOL000098 | quercetin | Prostaglandin G/H synthase 1 | PTGS1 |
| honghua | MOL000098 | quercetin | Insulin receptor | INSR |
| honghua | MOL000098 | quercetin | Prothrombin | F2 |
| honghua | MOL000098 | quercetin | Potassium voltage-gated channel subfamily H member 2 | KCNH2 |
| honghua | MOL000098 | quercetin | Androgen receptor | AR |
| honghua | MOL000098 | quercetin | Sodium channel protein type 5 subunit alpha | SCN5A |
| honghua | MOL000098 | quercetin | Peroxisome proliferator-activated receptor gamma | PPARG |
| honghua | MOL000098 | quercetin | Coagulation factor X | F10 |
| honghua | MOL000098 | quercetin | Apoptosis regulator Bcl-2 | BCL2 |
| honghua | MOL000098 | quercetin | Arachidonate 5-lipoxygenase | ALOX5 |
| honghua | MOL000098 | quercetin | Prostaglandin G/H synthase 2 | PTGS2 |
| honghua | MOL000098 | quercetin | Nitric-oxide synthase, endothelial | NOS3 |
| honghua | MOL000098 | quercetin | Coagulation factor VII | F7 |
| honghua | MOL000098 | quercetin | Ornithine decarboxylase | ODC1 |
| honghua | MOL000098 | quercetin | Retinoic acid receptor RXR-alpha | RXRA |
| honghua | MOL000098 | quercetin | Acetylcholinesterase | ACHE |
| honghua | MOL000098 | quercetin | Acetyl-CoA carboxylase 1 | ACACA |
| honghua | MOL000098 | quercetin | 72 kDa type IV collagenase | MMP2 |
| honghua | MOL000098 | quercetin | Beta-2 adrenergic receptor | ADRB2 |
| honghua | MOL000098 | quercetin | Tumor necrosis factor | TNF |
| honghua | MOL000098 | quercetin | Aldose reductase | AKR1B1 |
| honghua | MOL000098 | quercetin | Epidermal growth factor receptor | EGFR |
| honghua | MOL000098 | quercetin | Gamma-aminobutyric-acid receptor subunit alpha-1 | GABRA1 |
| honghua | MOL000098 | quercetin | Maltase-glucoamylase, intestinal | MGAM |
| honghua | MOL000098 | quercetin | Dipeptidyl peptidase 4 | DPP4 |
| honghua | MOL000098 | quercetin | Urokinase-type plasminogen activator | PLAU |
| honghua | MOL000098 | quercetin | Interleukin-6 | IL6 |
| honghua | MOL000098 | quercetin | Interstitial collagenase | MMP1 |
| honghua | MOL000098 | quercetin | Mitogen-activated protein kinase 1 | MAPK1 |
| honghua | MOL000098 | quercetin | Serum paraoxonase/arylesterase 1 | PON1 |
| honghua | MOL000098 | quercetin | Cathepsin D | CTSD |
| honghua | MOL000098 | quercetin | Interferon gamma | IFNG |
| honghua | MOL000098 | quercetin | Transcription factor AP-1 | JUN |
| honghua | MOL000098 | quercetin | Small inducible cytokine A2 | CCL2 |
| honghua | MOL000098 | quercetin | Interleukin-1 beta | IL1B |
| honghua | MOL000098 | quercetin | E-selectin | SELE |
| honghua | MOL000098 | quercetin | Myeloperoxidase | MPO |
| honghua | MOL000098 | quercetin | Tissue-type plasminogen activator | PLAT |
| honghua | MOL000098 | quercetin | Gap junction alpha-1 protein | GJA1 |
| honghua | MOL000098 | quercetin | Vascular cell adhesion protein 1 | VCAM1 |
| honghua | MOL000098 | quercetin | Stromelysin-1 | MMP3 |
| honghua | MOL000098 | quercetin | Heat shock protein HSP 90-alpha | HSP90AA1 |
| honghua | MOL000098 | quercetin | Thrombomodulin | THBD |
| honghua | MOL000098 | quercetin | Tissue factor | F3 |
| honghua | MOL000098 | quercetin | NAD(P)H dehydrogenase [quinone] 1 | NQO1 |
| honghua | MOL000098 | quercetin | Phosphatidylinositol-4,5-bisphosphate 3-kinase catalytic subunit gamma isoform | PIK3CG |
| honghua | MOL000098 | quercetin | Amine oxidase [flavin-containing] B | MAOB |
| honghua | MOL000098 | quercetin | Xanthine dehydrogenase/oxidase | XDH |
| honghua | MOL000098 | quercetin | Prostaglandin E2 receptor, EP3 subtype | PTGER3 |
| honghua | MOL000098 | quercetin | Superoxide dismutase [Cu-Zn] | SOD1 |
| honghua | MOL000098 | quercetin | Cytochrome P450 3A4 | CYP3A4 |
| honghua | MOL000098 | quercetin | Cellular tumor antigen p53 | TP53 |
| honghua | MOL000098 | quercetin | DNA topoisomerase 1 | TOP1 |
| honghua | MOL000098 | quercetin | Heme oxygenase 1 | HMOX1 |
| honghua | MOL000098 | quercetin | Collagen alpha-1(III) chain | COL3A1 |
| honghua | MOL000098 | quercetin | Retinoblastoma-associated protein | RB1 |
| honghua | MOL000098 | quercetin | Glutathione S-transferase Mu 1 | GSTM1 |
| honghua | MOL000098 | quercetin | 78 kDa glucose-regulated protein | HSPA5 |
| honghua | MOL000098 | quercetin | Aryl hydrocarbon receptor | AHR |
| honghua | MOL000098 | quercetin | Interleukin-2 | IL2 |
| honghua | MOL000098 | quercetin | Activator of 90 kDa heat shock protein ATPase homolog 1 | AHSA1 |
| honghua | MOL000098 | quercetin | Apoptosis regulator BAX | BAX |
| honghua | MOL000098 | quercetin | ATP-binding cassette sub-family G member 2 | ABCG2 |
| honghua | MOL000098 | quercetin | Baculoviral IAP repeat-containing protein 5 | BIRC5 |
| honghua | MOL000098 | quercetin | Bcl-2-like protein 1 | BCL2L1 |
| honghua | MOL000098 | quercetin | Caspase-3 | CASP3 |
| honghua | MOL000098 | quercetin | Caspase-8 | CASP8 |
| honghua | MOL000098 | quercetin | Caspase-9 | CASP9 |
| honghua | MOL000098 | quercetin | Caveolin-1 | CAV1 |
| honghua | MOL000098 | quercetin | CD40 ligand | CD40LG |
| honghua | MOL000098 | quercetin | C-reactive protein | CRP |
| honghua | MOL000098 | quercetin | C-X-C motif chemokine 10 | CXCL10 |
| honghua | MOL000098 | quercetin | C-X-C motif chemokine 11 | CXCL11 |
| honghua | MOL000098 | quercetin | C-X-C motif chemokine 2 | CXCL2 |
| honghua | MOL000098 | quercetin | Cyclin-dependent kinase inhibitor 1 | CDKN1A |
| honghua | MOL000098 | quercetin | Cyclin-dependent kinase inhibitor 2A, isoforms 1/2/3 | CDKN2A |
| honghua | MOL000098 | quercetin | Cytochrome P450 1A1 | CYP1A1 |
| honghua | MOL000098 | quercetin | Cytochrome P450 1B1 | CYP1B1 |
| honghua | MOL000098 | quercetin | DDB1- and CUL4-associated factor 5 | DCAF5 |
| honghua | MOL000098 | quercetin | Dual oxidase 2 | DUOX2 |
| honghua | MOL000098 | quercetin | ETS domain-containing protein Elk-1 | ELK1 |
| honghua | MOL000098 | quercetin | G1/S-specific cyclin-D1 | CCND1 |
| honghua | MOL000098 | quercetin | Heat shock factor protein 1 | HSF1 |
| honghua | MOL000098 | quercetin | Heat shock protein beta-1 | HSPB1 |
| honghua | MOL000098 | quercetin | Hexokinase-2 | HK2 |
| honghua | MOL000098 | quercetin | Hyaluronan synthase 2 | HAS2 |
| honghua | MOL000098 | quercetin | Hypoxia-inducible factor 1-alpha | HIF1A |
| honghua | MOL000098 | quercetin | Inhibitor of nuclear factor kappa-B kinase subunit alpha | CHUK |
| honghua | MOL000098 | quercetin | Insulin-like growth factor-binding protein 3 | IGFBP3 |
| honghua | MOL000098 | quercetin | Insulin-like growth factor II | IGF2 |
| honghua | MOL000098 | quercetin | Intercellular adhesion molecule 1 | ICAM1 |
| honghua | MOL000098 | quercetin | Interferon regulatory factor 1 | IRF1 |
| honghua | MOL000098 | quercetin | Interleukin-10 | IL10 |
| honghua | MOL000098 | quercetin | Interleukin-1 alpha | IL1A |
| honghua | MOL000098 | quercetin | Interleukin-8 | CXCL8 |
| honghua | MOL000098 | quercetin | Matrix metalloproteinase-9 | MMP9 |
| honghua | MOL000098 | quercetin | Myc proto-oncogene protein | MYC |
| honghua | MOL000098 | quercetin | Neutrophil cytosol factor 1 | NCF1 |
| honghua | MOL000098 | quercetin | NF-kappa-B inhibitor alpha | NFKBIA |
| honghua | MOL000098 | quercetin | Nuclear factor erythroid 2-related factor 2 | NFE2L2 |
| honghua | MOL000098 | quercetin | Nuclear receptor subfamily 1 group I member 2 | NR1I2 |
| honghua | MOL000098 | quercetin | Osteopontin | SPP1 |
| honghua | MOL000098 | quercetin | Peroxisome proliferator-activated receptor alpha | PPARA |
| honghua | MOL000098 | quercetin | Peroxisome proliferator-activated receptor delta | PPARD |
| honghua | MOL000098 | quercetin | Peroxisome proliferator-activated receptor gamma | PPARG |
| honghua | MOL000098 | quercetin | Plasminogen activator inhibitor 1 | SERPINE1 |
| honghua | MOL000098 | quercetin | Poly [ADP-ribose] polymerase 1 | PARP1 |
| honghua | MOL000098 | quercetin | Procollagen C-endopeptidase enhancer 1 | PCOLCE |
| honghua | MOL000098 | quercetin | Protein kinase C alpha type | PRKCA |
| honghua | MOL000098 | quercetin | Protein kinase C beta type | PRKCB |
| honghua | MOL000098 | quercetin | Proto-oncogene c-Fos | FOS |
| honghua | MOL000098 | quercetin | RAC-alpha serine/threonine-protein kinase | AKT1 |
| honghua | MOL000098 | quercetin | RAF proto-oncogene serine/threonine-protein kinase | RAF1 |
| honghua | MOL000098 | quercetin | Ras GTPase-activating protein 1 | RASA1 |
| honghua | MOL000098 | quercetin | Receptor tyrosine-protein kinase erbB-2 | ERBB2 |
| honghua | MOL000098 | quercetin | Runt-related transcription factor 2 | RUNX2 |
| honghua | MOL000098 | quercetin | Serine/threonine-protein kinase Chk2 | CHEK2 |
| honghua | MOL000098 | quercetin | Signal transducer and activator of transcription 1-alpha/beta | STAT1 |
| honghua | MOL000098 | quercetin | Solute carrier family 2, facilitated glucose transporter member 4 | SLC2A4 |
| honghua | MOL000098 | quercetin | Transcription factor E2F1 | E2F1 |
| honghua | MOL000098 | quercetin | Transcription factor p65 | RELA |
| honghua | MOL000098 | quercetin | Transforming growth factor beta-1 | TGFB1 |
| honghua | MOL000098 | quercetin | Type I iodothyronine deiodinase | DIO1 |
| honghua | MOL000098 | quercetin | Phosphatidylinositol-3,4,5-trisphosphate 3-phosphatase and dual-specificity protein phosphatase PTEN | PTEN |
| honghua | MOL000358 | beta-sitosterol | Prostaglandin G/H synthase 1 | PTGS1 |
| honghua | MOL000358 | beta-sitosterol | D(1A) dopamine receptor | DRD1 |
| honghua | MOL000358 | beta-sitosterol | Potassium voltage-gated channel subfamily H member 2 | KCNH2 |
| honghua | MOL000358 | beta-sitosterol | Muscarinic acetylcholine receptor M1 | CHRM1 |
| honghua | MOL000358 | beta-sitosterol | Sodium channel protein type 5 subunit alpha | SCN5A |
| honghua | MOL000358 | beta-sitosterol | Apoptosis regulator Bcl-2 | BCL2 |
| honghua | MOL000358 | beta-sitosterol | Prostaglandin G/H synthase 2 | PTGS2 |
| honghua | MOL000358 | beta-sitosterol | cGMP-inhibited 3',5'-cyclic phosphodiesterase A | PDE3A |
| honghua | MOL000358 | beta-sitosterol | 5-hydroxytryptamine 2A receptor | HTR2A |
| honghua | MOL000358 | beta-sitosterol | Progesterone receptor | PGR |
| honghua | MOL000358 | beta-sitosterol | Muscarinic acetylcholine receptor M2 | CHRM2 |
| honghua | MOL000358 | beta-sitosterol | Alpha-1B adrenergic receptor | ADRA1B |
| honghua | MOL000358 | beta-sitosterol | Beta-2 adrenergic receptor | ADRB2 |
| honghua | MOL000358 | beta-sitosterol | Neuronal acetylcholine receptor subunit alpha-2 | CHRNA2 |
| honghua | MOL000358 | beta-sitosterol | Sodium-dependent serotonin transporter | SLC6A4 |
| honghua | MOL000358 | beta-sitosterol | Mu-type opioid receptor | OPRM1 |
| honghua | MOL000358 | beta-sitosterol | Gamma-aminobutyric-acid receptor subunit alpha-1 | GABRA1 |
| honghua | MOL000358 | beta-sitosterol | Serum paraoxonase/arylesterase 1 | PON1 |
| honghua | MOL000358 | beta-sitosterol | Transcription factor AP-1 | JUN |
| honghua | MOL000358 | beta-sitosterol | Heat shock protein HSP 90-alpha | HSP90AA1 |
| honghua | MOL000358 | beta-sitosterol | Phosphatidylinositol-4,5-bisphosphate 3-kinase catalytic subunit gamma isoform | PIK3CG |
| honghua | MOL000358 | beta-sitosterol | Neuronal acetylcholine receptor subunit alpha-7 | CHRNA7 |
| honghua | MOL000358 | beta-sitosterol | Microtubule-associated protein 2 | MAP2 |
| honghua | MOL000358 | beta-sitosterol | Apoptosis regulator BAX | BAX |
| honghua | MOL000358 | beta-sitosterol | Caspase-3 | CASP3 |
| honghua | MOL000358 | beta-sitosterol | Caspase-8 | CASP8 |
| honghua | MOL000358 | beta-sitosterol | Caspase-9 | CASP9 |
| honghua | MOL000358 | beta-sitosterol | Protein kinase C alpha type | PRKCA |
| honghua | MOL000358 | beta-sitosterol | Transforming growth factor beta-1 | TGFB1 |
| honghua | MOL000422 | kaempferol | Nitric oxide synthase, inducible | NOS2 |
| honghua | MOL000422 | kaempferol | Prostaglandin G/H synthase 1 | PTGS1 |
| honghua | MOL000422 | kaempferol | Insulin receptor | INSR |
| honghua | MOL000422 | kaempferol | Prothrombin | F2 |
| honghua | MOL000422 | kaempferol | Muscarinic acetylcholine receptor M1 | CHRM1 |
| honghua | MOL000422 | kaempferol | Androgen receptor | AR |
| honghua | MOL000422 | kaempferol | Peroxisome proliferator-activated receptor gamma | PPARG |
| honghua | MOL000422 | kaempferol | Apoptosis regulator Bcl-2 | BCL2 |
| honghua | MOL000422 | kaempferol | Arachidonate 5-lipoxygenase | ALOX5 |
| honghua | MOL000422 | kaempferol | Prostaglandin G/H synthase 2 | PTGS2 |
| honghua | MOL000422 | kaempferol | Nitric-oxide synthase, endothelial | NOS3 |
| honghua | MOL000422 | kaempferol | Coagulation factor VII | F7 |
| honghua | MOL000422 | kaempferol | Acetylcholinesterase | ACHE |
| honghua | MOL000422 | kaempferol | Sodium-dependent noradrenaline transporter | SLC6A2 |
| honghua | MOL000422 | kaempferol | Progesterone receptor | PGR |
| honghua | MOL000422 | kaempferol | Muscarinic acetylcholine receptor M2 | CHRM2 |
| honghua | MOL000422 | kaempferol | Alpha-1B adrenergic receptor | ADRA1B |
| honghua | MOL000422 | kaempferol | Tumor necrosis factor | TNF |
| honghua | MOL000422 | kaempferol | Gamma-aminobutyric-acid receptor subunit alpha-1 | GABRA1 |
| honghua | MOL000422 | kaempferol | Dipeptidyl peptidase 4 | DPP4 |
| honghua | MOL000422 | kaempferol | Interstitial collagenase | MMP1 |
| honghua | MOL000422 | kaempferol | Transcription factor AP-1 | JUN |
| honghua | MOL000422 | kaempferol | E-selectin | SELE |
| honghua | MOL000422 | kaempferol | Vascular cell adhesion protein 1 | VCAM1 |
| honghua | MOL000422 | kaempferol | Heat shock protein HSP 90-alpha | HSP90AA1 |
| honghua | MOL000422 | kaempferol | Phosphatidylinositol-4,5-bisphosphate 3-kinase catalytic subunit gamma isoform | PIK3CG |
| honghua | MOL000422 | kaempferol | Xanthine dehydrogenase/oxidase | XDH |
| honghua | MOL000422 | kaempferol | Cytochrome P450 3A4 | CYP3A4 |
| honghua | MOL000422 | kaempferol | Mitogen-activated protein kinase 8 | MAPK8 |
| honghua | MOL000422 | kaempferol | Heme oxygenase 1 | HMOX1 |
| honghua | MOL000422 | kaempferol | Glutathione S-transferase Mu 1 | GSTM1 |
| honghua | MOL000422 | kaempferol | Aryl hydrocarbon receptor | AHR |
| honghua | MOL000422 | kaempferol | Calmodulin | CALM3 |
| honghua | MOL000422 | kaempferol | Serine/threonine-protein phosphatase 2B catalytic subunit alpha isoform | PPP3CA |
| honghua | MOL000422 | kaempferol | Activator of 90 kDa heat shock protein ATPase homolog 1 | AHSA1 |
| honghua | MOL000422 | kaempferol | Antileukoproteinase | SLPI |
| honghua | MOL000422 | kaempferol | Apoptosis regulator BAX | BAX |
| honghua | MOL000422 | kaempferol | Caspase-3 | CASP3 |
| honghua | MOL000422 | kaempferol | Cytochrome P450 1A1 | CYP1A1 |
| honghua | MOL000422 | kaempferol | Cytochrome P450 1B1 | CYP1B1 |
| honghua | MOL000422 | kaempferol | Hyaluronan synthase 2 | HAS2 |
| honghua | MOL000422 | kaempferol | Inhibitor of nuclear factor kappa-B kinase subunit beta | IKBKB |
| honghua | MOL000422 | kaempferol | Intercellular adhesion molecule 1 | ICAM1 |
| honghua | MOL000422 | kaempferol | Nuclear receptor subfamily 1 group I member 2 | NR1I2 |
| honghua | MOL000422 | kaempferol | Peroxisome proliferator-activated receptor gamma | PPARG |
| honghua | MOL000422 | kaempferol | RAC-alpha serine/threonine-protein kinase | AKT1 |
| honghua | MOL000422 | kaempferol | Signal transducer and activator of transcription 1-alpha/beta | STAT1 |
| honghua | MOL000422 | kaempferol | Solute carrier family 2, facilitated glucose transporter member 4 | SLC2A4 |
| honghua | MOL000422 | kaempferol | Transcription factor p65 | RELA |
| honghua | MOL000422 | kaempferol | Type I iodothyronine deiodinase | DIO1 |
| honghua | MOL000449 | Stigmasterol | Prostaglandin G/H synthase 1 | PTGS1 |
| honghua | MOL000449 | Stigmasterol | Muscarinic acetylcholine receptor M1 | CHRM1 |
| honghua | MOL000449 | Stigmasterol | Beta-1 adrenergic receptor | ADRB1 |
| honghua | MOL000449 | Stigmasterol | Sodium channel protein type 5 subunit alpha | SCN5A |
| honghua | MOL000449 | Stigmasterol | Prostaglandin G/H synthase 2 | PTGS2 |
| honghua | MOL000449 | Stigmasterol | Alpha-2A adrenergic receptor | ADRA2A |
| honghua | MOL000449 | Stigmasterol | Retinoic acid receptor RXR-alpha | RXRA |
| honghua | MOL000449 | Stigmasterol | 5-hydroxytryptamine 2A receptor | HTR2A |
| honghua | MOL000449 | Stigmasterol | Sodium-dependent noradrenaline transporter | SLC6A2 |
| honghua | MOL000449 | Stigmasterol | Progesterone receptor | PGR |
| honghua | MOL000449 | Stigmasterol | Muscarinic acetylcholine receptor M2 | CHRM2 |
| honghua | MOL000449 | Stigmasterol | Alpha-1B adrenergic receptor | ADRA1B |
| honghua | MOL000449 | Stigmasterol | Sodium-dependent dopamine transporter | SLC6A3 |
| honghua | MOL000449 | Stigmasterol | Mineralocorticoid receptor | NR3C2 |
| honghua | MOL000449 | Stigmasterol | Beta-2 adrenergic receptor | ADRB2 |
| honghua | MOL000449 | Stigmasterol | Aldose reductase | AKR1B1 |
| honghua | MOL000449 | Stigmasterol | Gamma-aminobutyric-acid receptor subunit alpha-1 | GABRA1 |
| honghua | MOL000449 | Stigmasterol | Urokinase-type plasminogen activator | PLAU |
| honghua | MOL000449 | Stigmasterol | Leukotriene A-4 hydrolase | LTA4H |
| honghua | MOL000449 | Stigmasterol | Amine oxidase [flavin-containing] B | MAOB |
| honghua | MOL000449 | Stigmasterol | Amine oxidase [flavin-containing] A | MAOA |
| honghua | MOL000449 | Stigmasterol | Neuronal acetylcholine receptor subunit alpha-7 | CHRNA7 |
| honghua | MOL000449 | Stigmasterol | Nuclear receptor coactivator 1 | NCOA1 |
| honghua | MOL000953 | CLR | Progesterone receptor | PGR |
| honghua | MOL000953 | CLR | Mineralocorticoid receptor | NR3C2 |
| honghua | MOL001771 | poriferast-5-en-3beta-ol | Progesterone receptor | PGR |
| honghua | MOL002694 | 4-[(E)-4-(3,5-dimethoxy-4-oxo-1-cyclohexa-2,5-dienylidene)but-2-enylidene]-2,6-dimethoxycyclohexa-2,5-dien-1-one | Prothrombin | F2 |
| honghua | MOL002694 | 4-[(E)-4-(3,5-dimethoxy-4-oxo-1-cyclohexa-2,5-dienylidene)but-2-enylidene]-2,6-dimethoxycyclohexa-2,5-dien-1-one | Prostaglandin G/H synthase 2 | PTGS2 |
| honghua | MOL002695 | lignan | Estrogen receptor | ESR1 |
| honghua | MOL002695 | lignan | Coagulation factor X | F10 |
| honghua | MOL002695 | lignan | Prostaglandin G/H synthase 2 | PTGS2 |
| honghua | MOL002695 | lignan | Carbonic anhydrase 2 | CA2 |
| honghua | MOL002695 | lignan | Calcium-activated potassium channel subunit alpha 1 | KCNMA1 |
| honghua | MOL002695 | lignan | Calmodulin | CALM3 |
| honghua | MOL002710 | Pyrethrin II | Prostaglandin G/H synthase 2 | PTGS2 |
| honghua | MOL002712 | 6-Hydroxykaempferol | Nitric oxide synthase, inducible | NOS2 |
| honghua | MOL002712 | 6-Hydroxykaempferol | Prostaglandin G/H synthase 1 | PTGS1 |
| honghua | MOL002712 | 6-Hydroxykaempferol | Androgen receptor | AR |
| honghua | MOL002712 | 6-Hydroxykaempferol | Peroxisome proliferator-activated receptor gamma | PPARG |
| honghua | MOL002712 | 6-Hydroxykaempferol | Prostaglandin G/H synthase 2 | PTGS2 |
| honghua | MOL002712 | 6-Hydroxykaempferol | Dipeptidyl peptidase 4 | DPP4 |
| honghua | MOL002712 | 6-Hydroxykaempferol | Heat shock protein HSP 90-alpha | HSP90AA1 |
| honghua | MOL002712 | 6-Hydroxykaempferol | Phosphatidylinositol-4,5-bisphosphate 3-kinase catalytic subunit gamma isoform | PIK3CG |
| honghua | MOL002714 | baicalein | Prostaglandin G/H synthase 1 | PTGS1 |
| honghua | MOL002714 | baicalein | Androgen receptor | AR |
| honghua | MOL002714 | baicalein | Apoptosis regulator Bcl-2 | BCL2 |
| honghua | MOL002714 | baicalein | Prostaglandin G/H synthase 2 | PTGS2 |
| honghua | MOL002714 | baicalein | cGMP-inhibited 3',5'-cyclic phosphodiesterase A | PDE3A |
| honghua | MOL002714 | baicalein | Dipeptidyl peptidase 4 | DPP4 |
| honghua | MOL002714 | baicalein | Myeloperoxidase | MPO |
| honghua | MOL002714 | baicalein | Heat shock protein HSP 90-alpha | HSP90AA1 |
| honghua | MOL002714 | baicalein | Phosphatidylinositol-4,5-bisphosphate 3-kinase catalytic subunit gamma isoform | PIK3CG |
| honghua | MOL002714 | baicalein | Cellular tumor antigen p53 | TP53 |
| honghua | MOL002714 | baicalein | Aryl hydrocarbon receptor | AHR |
| honghua | MOL002714 | baicalein | Nuclear receptor coactivator 1 | NCOA1 |
| honghua | MOL002714 | baicalein | Egl nine homolog 1 | EGLN1 |
| honghua | MOL002714 | baicalein | Cytochrome c | CYCS |
| honghua | MOL002714 | baicalein | Calmodulin | CALM3 |
| honghua | MOL002714 | baicalein | Apolipoprotein D | APOD |
| honghua | MOL002714 | baicalein | Apoptosis regulator BAX | BAX |
| honghua | MOL002714 | baicalein | Caspase-3 | CASP3 |
| honghua | MOL002714 | baicalein | Fatty acid-binding protein, epidermal | FABP5 |
| honghua | MOL002714 | baicalein | Hypoxia-inducible factor 1-alpha | HIF1A |
| honghua | MOL002714 | baicalein | Insulin-like growth factor II | IGF2 |
| honghua | MOL002714 | baicalein | Matrix metalloproteinase-9 | MMP9 |
| honghua | MOL002714 | baicalein | NADPH oxidase 5 | NOX5 |
| honghua | MOL002714 | baicalein | Nuclear factor of activated T-cells, cytoplasmic 1 | NFATC1 |
| honghua | MOL002714 | baicalein | Proto-oncogene c-Fos | FOS |
| honghua | MOL002714 | baicalein | RAC-alpha serine/threonine-protein kinase | AKT1 |
| honghua | MOL002714 | baicalein | Transcription factor p65 | RELA |
| honghua | MOL002714 | baicalein | Arachidonate 12-lipoxygenase, 12S-type | ALOX12 |
| honghua | MOL002717 | qt_carthamone | Prostaglandin G/H synthase 1 | PTGS1 |
| honghua | MOL002717 | qt_carthamone | Prostaglandin G/H synthase 2 | PTGS2 |
| honghua | MOL002717 | qt_carthamone | Heat shock protein HSP 90-alpha | HSP90AA1 |
| honghua | MOL002721 | quercetagetin | Androgen receptor | AR |
| honghua | MOL002721 | quercetagetin | Peroxisome proliferator-activated receptor gamma | PPARG |
| honghua | MOL002721 | quercetagetin | Prostaglandin G/H synthase 2 | PTGS2 |
| honghua | MOL002721 | quercetagetin | Dipeptidyl peptidase 4 | DPP4 |
| honghua | MOL002721 | quercetagetin | Heat shock protein HSP 90-alpha | HSP90AA1 |
| honghua | MOL002721 | quercetagetin | Phosphatidylinositol-4,5-bisphosphate 3-kinase catalytic subunit gamma isoform | PIK3CG |
| honghua | MOL002757 | 7,8-dimethyl-1H-pyrimido[5,6-g]quinoxaline-2,4-dione | Prostaglandin G/H synthase 1 | PTGS1 |
| honghua | MOL002757 | 7,8-dimethyl-1H-pyrimido[5,6-g]quinoxaline-2,4-dione | Prostaglandin G/H synthase 2 | PTGS2 |
| honghua | MOL002757 | 7,8-dimethyl-1H-pyrimido[5,6-g]quinoxaline-2,4-dione | Gamma-aminobutyric-acid receptor subunit alpha-1 | GABRA1 |
| honghua | MOL002757 | 7,8-dimethyl-1H-pyrimido[5,6-g]quinoxaline-2,4-dione | Heat shock protein HSP 90-alpha | HSP90AA1 |
| honghua | MOL002773 | beta-carotene | Apoptosis regulator Bcl-2 | BCL2 |
| honghua | MOL002773 | beta-carotene | Prostaglandin G/H synthase 2 | PTGS2 |
| honghua | MOL002773 | beta-carotene | 72 kDa type IV collagenase | MMP2 |
| honghua | MOL002773 | beta-carotene | Interstitial collagenase | MMP1 |
| honghua | MOL002773 | beta-carotene | Transcription factor AP-1 | JUN |
| honghua | MOL002773 | beta-carotene | Gap junction alpha-1 protein | GJA1 |
| honghua | MOL002773 | beta-carotene | Tissue factor | F3 |
| honghua | MOL002773 | beta-carotene | Cytochrome P450 3A4 | CYP3A4 |
| honghua | MOL002773 | beta-carotene | Heme oxygenase 1 | HMOX1 |
| honghua | MOL002773 | beta-carotene | Caspase-7 | CASP7 |
| honghua | MOL002773 | beta-carotene | Caspase-3 | CASP3 |
| honghua | MOL002773 | beta-carotene | Caspase-8 | CASP8 |
| honghua | MOL002773 | beta-carotene | Caspase-9 | CASP9 |
| honghua | MOL002773 | beta-carotene | Catenin beta-1 | CTNNB1 |
| honghua | MOL002773 | beta-carotene | Caveolin-1 | CAV1 |
| honghua | MOL002773 | beta-carotene | Cytochrome P450 2B1 | CYP2B6 |
| honghua | MOL002773 | beta-carotene | Myc proto-oncogene protein | MYC |
| honghua | MOL002773 | beta-carotene | RAC-alpha serine/threonine-protein kinase | AKT1 |
| honghua | MOL002773 | beta-carotene | Serum albumin | ALB |
| honghua | MOL002776 | Baicalin | Coagulation factor X | F10 |
| honghua | MOL002776 | Baicalin | Tyrosine-protein phosphatase non-receptor type 1 | PTPN1 |
