# Supplementary material for: Elucidating the mechanism of Buyang Huanwu Decoction in the treatment of ischemic stroke: A network pharmacology and molecular docking study
Source: Medicine (Baltimore). 2026 Jul 17;105(29):e49736. doi: 10.1097/MD.0000000000049736 (PMC13384647; doi:10.1097/MD.0000000000049736)
Supplement: Supplementary file 5 [file medi-105-e49736-s005.docx]

S 5. List of Relationships among Traditional Chinese Medicine Drugs, Active Ingredients, and Their Corresponding Degree.

| **Mol ID** | **Molecule name（Active Ingredients）** | **Degree** | **Chinese medicinal drugs** |
| --- | --- | --- | --- |
| MOL000098 | quercetin | 123 | huangqi, honghua |
| MOL000422 | kaempferol | 49 | huangqi, honghua |
| MOL000006 | luteolin | 45 | honghua |
| MOL000378 | 7-O-methylisomucronulatol | 35 | huangqi |
| MOL000354 | isorhamnetin | 32 | huangqi |
| MOL000392 | formononetin | 29 | huangqi |
| MOL000358 | beta-sitosterol | 29 | dilong, chishao, taoren, honghua |
| MOL002714 | baicalein | 28 | chishao, honghua |
| MOL002135 | Myricanone | 23 | chuanxiong |
| MOL000449 | Stigmasterol | 23 | danggui，chichao，honghua |
| MOL002773 | beta-carotene | 19 | honghua |
| MOL000371 | 3,9-di-O-methylnissolin | 18 | huangqi |
| MOL000417 | Calycosin | 18 | huangqi |
| MOL000380 | (6aR,11aR)-9,10-dimethoxy-6a,11a-dihydro-6H-benzofurano[3,2-c]chromen-3-ol | 16 | huangqi |
| MOL001002 | ellagic acid | 15 | chishao |
| MOL000296 | hederagenin | 13 | huangqi, taoren |
| MOL000239 | Jaranol | 11 | huangqi |
| MOL002712 | 6-Hydroxykaempferol | 8 | honghua |
| MOL000492 | (+)-catechin | 7 | chishao |
| MOL002695 | lignan | 6 | honghua |
| MOL002721 | quercetagetin | 6 | honghua |
| MOL000387 | Bifendate | 6 | huangqi |
| MOL001328 | 2,3-didehydro GA70 | 6 | taoren |
| MOL001358 | gibberellin 7 | 6 | taoren |
| MOL001368 | 3-O-p-coumaroylquinic acid | 6 | taoren |
| MOL006992 | (2R,3R)-4-methoxyl-distylin | 5 | chishao |
| MOL000493 | campesterol | 5 | taoren |
| MOL001340 | GA120 | 5 | taoren |
| MOL001352 | GA54 | 5 | taoren |
| MOL001924 | paeoniflorin | 4 | chishao |
| MOL002757 | 7,8-dimethyl-1H-pyrimido[5,6-g]quinoxaline-2,4-dione | 4 | honghua |
| MOL001323 | Sitosterol alpha1 | 4 | taoren |
| MOL001355 | GA63 | 4 | taoren |
| MOL002140 | Perlolyrine | 3 | chuanxiong |
| MOL002157 | wallichilide | 3 | chuanxiong |
| MOL002717 | qt_carthamone | 3 | honghua |
| MOL000433 | FA | 3 | huangqi |
| MOL000442 | 1,7-Dihydroxy-3,9-dimethoxy pterocarpene | 3 | huangqi |
| MOL001329 | 2,3-didehydro GA77 | 3 | taoren |
| MOL001353 | GA60 | 3 | taoren |
| MOL004355 | Spinasterol | 2 | chishao |
| MOL001494 | Mandenol | 2 | chuanxiong |
| MOL002694 | 4-[(E)-4-(3,5-dimethoxy-4-oxo-1-cyclohexa-2,5-dienylidene)but-2-enylidene]-2,6-dimethoxycyclohexa-2,5-dien-1-one | 2 | honghua |
| MOL000359 | sitosterol | 2 | chishao, chuanxiong |
| MOL002776 | Baicalin | 2 | chishao, honghua |
| MOL000953 | cholesterol | 2 | dilong, honghua |
| MOL001349 | 4a-formyl-7alpha-hydroxy-1-methyl-8-methylidene-4aalpha,4bbeta-gibbane-1alpha,10beta-dicarboxylic acid | 2 | taoren |
| MOL001351 | Gibberellin A44 | 2 | taoren |
| MOL001360 | GA77 | 2 | taoren |
| MOL001361 | GA87 | 2 | taoren |
| MOL001918 | paeoniflorgenone | 1 | chishao |
| MOL005043 | campest-5-en-3beta-ol | 1 | chishao |
| MOL006999 | stigmast-7-en-3-ol | 1 | chishao |
| MOL001771 | poriferast-5-en-3beta-ol | 1 | honghua |
| MOL002710 | Pyrethrin II | 1 | honghua |
| MOL000033 | (3S,8S,9S,10R,13R,14S,17R)-10,13-dimethyl-17-[(2R,5S)-5-propan-2-yloctan-2-yl]-2,3,4,7,8,9,11,12,14,15,16,17-dodecahydro-1H-cyclopenta[a]phenanthren-3-ol | 1 | huangqi |
| MOL000211 | Mairin | 1 | huangqi |
| MOL000379 | 9,10-dimethoxypterocarpan-3-O-β-D-glucoside | 1 | huangqi |
| MOL001342 | GA121-isolactone | 1 | taoren |
| MOL001344 | GA122-isolactone | 1 | taoren |
