# Supplementary material for: Elucidating the mechanism of Buyang Huanwu Decoction in the treatment of ischemic stroke: A network pharmacology and molecular docking study
Source: Medicine (Baltimore). 2026 Jul 17;105(29):e49736. doi: 10.1097/MD.0000000000049736 (PMC13384647; doi:10.1097/MD.0000000000049736)
Supplement: Supplementary file 6 [file medi-105-e49736-s006.docx]

**S 6.** Protein-protein interaction network data from STRING bioinformatics analysis.

| **node1** | **node2** | **node1_string_id** | **node2_string_id** | **neighborhood_on_chromosome** | **gene_fusion** | **phylogenetic_cooccurrence** | **homology** | **coexpression** | **experimentally_determined_interaction** | **database_annotated** | **automated_textmining** | **combined_score** |
| --- | --- | --- | --- | --- | --- | --- | --- | --- | --- | --- | --- | --- |
| ACHE | APP | 9606.ENSP00000303211 | 9606.ENSP00000284981 | 0 | 0 | 0 | 0 | 0.067 | 0.735 | 0 | 0.769 | 0.937 |
| ACHE | MAOB | 9606.ENSP00000303211 | 9606.ENSP00000367309 | 0 | 0 | 0 | 0 | 0.115 | 0 | 0 | 0.902 | 0.91 |
| ADRB1 | ADRB2 | 9606.ENSP00000358301 | 9606.ENSP00000305372 | 0 | 0 | 0 | 0.935 | 0.049 | 0.237 | 0.9 | 0.469 | 0.956 |
| AHR | ESR1 | 9606.ENSP00000242057 | 9606.ENSP00000405330 | 0 | 0 | 0 | 0 | 0.059 | 0.833 | 0 | 0.45 | 0.906 |
| AHR | CYP1A1 | 9606.ENSP00000242057 | 9606.ENSP00000378488 | 0 | 0 | 0 | 0 | 0.055 | 0 | 0.4 | 0.957 | 0.973 |
| AHR | HSP90AA1 | 9606.ENSP00000242057 | 9606.ENSP00000335153 | 0 | 0 | 0 | 0 | 0 | 0.869 | 0.8 | 0.717 | 0.992 |
| AHSA1 | HSP90AA1 | 9606.ENSP00000216479 | 9606.ENSP00000335153 | 0 | 0 | 0 | 0 | 0.477 | 0.968 | 0 | 0.997 | 0.999 |
| AKT1 | MAPK1 | 9606.ENSP00000451828 | 9606.ENSP00000215832 | 0 | 0 | 0 | 0.593 | 0.118 | 0.11 | 0.9 | 0.872 | 0.988 |
| AKT1 | CCND1 | 9606.ENSP00000451828 | 9606.ENSP00000227507 | 0 | 0 | 0 | 0 | 0.088 | 0 | 0 | 0.919 | 0.923 |
| AKT1 | MAPK14 | 9606.ENSP00000451828 | 9606.ENSP00000229795 | 0 | 0 | 0.119 | 0.621 | 0.246 | 0.739 | 0.9 | 0.535 | 0.99 |
| AKT1 | HSPB1 | 9606.ENSP00000451828 | 9606.ENSP00000248553 | 0 | 0 | 0 | 0 | 0.05 | 0.782 | 0 | 0.963 | 0.991 |
| AKT1 | MDM2 | 9606.ENSP00000451828 | 9606.ENSP00000258149 | 0 | 0 | 0 | 0 | 0 | 0.891 | 0.9 | 0.98 | 0.999 |
| AKT1 | TP53 | 9606.ENSP00000451828 | 9606.ENSP00000269305 | 0 | 0 | 0 | 0 | 0.049 | 0.184 | 0.9 | 0.934 | 0.994 |
| AKT1 | RASA1 | 9606.ENSP00000451828 | 9606.ENSP00000274376 | 0 | 0 | 0 | 0 | 0.06 | 0 | 0 | 0.901 | 0.902 |
| AKT1 | NCF1 | 9606.ENSP00000451828 | 9606.ENSP00000289473 | 0 | 0 | 0 | 0 | 0.086 | 0.101 | 0 | 0.993 | 0.993 |
| AKT1 | NOS3 | 9606.ENSP00000451828 | 9606.ENSP00000297494 | 0 | 0 | 0 | 0 | 0 | 0.786 | 0.9 | 0.909 | 0.997 |
| AKT1 | CASP3 | 9606.ENSP00000451828 | 9606.ENSP00000311032 | 0 | 0 | 0 | 0 | 0.044 | 0.311 | 0 | 0.923 | 0.945 |
| AKT1 | HSPA5 | 9606.ENSP00000451828 | 9606.ENSP00000324173 | 0 | 0 | 0 | 0 | 0.042 | 0.637 | 0 | 0.924 | 0.971 |
| AKT1 | GSK3B | 9606.ENSP00000451828 | 9606.ENSP00000324806 | 0 | 0 | 0 | 0.579 | 0.138 | 0.929 | 0.9 | 0.428 | 0.996 |
| AKT1 | NOS2 | 9606.ENSP00000451828 | 9606.ENSP00000327251 | 0 | 0 | 0 | 0 | 0 | 0.045 | 0.9 | 0.775 | 0.976 |
| AKT1 | CASP9 | 9606.ENSP00000451828 | 9606.ENSP00000330237 | 0 | 0 | 0 | 0 | 0.085 | 0.067 | 0.9 | 0.887 | 0.989 |
| AKT1 | HSP90AA1 | 9606.ENSP00000451828 | 9606.ENSP00000335153 | 0 | 0 | 0 | 0 | 0.049 | 0.883 | 0.9 | 0.996 | 0.999 |
| AKT1 | CAV1 | 9606.ENSP00000451828 | 9606.ENSP00000339191 | 0 | 0 | 0 | 0 | 0.074 | 0.046 | 0.5 | 0.875 | 0.937 |
| AKT1 | STAT1 | 9606.ENSP00000451828 | 9606.ENSP00000354394 | 0 | 0 | 0 | 0 | 0.106 | 0.096 | 0.4 | 0.93 | 0.961 |
| AKT1 | CHUK | 9606.ENSP00000451828 | 9606.ENSP00000359424 | 0 | 0 | 0 | 0.594 | 0.11 | 0.835 | 0.9 | 0.814 | 0.996 |
| AKT1 | XIAP | 9606.ENSP00000451828 | 9606.ENSP00000360242 | 0 | 0 | 0 | 0 | 0.049 | 0.786 | 0 | 0.725 | 0.939 |
| AKT1 | JUN | 9606.ENSP00000451828 | 9606.ENSP00000360266 | 0 | 0 | 0 | 0 | 0.049 | 0.085 | 0.9 | 0.915 | 0.991 |
| AKT1 | PTEN | 9606.ENSP00000451828 | 9606.ENSP00000361021 | 0 | 0 | 0 | 0 | 0 | 0.648 | 0 | 0.967 | 0.988 |
| AKT1 | BCL2L1 | 9606.ENSP00000451828 | 9606.ENSP00000365230 | 0 | 0 | 0 | 0 | 0.095 | 0.292 | 0.9 | 0.888 | 0.991 |
| AKT1 | NFE2L2 | 9606.ENSP00000451828 | 9606.ENSP00000380252 | 0 | 0 | 0 | 0 | 0.049 | 0.317 | 0.4 | 0.825 | 0.923 |
| AKT1 | BCL2 | 9606.ENSP00000451828 | 9606.ENSP00000381185 | 0 | 0 | 0 | 0 | 0 | 0 | 0.9 | 0.934 | 0.993 |
| AKT1 | CDKN1A | 9606.ENSP00000451828 | 9606.ENSP00000384849 | 0 | 0 | 0 | 0 | 0 | 0.776 | 0.9 | 0.833 | 0.995 |
| AKT1 | TNF | 9606.ENSP00000451828 | 9606.ENSP00000398698 | 0 | 0 | 0 | 0 | 0.074 | 0.057 | 0 | 0.903 | 0.907 |
| AKT1 | RAF1 | 9606.ENSP00000451828 | 9606.ENSP00000401888 | 0 | 0 | 0.145 | 0.583 | 0.075 | 0.816 | 0.9 | 0.557 | 0.992 |
| AKT1 | ESR1 | 9606.ENSP00000451828 | 9606.ENSP00000405330 | 0 | 0 | 0 | 0 | 0.055 | 0.314 | 0 | 0.877 | 0.913 |
| AKT1 | PIK3CG | 9606.ENSP00000451828 | 9606.ENSP00000419260 | 0 | 0 | 0 | 0 | 0 | 0.098 | 0.9 | 0.947 | 0.994 |
| AKT1 | IKBKB | 9606.ENSP00000451828 | 9606.ENSP00000430684 | 0 | 0 | 0 | 0.601 | 0.152 | 0.775 | 0.9 | 0.57 | 0.99 |
| AKT1 | HIF1A | 9606.ENSP00000451828 | 9606.ENSP00000437955 | 0 | 0 | 0 | 0 | 0 | 0.229 | 0 | 0.916 | 0.932 |
| AKT1 | MYC | 9606.ENSP00000451828 | 9606.ENSP00000478887 | 0 | 0 | 0 | 0 | 0 | 0.169 | 0 | 0.918 | 0.928 |
| AKT1 | CTNNB1 | 9606.ENSP00000451828 | 9606.ENSP00000495360 | 0 | 0 | 0 | 0 | 0.074 | 0.539 | 0.4 | 0.93 | 0.979 |
| ALB | CRP | 9606.ENSP00000295897 | 9606.ENSP00000255030 | 0 | 0 | 0 | 0 | 0.164 | 0 | 0 | 0.918 | 0.928 |
| ALB | APP | 9606.ENSP00000295897 | 9606.ENSP00000284981 | 0 | 0 | 0 | 0 | 0 | 0.292 | 0.4 | 0.787 | 0.901 |
| ALB | IL6 | 9606.ENSP00000295897 | 9606.ENSP00000385675 | 0 | 0 | 0 | 0 | 0 | 0 | 0 | 0.926 | 0.926 |
| ALB | CYCS | 9606.ENSP00000295897 | 9606.ENSP00000307786 | 0 | 0 | 0 | 0 | 0.056 | 0 | 0 | 0.986 | 0.986 |
| ALB | F2 | 9606.ENSP00000295897 | 9606.ENSP00000308541 | 0 | 0 | 0 | 0 | 0.652 | 0.052 | 0 | 0.973 | 0.99 |
| ALOX12 | CYP2B6 | 9606.ENSP00000251535 | 9606.ENSP00000324648 | 0 | 0 | 0 | 0 | 0.062 | 0.065 | 0.9 | 0.064 | 0.906 |
| ALOX12 | ALOX5 | 9606.ENSP00000251535 | 9606.ENSP00000363512 | 0 | 0 | 0.084 | 0.907 | 0.06 | 0 | 0.9 | 0.119 | 0.914 |
| ALOX12 | PTGS2 | 9606.ENSP00000251535 | 9606.ENSP00000356438 | 0 | 0 | 0 | 0 | 0.055 | 0 | 0.9 | 0.488 | 0.947 |
| ALOX12 | PTGS1 | 9606.ENSP00000251535 | 9606.ENSP00000354612 | 0 | 0 | 0 | 0 | 0.201 | 0 | 0.9 | 0.544 | 0.96 |
| ALOX5 | LTA4H | 9606.ENSP00000363512 | 9606.ENSP00000228740 | 0 | 0 | 0 | 0 | 0.104 | 0.05 | 0.9 | 0.924 | 0.992 |
| ALOX5 | CYP2B6 | 9606.ENSP00000363512 | 9606.ENSP00000324648 | 0 | 0 | 0 | 0 | 0.064 | 0.065 | 0.9 | 0.169 | 0.917 |
| ALOX5 | PTGS1 | 9606.ENSP00000363512 | 9606.ENSP00000354612 | 0 | 0 | 0 | 0 | 0.13 | 0 | 0.9 | 0.692 | 0.97 |
| ALOX5 | PTGS2 | 9606.ENSP00000363512 | 9606.ENSP00000356438 | 0 | 0 | 0 | 0 | 0.072 | 0 | 0.9 | 0.741 | 0.973 |
| APP | CASP3 | 9606.ENSP00000284981 | 9606.ENSP00000311032 | 0 | 0 | 0 | 0 | 0.06 | 0.603 | 0 | 0.774 | 0.908 |
| AR | CCND1 | 9606.ENSP00000363822 | 9606.ENSP00000227507 | 0 | 0 | 0 | 0 | 0.072 | 0.738 | 0 | 0.831 | 0.955 |
| AR | MDM2 | 9606.ENSP00000363822 | 9606.ENSP00000258149 | 0 | 0 | 0 | 0 | 0 | 0.92 | 0 | 0.979 | 0.998 |
| AR | TP53 | 9606.ENSP00000363822 | 9606.ENSP00000269305 | 0 | 0 | 0 | 0 | 0 | 0.439 | 0 | 0.838 | 0.905 |
| AR | HSP90AA1 | 9606.ENSP00000363822 | 9606.ENSP00000335153 | 0 | 0 | 0 | 0 | 0.054 | 0.901 | 0.9 | 0.99 | 0.999 |
| AR | ESR1 | 9606.ENSP00000363822 | 9606.ENSP00000405330 | 0 | 0 | 0 | 0.651 | 0.172 | 0 | 0 | 0.978 | 0.981 |
| AR | NCOA1 | 9606.ENSP00000363822 | 9606.ENSP00000385216 | 0 | 0 | 0 | 0 | 0.058 | 0.863 | 0 | 0.887 | 0.984 |
| AR | CTNNB1 | 9606.ENSP00000363822 | 9606.ENSP00000495360 | 0 | 0 | 0 | 0 | 0.052 | 0.888 | 0 | 0.968 | 0.996 |
| BAX | SIRT1 | 9606.ENSP00000293288 | 9606.ENSP00000212015 | 0 | 0 | 0 | 0 | 0.058 | 0 | 0.9 | 0.438 | 0.942 |
| BAX | TP53 | 9606.ENSP00000293288 | 9606.ENSP00000269305 | 0 | 0 | 0 | 0 | 0.08 | 0.782 | 0.9 | 0.774 | 0.994 |
| BAX | HK2 | 9606.ENSP00000293288 | 9606.ENSP00000290573 | 0 | 0 | 0 | 0 | 0.057 | 0 | 0.9 | 0.213 | 0.919 |
| BAX | CYCS | 9606.ENSP00000293288 | 9606.ENSP00000307786 | 0 | 0 | 0 | 0 | 0.055 | 0.287 | 0.9 | 0.91 | 0.993 |
| BAX | BCL2 | 9606.ENSP00000293288 | 9606.ENSP00000381185 | 0 | 0 | 0 | 0.641 | 0 | 0.991 | 0.9 | 0.972 | 0.999 |
| BAX | BCL2L1 | 9606.ENSP00000293288 | 9606.ENSP00000365230 | 0 | 0 | 0 | 0.639 | 0.082 | 0.999 | 0.9 | 0.882 | 0.999 |
| BCL2 | MAPK1 | 9606.ENSP00000381185 | 9606.ENSP00000215832 | 0 | 0 | 0 | 0 | 0.086 | 0.292 | 0.9 | 0.586 | 0.969 |
| BCL2 | CCND1 | 9606.ENSP00000381185 | 9606.ENSP00000227507 | 0 | 0 | 0 | 0 | 0.052 | 0 | 0 | 0.957 | 0.958 |
| BCL2 | CTSD | 9606.ENSP00000381185 | 9606.ENSP00000236671 | 0 | 0 | 0 | 0 | 0 | 0 | 0.9 | 0.514 | 0.949 |
| BCL2 | MDM2 | 9606.ENSP00000381185 | 9606.ENSP00000258149 | 0 | 0 | 0 | 0 | 0.056 | 0 | 0 | 0.953 | 0.953 |
| BCL2 | TP53 | 9606.ENSP00000381185 | 9606.ENSP00000269305 | 0 | 0 | 0 | 0 | 0.103 | 0.869 | 0.9 | 0.999 | 0.999 |
| BCL2 | SOD1 | 9606.ENSP00000381185 | 9606.ENSP00000270142 | 0 | 0 | 0 | 0 | 0 | 0.329 | 0.9 | 0.976 | 0.998 |
| BCL2 | CYCS | 9606.ENSP00000381185 | 9606.ENSP00000307786 | 0 | 0 | 0 | 0 | 0 | 0 | 0.9 | 0.972 | 0.997 |
| BCL2 | CASP3 | 9606.ENSP00000381185 | 9606.ENSP00000311032 | 0 | 0 | 0 | 0 | 0.098 | 0.354 | 0 | 0.98 | 0.987 |
| BCL2 | CASP9 | 9606.ENSP00000381185 | 9606.ENSP00000330237 | 0 | 0 | 0 | 0 | 0.091 | 0.125 | 0 | 0.938 | 0.946 |
| BCL2 | HSP90AA1 | 9606.ENSP00000381185 | 9606.ENSP00000335153 | 0 | 0 | 0 | 0 | 0.06 | 0.51 | 0 | 0.859 | 0.929 |
| BCL2 | CASP8 | 9606.ENSP00000381185 | 9606.ENSP00000351273 | 0 | 0 | 0 | 0 | 0.086 | 0.648 | 0 | 0.954 | 0.984 |
| BCL2 | PARP1 | 9606.ENSP00000381185 | 9606.ENSP00000355759 | 0 | 0 | 0 | 0 | 0.112 | 0.292 | 0 | 0.879 | 0.917 |
| BCL2 | XIAP | 9606.ENSP00000381185 | 9606.ENSP00000360242 | 0 | 0 | 0 | 0 | 0.048 | 0.299 | 0 | 0.933 | 0.951 |
| BCL2 | JUN | 9606.ENSP00000381185 | 9606.ENSP00000360266 | 0 | 0 | 0 | 0 | 0.047 | 0 | 0 | 0.927 | 0.927 |
| BCL2 | BCL2L1 | 9606.ENSP00000381185 | 9606.ENSP00000365230 | 0 | 0 | 0 | 0.894 | 0 | 0.863 | 0.9 | 0.984 | 0.999 |
| BCL2 | MAPK8 | 9606.ENSP00000381185 | 9606.ENSP00000378974 | 0 | 0 | 0 | 0 | 0.094 | 0.287 | 0.9 | 0.713 | 0.979 |
| BCL2 | HIF1A | 9606.ENSP00000381185 | 9606.ENSP00000437955 | 0 | 0 | 0 | 0 | 0 | 0.51 | 0 | 0.895 | 0.946 |
| BCL2 | MYC | 9606.ENSP00000381185 | 9606.ENSP00000478887 | 0 | 0 | 0 | 0 | 0.065 | 0.3 | 0 | 0.937 | 0.955 |
| BCL2L1 | IL2 | 9606.ENSP00000365230 | 9606.ENSP00000226730 | 0 | 0 | 0 | 0 | 0.083 | 0 | 0 | 0.908 | 0.912 |
| BCL2L1 | CTSD | 9606.ENSP00000365230 | 9606.ENSP00000236671 | 0 | 0 | 0 | 0 | 0.074 | 0 | 0.9 | 0.344 | 0.934 |
| BCL2L1 | TP53 | 9606.ENSP00000365230 | 9606.ENSP00000269305 | 0 | 0 | 0 | 0 | 0.074 | 0.986 | 0 | 0.998 | 0.999 |
| BCL2L1 | CYCS | 9606.ENSP00000365230 | 9606.ENSP00000307786 | 0 | 0 | 0 | 0 | 0.054 | 0.678 | 0.9 | 0.955 | 0.998 |
| BCL2L1 | CASP3 | 9606.ENSP00000365230 | 9606.ENSP00000311032 | 0 | 0 | 0 | 0 | 0.044 | 0.209 | 0 | 0.94 | 0.95 |
| BCL2L1 | CASP9 | 9606.ENSP00000365230 | 9606.ENSP00000330237 | 0 | 0 | 0 | 0 | 0.082 | 0.822 | 0 | 0.959 | 0.992 |
| BCL2L1 | CASP8 | 9606.ENSP00000365230 | 9606.ENSP00000351273 | 0 | 0 | 0 | 0 | 0.042 | 0.51 | 0 | 0.869 | 0.933 |
| BCL2L1 | XIAP | 9606.ENSP00000365230 | 9606.ENSP00000360242 | 0 | 0 | 0 | 0 | 0.082 | 0.051 | 0 | 0.936 | 0.939 |
| BCL2L1 | MAPK8 | 9606.ENSP00000365230 | 9606.ENSP00000378974 | 0 | 0 | 0 | 0 | 0.057 | 0.678 | 0.9 | 0.593 | 0.986 |
| BIRC5 | TP53 | 9606.ENSP00000301633 | 9606.ENSP00000269305 | 0 | 0 | 0 | 0 | 0.143 | 0 | 0.75 | 0.703 | 0.93 |
| BIRC5 | CASP9 | 9606.ENSP00000301633 | 9606.ENSP00000330237 | 0 | 0 | 0 | 0 | 0.124 | 0.728 | 0 | 0.658 | 0.911 |
| BIRC5 | CASP3 | 9606.ENSP00000301633 | 9606.ENSP00000311032 | 0 | 0 | 0 | 0 | 0.132 | 0.583 | 0 | 0.786 | 0.916 |
| BIRC5 | CCNA2 | 9606.ENSP00000301633 | 9606.ENSP00000481380 | 0 | 0 | 0 | 0 | 0.857 | 0 | 0 | 0.707 | 0.956 |
| BIRC5 | XIAP | 9606.ENSP00000301633 | 9606.ENSP00000360242 | 0 | 0 | 0 | 0 | 0 | 0.735 | 0 | 0.855 | 0.96 |
| CALM3 | NOX5 | 9606.ENSP00000291295 | 9606.ENSP00000373518 | 0 | 0 | 0 | 0 | 0 | 0.967 | 0 | 0.666 | 0.988 |
| CALM3 | CAV1 | 9606.ENSP00000291295 | 9606.ENSP00000339191 | 0 | 0 | 0 | 0 | 0.045 | 0 | 0.8 | 0.959 | 0.991 |
| CALM3 | ESR1 | 9606.ENSP00000291295 | 9606.ENSP00000405330 | 0 | 0 | 0 | 0 | 0 | 0.916 | 0 | 0.942 | 0.995 |
| CALM3 | HSP90AA1 | 9606.ENSP00000291295 | 9606.ENSP00000335153 | 0 | 0 | 0 | 0 | 0.045 | 0.071 | 0.8 | 0.984 | 0.996 |
| CALM3 | NOS3 | 9606.ENSP00000291295 | 9606.ENSP00000297494 | 0 | 0 | 0 | 0 | 0 | 0.927 | 0.9 | 0.996 | 0.999 |
| CALM3 | PPP3CA | 9606.ENSP00000291295 | 9606.ENSP00000378323 | 0 | 0 | 0 | 0 | 0.051 | 0.927 | 0.9 | 0.983 | 0.999 |
| CALM3 | NOS2 | 9606.ENSP00000291295 | 9606.ENSP00000327251 | 0 | 0 | 0 | 0 | 0 | 0.902 | 0.9 | 0.927 | 0.999 |
| CALM3 | SCN5A | 9606.ENSP00000291295 | 9606.ENSP00000328968 | 0 | 0 | 0 | 0 | 0 | 0.988 | 0 | 0.986 | 0.999 |
| CASP3 | MAPK1 | 9606.ENSP00000311032 | 9606.ENSP00000215832 | 0 | 0 | 0 | 0 | 0.1 | 0.045 | 0.9 | 0.599 | 0.961 |
| CASP3 | MDM2 | 9606.ENSP00000311032 | 9606.ENSP00000258149 | 0 | 0 | 0 | 0 | 0.066 | 0.832 | 0 | 0.803 | 0.966 |
| CASP3 | TP53 | 9606.ENSP00000311032 | 9606.ENSP00000269305 | 0 | 0 | 0 | 0 | 0.093 | 0.292 | 0 | 0.937 | 0.956 |
| CASP3 | CYCS | 9606.ENSP00000311032 | 9606.ENSP00000307786 | 0 | 0 | 0 | 0 | 0.074 | 0 | 0.9 | 0.975 | 0.997 |
| CASP3 | PRKCB | 9606.ENSP00000311032 | 9606.ENSP00000496129 | 0 | 0 | 0 | 0 | 0.104 | 0 | 0.9 | 0.301 | 0.931 |
| CASP3 | TNF | 9606.ENSP00000311032 | 9606.ENSP00000398698 | 0 | 0 | 0 | 0 | 0.098 | 0.096 | 0 | 0.932 | 0.939 |
| CASP3 | MAPK8 | 9606.ENSP00000311032 | 9606.ENSP00000378974 | 0 | 0 | 0 | 0 | 0.049 | 0.292 | 0 | 0.922 | 0.943 |
| CASP3 | PRKCA | 9606.ENSP00000311032 | 9606.ENSP00000408695 | 0 | 0 | 0 | 0 | 0.089 | 0 | 0.9 | 0.48 | 0.948 |
| CASP3 | CASP7 | 9606.ENSP00000311032 | 9606.ENSP00000358327 | 0 | 0 | 0.069 | 0.94 | 0.134 | 0.421 | 0.9 | 0.096 | 0.95 |
| CASP3 | CTNNB1 | 9606.ENSP00000311032 | 9606.ENSP00000495360 | 0 | 0 | 0 | 0 | 0.088 | 0.395 | 0.75 | 0.782 | 0.965 |
| CASP3 | CDKN1A | 9606.ENSP00000311032 | 9606.ENSP00000384849 | 0 | 0 | 0 | 0 | 0.057 | 0.828 | 0.9 | 0.806 | 0.996 |
| CASP3 | CASP8 | 9606.ENSP00000311032 | 9606.ENSP00000351273 | 0 | 0 | 0.088 | 0.828 | 0.105 | 0.786 | 0.9 | 0.983 | 0.999 |
| CASP3 | XIAP | 9606.ENSP00000311032 | 9606.ENSP00000360242 | 0 | 0 | 0 | 0 | 0.069 | 0.998 | 0.9 | 0.998 | 0.999 |
| CASP3 | PARP1 | 9606.ENSP00000311032 | 9606.ENSP00000355759 | 0 | 0 | 0 | 0 | 0.064 | 0.867 | 0.9 | 0.953 | 0.999 |
| CASP3 | CASP9 | 9606.ENSP00000311032 | 9606.ENSP00000330237 | 0 | 0 | 0 | 0.827 | 0.049 | 0.846 | 0.9 | 0.957 | 0.999 |
| CASP7 | CYCS | 9606.ENSP00000358327 | 9606.ENSP00000307786 | 0 | 0 | 0 | 0 | 0.076 | 0 | 0.4 | 0.903 | 0.942 |
| CASP7 | HSPA5 | 9606.ENSP00000358327 | 9606.ENSP00000324173 | 0 | 0 | 0 | 0 | 0.042 | 0.524 | 0 | 0.932 | 0.966 |
| CASP7 | CASP9 | 9606.ENSP00000358327 | 9606.ENSP00000330237 | 0 | 0 | 0 | 0.704 | 0.06 | 0.326 | 0.9 | 0.343 | 0.952 |
| CASP7 | CASP8 | 9606.ENSP00000358327 | 9606.ENSP00000351273 | 0 | 0 | 0.083 | 0.746 | 0.109 | 0.628 | 0.9 | 0.788 | 0.992 |
| CASP7 | PARP1 | 9606.ENSP00000358327 | 9606.ENSP00000355759 | 0 | 0 | 0 | 0 | 0.062 | 0.706 | 0.9 | 0.848 | 0.995 |
| CASP7 | XIAP | 9606.ENSP00000358327 | 9606.ENSP00000360242 | 0 | 0 | 0 | 0 | 0.09 | 0.993 | 0.9 | 0.997 | 0.999 |
| CASP8 | IL1B | 9606.ENSP00000351273 | 9606.ENSP00000263341 | 0 | 0 | 0 | 0 | 0.081 | 0.32 | 0.5 | 0.839 | 0.943 |
| CASP8 | TP53 | 9606.ENSP00000351273 | 9606.ENSP00000269305 | 0 | 0 | 0 | 0 | 0.086 | 0.292 | 0.9 | 0.879 | 0.991 |
| CASP8 | CYCS | 9606.ENSP00000351273 | 9606.ENSP00000307786 | 0 | 0 | 0 | 0 | 0.058 | 0 | 0 | 0.951 | 0.952 |
| CASP8 | CASP9 | 9606.ENSP00000351273 | 9606.ENSP00000330237 | 0 | 0 | 0 | 0.7 | 0.104 | 0.628 | 0.54 | 0.678 | 0.944 |
| CASP8 | CHUK | 9606.ENSP00000351273 | 9606.ENSP00000359424 | 0 | 0 | 0 | 0 | 0.093 | 0.311 | 0.5 | 0.861 | 0.95 |
| CASP8 | XIAP | 9606.ENSP00000351273 | 9606.ENSP00000360242 | 0 | 0 | 0 | 0 | 0.085 | 0.676 | 0 | 0.909 | 0.97 |
| CASP8 | TNF | 9606.ENSP00000351273 | 9606.ENSP00000398698 | 0 | 0 | 0 | 0 | 0.101 | 0.994 | 0 | 0.928 | 0.999 |
| CASP9 | TP53 | 9606.ENSP00000330237 | 9606.ENSP00000269305 | 0 | 0 | 0 | 0 | 0.067 | 0 | 0 | 0.952 | 0.953 |
| CASP9 | CYCS | 9606.ENSP00000330237 | 9606.ENSP00000307786 | 0 | 0 | 0 | 0 | 0.068 | 0.915 | 0.9 | 0.999 | 0.999 |
| CASP9 | PARP1 | 9606.ENSP00000330237 | 9606.ENSP00000355759 | 0 | 0 | 0 | 0 | 0.049 | 0.091 | 0.9 | 0.915 | 0.991 |
| CASP9 | XIAP | 9606.ENSP00000330237 | 9606.ENSP00000360242 | 0 | 0 | 0 | 0 | 0.085 | 0.997 | 0.9 | 0.998 | 0.999 |
| CAV1 | KDR | 9606.ENSP00000339191 | 9606.ENSP00000263923 | 0 | 0 | 0 | 0 | 0.071 | 0.301 | 0 | 0.947 | 0.962 |
| CAV1 | ICAM1 | 9606.ENSP00000339191 | 9606.ENSP00000264832 | 0 | 0 | 0 | 0 | 0.074 | 0 | 0 | 0.932 | 0.934 |
| CAV1 | EGFR | 9606.ENSP00000339191 | 9606.ENSP00000275493 | 0 | 0 | 0 | 0 | 0.206 | 0.882 | 0.8 | 0.986 | 0.999 |
| CAV1 | GJA1 | 9606.ENSP00000339191 | 9606.ENSP00000282561 | 0 | 0 | 0 | 0 | 0.165 | 0.329 | 0.4 | 0.908 | 0.965 |
| CAV1 | NOS3 | 9606.ENSP00000339191 | 9606.ENSP00000297494 | 0 | 0 | 0 | 0 | 0.044 | 0.736 | 0.8 | 0.995 | 0.999 |
| CAV1 | HSP90AA1 | 9606.ENSP00000339191 | 9606.ENSP00000335153 | 0 | 0 | 0 | 0 | 0.075 | 0.292 | 0.8 | 0.92 | 0.988 |
| CAV1 | PTEN | 9606.ENSP00000339191 | 9606.ENSP00000361021 | 0 | 0 | 0 | 0 | 0 | 0.345 | 0 | 0.872 | 0.912 |
| CAV1 | CTNNB1 | 9606.ENSP00000339191 | 9606.ENSP00000495360 | 0 | 0 | 0 | 0 | 0.043 | 0.514 | 0 | 0.886 | 0.942 |
| CAV1 | ESR2 | 9606.ENSP00000339191 | 9606.ENSP00000343925 | 0 | 0 | 0 | 0 | 0.053 | 0.049 | 0.5 | 0.945 | 0.971 |
| CAV1 | DPP4 | 9606.ENSP00000339191 | 9606.ENSP00000353731 | 0 | 0 | 0 | 0 | 0.058 | 0 | 0 | 0.991 | 0.991 |
| CAV1 | ESR1 | 9606.ENSP00000339191 | 9606.ENSP00000405330 | 0 | 0 | 0 | 0 | 0.053 | 0.624 | 0.5 | 0.992 | 0.998 |
| CCL2 | SERPINE1 | 9606.ENSP00000225831 | 9606.ENSP00000223095 | 0 | 0 | 0 | 0 | 0.259 | 0 | 0 | 0.895 | 0.918 |
| CCL2 | VCAM1 | 9606.ENSP00000225831 | 9606.ENSP00000294728 | 0 | 0 | 0 | 0 | 0.209 | 0.096 | 0 | 0.88 | 0.907 |
| CCL2 | IL4 | 9606.ENSP00000225831 | 9606.ENSP00000231449 | 0 | 0 | 0 | 0 | 0.045 | 0 | 0.4 | 0.873 | 0.921 |
| CCL2 | ICAM1 | 9606.ENSP00000225831 | 9606.ENSP00000264832 | 0 | 0 | 0 | 0 | 0.36 | 0 | 0 | 0.892 | 0.928 |
| CCL2 | CXCL2 | 9606.ENSP00000225831 | 9606.ENSP00000427279 | 0 | 0 | 0 | 0 | 0.451 | 0 | 0.4 | 0.803 | 0.929 |
| CCL2 | IL1A | 9606.ENSP00000225831 | 9606.ENSP00000263339 | 0 | 0 | 0 | 0 | 0.144 | 0 | 0.4 | 0.882 | 0.934 |
| CCL2 | IFNG | 9606.ENSP00000225831 | 9606.ENSP00000229135 | 0 | 0 | 0 | 0 | 0.195 | 0 | 0 | 0.926 | 0.938 |
| CCL2 | IL10 | 9606.ENSP00000225831 | 9606.ENSP00000412237 | 0 | 0 | 0 | 0 | 0.214 | 0 | 0.4 | 0.929 | 0.963 |
| CCL2 | CXCL10 | 9606.ENSP00000225831 | 9606.ENSP00000305651 | 0 | 0 | 0 | 0 | 0.373 | 0 | 0.4 | 0.915 | 0.965 |
| CCL2 | IL1B | 9606.ENSP00000225831 | 9606.ENSP00000263341 | 0 | 0 | 0 | 0 | 0.239 | 0 | 0.4 | 0.94 | 0.97 |
| CCL2 | TNF | 9606.ENSP00000225831 | 9606.ENSP00000398698 | 0 | 0 | 0 | 0 | 0.311 | 0 | 0.4 | 0.952 | 0.978 |
| CCL2 | JUN | 9606.ENSP00000225831 | 9606.ENSP00000360266 | 0 | 0 | 0 | 0 | 0.083 | 0 | 0.9 | 0.8 | 0.98 |
| CCL2 | IL6 | 9606.ENSP00000225831 | 9606.ENSP00000385675 | 0 | 0 | 0 | 0 | 0.517 | 0 | 0.4 | 0.953 | 0.985 |
| CCL2 | RELA | 9606.ENSP00000225831 | 9606.ENSP00000384273 | 0 | 0 | 0 | 0 | 0.113 | 0.51 | 0.9 | 0.745 | 0.987 |
| CCL2 | CXCL8 | 9606.ENSP00000225831 | 9606.ENSP00000306512 | 0 | 0 | 0 | 0 | 0.487 | 0.994 | 0.4 | 0.948 | 0.999 |
| CCNA2 | CDK2 | 9606.ENSP00000481380 | 9606.ENSP00000266970 | 0 | 0.003 | 0 | 0 | 0.453 | 0.999 | 0.9 | 0.999 | 0.999 |
| CCNA2 | RB1 | 9606.ENSP00000481380 | 9606.ENSP00000267163 | 0 | 0 | 0 | 0 | 0.162 | 0.847 | 0.9 | 0.911 | 0.998 |
| CCNA2 | TP53 | 9606.ENSP00000481380 | 9606.ENSP00000269305 | 0 | 0 | 0 | 0 | 0.099 | 0.292 | 0.5 | 0.994 | 0.997 |
| CCNA2 | E2F1 | 9606.ENSP00000481380 | 9606.ENSP00000345571 | 0 | 0 | 0 | 0 | 0.244 | 0.813 | 0.9 | 0.989 | 0.999 |
| CCNA2 | PCNA | 9606.ENSP00000481380 | 9606.ENSP00000368458 | 0 | 0 | 0 | 0 | 0.447 | 0.794 | 0 | 0.398 | 0.925 |
| CCNA2 | CDKN1A | 9606.ENSP00000481380 | 9606.ENSP00000384849 | 0 | 0 | 0 | 0 | 0 | 0.924 | 0.9 | 0.995 | 0.999 |
| CCNA2 | CHEK1 | 9606.ENSP00000481380 | 9606.ENSP00000391090 | 0 | 0 | 0 | 0 | 0.79 | 0 | 0 | 0.721 | 0.939 |
| CCND1 | ERBB2 | 9606.ENSP00000227507 | 9606.ENSP00000269571 | 0 | 0 | 0 | 0 | 0.084 | 0.301 | 0 | 0.862 | 0.904 |
| CCND1 | JUN | 9606.ENSP00000227507 | 9606.ENSP00000360266 | 0 | 0 | 0 | 0 | 0.042 | 0.292 | 0.5 | 0.79 | 0.919 |
| CCND1 | MDM2 | 9606.ENSP00000227507 | 9606.ENSP00000258149 | 0 | 0 | 0 | 0 | 0.055 | 0.292 | 0 | 0.898 | 0.926 |
| CCND1 | E2F1 | 9606.ENSP00000227507 | 9606.ENSP00000345571 | 0 | 0 | 0 | 0 | 0.062 | 0.068 | 0.5 | 0.876 | 0.938 |
| CCND1 | FOS | 9606.ENSP00000227507 | 9606.ENSP00000306245 | 0 | 0 | 0 | 0 | 0.055 | 0.469 | 0.5 | 0.799 | 0.942 |
| CCND1 | TP53 | 9606.ENSP00000227507 | 9606.ENSP00000269305 | 0 | 0 | 0 | 0 | 0 | 0.292 | 0 | 0.928 | 0.946 |
| CCND1 | MYC | 9606.ENSP00000227507 | 9606.ENSP00000478887 | 0 | 0 | 0 | 0 | 0.067 | 0 | 0.4 | 0.95 | 0.969 |
| CCND1 | CTNNB1 | 9606.ENSP00000227507 | 9606.ENSP00000495360 | 0 | 0 | 0 | 0 | 0.073 | 0.3 | 0.5 | 0.926 | 0.972 |
| CCND1 | GSK3B | 9606.ENSP00000227507 | 9606.ENSP00000324806 | 0 | 0 | 0 | 0 | 0 | 0.409 | 0.9 | 0.803 | 0.987 |
| CCND1 | PCNA | 9606.ENSP00000227507 | 9606.ENSP00000368458 | 0 | 0 | 0 | 0 | 0.049 | 0.841 | 0.9 | 0.373 | 0.989 |
| CCND1 | ESR1 | 9606.ENSP00000227507 | 9606.ENSP00000405330 | 0 | 0 | 0 | 0 | 0.069 | 0.625 | 0.75 | 0.971 | 0.997 |
| CCND1 | RB1 | 9606.ENSP00000227507 | 9606.ENSP00000267163 | 0 | 0 | 0 | 0 | 0 | 0.932 | 0.9 | 0.831 | 0.998 |
| CCND1 | CDKN2A | 9606.ENSP00000227507 | 9606.ENSP00000418915 | 0 | 0 | 0 | 0 | 0 | 0.726 | 0.9 | 0.946 | 0.998 |
| CCND1 | CDK2 | 9606.ENSP00000227507 | 9606.ENSP00000266970 | 0 | 0 | 0 | 0 | 0.135 | 0.901 | 0.8 | 0.998 | 0.999 |
| CCND1 | CDKN1A | 9606.ENSP00000227507 | 9606.ENSP00000384849 | 0 | 0 | 0 | 0 | 0.118 | 0.996 | 0.9 | 0.995 | 0.999 |
| CD14 | LBP | 9606.ENSP00000385519 | 9606.ENSP00000217407 | 0 | 0 | 0 | 0 | 0.057 | 0.625 | 0.6 | 0.945 | 0.991 |
| CD40LG | IL2 | 9606.ENSP00000359663 | 9606.ENSP00000226730 | 0 | 0 | 0 | 0 | 0.217 | 0 | 0 | 0.907 | 0.924 |
| CD40LG | IFNG | 9606.ENSP00000359663 | 9606.ENSP00000229135 | 0 | 0 | 0 | 0 | 0.14 | 0 | 0 | 0.909 | 0.919 |
| CD40LG | IL4 | 9606.ENSP00000359663 | 9606.ENSP00000231449 | 0 | 0 | 0 | 0 | 0.088 | 0 | 0 | 0.91 | 0.914 |
| CD40LG | TNF | 9606.ENSP00000359663 | 9606.ENSP00000398698 | 0 | 0 | 0 | 0 | 0.118 | 0 | 0.5 | 0.88 | 0.942 |
| CDK2 | PCNA | 9606.ENSP00000266970 | 9606.ENSP00000368458 | 0 | 0 | 0 | 0 | 0.406 | 0.876 | 0 | 0.39 | 0.951 |
| CDK2 | MYC | 9606.ENSP00000266970 | 9606.ENSP00000478887 | 0 | 0 | 0 | 0 | 0.114 | 0.327 | 0.5 | 0.884 | 0.96 |
| CDK2 | TP53 | 9606.ENSP00000266970 | 9606.ENSP00000269305 | 0 | 0 | 0 | 0 | 0.093 | 0.778 | 0.5 | 0.891 | 0.987 |
| CDK2 | CDKN2A | 9606.ENSP00000266970 | 9606.ENSP00000418915 | 0 | 0 | 0 | 0 | 0.072 | 0.094 | 0 | 0.993 | 0.993 |
| CDK2 | E2F1 | 9606.ENSP00000266970 | 9606.ENSP00000345571 | 0 | 0 | 0 | 0 | 0.164 | 0.796 | 0.9 | 0.873 | 0.997 |
| CDK2 | RB1 | 9606.ENSP00000266970 | 9606.ENSP00000267163 | 0 | 0 | 0 | 0 | 0.095 | 0.991 | 0.9 | 0.821 | 0.999 |
| CDK2 | CDKN1A | 9606.ENSP00000266970 | 9606.ENSP00000384849 | 0 | 0 | 0 | 0 | 0.085 | 0.994 | 0.9 | 0.999 | 0.999 |
| CDKN1A | MDM2 | 9606.ENSP00000384849 | 9606.ENSP00000258149 | 0 | 0 | 0 | 0 | 0.072 | 0.848 | 0 | 0.873 | 0.98 |
| CDKN1A | TP53 | 9606.ENSP00000384849 | 9606.ENSP00000269305 | 0 | 0 | 0 | 0 | 0 | 0.925 | 0.9 | 0.96 | 0.999 |
| CDKN1A | HSP90AA1 | 9606.ENSP00000384849 | 9606.ENSP00000335153 | 0 | 0 | 0 | 0 | 0.078 | 0.292 | 0.5 | 0.756 | 0.909 |
| CDKN1A | E2F1 | 9606.ENSP00000384849 | 9606.ENSP00000345571 | 0 | 0 | 0 | 0 | 0 | 0.226 | 0.5 | 0.947 | 0.977 |
| CDKN1A | PCNA | 9606.ENSP00000384849 | 9606.ENSP00000368458 | 0 | 0 | 0 | 0 | 0.104 | 0.999 | 0.9 | 0.249 | 0.999 |
| CDKN1A | CDKN2A | 9606.ENSP00000384849 | 9606.ENSP00000418915 | 0 | 0 | 0 | 0 | 0.06 | 0.049 | 0.5 | 0.933 | 0.966 |
| CDKN1A | MYC | 9606.ENSP00000384849 | 9606.ENSP00000478887 | 0 | 0 | 0 | 0 | 0.105 | 0.32 | 0.5 | 0.945 | 0.981 |
| CDKN2A | MAPK14 | 9606.ENSP00000418915 | 9606.ENSP00000229795 | 0 | 0 | 0 | 0 | 0.047 | 0.216 | 0.9 | 0.34 | 0.944 |
| CDKN2A | MDM2 | 9606.ENSP00000418915 | 9606.ENSP00000258149 | 0 | 0 | 0 | 0 | 0.068 | 0.992 | 0.9 | 0.999 | 0.999 |
| CDKN2A | TP53 | 9606.ENSP00000418915 | 9606.ENSP00000269305 | 0 | 0 | 0 | 0 | 0 | 0.874 | 0.9 | 0.97 | 0.999 |
| CDKN2A | EGFR | 9606.ENSP00000418915 | 9606.ENSP00000275493 | 0 | 0 | 0 | 0 | 0.069 | 0.045 | 0 | 0.906 | 0.91 |
| CDKN2A | E2F1 | 9606.ENSP00000418915 | 9606.ENSP00000345571 | 0 | 0 | 0 | 0 | 0.067 | 0.621 | 0 | 0.921 | 0.969 |
| CDKN2A | PTEN | 9606.ENSP00000418915 | 9606.ENSP00000361021 | 0 | 0 | 0 | 0 | 0.053 | 0.188 | 0 | 0.908 | 0.923 |
| CDKN2A | CTNNB1 | 9606.ENSP00000418915 | 9606.ENSP00000495360 | 0 | 0 | 0 | 0 | 0.056 | 0.053 | 0.5 | 0.862 | 0.93 |
| CDKN2A | HIF1A | 9606.ENSP00000418915 | 9606.ENSP00000437955 | 0 | 0 | 0 | 0 | 0 | 0.426 | 0 | 0.927 | 0.956 |
| CDKN2A | MYC | 9606.ENSP00000418915 | 9606.ENSP00000478887 | 0 | 0 | 0 | 0 | 0.055 | 0.783 | 0.9 | 0.929 | 0.998 |
| CHEK1 | TP53 | 9606.ENSP00000391090 | 9606.ENSP00000269305 | 0 | 0 | 0 | 0 | 0.074 | 0.783 | 0.9 | 0.906 | 0.997 |
| CHEK1 | CHEK2 | 9606.ENSP00000391090 | 9606.ENSP00000372023 | 0 | 0 | 0 | 0.593 | 0.298 | 0.922 | 0 | 0.526 | 0.971 |
| CHEK2 | MDM2 | 9606.ENSP00000372023 | 9606.ENSP00000258149 | 0 | 0 | 0 | 0 | 0.049 | 0.625 | 0.75 | 0.837 | 0.983 |
| CHEK2 | TP53 | 9606.ENSP00000372023 | 9606.ENSP00000269305 | 0 | 0 | 0 | 0 | 0.072 | 0.833 | 0.75 | 0.992 | 0.999 |
| CHRM1 | CHRM2 | 9606.ENSP00000306490 | 9606.ENSP00000399745 | 0 | 0 | 0.059 | 0.921 | 0.053 | 0 | 0.9 | 0.095 | 0.908 |
| CHRNA2 | CHRNA7 | 9606.ENSP00000385026 | 9606.ENSP00000407546 | 0 | 0 | 0.087 | 0.879 | 0.086 | 0.091 | 0.9 | 0.229 | 0.93 |
| CHUK | NFKBIA | 9606.ENSP00000359424 | 9606.ENSP00000216797 | 0 | 0 | 0 | 0 | 0 | 0.994 | 0.9 | 0.972 | 0.999 |
| CHUK | TP53 | 9606.ENSP00000359424 | 9606.ENSP00000269305 | 0 | 0 | 0 | 0 | 0.058 | 0.345 | 0.5 | 0.756 | 0.914 |
| CHUK | HSP90AA1 | 9606.ENSP00000359424 | 9606.ENSP00000335153 | 0 | 0 | 0 | 0 | 0.09 | 0.704 | 0 | 0.972 | 0.992 |
| CHUK | PTEN | 9606.ENSP00000359424 | 9606.ENSP00000361021 | 0 | 0 | 0 | 0 | 0.089 | 0.329 | 0.9 | 0.487 | 0.964 |
| CHUK | RELA | 9606.ENSP00000359424 | 9606.ENSP00000384273 | 0 | 0 | 0 | 0 | 0.057 | 0.88 | 0.9 | 0.968 | 0.999 |
| CHUK | TNF | 9606.ENSP00000359424 | 9606.ENSP00000398698 | 0 | 0 | 0 | 0 | 0.067 | 0.994 | 0.5 | 0.837 | 0.999 |
| CHUK | IKBKB | 9606.ENSP00000359424 | 9606.ENSP00000430684 | 0 | 0 | 0.068 | 0.941 | 0.06 | 0.994 | 0.9 | 0.983 | 0.999 |
| COL3A1 | PCOLCE | 9606.ENSP00000304408 | 9606.ENSP00000223061 | 0 | 0 | 0 | 0 | 0.336 | 0.909 | 0.4 | 0.428 | 0.976 |
| CRP | F2 | 9606.ENSP00000255030 | 9606.ENSP00000308541 | 0 | 0 | 0 | 0 | 0.208 | 0 | 0 | 0.887 | 0.906 |
| CRP | TNF | 9606.ENSP00000255030 | 9606.ENSP00000398698 | 0 | 0 | 0 | 0 | 0 | 0 | 0 | 0.938 | 0.938 |
| CRP | OLR1 | 9606.ENSP00000255030 | 9606.ENSP00000309124 | 0 | 0 | 0 | 0 | 0.057 | 0 | 0 | 0.965 | 0.965 |
| CRP | IL6 | 9606.ENSP00000255030 | 9606.ENSP00000385675 | 0 | 0 | 0 | 0 | 0.047 | 0 | 0 | 0.968 | 0.968 |
| CTNNB1 | SIRT1 | 9606.ENSP00000495360 | 9606.ENSP00000212015 | 0 | 0 | 0 | 0 | 0 | 0.3 | 0 | 0.866 | 0.902 |
| CTNNB1 | KDR | 9606.ENSP00000495360 | 9606.ENSP00000263923 | 0 | 0 | 0 | 0 | 0.095 | 0 | 0.8 | 0.981 | 0.996 |
| CTNNB1 | TP53 | 9606.ENSP00000495360 | 9606.ENSP00000269305 | 0 | 0 | 0 | 0 | 0.049 | 0 | 0 | 0.927 | 0.927 |
| CTNNB1 | ERBB2 | 9606.ENSP00000495360 | 9606.ENSP00000269571 | 0 | 0 | 0 | 0 | 0 | 0.662 | 0.9 | 0.954 | 0.998 |
| CTNNB1 | EGFR | 9606.ENSP00000495360 | 9606.ENSP00000275493 | 0 | 0 | 0 | 0 | 0.064 | 0.718 | 0.9 | 0.94 | 0.998 |
| CTNNB1 | PPARG | 9606.ENSP00000495360 | 9606.ENSP00000287820 | 0 | 0 | 0 | 0 | 0.052 | 0.735 | 0 | 0.98 | 0.994 |
| CTNNB1 | MET | 9606.ENSP00000495360 | 9606.ENSP00000317272 | 0 | 0 | 0 | 0 | 0.042 | 0.364 | 0.9 | 0.792 | 0.985 |
| CTNNB1 | GSK3B | 9606.ENSP00000495360 | 9606.ENSP00000324806 | 0 | 0 | 0 | 0 | 0.054 | 0.991 | 0.9 | 0.999 | 0.999 |
| CTNNB1 | PTPN1 | 9606.ENSP00000495360 | 9606.ENSP00000360683 | 0 | 0 | 0 | 0 | 0.053 | 0.297 | 0.9 | 0.612 | 0.97 |
| CTNNB1 | PTEN | 9606.ENSP00000495360 | 9606.ENSP00000361021 | 0 | 0 | 0 | 0 | 0.069 | 0.527 | 0 | 0.881 | 0.943 |
| CTNNB1 | RELA | 9606.ENSP00000495360 | 9606.ENSP00000384273 | 0 | 0 | 0 | 0 | 0.044 | 0.535 | 0 | 0.835 | 0.92 |
| CTNNB1 | ESR1 | 9606.ENSP00000495360 | 9606.ENSP00000405330 | 0 | 0 | 0 | 0 | 0.052 | 0.301 | 0 | 0.952 | 0.965 |
| CTNNB1 | HIF1A | 9606.ENSP00000495360 | 9606.ENSP00000437955 | 0 | 0 | 0 | 0 | 0.26 | 0.785 | 0 | 0.989 | 0.998 |
| CTNNB1 | MYC | 9606.ENSP00000495360 | 9606.ENSP00000478887 | 0 | 0 | 0 | 0 | 0.058 | 0.567 | 0.5 | 0.937 | 0.985 |
| CXCL10 | IFNG | 9606.ENSP00000305651 | 9606.ENSP00000229135 | 0 | 0 | 0 | 0 | 0.321 | 0 | 0 | 0.942 | 0.959 |
| CXCL10 | IL1A | 9606.ENSP00000305651 | 9606.ENSP00000263339 | 0 | 0 | 0 | 0 | 0.146 | 0 | 0.4 | 0.925 | 0.958 |
| CXCL10 | IL1B | 9606.ENSP00000305651 | 9606.ENSP00000263341 | 0 | 0 | 0 | 0 | 0.229 | 0 | 0.4 | 0.925 | 0.962 |
| CXCL10 | STAT1 | 9606.ENSP00000305651 | 9606.ENSP00000354394 | 0 | 0 | 0 | 0 | 0.557 | 0 | 0 | 0.89 | 0.949 |
| CXCL10 | IL6 | 9606.ENSP00000305651 | 9606.ENSP00000385675 | 0 | 0 | 0 | 0 | 0.203 | 0 | 0.4 | 0.923 | 0.96 |
| CXCL10 | IL10 | 9606.ENSP00000305651 | 9606.ENSP00000412237 | 0 | 0 | 0 | 0 | 0.225 | 0 | 0.4 | 0.925 | 0.962 |
| CXCL10 | CXCL2 | 9606.ENSP00000305651 | 9606.ENSP00000427279 | 0 | 0 | 0 | 0 | 0.172 | 0 | 0.5 | 0.922 | 0.965 |
| CXCL10 | TNF | 9606.ENSP00000305651 | 9606.ENSP00000398698 | 0 | 0 | 0 | 0 | 0.265 | 0 | 0.4 | 0.93 | 0.966 |
| CXCL10 | CXCL8 | 9606.ENSP00000305651 | 9606.ENSP00000306512 | 0 | 0 | 0 | 0 | 0.124 | 0 | 0.5 | 0.948 | 0.975 |
| CXCL10 | CXCL11 | 9606.ENSP00000305651 | 9606.ENSP00000306884 | 0 | 0 | 0 | 0.811 | 0.824 | 0.994 | 0.9 | 0.467 | 0.999 |
| CXCL11 | TGFB1 | 9606.ENSP00000306884 | 9606.ENSP00000221930 | 0 | 0 | 0 | 0 | 0 | 0 | 0 | 0.947 | 0.947 |
| CXCL11 | CXCL8 | 9606.ENSP00000306884 | 9606.ENSP00000306512 | 0 | 0 | 0 | 0 | 0.083 | 0 | 0.5 | 0.893 | 0.946 |
| CXCL11 | CXCL2 | 9606.ENSP00000306884 | 9606.ENSP00000427279 | 0 | 0 | 0 | 0 | 0.107 | 0.994 | 0.5 | 0.893 | 0.999 |
| CXCL2 | IL1A | 9606.ENSP00000427279 | 9606.ENSP00000263339 | 0 | 0 | 0 | 0 | 0.328 | 0 | 0.4 | 0.87 | 0.943 |
| CXCL2 | IL1B | 9606.ENSP00000427279 | 9606.ENSP00000263341 | 0 | 0 | 0 | 0 | 0.522 | 0 | 0.4 | 0.898 | 0.968 |
| CXCL2 | CXCL8 | 9606.ENSP00000427279 | 9606.ENSP00000306512 | 0 | 0 | 0.062 | 0.87 | 0.794 | 0 | 0.4 | 0.866 | 0.982 |
| CXCL2 | JUN | 9606.ENSP00000427279 | 9606.ENSP00000360266 | 0 | 0 | 0 | 0 | 0.25 | 0 | 0.9 | 0.431 | 0.953 |
| CXCL2 | RELA | 9606.ENSP00000427279 | 9606.ENSP00000384273 | 0 | 0 | 0 | 0 | 0.055 | 0.292 | 0.9 | 0.38 | 0.953 |
| CXCL2 | IL6 | 9606.ENSP00000427279 | 9606.ENSP00000385675 | 0 | 0 | 0 | 0 | 0.639 | 0 | 0.4 | 0.855 | 0.966 |
| CXCL2 | TNF | 9606.ENSP00000427279 | 9606.ENSP00000398698 | 0 | 0 | 0 | 0 | 0.292 | 0 | 0.4 | 0.844 | 0.928 |
| CXCL8 | IL2 | 9606.ENSP00000306512 | 9606.ENSP00000226730 | 0 | 0 | 0 | 0 | 0.048 | 0 | 0 | 0.985 | 0.985 |
| CXCL8 | IFNG | 9606.ENSP00000306512 | 9606.ENSP00000229135 | 0 | 0 | 0.281 | 0 | 0.152 | 0 | 0 | 0.942 | 0.961 |
| CXCL8 | IL4 | 9606.ENSP00000306512 | 9606.ENSP00000231449 | 0 | 0 | 0 | 0 | 0.049 | 0 | 0.4 | 0.996 | 0.997 |
| CXCL8 | IL1A | 9606.ENSP00000306512 | 9606.ENSP00000263339 | 0 | 0 | 0 | 0 | 0.451 | 0 | 0.4 | 0.928 | 0.974 |
| CXCL8 | IL1B | 9606.ENSP00000306512 | 9606.ENSP00000263341 | 0 | 0 | 0 | 0 | 0.776 | 0 | 0.4 | 0.962 | 0.994 |
| CXCL8 | ICAM1 | 9606.ENSP00000306512 | 9606.ENSP00000264832 | 0 | 0 | 0 | 0 | 0.259 | 0 | 0 | 0.927 | 0.943 |
| CXCL8 | IL10 | 9606.ENSP00000306512 | 9606.ENSP00000412237 | 0 | 0 | 0 | 0 | 0.147 | 0 | 0.4 | 0.946 | 0.97 |
| CXCL8 | PTGS2 | 9606.ENSP00000306512 | 9606.ENSP00000356438 | 0 | 0 | 0 | 0 | 0.767 | 0 | 0 | 0.901 | 0.975 |
| CXCL8 | TNF | 9606.ENSP00000306512 | 9606.ENSP00000398698 | 0 | 0 | 0 | 0 | 0.294 | 0 | 0.4 | 0.965 | 0.983 |
| CXCL8 | JUN | 9606.ENSP00000306512 | 9606.ENSP00000360266 | 0 | 0 | 0 | 0 | 0.203 | 0.292 | 0.9 | 0.802 | 0.987 |
| CXCL8 | IL6 | 9606.ENSP00000306512 | 9606.ENSP00000385675 | 0 | 0 | 0 | 0 | 0.445 | 0 | 0.4 | 0.976 | 0.991 |
| CXCL8 | RELA | 9606.ENSP00000306512 | 9606.ENSP00000384273 | 0 | 0 | 0 | 0 | 0.062 | 0.785 | 0.9 | 0.82 | 0.995 |
| CYCS | HSPB1 | 9606.ENSP00000307786 | 9606.ENSP00000248553 | 0 | 0 | 0 | 0 | 0.056 | 0.292 | 0 | 0.99 | 0.993 |
| CYCS | TP53 | 9606.ENSP00000307786 | 9606.ENSP00000269305 | 0 | 0 | 0 | 0 | 0 | 0 | 0 | 0.911 | 0.911 |
| CYCS | XIAP | 9606.ENSP00000307786 | 9606.ENSP00000360242 | 0 | 0 | 0 | 0 | 0.055 | 0 | 0.5 | 0.873 | 0.935 |
| CYP1A1 | GSTM1 | 9606.ENSP00000378488 | 9606.ENSP00000311469 | 0 | 0 | 0 | 0 | 0.067 | 0.045 | 0.9 | 0.918 | 0.991 |
| CYP1A1 | CYP2B6 | 9606.ENSP00000378488 | 9606.ENSP00000324648 | 0 | 0 | 0.142 | 0.794 | 0.058 | 0 | 0.91 | 0.497 | 0.959 |
| CYP1A1 | CYP1B1 | 9606.ENSP00000378488 | 9606.ENSP00000478561 | 0 | 0 | 0.095 | 0.889 | 0.135 | 0 | 0.91 | 0.177 | 0.937 |
| CYP1A1 | GSTA2 | 9606.ENSP00000378488 | 9606.ENSP00000420168 | 0 | 0 | 0 | 0 | 0.067 | 0.045 | 0.9 | 0.49 | 0.948 |
| CYP1A1 | CYP3A4 | 9606.ENSP00000378488 | 9606.ENSP00000498939 | 0 | 0 | 0.233 | 0.604 | 0.117 | 0.076 | 0.91 | 0.81 | 0.987 |
| CYP1B1 | GSTM1 | 9606.ENSP00000478561 | 9606.ENSP00000311469 | 0 | 0 | 0 | 0 | 0.067 | 0.045 | 0.9 | 0.625 | 0.962 |
| CYP1B1 | CYP2B6 | 9606.ENSP00000478561 | 9606.ENSP00000324648 | 0 | 0 | 0.136 | 0.804 | 0 | 0 | 0.91 | 0.184 | 0.932 |
| CYP1B1 | GSTA2 | 9606.ENSP00000478561 | 9606.ENSP00000420168 | 0 | 0 | 0 | 0 | 0.067 | 0.045 | 0.9 | 0.376 | 0.936 |
| CYP2B6 | PTGS2 | 9606.ENSP00000324648 | 9606.ENSP00000356438 | 0 | 0 | 0 | 0 | 0.056 | 0 | 0.9 | 0.198 | 0.917 |
| CYP2B6 | PTGS1 | 9606.ENSP00000324648 | 9606.ENSP00000354612 | 0 | 0 | 0 | 0 | 0.056 | 0 | 0.9 | 0.208 | 0.918 |
| CYP2B6 | CYP3A4 | 9606.ENSP00000324648 | 9606.ENSP00000498939 | 0 | 0 | 0.221 | 0.634 | 0.132 | 0.076 | 0.91 | 0.411 | 0.96 |
| CYP3A4 | GSTM1 | 9606.ENSP00000498939 | 9606.ENSP00000311469 | 0 | 0 | 0 | 0 | 0.093 | 0 | 0.9 | 0.574 | 0.958 |
| CYP3A4 | NR1I2 | 9606.ENSP00000498939 | 9606.ENSP00000336528 | 0 | 0 | 0 | 0 | 0.162 | 0.071 | 0 | 0.955 | 0.962 |
| CYP3A4 | MAOA | 9606.ENSP00000498939 | 9606.ENSP00000340684 | 0 | 0 | 0 | 0 | 0.086 | 0 | 0.9 | 0.536 | 0.953 |
| CYP3A4 | MAOB | 9606.ENSP00000498939 | 9606.ENSP00000367309 | 0 | 0 | 0 | 0 | 0.089 | 0 | 0.9 | 0.53 | 0.953 |
| CYP3A4 | GSTA2 | 9606.ENSP00000498939 | 9606.ENSP00000420168 | 0 | 0 | 0 | 0 | 0.149 | 0 | 0.9 | 0.378 | 0.942 |
| DRD1 | SLC6A3 | 9606.ENSP00000377353 | 9606.ENSP00000270349 | 0 | 0 | 0 | 0 | 0 | 0 | 0 | 0.906 | 0.906 |
| DUOX2 | NCF1 | 9606.ENSP00000373691 | 9606.ENSP00000289473 | 0 | 0 | 0 | 0 | 0.075 | 0 | 0.54 | 0.814 | 0.914 |
| E2F1 | SIRT1 | 9606.ENSP00000345571 | 9606.ENSP00000212015 | 0 | 0 | 0 | 0 | 0 | 0.625 | 0 | 0.806 | 0.924 |
| E2F1 | MDM2 | 9606.ENSP00000345571 | 9606.ENSP00000258149 | 0 | 0 | 0 | 0 | 0 | 0.625 | 0 | 0.98 | 0.992 |
| E2F1 | RB1 | 9606.ENSP00000345571 | 9606.ENSP00000267163 | 0 | 0 | 0 | 0 | 0.067 | 0.999 | 0.9 | 0.995 | 0.999 |
| E2F1 | TP53 | 9606.ENSP00000345571 | 9606.ENSP00000269305 | 0 | 0 | 0 | 0 | 0 | 0.51 | 0 | 0.974 | 0.986 |
| E2F1 | MYC | 9606.ENSP00000345571 | 9606.ENSP00000478887 | 0 | 0 | 0 | 0 | 0.084 | 0.166 | 0.5 | 0.99 | 0.995 |
| EGFR | TGFB1 | 9606.ENSP00000275493 | 9606.ENSP00000221930 | 0 | 0 | 0 | 0 | 0 | 0.152 | 0 | 0.972 | 0.975 |
| EGFR | TP53 | 9606.ENSP00000275493 | 9606.ENSP00000269305 | 0 | 0 | 0 | 0 | 0 | 0.329 | 0 | 0.919 | 0.943 |
| EGFR | ERBB2 | 9606.ENSP00000275493 | 9606.ENSP00000269571 | 0 | 0 | 0.064 | 0.939 | 0.08 | 0.998 | 0.9 | 0.983 | 0.999 |
| EGFR | RASA1 | 9606.ENSP00000275493 | 9606.ENSP00000274376 | 0 | 0 | 0 | 0 | 0.042 | 0.716 | 0 | 0.901 | 0.97 |
| EGFR | PRKCA | 9606.ENSP00000275493 | 9606.ENSP00000408695 | 0 | 0 | 0 | 0.562 | 0.049 | 0.788 | 0 | 0.693 | 0.932 |
| EGFR | HIF1A | 9606.ENSP00000275493 | 9606.ENSP00000437955 | 0 | 0 | 0 | 0 | 0.055 | 0 | 0.5 | 0.873 | 0.934 |
| EGFR | FOS | 9606.ENSP00000275493 | 9606.ENSP00000306245 | 0 | 0 | 0 | 0 | 0.086 | 0.591 | 0.4 | 0.765 | 0.94 |
| EGFR | PTEN | 9606.ENSP00000275493 | 9606.ENSP00000361021 | 0 | 0 | 0 | 0 | 0 | 0.608 | 0 | 0.925 | 0.969 |
| EGFR | STAT1 | 9606.ENSP00000275493 | 9606.ENSP00000354394 | 0 | 0 | 0 | 0 | 0.042 | 0.84 | 0 | 0.851 | 0.975 |
| EGFR | IL6 | 9606.ENSP00000275493 | 9606.ENSP00000385675 | 0 | 0 | 0 | 0 | 0.042 | 0 | 0.65 | 0.943 | 0.979 |
| EGFR | IGFBP3 | 9606.ENSP00000275493 | 9606.ENSP00000370473 | 0 | 0 | 0 | 0 | 0.169 | 0.292 | 0 | 0.971 | 0.981 |
| EGFR | ESR1 | 9606.ENSP00000275493 | 9606.ENSP00000405330 | 0 | 0 | 0 | 0 | 0 | 0.583 | 0 | 0.974 | 0.988 |
| EGFR | MET | 9606.ENSP00000275493 | 9606.ENSP00000317272 | 0 | 0 | 0 | 0.582 | 0.25 | 0.806 | 0 | 0.978 | 0.996 |
| EGFR | PTPN1 | 9606.ENSP00000275493 | 9606.ENSP00000360683 | 0 | 0 | 0 | 0 | 0.044 | 0.869 | 0 | 0.978 | 0.997 |
| EGFR | HSP90AA1 | 9606.ENSP00000275493 | 9606.ENSP00000335153 | 0 | 0 | 0 | 0 | 0.049 | 0.854 | 0.5 | 0.993 | 0.999 |
| EGLN1 | PRKCB | 9606.ENSP00000355601 | 9606.ENSP00000496129 | 0 | 0 | 0 | 0 | 0.056 | 0 | 0.9 | 0.075 | 0.905 |
| EGLN1 | PRKCA | 9606.ENSP00000355601 | 9606.ENSP00000408695 | 0 | 0 | 0 | 0 | 0.067 | 0 | 0.9 | 0.13 | 0.911 |
| EGLN1 | HIF1A | 9606.ENSP00000355601 | 9606.ENSP00000437955 | 0 | 0 | 0 | 0 | 0.05 | 0.968 | 0.9 | 0.981 | 0.999 |
| ELK1 | MAPK1 | 9606.ENSP00000366182 | 9606.ENSP00000215832 | 0 | 0 | 0 | 0 | 0.049 | 0.927 | 0.9 | 0.263 | 0.994 |
| ELK1 | MAPK14 | 9606.ENSP00000366182 | 9606.ENSP00000229795 | 0 | 0 | 0 | 0 | 0.049 | 0.094 | 0.9 | 0.074 | 0.909 |
| ELK1 | PRKCB | 9606.ENSP00000366182 | 9606.ENSP00000496129 | 0 | 0 | 0 | 0 | 0.064 | 0 | 0.9 | 0 | 0.902 |
| ELK1 | PRKCA | 9606.ENSP00000366182 | 9606.ENSP00000408695 | 0 | 0 | 0 | 0 | 0.094 | 0 | 0.9 | 0.108 | 0.912 |
| ELK1 | MAPK8 | 9606.ENSP00000366182 | 9606.ENSP00000378974 | 0 | 0 | 0 | 0 | 0.111 | 0.722 | 0.9 | 0.143 | 0.976 |
| ERBB2 | TP53 | 9606.ENSP00000269571 | 9606.ENSP00000269305 | 0 | 0 | 0 | 0 | 0.06 | 0.172 | 0 | 0.924 | 0.935 |
| ERBB2 | ESR1 | 9606.ENSP00000269571 | 9606.ENSP00000405330 | 0 | 0 | 0 | 0 | 0 | 0.329 | 0 | 0.966 | 0.976 |
| ERBB2 | PGR | 9606.ENSP00000269571 | 9606.ENSP00000325120 | 0 | 0 | 0 | 0 | 0 | 0.329 | 0 | 0.972 | 0.98 |
| ERBB2 | HSP90AA1 | 9606.ENSP00000269571 | 9606.ENSP00000335153 | 0 | 0 | 0 | 0 | 0 | 0.898 | 0.5 | 0.985 | 0.999 |
| ESR1 | MAPK1 | 9606.ENSP00000405330 | 9606.ENSP00000215832 | 0 | 0 | 0 | 0 | 0 | 0.625 | 0.9 | 0.681 | 0.987 |
| ESR1 | MAPK14 | 9606.ENSP00000405330 | 9606.ENSP00000229795 | 0 | 0 | 0 | 0 | 0 | 0 | 0.9 | 0.561 | 0.954 |
| ESR1 | MDM2 | 9606.ENSP00000405330 | 9606.ENSP00000258149 | 0 | 0 | 0 | 0 | 0.042 | 0.785 | 0 | 0.762 | 0.946 |
| ESR1 | TP53 | 9606.ENSP00000405330 | 9606.ENSP00000269305 | 0 | 0 | 0 | 0 | 0 | 0.735 | 0 | 0.974 | 0.992 |
| ESR1 | FOS | 9606.ENSP00000405330 | 9606.ENSP00000306245 | 0 | 0 | 0 | 0 | 0.044 | 0.522 | 0.9 | 0.994 | 0.999 |
| ESR1 | PGR | 9606.ENSP00000405330 | 9606.ENSP00000325120 | 0 | 0 | 0 | 0.673 | 0.24 | 0.771 | 0.7 | 0.56 | 0.974 |
| ESR1 | HSP90AA1 | 9606.ENSP00000405330 | 9606.ENSP00000335153 | 0 | 0 | 0 | 0 | 0.054 | 0.889 | 0.5 | 0.988 | 0.999 |
| ESR1 | ESR2 | 9606.ENSP00000405330 | 9606.ENSP00000343925 | 0 | 0 | 0.062 | 0.923 | 0.042 | 0.83 | 0.9 | 0.934 | 0.998 |
| ESR1 | STAT1 | 9606.ENSP00000405330 | 9606.ENSP00000354394 | 0 | 0 | 0 | 0 | 0.043 | 0.304 | 0.5 | 0.775 | 0.915 |
| ESR1 | JUN | 9606.ENSP00000405330 | 9606.ENSP00000360266 | 0 | 0 | 0 | 0 | 0 | 0.631 | 0.9 | 0.995 | 0.999 |
| ESR1 | RUNX2 | 9606.ENSP00000405330 | 9606.ENSP00000360493 | 0 | 0 | 0 | 0 | 0.056 | 0.071 | 0.75 | 0.77 | 0.942 |
| ESR1 | MAPK8 | 9606.ENSP00000405330 | 9606.ENSP00000378974 | 0 | 0 | 0 | 0 | 0 | 0 | 0.9 | 0.659 | 0.964 |
| ESR1 | RELA | 9606.ENSP00000405330 | 9606.ENSP00000384273 | 0 | 0 | 0 | 0 | 0 | 0.787 | 0 | 0.904 | 0.978 |
| ESR1 | NCOA1 | 9606.ENSP00000405330 | 9606.ENSP00000385216 | 0 | 0 | 0 | 0 | 0 | 0.997 | 0.8 | 0.986 | 0.999 |
| ESR1 | HIF1A | 9606.ENSP00000405330 | 9606.ENSP00000437955 | 0 | 0 | 0 | 0 | 0.067 | 0.636 | 0 | 0.861 | 0.948 |
| ESR1 | MYC | 9606.ENSP00000405330 | 9606.ENSP00000478887 | 0 | 0 | 0 | 0 | 0.053 | 0.62 | 0.5 | 0.913 | 0.982 |
| ESR2 | MAPK1 | 9606.ENSP00000343925 | 9606.ENSP00000215832 | 0 | 0 | 0 | 0 | 0 | 0.292 | 0.9 | 0.332 | 0.948 |
| ESR2 | MAPK14 | 9606.ENSP00000343925 | 9606.ENSP00000229795 | 0 | 0 | 0 | 0 | 0 | 0 | 0.9 | 0.247 | 0.921 |
| ESR2 | FOS | 9606.ENSP00000343925 | 9606.ENSP00000306245 | 0 | 0 | 0 | 0 | 0 | 0.095 | 0.9 | 0.611 | 0.961 |
| ESR2 | MAPK8 | 9606.ENSP00000343925 | 9606.ENSP00000378974 | 0 | 0 | 0 | 0 | 0 | 0 | 0.9 | 0.269 | 0.923 |
| ESR2 | JUN | 9606.ENSP00000343925 | 9606.ENSP00000360266 | 0 | 0 | 0 | 0 | 0 | 0.057 | 0.9 | 0.686 | 0.967 |
| ESR2 | NCOA1 | 9606.ENSP00000343925 | 9606.ENSP00000385216 | 0 | 0 | 0 | 0 | 0 | 0.984 | 0.8 | 0.946 | 0.999 |
| F10 | F2 | 9606.ENSP00000364709 | 9606.ENSP00000308541 | 0 | 0 | 0.125 | 0.755 | 0.209 | 0.93 | 0.75 | 0.972 | 0.999 |
| F10 | F3 | 9606.ENSP00000364709 | 9606.ENSP00000334145 | 0 | 0 | 0 | 0 | 0 | 0 | 0.7 | 0.998 | 0.999 |
| F10 | F7 | 9606.ENSP00000364709 | 9606.ENSP00000364731 | 0 | 0 | 0.093 | 0.894 | 0.408 | 0.292 | 0.9 | 0.744 | 0.988 |
| F2 | F7 | 9606.ENSP00000308541 | 9606.ENSP00000364731 | 0 | 0 | 0.147 | 0.698 | 0.192 | 0 | 0.54 | 0.794 | 0.926 |
| F2 | F3 | 9606.ENSP00000308541 | 9606.ENSP00000334145 | 0 | 0 | 0 | 0 | 0 | 0 | 0.54 | 0.993 | 0.996 |
| F2 | THBD | 9606.ENSP00000308541 | 9606.ENSP00000366307 | 0 | 0 | 0 | 0 | 0 | 0.967 | 0.9 | 0.927 | 0.999 |
| F3 | PLAT | 9606.ENSP00000334145 | 9606.ENSP00000220809 | 0 | 0 | 0 | 0 | 0.169 | 0 | 0 | 0.922 | 0.932 |
| F3 | SERPINE1 | 9606.ENSP00000334145 | 9606.ENSP00000223095 | 0 | 0 | 0 | 0 | 0.168 | 0 | 0 | 0.887 | 0.902 |
| F3 | THBD | 9606.ENSP00000334145 | 9606.ENSP00000366307 | 0 | 0 | 0 | 0 | 0.075 | 0 | 0.54 | 0.963 | 0.982 |
| F3 | F7 | 9606.ENSP00000334145 | 9606.ENSP00000364731 | 0 | 0 | 0 | 0 | 0 | 0.987 | 0.9 | 0.999 | 0.999 |
| F7 | THBD | 9606.ENSP00000364731 | 9606.ENSP00000366307 | 0 | 0 | 0 | 0 | 0 | 0.094 | 0.54 | 0.872 | 0.942 |
| FABP5 | PPARD | 9606.ENSP00000297258 | 9606.ENSP00000310928 | 0 | 0 | 0 | 0 | 0.044 | 0.05 | 0.5 | 0.977 | 0.988 |
| FOS | MAPK1 | 9606.ENSP00000306245 | 9606.ENSP00000215832 | 0 | 0 | 0 | 0 | 0.055 | 0.45 | 0.9 | 0.864 | 0.992 |
| FOS | MAPK14 | 9606.ENSP00000306245 | 9606.ENSP00000229795 | 0 | 0 | 0 | 0 | 0.067 | 0.256 | 0.9 | 0.814 | 0.985 |
| FOS | NR3C1 | 9606.ENSP00000306245 | 9606.ENSP00000231509 | 0 | 0 | 0 | 0 | 0 | 0.108 | 0 | 0.958 | 0.961 |
| FOS | TP53 | 9606.ENSP00000306245 | 9606.ENSP00000269305 | 0 | 0 | 0 | 0 | 0.053 | 0.23 | 0.5 | 0.884 | 0.952 |
| FOS | RELA | 9606.ENSP00000306245 | 9606.ENSP00000384273 | 0 | 0 | 0 | 0 | 0.101 | 0.528 | 0 | 0.82 | 0.917 |
| FOS | PRKCB | 9606.ENSP00000306245 | 9606.ENSP00000496129 | 0 | 0 | 0 | 0 | 0.056 | 0 | 0.9 | 0.319 | 0.93 |
| FOS | NFATC1 | 9606.ENSP00000306245 | 9606.ENSP00000389377 | 0 | 0 | 0 | 0 | 0.047 | 0.233 | 0 | 0.927 | 0.942 |
| FOS | PRKCA | 9606.ENSP00000306245 | 9606.ENSP00000408695 | 0 | 0 | 0 | 0 | 0.049 | 0 | 0.9 | 0.48 | 0.946 |
| FOS | NCOA1 | 9606.ENSP00000306245 | 9606.ENSP00000385216 | 0 | 0 | 0 | 0 | 0.049 | 0.643 | 0.8 | 0.34 | 0.949 |
| FOS | MYC | 9606.ENSP00000306245 | 9606.ENSP00000478887 | 0 | 0 | 0 | 0 | 0.134 | 0.221 | 0.4 | 0.924 | 0.965 |
| FOS | MAPK8 | 9606.ENSP00000306245 | 9606.ENSP00000378974 | 0 | 0 | 0 | 0 | 0 | 0.174 | 0.9 | 0.849 | 0.986 |
| FOS | RUNX2 | 9606.ENSP00000306245 | 9606.ENSP00000360493 | 0 | 0 | 0 | 0 | 0.052 | 0.51 | 0.9 | 0.761 | 0.987 |
| FOS | JUN | 9606.ENSP00000306245 | 9606.ENSP00000360266 | 0 | 0 | 0 | 0 | 0.69 | 0.999 | 0.9 | 0.999 | 0.999 |
| GJA1 | MAPK1 | 9606.ENSP00000282561 | 9606.ENSP00000215832 | 0 | 0 | 0 | 0 | 0 | 0.292 | 0.9 | 0.269 | 0.943 |
| GSK3B | TP53 | 9606.ENSP00000324806 | 9606.ENSP00000269305 | 0 | 0 | 0 | 0 | 0 | 0.633 | 0 | 0.981 | 0.992 |
| GSK3B | HSF1 | 9606.ENSP00000324806 | 9606.ENSP00000431512 | 0 | 0 | 0 | 0 | 0.047 | 0.516 | 0.75 | 0.268 | 0.904 |
| GSK3B | PTEN | 9606.ENSP00000324806 | 9606.ENSP00000361021 | 0 | 0 | 0 | 0 | 0.056 | 0.092 | 0 | 0.899 | 0.906 |
| GSK3B | JUN | 9606.ENSP00000324806 | 9606.ENSP00000360266 | 0 | 0 | 0 | 0 | 0.059 | 0.697 | 0 | 0.704 | 0.908 |
| GSK3B | RAF1 | 9606.ENSP00000324806 | 9606.ENSP00000401888 | 0 | 0 | 0 | 0.575 | 0.061 | 0 | 0.9 | 0.241 | 0.922 |
| GSK3B | NFE2L2 | 9606.ENSP00000324806 | 9606.ENSP00000380252 | 0 | 0 | 0 | 0 | 0.049 | 0.547 | 0.75 | 0.62 | 0.953 |
| GSK3B | NFATC1 | 9606.ENSP00000324806 | 9606.ENSP00000389377 | 0 | 0 | 0 | 0 | 0 | 0.166 | 0.9 | 0.481 | 0.953 |
| GSK3B | PPP3CA | 9606.ENSP00000324806 | 9606.ENSP00000378323 | 0 | 0 | 0 | 0 | 0.062 | 0.403 | 0.9 | 0.306 | 0.955 |
| GSK3B | HSP90AA1 | 9606.ENSP00000324806 | 9606.ENSP00000335153 | 0 | 0 | 0 | 0 | 0.054 | 0.639 | 0 | 0.887 | 0.958 |
| GSK3B | MYC | 9606.ENSP00000324806 | 9606.ENSP00000478887 | 0 | 0 | 0 | 0 | 0 | 0.822 | 0.9 | 0.839 | 0.996 |
| GSTM1 | SPP1 | 9606.ENSP00000311469 | 9606.ENSP00000378517 | 0 | 0 | 0 | 0 | 0 | 0 | 0 | 0.981 | 0.981 |
| HIF1A | SIRT1 | 9606.ENSP00000437955 | 9606.ENSP00000212015 | 0 | 0 | 0 | 0 | 0.083 | 0.51 | 0 | 0.851 | 0.927 |
| HIF1A | MAPK1 | 9606.ENSP00000437955 | 9606.ENSP00000215832 | 0 | 0 | 0 | 0 | 0.049 | 0.626 | 0.9 | 0.648 | 0.985 |
| HIF1A | MDM2 | 9606.ENSP00000437955 | 9606.ENSP00000258149 | 0 | 0 | 0 | 0 | 0.06 | 0.839 | 0 | 0.929 | 0.988 |
| HIF1A | TP53 | 9606.ENSP00000437955 | 9606.ENSP00000269305 | 0 | 0 | 0 | 0 | 0 | 0.847 | 0 | 0.994 | 0.999 |
| HIF1A | HSP90AA1 | 9606.ENSP00000437955 | 9606.ENSP00000335153 | 0 | 0 | 0 | 0 | 0.131 | 0.923 | 0 | 0.994 | 0.999 |
| HIF1A | JUN | 9606.ENSP00000437955 | 9606.ENSP00000360266 | 0 | 0 | 0 | 0 | 0.056 | 0.621 | 0 | 0.901 | 0.961 |
| HIF1A | RELA | 9606.ENSP00000437955 | 9606.ENSP00000384273 | 0 | 0 | 0 | 0 | 0.079 | 0.292 | 0 | 0.985 | 0.989 |
| HIF1A | MYC | 9606.ENSP00000437955 | 9606.ENSP00000478887 | 0 | 0 | 0 | 0 | 0.091 | 0.435 | 0.4 | 0.982 | 0.993 |
| HK2 | MGAM | 9606.ENSP00000290573 | 9606.ENSP00000447378 | 0 | 0 | 0 | 0 | 0.056 | 0 | 0.91 | 0.129 | 0.922 |
| HMOX1 | NQO1 | 9606.ENSP00000216117 | 9606.ENSP00000319788 | 0 | 0 | 0 | 0 | 0.061 | 0 | 0 | 0.914 | 0.916 |
| HMOX1 | NFE2L2 | 9606.ENSP00000216117 | 9606.ENSP00000380252 | 0 | 0 | 0 | 0 | 0.05 | 0 | 0.75 | 0.96 | 0.989 |
| HSF1 | HSP90AA1 | 9606.ENSP00000431512 | 9606.ENSP00000335153 | 0 | 0 | 0 | 0 | 0.049 | 0.87 | 0.7 | 0.956 | 0.998 |
| HSP90AA1 | MAPK1 | 9606.ENSP00000335153 | 9606.ENSP00000215832 | 0 | 0 | 0 | 0 | 0.057 | 0.113 | 0.4 | 0.897 | 0.941 |
| HSP90AA1 | NR3C1 | 9606.ENSP00000335153 | 9606.ENSP00000231509 | 0 | 0 | 0 | 0 | 0.054 | 0.994 | 0.5 | 0.996 | 0.999 |
| HSP90AA1 | HSPB1 | 9606.ENSP00000335153 | 9606.ENSP00000248553 | 0 | 0 | 0 | 0 | 0.274 | 0.292 | 0 | 0.924 | 0.957 |
| HSP90AA1 | MDM2 | 9606.ENSP00000335153 | 9606.ENSP00000258149 | 0 | 0 | 0 | 0 | 0.049 | 0.539 | 0 | 0.81 | 0.909 |
| HSP90AA1 | KCNH2 | 9606.ENSP00000335153 | 9606.ENSP00000262186 | 0 | 0 | 0 | 0 | 0 | 0.735 | 0 | 0.851 | 0.958 |
| HSP90AA1 | KDR | 9606.ENSP00000335153 | 9606.ENSP00000263923 | 0 | 0 | 0 | 0 | 0 | 0.313 | 0.5 | 0.878 | 0.954 |
| HSP90AA1 | TP53 | 9606.ENSP00000335153 | 9606.ENSP00000269305 | 0 | 0 | 0 | 0 | 0 | 0.903 | 0 | 0.995 | 0.999 |
| HSP90AA1 | NOS3 | 9606.ENSP00000335153 | 9606.ENSP00000297494 | 0 | 0 | 0 | 0 | 0 | 0.789 | 0.8 | 0.992 | 0.999 |
| HSP90AA1 | HSPA5 | 9606.ENSP00000335153 | 9606.ENSP00000324173 | 0 | 0.001 | 0 | 0 | 0.379 | 0.462 | 0 | 0.912 | 0.968 |
| HSP90AA1 | PGR | 9606.ENSP00000335153 | 9606.ENSP00000325120 | 0 | 0 | 0 | 0 | 0.054 | 0.582 | 0.5 | 0.956 | 0.99 |
| HSP90AA1 | MYC | 9606.ENSP00000335153 | 9606.ENSP00000478887 | 0 | 0 | 0 | 0 | 0.062 | 0.606 | 0 | 0.939 | 0.975 |
| HSP90AA1 | IKBKB | 9606.ENSP00000335153 | 9606.ENSP00000430684 | 0 | 0 | 0 | 0 | 0.06 | 0.643 | 0 | 0.933 | 0.975 |
| HSP90AA1 | RAF1 | 9606.ENSP00000335153 | 9606.ENSP00000401888 | 0 | 0 | 0 | 0 | 0.054 | 0.941 | 0 | 0.766 | 0.985 |
| HSPA5 | SOD1 | 9606.ENSP00000324173 | 9606.ENSP00000270142 | 0 | 0 | 0 | 0 | 0.093 | 0.236 | 0.9 | 0.691 | 0.975 |
| HSPB1 | MAPK14 | 9606.ENSP00000248553 | 9606.ENSP00000229795 | 0 | 0 | 0 | 0 | 0.085 | 0.316 | 0.5 | 0.747 | 0.91 |
| HSPB1 | TP53 | 9606.ENSP00000248553 | 9606.ENSP00000269305 | 0 | 0 | 0 | 0 | 0 | 0.776 | 0 | 0.809 | 0.955 |
| HTR2A | SLC6A4 | 9606.ENSP00000437737 | 9606.ENSP00000261707 | 0 | 0 | 0 | 0 | 0 | 0 | 0 | 0.961 | 0.961 |
| ICAM1 | IFNG | 9606.ENSP00000264832 | 9606.ENSP00000229135 | 0 | 0 | 0 | 0 | 0.061 | 0.164 | 0 | 0.885 | 0.902 |
| ICAM1 | IL1B | 9606.ENSP00000264832 | 9606.ENSP00000263341 | 0 | 0 | 0 | 0 | 0.271 | 0 | 0 | 0.885 | 0.912 |
| ICAM1 | TNF | 9606.ENSP00000264832 | 9606.ENSP00000398698 | 0 | 0 | 0 | 0 | 0.255 | 0 | 0 | 0.929 | 0.944 |
| ICAM1 | IL6 | 9606.ENSP00000264832 | 9606.ENSP00000385675 | 0 | 0 | 0 | 0 | 0.27 | 0 | 0 | 0.931 | 0.947 |
| ICAM1 | SELE | 9606.ENSP00000264832 | 9606.ENSP00000331736 | 0 | 0 | 0 | 0 | 0.17 | 0 | 0 | 0.941 | 0.949 |
| ICAM1 | VCAM1 | 9606.ENSP00000264832 | 9606.ENSP00000294728 | 0 | 0 | 0 | 0 | 0.146 | 0 | 0 | 0.999 | 0.999 |
| IFNG | IL2 | 9606.ENSP00000229135 | 9606.ENSP00000226730 | 0 | 0 | 0 | 0 | 0.295 | 0 | 0 | 0.987 | 0.99 |
| IFNG | IRF1 | 9606.ENSP00000229135 | 9606.ENSP00000245414 | 0 | 0 | 0 | 0 | 0.142 | 0 | 0 | 0.908 | 0.917 |
| IFNG | IL1A | 9606.ENSP00000229135 | 9606.ENSP00000263339 | 0 | 0 | 0 | 0 | 0.166 | 0 | 0 | 0.966 | 0.971 |
| IFNG | IL6 | 9606.ENSP00000229135 | 9606.ENSP00000385675 | 0 | 0 | 0 | 0 | 0.128 | 0 | 0 | 0.981 | 0.983 |
| IFNG | STAT1 | 9606.ENSP00000229135 | 9606.ENSP00000354394 | 0 | 0 | 0 | 0 | 0.131 | 0 | 0.5 | 0.971 | 0.986 |
| IFNG | IL4 | 9606.ENSP00000229135 | 9606.ENSP00000231449 | 0 | 0 | 0 | 0 | 0.11 | 0 | 0 | 0.99 | 0.99 |
| IFNG | TNF | 9606.ENSP00000229135 | 9606.ENSP00000398698 | 0 | 0 | 0 | 0 | 0.202 | 0 | 0 | 0.99 | 0.991 |
| IFNG | IL10 | 9606.ENSP00000229135 | 9606.ENSP00000412237 | 0 | 0 | 0 | 0 | 0.566 | 0 | 0 | 0.989 | 0.995 |
| IFNG | IL1B | 9606.ENSP00000229135 | 9606.ENSP00000263341 | 0 | 0 | 0 | 0 | 0.273 | 0 | 0 | 0.997 | 0.998 |
| IGF2 | INSR | 9606.ENSP00000391826 | 9606.ENSP00000303830 | 0 | 0 | 0 | 0 | 0 | 0.164 | 0.65 | 0.971 | 0.991 |
| IGF2 | IGFBP3 | 9606.ENSP00000391826 | 9606.ENSP00000370473 | 0 | 0 | 0 | 0 | 0.19 | 0.789 | 0.5 | 0.999 | 0.999 |
| IGFBP3 | TP53 | 9606.ENSP00000370473 | 9606.ENSP00000269305 | 0 | 0 | 0 | 0 | 0 | 0 | 0.75 | 0.622 | 0.901 |
| IGFBP3 | RXRA | 9606.ENSP00000370473 | 9606.ENSP00000419692 | 0 | 0 | 0 | 0 | 0 | 0.292 | 0 | 0.907 | 0.931 |
| IKBKB | NFKBIA | 9606.ENSP00000430684 | 9606.ENSP00000216797 | 0 | 0 | 0 | 0 | 0.042 | 0.994 | 0.9 | 0.995 | 0.999 |
| IKBKB | TP53 | 9606.ENSP00000430684 | 9606.ENSP00000269305 | 0 | 0 | 0 | 0 | 0.076 | 0.51 | 0.5 | 0.739 | 0.933 |
| IKBKB | PTEN | 9606.ENSP00000430684 | 9606.ENSP00000361021 | 0 | 0 | 0 | 0 | 0.057 | 0.092 | 0.9 | 0.514 | 0.952 |
| IKBKB | RELA | 9606.ENSP00000430684 | 9606.ENSP00000384273 | 0 | 0 | 0 | 0 | 0.067 | 0.877 | 0.9 | 0.957 | 0.999 |
| IKBKB | TNF | 9606.ENSP00000430684 | 9606.ENSP00000398698 | 0 | 0 | 0 | 0 | 0.089 | 0.994 | 0.5 | 0.934 | 0.999 |
| IL10 | TGFB1 | 9606.ENSP00000412237 | 9606.ENSP00000221930 | 0 | 0 | 0 | 0 | 0 | 0 | 0.4 | 0.891 | 0.932 |
| IL10 | IL2 | 9606.ENSP00000412237 | 9606.ENSP00000226730 | 0 | 0 | 0 | 0 | 0.056 | 0 | 0 | 0.963 | 0.964 |
| IL10 | IL4 | 9606.ENSP00000412237 | 9606.ENSP00000231449 | 0 | 0 | 0 | 0 | 0.043 | 0 | 0 | 0.986 | 0.986 |
| IL10 | IL1A | 9606.ENSP00000412237 | 9606.ENSP00000263339 | 0 | 0 | 0 | 0 | 0.209 | 0 | 0.4 | 0.944 | 0.971 |
| IL10 | IL1B | 9606.ENSP00000412237 | 9606.ENSP00000263341 | 0 | 0 | 0 | 0 | 0.409 | 0 | 0.4 | 0.978 | 0.991 |
| IL10 | IL6 | 9606.ENSP00000412237 | 9606.ENSP00000385675 | 0 | 0 | 0 | 0 | 0.13 | 0 | 0.8 | 0.988 | 0.997 |
| IL10 | TNF | 9606.ENSP00000412237 | 9606.ENSP00000398698 | 0 | 0 | 0 | 0 | 0.234 | 0 | 0.4 | 0.984 | 0.992 |
| IL1A | IL2 | 9606.ENSP00000263339 | 9606.ENSP00000226730 | 0 | 0 | 0 | 0 | 0.111 | 0 | 0 | 0.997 | 0.997 |
| IL1A | IL4 | 9606.ENSP00000263339 | 9606.ENSP00000231449 | 0 | 0 | 0 | 0 | 0.111 | 0 | 0.4 | 0.992 | 0.995 |
| IL1A | JUN | 9606.ENSP00000263339 | 9606.ENSP00000360266 | 0 | 0 | 0 | 0 | 0.087 | 0 | 0.75 | 0.775 | 0.944 |
| IL1A | IL6 | 9606.ENSP00000263339 | 9606.ENSP00000385675 | 0 | 0 | 0 | 0 | 0.376 | 0 | 0.4 | 0.974 | 0.989 |
| IL1A | TNF | 9606.ENSP00000263339 | 9606.ENSP00000398698 | 0 | 0 | 0 | 0 | 0.619 | 0 | 0.4 | 0.976 | 0.994 |
| IL1A | IL1B | 9606.ENSP00000263339 | 9606.ENSP00000263341 | 0 | 0 | 0 | 0 | 0.961 | 0 | 0.9 | 0.983 | 0.999 |
| IL1B | NFKBIA | 9606.ENSP00000263341 | 9606.ENSP00000216797 | 0 | 0 | 0 | 0 | 0.283 | 0 | 0 | 0.927 | 0.945 |
| IL1B | TGFB1 | 9606.ENSP00000263341 | 9606.ENSP00000221930 | 0 | 0 | 0 | 0 | 0.14 | 0 | 0 | 0.924 | 0.932 |
| IL1B | IL2 | 9606.ENSP00000263341 | 9606.ENSP00000226730 | 0 | 0 | 0 | 0 | 0.073 | 0 | 0 | 0.999 | 0.999 |
| IL1B | IL4 | 9606.ENSP00000263341 | 9606.ENSP00000231449 | 0 | 0 | 0 | 0 | 0.072 | 0 | 0.4 | 0.998 | 0.998 |
| IL1B | MMP9 | 9606.ENSP00000263341 | 9606.ENSP00000361405 | 0 | 0 | 0 | 0 | 0.275 | 0.051 | 0 | 0.932 | 0.949 |
| IL1B | PTGS2 | 9606.ENSP00000263341 | 9606.ENSP00000356438 | 0 | 0 | 0 | 0 | 0.656 | 0 | 0 | 0.928 | 0.974 |
| IL1B | JUN | 9606.ENSP00000263341 | 9606.ENSP00000360266 | 0 | 0 | 0 | 0 | 0.166 | 0 | 0.9 | 0.857 | 0.987 |
| IL1B | RELA | 9606.ENSP00000263341 | 9606.ENSP00000384273 | 0 | 0 | 0 | 0 | 0.118 | 0 | 0.9 | 0.903 | 0.99 |
| IL1B | IL6 | 9606.ENSP00000263341 | 9606.ENSP00000385675 | 0 | 0 | 0 | 0 | 0.515 | 0 | 0.4 | 0.989 | 0.996 |
| IL1B | TNF | 9606.ENSP00000263341 | 9606.ENSP00000398698 | 0 | 0 | 0 | 0 | 0.616 | 0 | 0.4 | 0.993 | 0.998 |
| IL2 | TNF | 9606.ENSP00000226730 | 9606.ENSP00000398698 | 0 | 0 | 0 | 0 | 0.072 | 0 | 0 | 0.955 | 0.957 |
| IL2 | IL4 | 9606.ENSP00000226730 | 9606.ENSP00000231449 | 0 | 0 | 0 | 0 | 0.432 | 0 | 0 | 0.999 | 0.999 |
| IL2 | IL6 | 9606.ENSP00000226730 | 9606.ENSP00000385675 | 0 | 0 | 0 | 0 | 0.055 | 0 | 0 | 0.999 | 0.999 |
| IL4 | TNF | 9606.ENSP00000231449 | 9606.ENSP00000398698 | 0 | 0 | 0 | 0 | 0.055 | 0 | 0.4 | 0.963 | 0.977 |
| IL4 | IL6 | 9606.ENSP00000231449 | 9606.ENSP00000385675 | 0 | 0 | 0 | 0 | 0.044 | 0 | 0.4 | 0.978 | 0.986 |
| IL6 | MAPK1 | 9606.ENSP00000385675 | 9606.ENSP00000215832 | 0 | 0 | 0 | 0 | 0.055 | 0 | 0.4 | 0.897 | 0.936 |
| IL6 | NFKBIA | 9606.ENSP00000385675 | 9606.ENSP00000216797 | 0 | 0 | 0 | 0 | 0.175 | 0 | 0 | 0.915 | 0.927 |
| IL6 | TGFB1 | 9606.ENSP00000385675 | 9606.ENSP00000221930 | 0 | 0 | 0 | 0 | 0.106 | 0 | 0.4 | 0.932 | 0.96 |
| IL6 | VCAM1 | 9606.ENSP00000385675 | 9606.ENSP00000294728 | 0 | 0 | 0 | 0 | 0.171 | 0 | 0 | 0.909 | 0.921 |
| IL6 | STAT1 | 9606.ENSP00000385675 | 9606.ENSP00000354394 | 0 | 0 | 0 | 0 | 0.11 | 0.087 | 0.5 | 0.941 | 0.972 |
| IL6 | PTGS2 | 9606.ENSP00000385675 | 9606.ENSP00000356438 | 0 | 0 | 0 | 0 | 0.484 | 0 | 0 | 0.926 | 0.96 |
| IL6 | JUN | 9606.ENSP00000385675 | 9606.ENSP00000360266 | 0 | 0 | 0 | 0 | 0.14 | 0 | 0.9 | 0.882 | 0.989 |
| IL6 | MMP9 | 9606.ENSP00000385675 | 9606.ENSP00000361405 | 0 | 0 | 0 | 0 | 0.106 | 0 | 0.4 | 0.93 | 0.959 |
| IL6 | MAPK8 | 9606.ENSP00000385675 | 9606.ENSP00000378974 | 0 | 0 | 0 | 0 | 0 | 0 | 0 | 0.938 | 0.938 |
| IL6 | RELA | 9606.ENSP00000385675 | 9606.ENSP00000384273 | 0 | 0 | 0 | 0 | 0.108 | 0.292 | 0.9 | 0.816 | 0.986 |
| IL6 | TNF | 9606.ENSP00000385675 | 9606.ENSP00000398698 | 0 | 0 | 0 | 0 | 0.261 | 0 | 0.4 | 0.989 | 0.994 |
| INSR | PRKCB | 9606.ENSP00000303830 | 9606.ENSP00000496129 | 0 | 0 | 0 | 0.556 | 0 | 0.05 | 0.9 | 0.143 | 0.911 |
| INSR | PTPN1 | 9606.ENSP00000303830 | 9606.ENSP00000360683 | 0 | 0 | 0 | 0 | 0.055 | 0.991 | 0.9 | 0.76 | 0.999 |
| IRF1 | JUN | 9606.ENSP00000245414 | 9606.ENSP00000360266 | 0 | 0 | 0 | 0 | 0.164 | 0.068 | 0 | 0.905 | 0.919 |
| IRF1 | MDM2 | 9606.ENSP00000245414 | 9606.ENSP00000258149 | 0 | 0 | 0 | 0 | 0.049 | 0.626 | 0.9 | 0.272 | 0.97 |
| IRF1 | STAT1 | 9606.ENSP00000245414 | 9606.ENSP00000354394 | 0 | 0 | 0 | 0 | 0.44 | 0.805 | 0.5 | 0.997 | 0.999 |
| JUN | SIRT1 | 9606.ENSP00000360266 | 9606.ENSP00000212015 | 0 | 0 | 0 | 0 | 0.044 | 0.71 | 0 | 0.758 | 0.927 |
| JUN | MAPK1 | 9606.ENSP00000360266 | 9606.ENSP00000215832 | 0 | 0 | 0 | 0 | 0 | 0.771 | 0.9 | 0.958 | 0.998 |
| JUN | NFKBIA | 9606.ENSP00000360266 | 9606.ENSP00000216797 | 0 | 0 | 0 | 0 | 0.235 | 0.292 | 0 | 0.844 | 0.908 |
| JUN | MAPK14 | 9606.ENSP00000360266 | 9606.ENSP00000229795 | 0 | 0 | 0 | 0 | 0 | 0.328 | 0.9 | 0.891 | 0.992 |
| JUN | NR3C1 | 9606.ENSP00000360266 | 9606.ENSP00000231509 | 0 | 0 | 0 | 0 | 0 | 0.522 | 0 | 0.922 | 0.961 |
| JUN | TP53 | 9606.ENSP00000360266 | 9606.ENSP00000269305 | 0 | 0 | 0 | 0 | 0 | 0.324 | 0.5 | 0.992 | 0.997 |
| JUN | STAT1 | 9606.ENSP00000360266 | 9606.ENSP00000354394 | 0 | 0 | 0 | 0 | 0.055 | 0.328 | 0 | 0.869 | 0.91 |
| JUN | NFE2L2 | 9606.ENSP00000360266 | 9606.ENSP00000380252 | 0 | 0 | 0 | 0 | 0.049 | 0.629 | 0 | 0.791 | 0.92 |
| JUN | NFATC1 | 9606.ENSP00000360266 | 9606.ENSP00000389377 | 0 | 0 | 0 | 0 | 0.042 | 0.33 | 0 | 0.948 | 0.963 |
| JUN | NCOA1 | 9606.ENSP00000360266 | 9606.ENSP00000385216 | 0 | 0 | 0 | 0 | 0 | 0.625 | 0.8 | 0.574 | 0.965 |
| JUN | RUNX2 | 9606.ENSP00000360266 | 9606.ENSP00000360493 | 0 | 0 | 0 | 0 | 0.055 | 0.51 | 0 | 0.937 | 0.968 |
| JUN | MYC | 9606.ENSP00000360266 | 9606.ENSP00000478887 | 0 | 0 | 0 | 0 | 0.079 | 0.626 | 0 | 0.922 | 0.97 |
| JUN | PPARA | 9606.ENSP00000360266 | 9606.ENSP00000385523 | 0 | 0 | 0 | 0 | 0 | 0.057 | 0 | 0.979 | 0.979 |
| JUN | RELA | 9606.ENSP00000360266 | 9606.ENSP00000384273 | 0 | 0 | 0 | 0 | 0.087 | 0.271 | 0 | 0.98 | 0.986 |
| JUN | TNF | 9606.ENSP00000360266 | 9606.ENSP00000398698 | 0 | 0 | 0 | 0 | 0.151 | 0 | 0.9 | 0.881 | 0.989 |
| JUN | MAPK8 | 9606.ENSP00000360266 | 9606.ENSP00000378974 | 0 | 0 | 0 | 0 | 0.049 | 0.997 | 0.9 | 0.978 | 0.999 |
| KCNH2 | SCN5A | 9606.ENSP00000262186 | 9606.ENSP00000328968 | 0 | 0 | 0 | 0 | 0.048 | 0.059 | 0 | 0.978 | 0.978 |
| KDR | PTPN1 | 9606.ENSP00000263923 | 9606.ENSP00000360683 | 0 | 0 | 0 | 0 | 0 | 0.051 | 0 | 0.938 | 0.938 |
| LYZ | MPO | 9606.ENSP00000261267 | 9606.ENSP00000225275 | 0 | 0 | 0 | 0 | 0.114 | 0 | 0 | 0.912 | 0.918 |
| MAOA | SLC6A4 | 9606.ENSP00000340684 | 9606.ENSP00000261707 | 0 | 0 | 0 | 0 | 0.044 | 0 | 0 | 0.942 | 0.942 |
| MAOA | MAOB | 9606.ENSP00000340684 | 9606.ENSP00000367309 | 0 | 0 | 0.052 | 0.976 | 0.163 | 0.609 | 0.9 | 0.063 | 0.965 |
| MAPK1 | NCF1 | 9606.ENSP00000215832 | 9606.ENSP00000289473 | 0 | 0 | 0 | 0 | 0.058 | 0.113 | 0.9 | 0.193 | 0.923 |
| MAPK1 | NFATC1 | 9606.ENSP00000215832 | 9606.ENSP00000389377 | 0 | 0 | 0 | 0 | 0.102 | 0 | 0.9 | 0.26 | 0.927 |
| MAPK1 | PRKCB | 9606.ENSP00000215832 | 9606.ENSP00000496129 | 0 | 0 | 0 | 0.591 | 0.126 | 0.102 | 0.9 | 0.196 | 0.928 |
| MAPK1 | PRKCA | 9606.ENSP00000215832 | 9606.ENSP00000408695 | 0 | 0 | 0 | 0.594 | 0.127 | 0.102 | 0.9 | 0.233 | 0.931 |
| MAPK1 | STAT1 | 9606.ENSP00000215832 | 9606.ENSP00000354394 | 0 | 0 | 0 | 0 | 0.09 | 0.091 | 0.9 | 0.471 | 0.95 |
| MAPK1 | RAF1 | 9606.ENSP00000215832 | 9606.ENSP00000401888 | 0 | 0 | 0 | 0.588 | 0.115 | 0.8 | 0.7 | 0.593 | 0.975 |
| MAPK1 | MYC | 9606.ENSP00000215832 | 9606.ENSP00000478887 | 0 | 0 | 0 | 0 | 0 | 0.643 | 0.9 | 0.582 | 0.983 |
| MAPK1 | MAPK14 | 9606.ENSP00000215832 | 9606.ENSP00000229795 | 0 | 0 | 0.067 | 0.924 | 0.342 | 0.816 | 0.9 | 0.677 | 0.995 |
| MAPK1 | TP53 | 9606.ENSP00000215832 | 9606.ENSP00000269305 | 0 | 0 | 0 | 0 | 0.045 | 0.806 | 0.9 | 0.912 | 0.998 |
| MAPK14 | PGR | 9606.ENSP00000229795 | 9606.ENSP00000325120 | 0 | 0 | 0 | 0 | 0.055 | 0 | 0.9 | 0.221 | 0.92 |
| MAPK14 | MAPK8 | 9606.ENSP00000229795 | 9606.ENSP00000378974 | 0 | 0 | 0.061 | 0.922 | 0.11 | 0.292 | 0.9 | 0.366 | 0.955 |
| MAPK14 | TP53 | 9606.ENSP00000229795 | 9606.ENSP00000269305 | 0 | 0 | 0 | 0 | 0.046 | 0.818 | 0.9 | 0.744 | 0.994 |
| MAPK8 | TP53 | 9606.ENSP00000378974 | 9606.ENSP00000269305 | 0 | 0 | 0 | 0 | 0.086 | 0.845 | 0.9 | 0.861 | 0.997 |
| MAPK8 | PGR | 9606.ENSP00000378974 | 9606.ENSP00000325120 | 0 | 0 | 0 | 0 | 0 | 0 | 0.9 | 0.248 | 0.921 |
| MAPK8 | NFATC1 | 9606.ENSP00000378974 | 9606.ENSP00000389377 | 0 | 0 | 0 | 0 | 0 | 0.234 | 0.9 | 0.419 | 0.951 |
| MAPK8 | RELA | 9606.ENSP00000378974 | 9606.ENSP00000384273 | 0 | 0 | 0 | 0 | 0.054 | 0.045 | 0.9 | 0.758 | 0.975 |
| MDM2 | PTEN | 9606.ENSP00000258149 | 9606.ENSP00000361021 | 0 | 0 | 0 | 0 | 0.069 | 0.045 | 0.4 | 0.838 | 0.902 |
| MDM2 | XIAP | 9606.ENSP00000258149 | 9606.ENSP00000360242 | 0 | 0 | 0 | 0 | 0.264 | 0.596 | 0 | 0.782 | 0.929 |
| MDM2 | RB1 | 9606.ENSP00000258149 | 9606.ENSP00000267163 | 0 | 0 | 0 | 0 | 0.042 | 0.889 | 0.9 | 0.925 | 0.999 |
| MDM2 | TP53 | 9606.ENSP00000258149 | 9606.ENSP00000269305 | 0 | 0 | 0 | 0 | 0.116 | 0.999 | 0.9 | 0.999 | 0.999 |
| MET | PTPN1 | 9606.ENSP00000317272 | 9606.ENSP00000360683 | 0 | 0 | 0 | 0 | 0 | 0.692 | 0.4 | 0.771 | 0.954 |
| MGAM | PYGM | 9606.ENSP00000447378 | 9606.ENSP00000164139 | 0.057 | 0 | 0 | 0 | 0 | 0 | 0.93 | 0.604 | 0.969 |
| MMP1 | MMP2 | 9606.ENSP00000322788 | 9606.ENSP00000219070 | 0 | 0 | 0 | 0.864 | 0.181 | 0 | 0.9 | 0.215 | 0.93 |
| MMP1 | MMP3 | 9606.ENSP00000322788 | 9606.ENSP00000299855 | 0 | 0 | 0.064 | 0.951 | 0.657 | 0 | 0.9 | 0.597 | 0.985 |
| MMP1 | MMP9 | 9606.ENSP00000322788 | 9606.ENSP00000361405 | 0 | 0 | 0 | 0.743 | 0.154 | 0 | 0.9 | 0.725 | 0.974 |
| MMP2 | MMP9 | 9606.ENSP00000219070 | 9606.ENSP00000361405 | 0 | 0 | 0.061 | 0.927 | 0.128 | 0 | 0.9 | 0.116 | 0.918 |
| MMP2 | NCF1 | 9606.ENSP00000219070 | 9606.ENSP00000289473 | 0 | 0 | 0 | 0 | 0.056 | 0 | 0.9 | 0.3 | 0.928 |
| MMP2 | TGFB1 | 9606.ENSP00000219070 | 9606.ENSP00000221930 | 0 | 0 | 0 | 0 | 0.07 | 0.6 | 0.4 | 0.85 | 0.962 |
| MMP3 | MMP9 | 9606.ENSP00000299855 | 9606.ENSP00000361405 | 0 | 0 | 0.075 | 0.784 | 0.145 | 0 | 0.9 | 0.514 | 0.956 |
| MMP3 | SPP1 | 9606.ENSP00000299855 | 9606.ENSP00000378517 | 0 | 0 | 0 | 0 | 0.07 | 0 | 0.75 | 0.992 | 0.997 |
| MMP9 | TGFB1 | 9606.ENSP00000361405 | 9606.ENSP00000221930 | 0 | 0 | 0 | 0 | 0.082 | 0.599 | 0.4 | 0.872 | 0.968 |
| MMP9 | MPO | 9606.ENSP00000361405 | 9606.ENSP00000225275 | 0 | 0 | 0 | 0 | 0.085 | 0 | 0 | 0.967 | 0.969 |
| MMP9 | NCF1 | 9606.ENSP00000361405 | 9606.ENSP00000289473 | 0 | 0 | 0 | 0 | 0.189 | 0 | 0.9 | 0.432 | 0.95 |
| MMP9 | TNF | 9606.ENSP00000361405 | 9606.ENSP00000398698 | 0 | 0 | 0 | 0 | 0.207 | 0 | 0 | 0.915 | 0.93 |
| MMP9 | SPP1 | 9606.ENSP00000361405 | 9606.ENSP00000378517 | 0 | 0 | 0 | 0 | 0.138 | 0 | 0 | 0.941 | 0.947 |
| MYC | TP53 | 9606.ENSP00000478887 | 9606.ENSP00000269305 | 0 | 0 | 0 | 0 | 0.067 | 0.3 | 0 | 0.997 | 0.997 |
| MYC | PTEN | 9606.ENSP00000478887 | 9606.ENSP00000361021 | 0 | 0 | 0 | 0 | 0.044 | 0.317 | 0 | 0.87 | 0.908 |
| MYC | NFE2L2 | 9606.ENSP00000478887 | 9606.ENSP00000380252 | 0 | 0 | 0 | 0 | 0.066 | 0.531 | 0.75 | 0.693 | 0.961 |
| NCF1 | VCAM1 | 9606.ENSP00000289473 | 9606.ENSP00000294728 | 0 | 0 | 0 | 0 | 0.043 | 0 | 0.9 | 0.445 | 0.942 |
| NCF1 | NOX5 | 9606.ENSP00000289473 | 9606.ENSP00000373518 | 0 | 0 | 0 | 0 | 0.073 | 0 | 0 | 0.945 | 0.947 |
| NCF1 | PRKCA | 9606.ENSP00000289473 | 9606.ENSP00000408695 | 0 | 0 | 0 | 0 | 0.06 | 0.51 | 0.9 | 0.417 | 0.969 |
| NCF1 | PRKCB | 9606.ENSP00000289473 | 9606.ENSP00000496129 | 0 | 0 | 0 | 0 | 0.178 | 0.625 | 0.9 | 0.305 | 0.975 |
| NCOA1 | NR3C1 | 9606.ENSP00000385216 | 9606.ENSP00000231509 | 0 | 0 | 0 | 0 | 0.067 | 0.989 | 0 | 0.8 | 0.997 |
| NCOA1 | PPARG | 9606.ENSP00000385216 | 9606.ENSP00000287820 | 0 | 0 | 0 | 0 | 0 | 0.992 | 0.5 | 0.907 | 0.999 |
| NCOA1 | PGR | 9606.ENSP00000385216 | 9606.ENSP00000325120 | 0 | 0 | 0 | 0 | 0 | 0.855 | 0.8 | 0.923 | 0.997 |
| NCOA1 | NR1I2 | 9606.ENSP00000385216 | 9606.ENSP00000336528 | 0 | 0 | 0 | 0 | 0 | 0.989 | 0 | 0.883 | 0.998 |
| NCOA1 | NR3C2 | 9606.ENSP00000385216 | 9606.ENSP00000350815 | 0 | 0 | 0 | 0 | 0.068 | 0.942 | 0 | 0.541 | 0.973 |
| NCOA1 | PPARA | 9606.ENSP00000385216 | 9606.ENSP00000385523 | 0 | 0 | 0 | 0 | 0.06 | 0.985 | 0.5 | 0.784 | 0.998 |
| NCOA1 | RXRA | 9606.ENSP00000385216 | 9606.ENSP00000419692 | 0 | 0 | 0 | 0 | 0.083 | 0.995 | 0.8 | 0.801 | 0.999 |
| NFATC1 | PPP3CA | 9606.ENSP00000389377 | 9606.ENSP00000378323 | 0 | 0 | 0 | 0 | 0 | 0.976 | 0.9 | 0.85 | 0.999 |
| NFE2L2 | NQO1 | 9606.ENSP00000380252 | 9606.ENSP00000319788 | 0 | 0 | 0 | 0 | 0.082 | 0.292 | 0.5 | 0.94 | 0.978 |
| NFKBIA | TP53 | 9606.ENSP00000216797 | 9606.ENSP00000269305 | 0 | 0 | 0 | 0 | 0.051 | 0.623 | 0 | 0.781 | 0.915 |
| NFKBIA | STAT1 | 9606.ENSP00000216797 | 9606.ENSP00000354394 | 0 | 0 | 0 | 0 | 0.115 | 0.294 | 0 | 0.877 | 0.917 |
| NFKBIA | TNF | 9606.ENSP00000216797 | 9606.ENSP00000398698 | 0 | 0 | 0 | 0 | 0.338 | 0.292 | 0 | 0.937 | 0.967 |
| NFKBIA | RELA | 9606.ENSP00000216797 | 9606.ENSP00000384273 | 0 | 0.003 | 0 | 0 | 0.147 | 0.999 | 0.9 | 0.999 | 0.999 |
| NOS2 | NOS3 | 9606.ENSP00000327251 | 9606.ENSP00000297494 | 0 | 0 | 0.069 | 0.942 | 0 | 0.087 | 0.9 | 0.09 | 0.912 |
| NOS2 | PTGS2 | 9606.ENSP00000327251 | 9606.ENSP00000356438 | 0 | 0 | 0 | 0 | 0 | 0.095 | 0 | 0.902 | 0.908 |
| NQO1 | ODC1 | 9606.ENSP00000319788 | 9606.ENSP00000234111 | 0.061 | 0 | 0 | 0 | 0.049 | 0 | 0.9 | 0.53 | 0.952 |
| NQO1 | TP53 | 9606.ENSP00000319788 | 9606.ENSP00000269305 | 0 | 0 | 0 | 0 | 0 | 0.735 | 0 | 0.806 | 0.946 |
| NR1I2 | RXRA | 9606.ENSP00000336528 | 9606.ENSP00000419692 | 0 | 0 | 0 | 0.628 | 0 | 0.97 | 0 | 0.982 | 0.999 |
| NR3C1 | NR3C2 | 9606.ENSP00000231509 | 9606.ENSP00000350815 | 0 | 0 | 0.075 | 0.856 | 0.044 | 0.51 | 0.5 | 0.725 | 0.929 |
| NR3C1 | TP53 | 9606.ENSP00000231509 | 9606.ENSP00000269305 | 0 | 0 | 0 | 0 | 0 | 0.51 | 0 | 0.875 | 0.936 |
| OPRD1 | OPRM1 | 9606.ENSP00000234961 | 9606.ENSP00000394624 | 0 | 0 | 0.052 | 0.961 | 0.121 | 0.516 | 0.9 | 0.319 | 0.967 |
| PARP1 | TP53 | 9606.ENSP00000355759 | 9606.ENSP00000269305 | 0 | 0 | 0 | 0 | 0.067 | 0.794 | 0 | 0.87 | 0.972 |
| PARP1 | TOP1 | 9606.ENSP00000355759 | 9606.ENSP00000354522 | 0 | 0 | 0 | 0 | 0.06 | 0.628 | 0 | 0.831 | 0.936 |
| PCNA | TP53 | 9606.ENSP00000368458 | 9606.ENSP00000269305 | 0 | 0 | 0 | 0 | 0.067 | 0.777 | 0.5 | 0.265 | 0.913 |
| PLAT | THBD | 9606.ENSP00000220809 | 9606.ENSP00000366307 | 0 | 0 | 0 | 0 | 0.092 | 0.094 | 0 | 0.903 | 0.913 |
| PLAT | SERPINE1 | 9606.ENSP00000220809 | 9606.ENSP00000223095 | 0 | 0 | 0 | 0 | 0.202 | 0.959 | 0.9 | 0.999 | 0.999 |
| PLAU | SERPINE1 | 9606.ENSP00000361850 | 9606.ENSP00000223095 | 0 | 0 | 0 | 0 | 0.259 | 0.977 | 0.54 | 0.999 | 0.999 |
| PPARA | SIRT1 | 9606.ENSP00000385523 | 9606.ENSP00000212015 | 0 | 0 | 0 | 0 | 0 | 0.345 | 0 | 0.862 | 0.906 |
| PPARA | RXRA | 9606.ENSP00000385523 | 9606.ENSP00000419692 | 0 | 0 | 0 | 0.689 | 0.084 | 0.84 | 0.9 | 0.986 | 0.999 |
| PPARD | RXRA | 9606.ENSP00000310928 | 9606.ENSP00000419692 | 0 | 0 | 0 | 0.699 | 0.049 | 0.761 | 0.9 | 0.838 | 0.995 |
| PPARG | SIRT1 | 9606.ENSP00000287820 | 9606.ENSP00000212015 | 0 | 0 | 0 | 0 | 0 | 0.345 | 0.9 | 0.981 | 0.998 |
| PPARG | TGFB1 | 9606.ENSP00000287820 | 9606.ENSP00000221930 | 0 | 0 | 0 | 0 | 0.105 | 0 | 0.75 | 0.68 | 0.922 |
| PPARG | TP53 | 9606.ENSP00000287820 | 9606.ENSP00000269305 | 0 | 0 | 0 | 0 | 0 | 0.305 | 0.5 | 0.866 | 0.949 |
| PPARG | SLC2A4 | 9606.ENSP00000287820 | 9606.ENSP00000320935 | 0 | 0 | 0 | 0 | 0.059 | 0 | 0.5 | 0.872 | 0.934 |
| PPARG | RELA | 9606.ENSP00000287820 | 9606.ENSP00000384273 | 0 | 0 | 0 | 0 | 0.087 | 0.834 | 0.75 | 0.976 | 0.998 |
| PPARG | RXRA | 9606.ENSP00000287820 | 9606.ENSP00000419692 | 0 | 0 | 0 | 0.688 | 0.061 | 0.999 | 0.9 | 0.987 | 0.999 |
| PPP3CA | SOD1 | 9606.ENSP00000378323 | 9606.ENSP00000270142 | 0 | 0 | 0 | 0 | 0.055 | 0.106 | 0.9 | 0.486 | 0.95 |
| PRKCA | SLC6A3 | 9606.ENSP00000408695 | 9606.ENSP00000270349 | 0 | 0 | 0 | 0 | 0 | 0 | 0.9 | 0.174 | 0.913 |
| PRKCA | RAF1 | 9606.ENSP00000408695 | 9606.ENSP00000401888 | 0 | 0 | 0 | 0.58 | 0.042 | 0.05 | 0.9 | 0.199 | 0.917 |
| PRKCA | PRKCB | 9606.ENSP00000408695 | 9606.ENSP00000496129 | 0 | 0 | 0.05 | 0.981 | 0.084 | 0.623 | 0.9 | 0.056 | 0.963 |
| PRKCB | SLC6A3 | 9606.ENSP00000496129 | 9606.ENSP00000270349 | 0 | 0 | 0 | 0 | 0 | 0 | 0.9 | 0.205 | 0.917 |
| PRKCB | RAF1 | 9606.ENSP00000496129 | 9606.ENSP00000401888 | 0 | 0 | 0 | 0.572 | 0.047 | 0.05 | 0.9 | 0.111 | 0.908 |
| PTEN | TP53 | 9606.ENSP00000361021 | 9606.ENSP00000269305 | 0 | 0 | 0 | 0 | 0.067 | 0.748 | 0.9 | 0.968 | 0.999 |
| PTEN | XIAP | 9606.ENSP00000361021 | 9606.ENSP00000360242 | 0 | 0 | 0 | 0 | 0.103 | 0.534 | 0.4 | 0.723 | 0.921 |
| PTGES | PTGS1 | 9606.ENSP00000342385 | 9606.ENSP00000354612 | 0 | 0 | 0 | 0 | 0 | 0 | 0.9 | 0.741 | 0.973 |
| PTGES | PTGS2 | 9606.ENSP00000342385 | 9606.ENSP00000356438 | 0 | 0 | 0 | 0 | 0.074 | 0 | 0.9 | 0.828 | 0.982 |
| PTGS1 | PTGS2 | 9606.ENSP00000354612 | 9606.ENSP00000356438 | 0 | 0 | 0.057 | 0.966 | 0.157 | 0.292 | 0.9 | 0.192 | 0.946 |
| PTGS2 | TP53 | 9606.ENSP00000356438 | 9606.ENSP00000269305 | 0 | 0 | 0 | 0 | 0 | 0.82 | 0 | 0.866 | 0.974 |
| PTGS2 | TNF | 9606.ENSP00000356438 | 9606.ENSP00000398698 | 0 | 0 | 0 | 0 | 0.185 | 0 | 0 | 0.901 | 0.916 |
| RAF1 | RELA | 9606.ENSP00000401888 | 9606.ENSP00000384273 | 0 | 0 | 0 | 0 | 0.101 | 0.098 | 0.9 | 0.186 | 0.925 |
| RB1 | TP53 | 9606.ENSP00000267163 | 9606.ENSP00000269305 | 0 | 0 | 0 | 0 | 0.064 | 0.51 | 0 | 0.872 | 0.936 |
| RB1 | RUNX2 | 9606.ENSP00000267163 | 9606.ENSP00000360493 | 0 | 0 | 0 | 0 | 0 | 0.512 | 0.7 | 0.816 | 0.97 |
| RELA | SIRT1 | 9606.ENSP00000384273 | 9606.ENSP00000212015 | 0 | 0 | 0 | 0 | 0.049 | 0.646 | 0.9 | 0.89 | 0.995 |
| RELA | TP53 | 9606.ENSP00000384273 | 9606.ENSP00000269305 | 0 | 0 | 0 | 0 | 0.126 | 0.45 | 0 | 0.914 | 0.955 |
| RELA | TNF | 9606.ENSP00000384273 | 9606.ENSP00000398698 | 0 | 0 | 0 | 0 | 0.118 | 0.516 | 0.9 | 0.944 | 0.997 |
| RUNX2 | STAT1 | 9606.ENSP00000360493 | 9606.ENSP00000354394 | 0 | 0 | 0 | 0 | 0.059 | 0 | 0.7 | 0.812 | 0.942 |
| RUNX2 | SPP1 | 9606.ENSP00000360493 | 9606.ENSP00000378517 | 0 | 0 | 0 | 0 | 0 | 0 | 0 | 0.901 | 0.901 |
| SELE | VCAM1 | 9606.ENSP00000331736 | 9606.ENSP00000294728 | 0 | 0 | 0 | 0 | 0.179 | 0 | 0 | 0.976 | 0.979 |
| SIRT1 | TP53 | 9606.ENSP00000212015 | 9606.ENSP00000269305 | 0 | 0 | 0 | 0 | 0 | 0.886 | 0.9 | 0.99 | 0.999 |
| TGFB1 | TNF | 9606.ENSP00000221930 | 9606.ENSP00000398698 | 0 | 0 | 0 | 0 | 0.125 | 0 | 0 | 0.935 | 0.941 |
| TNF | VCAM1 | 9606.ENSP00000398698 | 9606.ENSP00000294728 | 0 | 0 | 0 | 0 | 0.1 | 0 | 0 | 0.918 | 0.923 |
| TNF | XIAP | 9606.ENSP00000398698 | 9606.ENSP00000360242 | 0 | 0 | 0 | 0 | 0.055 | 0 | 0.5 | 0.81 | 0.902 |
| TOP1 | TP53 | 9606.ENSP00000354522 | 9606.ENSP00000269305 | 0 | 0 | 0 | 0 | 0 | 0.643 | 0 | 0.86 | 0.947 |
