# Supplementary material for: Elucidating the mechanism of Buyang Huanwu Decoction in the treatment of ischemic stroke: A network pharmacology and molecular docking study
Source: Medicine (Baltimore). 2026 Jul 17;105(29):e49736. doi: 10.1097/MD.0000000000049736 (PMC13384647; doi:10.1097/MD.0000000000049736)
Supplement: Supplementary file 7 [file medi-105-e49736-s007.docx]

**S 7.** Initial CytoNCA analysis results following the import of Protein-protein interaction network data into Cytoscape.

| **Name** | **Betweenness** | **Closeness** | **Degree** | **Eigenvector** | **LAC** | **Network** |
| --- | --- | --- | --- | --- | --- | --- |
| ACHE | 269.6663431 | 0.063377609 | 2 | 6.99E-04 | 0 | 0 |
| APP | 537.0229048 | 0.066454437 | 3 | 0.010775027 | 0 | 0 |
| MAOB | 170.0682551 | 0.061600885 | 3 | 1.01E-04 | 0.666667 | 1 |
| ADRB1 | 0 | 0.005988024 | 1 | 6.41E-08 | 0 | 0 |
| ADRB2 | 0 | 0.005988024 | 1 | 3.76E-08 | 0 | 0 |
| AHR | 622.6434466 | 0.066880256 | 3 | 0.026047695 | 0.666667 | 1 |
| ESR1 | 1125.219034 | 0.069991618 | 25 | 0.216380849 | 7.84 | 15.12600727 |
| CYP1A1 | 531.5738307 | 0.064678544 | 6 | 0.002111051 | 2 | 3.666666667 |
| HSP90AA1 | 1929.928326 | 0.069641368 | 28 | 0.186830431 | 4.714286 | 12.362055 |
| AHSA1 | 0 | 0.065438871 | 1 | 0.012008185 | 0 | 0 |
| AKT1 | 2130.83147 | 0.070464135 | 33 | 0.253844559 | 6.909091 | 16.71016398 |
| MAPK1 | 1085.246841 | 0.070079731 | 21 | 0.179208681 | 6.095238 | 9.94745939 |
| CCND1 | 183.3435164 | 0.069008264 | 18 | 0.170524076 | 7.111111 | 10.77009061 |
| MAPK14 | 116.5507488 | 0.06869601 | 12 | 0.117871381 | 5 | 6.568831169 |
| HSPB1 | 19.60647447 | 0.068135455 | 5 | 0.061971683 | 2.4 | 3 |
| MDM2 | 257.0916234 | 0.068752573 | 17 | 0.15356575 | 5.764706 | 8.18488456 |
| TP53 | 5400.982362 | 0.071003401 | 48 | 0.329142243 | 8.083333 | 35.60081235 |
| RASA1 | 0.640873016 | 0.066693291 | 2 | 0.024745176 | 0 | 0 |
| NCF1 | 926.6408607 | 0.067803492 | 9 | 0.039095964 | 1.111111 | 1.458333333 |
| NOS3 | 15.28806478 | 0.067230274 | 5 | 0.037744053 | 2.8 | 3.666666667 |
| CASP3 | 1314.385814 | 0.069816054 | 20 | 0.150842264 | 5.7 | 10.09592739 |
| HSPA5 | 67.64050518 | 0.066987565 | 4 | 0.031653628 | 0.5 | 0.666666667 |
| GSK3B | 349.7319 | 0.068922823 | 13 | 0.119467244 | 4.461538 | 6.78030303 |
| NOS2 | 122.249263 | 0.067230274 | 4 | 0.024726855 | 1 | 1.333333333 |
| CASP9 | 47.14101331 | 0.068274734 | 11 | 0.090097852 | 6.545455 | 8.383333333 |
| CAV1 | 642.3795255 | 0.068246833 | 13 | 0.085065819 | 2.923077 | 4.427489177 |
| STAT1 | 213.793087 | 0.068894389 | 11 | 0.094804652 | 3.454545 | 4.516666667 |
| CHUK | 79.35712446 | 0.06895128 | 9 | 0.094890885 | 4.444444 | 5.125 |
| XIAP | 141.8283722 | 0.068583162 | 12 | 0.092817657 | 5.5 | 6.812121212 |
| JUN | 2477.83342 | 0.070553443 | 31 | 0.243209034 | 7.483871 | 17.78709543 |
| PTEN | 63.23953748 | 0.068386568 | 12 | 0.116941251 | 4.833333 | 5.530844156 |
| BCL2L1 | 303.3868734 | 0.06872428 | 12 | 0.097112656 | 5.333333 | 7.236363636 |
| NFE2L2 | 254.4152714 | 0.067474747 | 6 | 0.053591982 | 2.333333 | 3.733333333 |
| BCL2 | 801.91342 | 0.069525396 | 20 | 0.175440788 | 6.7 | 10.90179236 |
| CDKN1A | 97.78702292 | 0.068358576 | 12 | 0.116210677 | 6.166667 | 7.951479076 |
| TNF | 2276.254144 | 0.069991618 | 27 | 0.155843392 | 7.407407 | 17.62634851 |
| RAF1 | 90.34179316 | 0.067858594 | 7 | 0.064197756 | 2.285714 | 2.666666667 |
| PIK3CG | 0 | 0.066164818 | 1 | 0.016313588 | 0 | 0 |
| IKBKB | 68.23845727 | 0.068894389 | 8 | 0.089418799 | 4.5 | 5.142857143 |
| HIF1A | 270.6665041 | 0.069065343 | 15 | 0.16718097 | 6.933333 | 7.74025974 |
| MYC | 172.4906282 | 0.069179785 | 18 | 0.187474549 | 8.111111 | 10.57370236 |
| CTNNB1 | 612.2800878 | 0.069380972 | 20 | 0.165920228 | 5.9 | 10.7415877 |
| ALB | 795.5658034 | 0.067311568 | 5 | 0.016116688 | 0.8 | 1 |
| CRP | 845.3896728 | 0.066719936 | 5 | 0.020314587 | 1.2 | 1.5 |
| IL6 | 2130.835332 | 0.069903725 | 25 | 0.141049668 | 7.44 | 15.7573296 |
| CYCS | 497.9023433 | 0.068246833 | 11 | 0.076544233 | 5.090909 | 6.316666667 |
| F2 | 945.3172787 | 0.063935681 | 6 | 0.002417315 | 2 | 3.766666667 |
| ALOX12 | 0 | 0.065515889 | 4 | 0.004518966 | 3 | 4 |
| CYP2B6 | 968.2486837 | 0.065955766 | 7 | 0.004724761 | 2.285714 | 3.9 |
| ALOX5 | 308 | 0.065541601 | 5 | 0.00453649 | 2.4 | 3.25 |
| PTGS2 | 2575.267358 | 0.069265865 | 11 | 0.056423236 | 2.363636 | 5.45 |
| PTGS1 | 14.9 | 0.065541601 | 5 | 0.004755524 | 2.8 | 4.5 |
| LTA4H | 0 | 0.06180607 | 1 | 2.91E-04 | 0 | 0 |
| AR | 44.08838589 | 0.067996743 | 7 | 0.082618743 | 3.714286 | 4.333333333 |
| NCOA1 | 916.6457973 | 0.067996743 | 12 | 0.063155308 | 2.5 | 5.577489177 |
| BAX | 918.6056277 | 0.067393059 | 6 | 0.049104594 | 2.333333 | 2.8 |
| SIRT1 | 178.8569838 | 0.068358576 | 9 | 0.082569629 | 3.333333 | 4.086904762 |
| HK2 | 612 | 0.063546423 | 2 | 0.003170175 | 0 | 0 |
| CTSD | 0 | 0.065490196 | 2 | 0.017519724 | 1 | 2 |
| SOD1 | 22.30197548 | 0.065593087 | 3 | 0.014236592 | 0 | 0 |
| CASP8 | 160.6041301 | 0.06869601 | 11 | 0.090629399 | 5.272727 | 6.116666667 |
| PARP1 | 27.24753584 | 0.067556634 | 6 | 0.051911179 | 3 | 4.4 |
| MAPK8 | 317.4669912 | 0.069323371 | 14 | 0.128059551 | 4.571429 | 5.992057942 |
| IL2 | 99.56438258 | 0.067122186 | 10 | 0.057060055 | 6.2 | 7.555555556 |
| BIRC5 | 13.02062859 | 0.067447496 | 5 | 0.045853704 | 2.4 | 3 |
| CCNA2 | 14.93780989 | 0.066960706 | 8 | 0.050459333 | 3.5 | 4.857142857 |
| CALM3 | 450.6287395 | 0.067068273 | 8 | 0.036833756 | 1.5 | 2.226190476 |
| NOX5 | 37.51454101 | 0.064804036 | 2 | 0.004878792 | 0 | 0 |
| PPP3CA | 24.42816082 | 0.065438871 | 4 | 0.014385656 | 0.5 | 0.666666667 |
| SCN5A | 3.166666667 | 0.06320969 | 2 | 0.003152122 | 0 | 0 |
| PRKCB | 565.1221758 | 0.067529317 | 10 | 0.044486646 | 2.6 | 4.083333333 |
| PRKCA | 460.1613031 | 0.067775974 | 10 | 0.052140135 | 2.8 | 4.305555556 |
| CASP7 | 26.0984349 | 0.066454437 | 7 | 0.037573583 | 3.428571 | 4.066666667 |
| IL1B | 500.5175579 | 0.069036792 | 19 | 0.110274047 | 8.315789 | 13.05137363 |
| KDR | 22.05144177 | 0.066428003 | 4 | 0.02959045 | 1.5 | 2 |
| ICAM1 | 337.0099845 | 0.067176187 | 9 | 0.050354261 | 4.222222 | 5.925 |
| EGFR | 1180.598794 | 0.069845253 | 18 | 0.131229922 | 3.666667 | 6.482536499 |
| GJA1 | 1.671572872 | 0.066217288 | 2 | 0.016981026 | 0 | 0 |
| ESR2 | 48.5744263 | 0.067969068 | 8 | 0.075719088 | 4.5 | 5.142857143 |
| DPP4 | 0 | 0.064206075 | 1 | 0.005466256 | 0 | 0 |
| CCL2 | 1203.72773 | 0.068358576 | 15 | 0.089927062 | 8.133333 | 10.71806527 |
| SERPINE1 | 852.1271657 | 0.064653504 | 4 | 0.005853191 | 0.5 | 0.833333333 |
| VCAM1 | 205.3073018 | 0.066826731 | 6 | 0.030877357 | 2.333333 | 3.6 |
| IL4 | 10.02680822 | 0.066666667 | 10 | 0.056611296 | 7.6 | 9.111111111 |
| CXCL2 | 69.31232567 | 0.067886179 | 10 | 0.074329406 | 7 | 8.222222222 |
| IL1A | 34.43252697 | 0.067831032 | 12 | 0.078276455 | 9 | 10.41717172 |
| IFNG | 86.05012713 | 0.067149176 | 14 | 0.071371071 | 7.571429 | 10.58848096 |
| IL10 | 13.79782237 | 0.066853483 | 11 | 0.061883733 | 8.181818 | 9.241666667 |
| CXCL10 | 33.32771427 | 0.066719936 | 11 | 0.063553602 | 6.909091 | 8.144444444 |
| RELA | 713.9174891 | 0.069670421 | 19 | 0.163136661 | 6 | 8.764774115 |
| CXCL8 | 304.8354992 | 0.068583162 | 16 | 0.09563075 | 8.75 | 12.35137363 |
| CDK2 | 5.967284956 | 0.066987565 | 9 | 0.074761331 | 5.777778 | 7.255952381 |
| RB1 | 57.54359592 | 0.067230274 | 7 | 0.058728065 | 3.428571 | 4 |
| E2F1 | 13.46866902 | 0.067122186 | 10 | 0.086144149 | 5.6 | 6.738095238 |
| PCNA | 0.333333333 | 0.066746603 | 5 | 0.047635976 | 3.6 | 4.5 |
| CHEK1 | 1 | 0.066693291 | 3 | 0.026502497 | 1.333333 | 2 |
| ERBB2 | 17.50831317 | 0.068107667 | 7 | 0.080394804 | 4 | 4.733333333 |
| FOS | 466.1175966 | 0.069294606 | 17 | 0.145646229 | 5.647059 | 8.21025086 |
| CDKN2A | 34.18309398 | 0.067913786 | 12 | 0.116779633 | 5.833333 | 6.65530303 |
| CD14 | 0 | 0.005988024 | 1 | 0 | 0 | 0 |
| LBP | 0 | 0.005988024 | 1 | 0 | 0 | 0 |
| CD40LG | 0 | 0.066034006 | 4 | 0.021851515 | 3 | 4 |
| CHEK2 | 1.887387387 | 0.066773291 | 3 | 0.032729574 | 1.333333 | 2 |
| CHRM1 | 0 | 0.005988024 | 1 | 0 | 0 | 0 |
| CHRM2 | 0 | 0.005988024 | 1 | 0 | 0 | 0 |
| CHRNA2 | 0 | 0.005988024 | 1 | 0 | 0 | 0 |
| CHRNA7 | 0 | 0.005988024 | 1 | 0 | 0 | 0 |
| NFKBIA | 59.28983235 | 0.068583162 | 9 | 0.091284238 | 4.666667 | 5.321428571 |
| COL3A1 | 0 | 0.005988024 | 1 | 0 | 0 | 0 |
| PCOLCE | 0 | 0.005988024 | 1 | 0 | 0 | 0 |
| OLR1 | 0 | 0.062852842 | 1 | 0.001302252 | 0 | 0 |
| PPARG | 733.7435733 | 0.068583162 | 8 | 0.055566002 | 1.75 | 2.057142857 |
| MET | 0 | 0.066217288 | 3 | 0.020547589 | 2 | 3 |
| PTPN1 | 96.53801883 | 0.066322478 | 5 | 0.022603685 | 1.6 | 2.583333333 |
| CXCL11 | 2.649350649 | 0.065464524 | 4 | 0.017903848 | 1.5 | 2 |
| TGFB1 | 436.7793558 | 0.068135455 | 9 | 0.045826685 | 2.444444 | 2.958333333 |
| GSTM1 | 192.0157204 | 0.063137996 | 4 | 5.70E-04 | 1 | 1.333333333 |
| CYP1B1 | 48.43945584 | 0.062900188 | 4 | 4.89E-04 | 1.5 | 2.166666667 |
| GSTA2 | 6.206722689 | 0.061464851 | 3 | 2.19E-04 | 1.333333 | 2 |
| CYP3A4 | 1161.312146 | 0.063960169 | 7 | 8.11E-04 | 1.142857 | 2.6 |
| NR1I2 | 452.7074948 | 0.064980545 | 3 | 0.004854016 | 0.666667 | 1 |
| MAOA | 612 | 0.060727273 | 3 | 5.88E-05 | 0.666667 | 1 |
| DRD1 | 0 | 0.060354174 | 1 | 4.01E-04 | 0 | 0 |
| SLC6A3 | 308 | 0.063911213 | 3 | 0.00623541 | 0.666667 | 1 |
| DUOX2 | 0 | 0.063813527 | 1 | 0.002511592 | 0 | 0 |
| IGFBP3 | 464.845182 | 0.067969068 | 4 | 0.030474909 | 0.5 | 0.666666667 |
| EGLN1 | 4.593338907 | 0.065696302 | 3 | 0.01695318 | 0.666667 | 1 |
| ELK1 | 10.48864086 | 0.066296149 | 5 | 0.033525635 | 2 | 2.5 |
| PGR | 30.20203919 | 0.067284448 | 6 | 0.050937522 | 2.333333 | 2.8 |
| RUNX2 | 316.0062379 | 0.067666126 | 6 | 0.049092587 | 1.666667 | 2 |
| F10 | 0 | 0.060507246 | 3 | 2.08E-04 | 2 | 3 |
| F3 | 92.2062389 | 0.061783204 | 6 | 6.00E-04 | 2.333333 | 4.933333333 |
| F7 | 0.666666667 | 0.060529177 | 4 | 2.22E-04 | 2.5 | 3.666666667 |
| THBD | 11.89177489 | 0.060529177 | 4 | 2.35E-04 | 2 | 2.833333333 |
| PLAT | 17.74584068 | 0.061306902 | 3 | 4.28E-04 | 1.333333 | 2 |
| FABP5 | 0 | 0.058844257 | 1 | 4.80E-05 | 0 | 0 |
| PPARD | 308 | 0.062220566 | 2 | 7.48E-04 | 0 | 0 |
| NR3C1 | 205.8054019 | 0.068667763 | 6 | 0.062714621 | 2.333333 | 3.6 |
| NFATC1 | 74.81235356 | 0.067230274 | 6 | 0.053319991 | 2.333333 | 2.933333333 |
| HSF1 | 0 | 0.065929728 | 2 | 0.019685987 | 1 | 2 |
| SPP1 | 396.8140605 | 0.065567334 | 4 | 0.005466864 | 0.5 | 0.833333333 |
| MGAM | 308 | 0.060071942 | 2 | 2.05E-04 | 0 | 0 |
| HMOX1 | 0 | 0.064304967 | 2 | 0.005052179 | 1 | 2 |
| NQO1 | 422.2699167 | 0.0668 | 4 | 0.025025778 | 0.5 | 1.333333333 |
| KCNH2 | 57.72759094 | 0.065490196 | 2 | 0.012210777 | 0 | 0 |
| HTR2A | 0 | 0.054646597 | 1 | 2.52E-07 | 0 | 0 |
| SLC6A4 | 308 | 0.05754652 | 2 | 3.77E-06 | 0 | 0 |
| SELE | 0 | 0.063522252 | 2 | 0.005205893 | 1 | 2 |
| IRF1 | 24.05348684 | 0.067068273 | 4 | 0.036148779 | 1 | 1.333333333 |
| IGF2 | 18.6937875 | 0.064304967 | 2 | 0.002245004 | 0 | 0 |
| INSR | 47.33896741 | 0.064230769 | 3 | 0.004455861 | 0 | 0 |
| RXRA | 721.8650418 | 0.065955766 | 6 | 0.011597208 | 1 | 1.633333333 |
| MMP9 | 1438.861267 | 0.067365873 | 10 | 0.032889567 | 2.2 | 4.958333333 |
| PPARA | 97.20583371 | 0.067068273 | 4 | 0.025725324 | 1.5 | 2 |
| LYZ | 0 | 0.059964093 | 1 | 1.36E-04 | 0 | 0 |
| MPO | 308 | 0.063473964 | 2 | 0.002116825 | 0 | 0 |
| PYGM | 0 | 0.056918882 | 1 | 1.32E-05 | 0 | 0 |
| MMP1 | 1 | 0.063473964 | 3 | 0.002773019 | 1.333333 | 2 |
| MMP2 | 57.64078732 | 0.065618861 | 4 | 0.007735577 | 1.5 | 2.166666667 |
| MMP3 | 12.70282998 | 0.063935681 | 3 | 0.002636435 | 1.333333 | 2 |
| NR3C2 | 0 | 0.065107212 | 2 | 0.008086651 | 1 | 2 |
| ODC1 | 0 | 0.062923888 | 1 | 0.001608595 | 0 | 0 |
| OPRD1 | 0 | 0.005988024 | 1 | 0 | 0 | 0 |
| OPRM1 | 0 | 0.005988024 | 1 | 0 | 0 | 0 |
| TOP1 | 0 | 0.066693291 | 2 | 0.024491949 | 1 | 2 |
| PLAU | 0 | 0.061015711 | 1 | 3.75E-04 | 0 | 0 |
| SLC2A4 | 0 | 0.064503669 | 1 | 0.003569704 | 0 | 0 |
| PTGES | 0 | 0.065132605 | 2 | 0.003925062 | 1 | 2 |
