# Supplementary material for: Elucidating the mechanism of Buyang Huanwu Decoction in the treatment of ischemic stroke: A network pharmacology and molecular docking study
Source: Medicine (Baltimore). 2026 Jul 17;105(29):e49736. doi: 10.1097/MD.0000000000049736 (PMC13384647; doi:10.1097/MD.0000000000049736)
Supplement: Supplementary file 8 [file medi-105-e49736-s008.docx]

S 8. Sensitivity analysis of core targets

| **Threshold (quantile)** | **Cutoff** | **Number of core targets** | **Target name** |
| --- | --- | --- | --- |
| 50% | BC: 18.11743 CC: 0.51310 DC: 8.50000 EC: 0.10060 LAC: 4.77500 NC: 5.94167 | 16 | TP53，JUN，AKT1，TNF，IL6，ESR1，RELA，MAPK1，HSP90AA1，BCL2，CASP3，FOS，IL1B，MYC，CTNNB1，BCL2L1， |
| 75% | BC: 425.89728 CC: 0.06811  DC: 10.00000 EC: 0.07593 LAC: 4.47115  NC: 5.54251 | 16 | ESR1，HSP90AA1，AKT1，MAPK1，TP53，CASP3，JUN，BCL2，TNF，CTNNB1，IL6，CYCS，IL1B，CCL2，RELA，FOS， |
| **Overlap rate=13/16*100%=81.25%** | | | |
