# Supplementary material for: Elucidating the mechanism of Buyang Huanwu Decoction in the treatment of ischemic stroke: A network pharmacology and molecular docking study
Source: Medicine (Baltimore). 2026 Jul 17;105(29):e49736. doi: 10.1097/MD.0000000000049736 (PMC13384647; doi:10.1097/MD.0000000000049736)
Supplement: Supplementary file 9 [file medi-105-e49736-s009.docx]

**S 9.** Functional Enrichment Analysis of GO Terms Using DAVID: Raw Data.

| **Class** | **Term** | **Count** | **Gene ratio** | **PValue** | **Genes** | **FDR** |
| --- | --- | --- | --- | --- | --- | --- |
| MF | GO:0042277~peptide binding | 3 | 1.578947 | 0.098629 | RXRA, PPARG, RELA | 0.352693 |
| BP | GO:0050877~neurological system process | 3 | 1.578947 | 0.0985 | GABRA1, CHRNA2, CHRNA7 | 0.31891 |
| BP | GO:0006968~cellular defense response | 3 | 1.578947 | 0.0985 | NCF1, LBP, RELA | 0.31891 |
| BP | GO:0071320~cellular response to cAMP | 3 | 1.578947 | 0.0985 | CYP1B1, AHR, PIK3CG | 0.31891 |
| MF | GO:0004222~metalloendopeptidase activity | 4 | 2.105263 | 0.098332 | MMP1, MMP2, MMP3, MMP9 | 0.352693 |
| CC | GO:1905370~serine-type endopeptidase complex | 2 | 1.052632 | 0.096795 | THBD, PLAU | 0.316328 |
| CC | GO:0035631~CD40 receptor complex | 2 | 1.052632 | 0.096795 | IKBKB, CHUK | 0.316328 |
| MF | GO:0043295~glutathione binding | 2 | 1.052632 | 0.095722 | GSTM1, PTGES | 0.346685 |
| MF | GO:1990763~arrestin family protein binding | 2 | 1.052632 | 0.095722 | CHRM2, DRD1 | 0.346685 |
| MF | GO:0001094~TFIID-class transcription factor binding | 2 | 1.052632 | 0.095722 | AHR, TP53 | 0.346685 |
| BP | GO:0032092~positive regulation of protein binding | 3 | 1.578947 | 0.095444 | GSK3B, APP, CAV1 | 0.310194 |
| CC | GO:0001673~male germ cell nucleus | 3 | 1.578947 | 0.094119 | PCNA, CDK2, TOP1 | 0.314059 |
| CC | GO:0016323~basolateral plasma membrane | 6 | 3.157895 | 0.09395 | HSP90AA1, CAV1, ERBB2, CTNNB1, EGFR, ADRA2A | 0.314059 |
| CC | GO:0031965~nuclear membrane | 6 | 3.157895 | 0.09395 | CCND1, ALOX5, BCL2, ADRA1B, EGFR, BCL2L1 | 0.314059 |
| BP | GO:1901857~positive regulation of cellular respiration | 2 | 1.052632 | 0.093949 | IL4, IFNG | 0.305721 |
| BP | GO:0060068~vagina development | 2 | 1.052632 | 0.093949 | BAX, ESR1 | 0.305721 |
| BP | GO:0001783~B cell apoptotic process | 2 | 1.052632 | 0.093949 | BCL2, BAX | 0.305721 |
| BP | GO:2000288~positive regulation of myoblast proliferation | 2 | 1.052632 | 0.093949 | CTNNB1, PPARD | 0.305721 |
| BP | GO:0033629~negative regulation of cell adhesion mediated by integrin | 2 | 1.052632 | 0.093949 | SERPINE1, CYP1B1 | 0.305721 |
| BP | GO:0002262~myeloid cell homeostasis | 2 | 1.052632 | 0.093949 | BAX, SOD1 | 0.305721 |
| BP | GO:0032886~regulation of microtubule-based process | 2 | 1.052632 | 0.093949 | GSK3B, ERBB2 | 0.305721 |
| BP | GO:0031666~positive regulation of lipopolysaccharide-mediated signaling pathway | 2 | 1.052632 | 0.093949 | PRKCA, CD14 | 0.305721 |
| BP | GO:0060371~regulation of atrial cardiac muscle cell membrane depolarization | 2 | 1.052632 | 0.093949 | GJA1, SCN5A | 0.305721 |
| BP | GO:0002819~regulation of adaptive immune response | 2 | 1.052632 | 0.093949 | IRF1, AHR | 0.305721 |
| BP | GO:0070723~response to cholesterol | 2 | 1.052632 | 0.093949 | F7, TGFB1 | 0.305721 |
| BP | GO:0042359~vitamin D metabolic process | 2 | 1.052632 | 0.093949 | CYP1A1, CYP3A4 | 0.305721 |
| BP | GO:1902065~response to L-glutamate | 2 | 1.052632 | 0.093949 | PCNA, TNF | 0.305721 |
| BP | GO:0061298~retina vasculature development in camera-type eye | 2 | 1.052632 | 0.093949 | TGFB1, HIF1A | 0.305721 |
| BP | GO:2000279~negative regulation of DNA biosynthetic process | 2 | 1.052632 | 0.093949 | CDKN1A, CHEK1 | 0.305721 |
| BP | GO:2000320~negative regulation of T-helper 17 cell differentiation | 2 | 1.052632 | 0.093949 | IL4, IL2 | 0.305721 |
| BP | GO:0019065~receptor-mediated endocytosis of virus by host cell | 2 | 1.052632 | 0.093949 | DPP4, CAV1 | 0.305721 |
| BP | GO:0014912~negative regulation of smooth muscle cell migration | 2 | 1.052632 | 0.093949 | IGFBP3, SERPINE1 | 0.305721 |
| BP | GO:0009566~fertilization | 3 | 1.578947 | 0.092417 | BAX, DUOX2, BCL2L1 | 0.305721 |
| BP | GO:0031648~protein destabilization | 3 | 1.578947 | 0.089419 | CDKN2A, MDM2, SIRT1 | 0.298178 |
| CC | GO:0098981~cholinergic synapse | 2 | 1.052632 | 0.088394 | CHRM2, CHRM1 | 0.304577 |
| MF | GO:0043167~ion binding | 2 | 1.052632 | 0.086575 | RXRA, SPP1 | 0.319706 |
| BP | GO:0048013~ephrin receptor signaling pathway | 3 | 1.578947 | 0.08645 | RASA1, MMP2, MMP9 | 0.288656 |
| BP | GO:0051603~proteolysis involved in cellular protein catabolic process | 3 | 1.578947 | 0.08645 | CASP8, HSPA5, MDM2 | 0.288656 |
| BP | GO:0009968~negative regulation of signal transduction | 3 | 1.578947 | 0.08645 | GSK3B, PTPN1, IGFBP3 | 0.288656 |
| BP | GO:0008542~visual learning | 3 | 1.578947 | 0.08645 | APP, DRD1, HIF1A | 0.288656 |
| MF | GO:0001222~transcription corepressor binding | 3 | 1.578947 | 0.08634 | STAT1, CTNNB1, ESR1 | 0.319706 |
| BP | GO:0005975~carbohydrate metabolic process | 5 | 2.631579 | 0.085224 | GSK3B, MGAM, INSR, AKR1B1, SLC2A4 | 0.286053 |
| BP | GO:0051901~positive regulation of mitochondrial depolarization | 2 | 1.052632 | 0.084963 | PARP1, KDR | 0.285552 |
| BP | GO:0043268~positive regulation of potassium ion transport | 2 | 1.052632 | 0.084963 | DRD1, ADRA2A | 0.285552 |
| BP | GO:1990314~cellular response to insulin-like growth factor stimulus | 2 | 1.052632 | 0.084963 | CCNA2, TGFB1 | 0.285552 |
| BP | GO:0015872~dopamine transport | 2 | 1.052632 | 0.084963 | DRD1, SLC6A3 | 0.285552 |
| BP | GO:0032364~oxygen homeostasis | 2 | 1.052632 | 0.084963 | EGLN1, HIF1A | 0.285552 |
| BP | GO:0002752~cell surface pattern recognition receptor signaling pathway | 2 | 1.052632 | 0.084963 | CD14, LBP | 0.285552 |
| BP | GO:0046209~nitric oxide metabolic process | 2 | 1.052632 | 0.084963 | CYP1A1, PPARA | 0.285552 |
| BP | GO:2000669~negative regulation of dendritic cell apoptotic process | 2 | 1.052632 | 0.084963 | BCL2, BCL2L1 | 0.285552 |
| BP | GO:0019371~cyclooxygenase pathway | 2 | 1.052632 | 0.084963 | PTGS2, PTGS1 | 0.285552 |
| BP | GO:0046621~negative regulation of organ growth | 2 | 1.052632 | 0.084963 | PTEN, SLC6A4 | 0.285552 |
| BP | GO:0042053~regulation of dopamine metabolic process | 2 | 1.052632 | 0.084963 | DRD1, SLC6A3 | 0.285552 |
| BP | GO:2000242~negative regulation of reproductive process | 2 | 1.052632 | 0.084963 | BCL2L1, SOD1 | 0.285552 |
| BP | GO:0032287~peripheral nervous system myelin maintenance | 2 | 1.052632 | 0.084963 | AKT1, SOD1 | 0.285552 |
| BP | GO:0043267~negative regulation of potassium ion transport | 2 | 1.052632 | 0.084963 | NOS3, HTR2A | 0.285552 |
| BP | GO:0086014~atrial cardiac muscle cell action potential | 2 | 1.052632 | 0.084963 | GJA1, SCN5A | 0.285552 |
| BP | GO:0034351~negative regulation of glial cell apoptotic process | 2 | 1.052632 | 0.084963 | CCL2, PRKCA | 0.285552 |
| BP | GO:0070168~negative regulation of biomineral tissue development | 2 | 1.052632 | 0.084963 | TGFB1, NOS3 | 0.285552 |
| BP | GO:0009308~amine metabolic process | 2 | 1.052632 | 0.084963 | VCAM1, CYP1A1 | 0.285552 |
| BP | GO:0060253~negative regulation of glial cell proliferation | 2 | 1.052632 | 0.084963 | RB1, TP53 | 0.285552 |
| BP | GO:0002232~leukocyte chemotaxis involved in inflammatory response | 2 | 1.052632 | 0.084963 | ALOX5, LBP | 0.285552 |
| BP | GO:2000553~positive regulation of T-helper 2 cell cytokine production | 2 | 1.052632 | 0.084963 | IL4, IL6 | 0.285552 |
| BP | GO:0038003~opioid receptor signaling pathway | 2 | 1.052632 | 0.084963 | OPRD1, OPRM1 | 0.285552 |
| BP | GO:1901844~regulation of cell communication by electrical coupling involved in cardiac conduction | 2 | 1.052632 | 0.084963 | CAV1, CALM3 | 0.285552 |
| BP | GO:1900027~regulation of ruffle assembly | 2 | 1.052632 | 0.084963 | CAV1, ICAM1 | 0.285552 |
| BP | GO:0042789~mRNA transcription from RNA polymerase II promoter | 3 | 1.578947 | 0.083512 | NCOA1, RXRA, PPARG | 0.285552 |
| BP | GO:0034198~cellular response to amino acid starvation | 3 | 1.578947 | 0.083512 | CDKN1A, MAPK8, MAPK1 | 0.285552 |
| BP | GO:0007188~adenylate cyclase-modulating G-protein coupled receptor signaling pathway | 3 | 1.578947 | 0.083512 | CHRM2, ADRB2, ADRA1B | 0.285552 |
| MF | GO:0005216~ion channel activity | 3 | 1.578947 | 0.083346 | KCNH2, CHRNA7, KCNMA1 | 0.311856 |
| BP | GO:0050714~positive regulation of protein secretion | 3 | 1.578947 | 0.080606 | IL1A, ACHE, TGFB1 | 0.280861 |
| BP | GO:0021762~substantia nigra development | 3 | 1.578947 | 0.080606 | MAOB, HSPA5, CALM3 | 0.280861 |
| BP | GO:0021549~cerebellum development | 3 | 1.578947 | 0.080606 | NCOA1, SCN5A, TP53 | 0.280861 |
| CC | GO:0044294~dendritic growth cone | 2 | 1.052632 | 0.079917 | HSP90AA1, MAP2 | 0.278391 |
| CC | GO:0005916~fascia adherens | 2 | 1.052632 | 0.079917 | GJA1, CTNNB1 | 0.278391 |
| CC | GO:0016600~flotillin complex | 2 | 1.052632 | 0.079917 | CTNNB1, SLC6A3 | 0.278391 |
| CC | GO:0098590~plasma membrane region | 2 | 1.052632 | 0.079917 | ERBB2, EGFR | 0.278391 |
| CC | GO:0043231~intracellular membrane-bounded organelle | 14 | 7.368421 | 0.079291 | EGLN1, HSPA5, CYP3A4, MPO, ESR2, HK2, PTGS1, GJA1, CYP2B6, CHEK1, CYP1A1, CYP1B1, OLR1, PPARG | 0.278391 |
| BP | GO:0030182~neuron differentiation | 5 | 2.631579 | 0.078378 | CCND1, CASP3, ERBB2, MET, RUNX2 | 0.274215 |
| BP | GO:0045739~positive regulation of DNA repair | 3 | 1.578947 | 0.077733 | PCNA, SIRT1, EGFR | 0.272331 |
| BP | GO:0070527~platelet aggregation | 3 | 1.578947 | 0.077733 | HSPB1, PIK3CG, SLC6A4 | 0.272331 |
| BP | GO:0042100~B cell proliferation | 3 | 1.578947 | 0.077733 | IL10, CD40LG, BCL2 | 0.272331 |
| BP | GO:0009411~response to UV | 3 | 1.578947 | 0.077733 | CASP7, MAPK8, CASP3 | 0.272331 |
| MF | GO:0097200~cysteine-type endopeptidase activity involved in execution phase of apoptosis | 2 | 1.052632 | 0.077336 | CASP7, CASP3 | 0.2913 |
| MF | GO:0034618~arginine binding | 2 | 1.052632 | 0.077336 | NOS2, NOS3 | 0.2913 |
| MF | GO:0031995~insulin-like growth factor II binding | 2 | 1.052632 | 0.077336 | INSR, IGFBP3 | 0.2913 |
| BP | GO:0006351~transcription, DNA-templated | 4 | 2.105263 | 0.077316 | CCNA2, CDK2, E2F1, RELA | 0.272331 |
| BP | GO:0000209~protein polyubiquitination | 5 | 2.631579 | 0.077035 | NQO1, CDKN2A, MDM2, BCL2, CTNNB1 | 0.271745 |
| BP | GO:0034393~positive regulation of smooth muscle cell apoptotic process | 2 | 1.052632 | 0.075889 | IFNG, CDKN2A | 0.268072 |
| BP | GO:0042092~type 2 immune response | 2 | 1.052632 | 0.075889 | IL10, IL4 | 0.268072 |
| BP | GO:0009743~response to carbohydrate | 2 | 1.052632 | 0.075889 | NQO1, IL1B | 0.268072 |
| BP | GO:0008228~opsonization | 2 | 1.052632 | 0.075889 | CRP, LBP | 0.268072 |
| BP | GO:0002070~epithelial cell maturation | 2 | 1.052632 | 0.075889 | KDR, AKR1B1 | 0.268072 |
| BP | GO:0060373~regulation of ventricular cardiac muscle cell membrane depolarization | 2 | 1.052632 | 0.075889 | GJA1, SCN5A | 0.268072 |
| BP | GO:0060740~prostate gland epithelium morphogenesis | 2 | 1.052632 | 0.075889 | AR, MMP2 | 0.268072 |
| BP | GO:0002674~negative regulation of acute inflammatory response | 2 | 1.052632 | 0.075889 | IL4, PPARG | 0.268072 |
| BP | GO:1903071~positive regulation of ER-associated ubiquitin-dependent protein catabolic process | 2 | 1.052632 | 0.075889 | CAV1, NFE2L2 | 0.268072 |
| BP | GO:1903896~positive regulation of IRE1-mediated unfolded protein response | 2 | 1.052632 | 0.075889 | PTPN1, BAX | 0.268072 |
| BP | GO:0043504~mitochondrial DNA repair | 2 | 1.052632 | 0.075889 | PARP1, TP53 | 0.268072 |
| BP | GO:0038134~ERBB2-EGFR signaling pathway | 2 | 1.052632 | 0.075889 | ERBB2, EGFR | 0.268072 |
| BP | GO:0010224~response to UV-B | 2 | 1.052632 | 0.075889 | BCL2, RELA | 0.268072 |
| BP | GO:0098815~modulation of excitatory postsynaptic potential | 2 | 1.052632 | 0.075889 | APP, CHRNA7 | 0.268072 |
| CC | GO:0000781~chromosome, telomeric region | 5 | 2.631579 | 0.075762 | PCNA, PARP1, CHEK2, CHEK1, CDK2 | 0.278391 |
| BP | GO:0048863~stem cell differentiation | 3 | 1.578947 | 0.074893 | MAPK14, ESR1, RUNX2 | 0.268072 |
| BP | GO:0007259~JAK-STAT cascade | 3 | 1.578947 | 0.074893 | IFNG, STAT1, CCL2 | 0.268072 |
| BP | GO:0060395~SMAD protein signal transduction | 3 | 1.578947 | 0.074893 | JUN, FOS, RUNX2 | 0.268072 |
| BP | GO:0006836~neurotransmitter transport | 3 | 1.578947 | 0.074893 | SLC6A2, SLC6A3, SLC6A4 | 0.268072 |
| BP | GO:0048286~lung alveolus development | 3 | 1.578947 | 0.074893 | TGFB1, KDR, PGR | 0.268072 |
| BP | GO:0006865~amino acid transport | 3 | 1.578947 | 0.074893 | SLC6A2, SLC6A3, SLC6A4 | 0.268072 |
| BP | GO:0043161~proteasome-mediated ubiquitin-dependent protein catabolic process | 6 | 3.157895 | 0.07344 | GSK3B, MDM2, CTNNB1, AKT1, SIRT1, NFE2L2 | 0.26678 |
| CC | GO:0016529~sarcoplasmic reticulum | 3 | 1.578947 | 0.072251 | SLC2A4, XDH, HK2 | 0.269455 |
| BP | GO:0032526~response to retinoic acid | 3 | 1.578947 | 0.072088 | NCOA1, RXRA, MMP2 | 0.262239 |
| CC | GO:0008385~IkappaB kinase complex | 2 | 1.052632 | 0.071361 | IKBKB, CHUK | 0.269301 |
| CC | GO:0032279~asymmetric synapse | 2 | 1.052632 | 0.071361 | CHRM2, GRIA2 | 0.269301 |
| BP | GO:0001658~branching involved in ureteric bud morphogenesis | 3 | 1.578947 | 0.069317 | MYC, BCL2, CTNNB1 | 0.25252 |
| MF | GO:0048156~tau protein binding | 3 | 1.578947 | 0.068887 | GSK3B, HSP90AA1, MAP2 | 0.264771 |
| MF | GO:0050661~NADP binding | 3 | 1.578947 | 0.068887 | NOS2, NOS3, NOX5 | 0.264771 |
| BP | GO:0035264~multicellular organism growth | 4 | 2.105263 | 0.068626 | AR, COL3A1, TP53, DUOX2 | 0.250359 |
| MF | GO:0016907~G-protein coupled acetylcholine receptor activity | 2 | 1.052632 | 0.068004 | CHRM2, CHRM1 | 0.264771 |
| MF | GO:0035240~dopamine binding | 2 | 1.052632 | 0.068004 | DRD1, SLC6A3 | 0.264771 |
| MF | GO:0048273~mitogen-activated protein kinase p38 binding | 2 | 1.052632 | 0.068004 | NFATC1, MAPK14 | 0.264771 |
| MF | GO:0050693~LBD domain binding | 2 | 1.052632 | 0.068004 | RXRA, PPARG | 0.264771 |
| BP | GO:0006631~fatty acid metabolic process | 4 | 2.105263 | 0.066945 | CYP1A1, PPARG, PPARA, PPARD | 0.244574 |
| BP | GO:1900076~regulation of cellular response to insulin stimulus | 2 | 1.052632 | 0.066726 | NCOA1, PPARG | 0.244121 |
| BP | GO:0034405~response to fluid shear stress | 2 | 1.052632 | 0.066726 | NOS3, AKT1 | 0.244121 |
| BP | GO:0019217~regulation of fatty acid metabolic process | 2 | 1.052632 | 0.066726 | CAV1, PPARA | 0.244121 |
| BP | GO:0002719~negative regulation of cytokine production involved in immune response | 2 | 1.052632 | 0.066726 | IL10, TNF | 0.244121 |
| BP | GO:0007077~mitotic nuclear envelope disassembly | 2 | 1.052632 | 0.066726 | PRKCB, PRKCA | 0.244121 |
| BP | GO:1905144~response to acetylcholine | 2 | 1.052632 | 0.066726 | CHRNA2, CHRNA7 | 0.244121 |
| BP | GO:0090594~inflammatory response to wounding | 2 | 1.052632 | 0.066726 | IL6, TNF | 0.244121 |
| BP | GO:0014012~peripheral nervous system axon regeneration | 2 | 1.052632 | 0.066726 | MMP2, APOD | 0.244121 |
| BP | GO:0002933~lipid hydroxylation | 2 | 1.052632 | 0.066726 | CYP1A1, CYP3A4 | 0.244121 |
| BP | GO:1904037~positive regulation of epithelial cell apoptotic process | 2 | 1.052632 | 0.066726 | BAX, HMOX1 | 0.244121 |
| BP | GO:0072655~establishment of protein localization to mitochondrion | 2 | 1.052632 | 0.066726 | AKT1, HK2 | 0.244121 |
| BP | GO:0010883~regulation of lipid storage | 2 | 1.052632 | 0.066726 | PPARA, SIRT1 | 0.244121 |
| BP | GO:0009624~response to nematode | 2 | 1.052632 | 0.066726 | CYP1A1, PTGS2 | 0.244121 |
| BP | GO:1901331~positive regulation of odontoblast differentiation | 2 | 1.052632 | 0.066726 | SERPINE1, CTNNB1 | 0.244121 |
| BP | GO:0031065~positive regulation of histone deacetylation | 2 | 1.052632 | 0.066726 | TGFB1, SIRT1 | 0.244121 |
| BP | GO:0031017~exocrine pancreas development | 2 | 1.052632 | 0.066726 | INSR, IGF2 | 0.244121 |
| BP | GO:0034440~lipid oxidation | 2 | 1.052632 | 0.066726 | ALOX5, ALOX12 | 0.244121 |
| BP | GO:0031394~positive regulation of prostaglandin biosynthetic process | 2 | 1.052632 | 0.066726 | IL1B, PTGS2 | 0.244121 |
| BP | GO:0032411~positive regulation of transporter activity | 2 | 1.052632 | 0.066726 | RXRA, PON1 | 0.244121 |
| BP | GO:1903944~negative regulation of hepatocyte apoptotic process | 2 | 1.052632 | 0.066726 | RB1, PPARA | 0.244121 |
| BP | GO:0002637~regulation of immunoglobulin production | 2 | 1.052632 | 0.066726 | CD40LG, TNF | 0.244121 |
| BP | GO:0044321~response to leptin | 2 | 1.052632 | 0.066726 | CCND1, SIRT1 | 0.244121 |
| BP | GO:0071219~cellular response to molecule of bacterial origin | 2 | 1.052632 | 0.066726 | CD14, AHR | 0.244121 |
| BP | GO:0007435~salivary gland morphogenesis | 2 | 1.052632 | 0.066726 | TGFB1, EGFR | 0.244121 |
| BP | GO:0051620~norepinephrine uptake | 2 | 1.052632 | 0.066726 | SLC6A2, SLC6A3 | 0.244121 |
| BP | GO:0060440~trachea formation | 2 | 1.052632 | 0.066726 | MAPK1, CTNNB1 | 0.244121 |
| BP | GO:0086010~membrane depolarization during action potential | 2 | 1.052632 | 0.066726 | KCNH2, SCN5A | 0.244121 |
| BP | GO:0015874~norepinephrine transport | 2 | 1.052632 | 0.066726 | SLC6A2, SLC6A3 | 0.244121 |
| BP | GO:0030213~hyaluronan biosynthetic process | 2 | 1.052632 | 0.066726 | IL1B, HAS2 | 0.244121 |
| BP | GO:0071321~cellular response to cGMP | 2 | 1.052632 | 0.066726 | PDE3A, SLC6A4 | 0.244121 |
| BP | GO:0035249~synaptic transmission, glutamatergic | 3 | 1.578947 | 0.066584 | GRIA2, DRD1, NR3C1 | 0.244121 |
| BP | GO:0042098~T cell proliferation | 3 | 1.578947 | 0.066584 | GJA1, TGFB1, PIK3CG | 0.244121 |
| BP | GO:1901796~regulation of signal transduction by p53 class mediator | 3 | 1.578947 | 0.066584 | CHEK2, CHEK1, AKT1 | 0.244121 |
| BP | GO:0030501~positive regulation of bone mineralization | 3 | 1.578947 | 0.063887 | RXRA, ALOX5, ADRB2 | 0.244121 |
| BP | GO:0032733~positive regulation of interleukin-10 production | 3 | 1.578947 | 0.063887 | IL4, IL6, CD40LG | 0.244121 |
| BP | GO:1900016~negative regulation of cytokine production involved in inflammatory response | 3 | 1.578947 | 0.063887 | APOD, F2, PPARA | 0.244121 |
| CC | GO:0031264~death-inducing signaling complex | 2 | 1.052632 | 0.062725 | CASP8, CASP3 | 0.242487 |
| MF | GO:0008013~beta-catenin binding | 4 | 2.105263 | 0.061642 | GSK3B, AR, GJA1, ESR1 | 0.247005 |
| BP | GO:0007405~neuroblast proliferation | 3 | 1.578947 | 0.061229 | CTNNB1, HIF1A, TP53 | 0.236156 |
| BP | GO:0006749~glutathione metabolic process | 3 | 1.578947 | 0.061229 | GSTM1, GSTA2, SOD1 | 0.236156 |
| BP | GO:0043537~negative regulation of blood vessel endothelial cell migration | 3 | 1.578947 | 0.061229 | TGFB1, PPARG, TNF | 0.236156 |
| BP | GO:0019233~sensory perception of pain | 3 | 1.578947 | 0.061229 | CCL2, OPRM1, PTGES | 0.236156 |
| CC | GO:0043197~dendritic spine | 5 | 2.631579 | 0.059617 | PPP3CA, GRIA2, APP, PTEN, DRD1 | 0.233318 |
| CC | GO:0098839~postsynaptic density membrane | 4 | 2.105263 | 0.059016 | OPRD1, GRIA2, CHRM1, ADRA2A | 0.233318 |
| CC | GO:0030141~secretory granule | 4 | 2.105263 | 0.059016 | TGFB1, IL1B, PLAT, MPO | 0.233318 |
| BP | GO:0009725~response to hormone | 3 | 1.578947 | 0.05861 | NQO1, NOS2, NOS3 | 0.227425 |
| BP | GO:0001974~blood vessel remodeling | 3 | 1.578947 | 0.05861 | NOS3, MDM2, BAX | 0.227425 |
| BP | GO:0086091~regulation of heart rate by cardiac conduction | 3 | 1.578947 | 0.05861 | KCNH2, CAV1, SCN5A | 0.227425 |
| BP | GO:0071526~semaphorin-plexin signaling pathway | 3 | 1.578947 | 0.05861 | ERBB2, KDR, MET | 0.227425 |
| MF | GO:0051379~epinephrine binding | 2 | 1.052632 | 0.058579 | ADRA2C, ADRA2A | 0.236407 |
| MF | GO:0051525~NFAT protein binding | 2 | 1.052632 | 0.058579 | MAPK14, PPARA | 0.236407 |
| MF | GO:0032795~heterotrimeric G-protein binding | 2 | 1.052632 | 0.058579 | DRD1, ADRA2A | 0.236407 |
| MF | GO:0004064~arylesterase activity | 2 | 1.052632 | 0.058579 | CA2, PON1 | 0.236407 |
| MF | GO:0004985~opioid receptor activity | 2 | 1.052632 | 0.058579 | OPRD1, OPRM1 | 0.236407 |
| MF | GO:0033612~receptor serine/threonine kinase binding | 2 | 1.052632 | 0.058579 | OPRD1, MDM2 | 0.236407 |
| MF | GO:0001530~lipopolysaccharide binding | 3 | 1.578947 | 0.057998 | CD14, LBP, F2 | 0.236407 |
| BP | GO:0072656~maintenance of protein location in mitochondrion | 2 | 1.052632 | 0.057472 | AKT1, HK2 | 0.224368 |
| BP | GO:0033092~positive regulation of immature T cell proliferation in thymus | 2 | 1.052632 | 0.057472 | IL1A, IL1B | 0.224368 |
| BP | GO:0042596~fear response | 2 | 1.052632 | 0.057472 | ADRB1, ADRA2A | 0.224368 |
| BP | GO:0060574~intestinal epithelial cell maturation | 2 | 1.052632 | 0.057472 | CDKN1A, HIF1A | 0.224368 |
| BP | GO:0007617~mating behavior | 2 | 1.052632 | 0.057472 | APP, DRD1 | 0.224368 |
| BP | GO:0010891~negative regulation of sequestering of triglyceride | 2 | 1.052632 | 0.057472 | PPARG, PPARA | 0.224368 |
| BP | GO:1903038~negative regulation of leukocyte cell-cell adhesion | 2 | 1.052632 | 0.057472 | AKT1, PPARA | 0.224368 |
| BP | GO:1904894~positive regulation of STAT cascade | 2 | 1.052632 | 0.057472 | IL6, TGFB1 | 0.224368 |
| BP | GO:0010700~negative regulation of norepinephrine secretion | 2 | 1.052632 | 0.057472 | ADRA2C, ADRA2A | 0.224368 |
| BP | GO:1901984~negative regulation of protein acetylation | 2 | 1.052632 | 0.057472 | GSK3B, SIRT1 | 0.224368 |
| BP | GO:0031536~positive regulation of exit from mitosis | 2 | 1.052632 | 0.057472 | TGFB1, BIRC5 | 0.224368 |
| BP | GO:0010716~negative regulation of extracellular matrix disassembly | 2 | 1.052632 | 0.057472 | DPP4, TGFB1 | 0.224368 |
| BP | GO:1903721~positive regulation of I-kappaB phosphorylation | 2 | 1.052632 | 0.057472 | AKT1, TNF | 0.224368 |
| BP | GO:0097398~cellular response to interleukin-17 | 2 | 1.052632 | 0.057472 | CXCL10, IL1B | 0.224368 |
| BP | GO:0007253~cytoplasmic sequestering of NF-kappaB | 2 | 1.052632 | 0.057472 | IL10, NFKBIA | 0.224368 |
| BP | GO:0051712~positive regulation of killing of cells of other organism | 2 | 1.052632 | 0.057472 | IFNG, NOS2 | 0.224368 |
| BP | GO:0033088~negative regulation of immature T cell proliferation in thymus | 2 | 1.052632 | 0.057472 | CDKN2A, ERBB2 | 0.224368 |
| BP | GO:0036003~positive regulation of transcription from RNA polymerase II promoter in response to stress | 2 | 1.052632 | 0.057472 | TP53, NFE2L2 | 0.224368 |
| BP | GO:0046813~receptor-mediated virion attachment to host cell | 2 | 1.052632 | 0.057472 | DPP4, ICAM1 | 0.224368 |
| BP | GO:0045760~positive regulation of action potential | 2 | 1.052632 | 0.057472 | SCN5A, TNF | 0.224368 |
| BP | GO:0051902~negative regulation of mitochondrial depolarization | 2 | 1.052632 | 0.057472 | ALB, BCL2 | 0.224368 |
| BP | GO:0071455~cellular response to hyperoxia | 2 | 1.052632 | 0.057472 | CAV1, PPARG | 0.224368 |
| BP | GO:0006527~arginine catabolic process | 2 | 1.052632 | 0.057472 | NOS2, NOS3 | 0.224368 |
| BP | GO:0009750~response to fructose | 2 | 1.052632 | 0.057472 | PTGS2, TNF | 0.224368 |
| BP | GO:1903347~negative regulation of bicellular tight junction assembly | 2 | 1.052632 | 0.057472 | IKBKB, TNF | 0.224368 |
| BP | GO:0043619~regulation of transcription from RNA polymerase II promoter in response to oxidative stress | 2 | 1.052632 | 0.057472 | HMOX1, HIF1A | 0.224368 |
| BP | GO:0046321~positive regulation of fatty acid oxidation | 2 | 1.052632 | 0.057472 | PPARG, PPARA | 0.224368 |
| BP | GO:0051583~dopamine uptake involved in synaptic transmission | 2 | 1.052632 | 0.057472 | SLC6A2, SLC6A3 | 0.224368 |
| BP | GO:0030183~B cell differentiation | 4 | 2.105263 | 0.057268 | IL10, IL4, VCAM1, CD40LG | 0.224368 |
| CC | GO:0043198~dendritic shaft | 3 | 1.578947 | 0.057137 | APP, MAP2, HTR2A | 0.232209 |
| BP | GO:0032008~positive regulation of TOR signaling | 3 | 1.578947 | 0.056032 | F7, F10, F3 | 0.224368 |
| BP | GO:0032007~negative regulation of TOR signaling | 3 | 1.578947 | 0.056032 | GSK3B, HIF1A, SIRT1 | 0.224368 |
| BP | GO:0033077~T cell differentiation in thymus | 3 | 1.578947 | 0.056032 | BCL2, CTNNB1, TP53 | 0.224368 |
| BP | GO:2001237~negative regulation of extrinsic apoptotic signaling pathway | 3 | 1.578947 | 0.056032 | IL4, AR, RELA | 0.224368 |
| MF | GO:0044325~ion channel binding | 5 | 2.631579 | 0.054549 | HSP90AA1, CAV1, CTNNB1, CALM3, SCN5A | 0.231731 |
| CC | GO:0097441~basilar dendrite | 2 | 1.052632 | 0.05401 | MAPK8, MAP2 | 0.222353 |
| CC | GO:0097443~sorting endosome | 2 | 1.052632 | 0.05401 | PTPN1, KDR | 0.222353 |
| BP | GO:0043029~T cell homeostasis | 3 | 1.578947 | 0.053495 | TGFB1, CASP3, BCL2 | 0.219904 |
| MF | GO:0019825~oxygen binding | 3 | 1.578947 | 0.052803 | ALB, CYP1A1, CYP3A4 | 0.226014 |
| BP | GO:0007626~locomotory behavior | 4 | 2.105263 | 0.052699 | APP, PTEN, SLC6A3, SOD1 | 0.216983 |
| CC | GO:0035578~azurophil granule lumen | 4 | 2.105263 | 0.051848 | FABP5, MAPK1, LYZ, MPO | 0.219143 |
| BP | GO:0019369~arachidonic acid metabolic process | 3 | 1.578947 | 0.051 | ALOX5, CYP1B1, ALOX12 | 0.210325 |
| BP | GO:0010975~regulation of neuron projection development | 3 | 1.578947 | 0.051 | GSK3B, PTEN, AKT1 | 0.210325 |
| BP | GO:0045787~positive regulation of cell cycle | 3 | 1.578947 | 0.051 | IL10, CHEK1, SLC6A4 | 0.210325 |
| BP | GO:0043407~negative regulation of MAP kinase activity | 3 | 1.578947 | 0.051 | PTPN1, IL1B, PPARG | 0.210325 |
| BP | GO:0040018~positive regulation of multicellular organism growth | 3 | 1.578947 | 0.051 | BCL2, IGF2, SLC6A3 | 0.210325 |
| BP | GO:2001244~positive regulation of intrinsic apoptotic signaling pathway | 3 | 1.578947 | 0.051 | CAV1, BAX, TP53 | 0.210325 |
| MF | GO:0004190~aspartic-type endopeptidase activity | 3 | 1.578947 | 0.050272 | CASP7, CASP3, CTSD | 0.216821 |
| CC | GO:0005819~spindle | 5 | 2.631579 | 0.050082 | RB1, HSPB1, BIRC5, AKT1, NR3C1 | 0.214541 |
| BP | GO:0045727~positive regulation of translation | 4 | 2.105263 | 0.049756 | IL6, MYC, ERBB2, CYP1B1 | 0.207197 |
| CC | GO:0010008~endosome membrane | 7 | 3.684211 | 0.04957 | INSR, ERBB2, CD14, ADRB2, CTSD, EGFR, SLC6A4 | 0.214541 |
| MF | GO:0031694~alpha-2A adrenergic receptor binding | 2 | 1.052632 | 0.049058 | ADRB1, ADRA2C | 0.213215 |
| MF | GO:0070891~lipoteichoic acid binding | 2 | 1.052632 | 0.049058 | CD14, LBP | 0.213215 |
| MF | GO:0048248~CXCR3 chemokine receptor binding | 2 | 1.052632 | 0.049058 | CXCL10, CXCL11 | 0.213215 |
| MF | GO:0004784~superoxide dismutase activity | 2 | 1.052632 | 0.049058 | NQO1, SOD1 | 0.213215 |
| BP | GO:0042113~B cell activation | 3 | 1.578947 | 0.048549 | IL4, CASP8, PRKCB | 0.2025 |
| BP | GO:0001890~placenta development | 3 | 1.578947 | 0.048549 | PPARG, MAPK14, SOD1 | 0.2025 |
| BP | GO:0090050~positive regulation of cell migration involved in sprouting angiogenesis | 3 | 1.578947 | 0.048549 | KDR, HMOX1, PTGS2 | 0.2025 |
| BP | GO:0071494~cellular response to UV-C | 2 | 1.052632 | 0.048127 | MDM2, TP53 | 0.201722 |
| BP | GO:0035509~negative regulation of myosin-light-chain-phosphatase activity | 2 | 1.052632 | 0.048127 | IKBKB, TNF | 0.201722 |
| BP | GO:0071373~cellular response to luteinizing hormone stimulus | 2 | 1.052632 | 0.048127 | CCNA2, CYP1B1 | 0.201722 |
| BP | GO:0031284~positive regulation of guanylate cyclase activity | 2 | 1.052632 | 0.048127 | NOS2, NOS3 | 0.201722 |
| BP | GO:0072584~caveolin-mediated endocytosis | 2 | 1.052632 | 0.048127 | CAV1, MAPK1 | 0.201722 |
| BP | GO:0002326~B cell lineage commitment | 2 | 1.052632 | 0.048127 | BCL2, TP53 | 0.201722 |
| BP | GO:0032811~negative regulation of epinephrine secretion | 2 | 1.052632 | 0.048127 | ADRA2C, ADRA2A | 0.201722 |
| BP | GO:2000535~regulation of entry of bacterium into host cell | 2 | 1.052632 | 0.048127 | CXCL8, CAV1 | 0.201722 |
| BP | GO:0038033~positive regulation of endothelial cell chemotaxis by VEGF-activated vascular endothelial growth factor receptor signaling pathway | 2 | 1.052632 | 0.048127 | KDR, HSPB1 | 0.201722 |
| BP | GO:0002071~glandular epithelial cell maturation | 2 | 1.052632 | 0.048127 | PGR, HIF1A | 0.201722 |
| BP | GO:0060355~positive regulation of cell adhesion molecule production | 2 | 1.052632 | 0.048127 | IL1B, CAV1 | 0.201722 |
| BP | GO:0071312~cellular response to alkaloid | 2 | 1.052632 | 0.048127 | MDM2, BCL2L1 | 0.201722 |
| BP | GO:0043243~positive regulation of protein complex disassembly | 2 | 1.052632 | 0.048127 | INSR, TNF | 0.201722 |
| BP | GO:2000481~positive regulation of cAMP-dependent protein kinase activity | 2 | 1.052632 | 0.048127 | ADRB2, SIRT1 | 0.201722 |
| BP | GO:0022614~membrane to membrane docking | 2 | 1.052632 | 0.048127 | VCAM1, ICAM1 | 0.201722 |
| BP | GO:0072734~cellular response to staurosporine | 2 | 1.052632 | 0.048127 | CASP7, CASP3 | 0.201722 |
| BP | GO:0070427~nucleotide-binding oligomerization domain containing 1 signaling pathway | 2 | 1.052632 | 0.048127 | NFKBIA, XIAP | 0.201722 |
| BP | GO:0032310~prostaglandin secretion | 2 | 1.052632 | 0.048127 | NOS2, PTGS2 | 0.201722 |
| BP | GO:0008544~epidermis development | 4 | 2.105263 | 0.046897 | PPP3CA, FABP5, INSR, PPARA | 0.201722 |
| MF | GO:0001618~virus receptor activity | 4 | 2.105263 | 0.04629 | DPP4, HTR2A, EGFR, ICAM1 | 0.20757 |
| BP | GO:0048678~response to axon injury | 3 | 1.578947 | 0.046143 | BAX, APOD, SOD1 | 0.199616 |
| BP | GO:0032481~positive regulation of type I interferon production | 3 | 1.578947 | 0.046143 | IRF1, XIAP, CD14 | 0.199616 |
| BP | GO:0043542~endothelial cell migration | 3 | 1.578947 | 0.046143 | DPP4, NOS3, CYP1B1 | 0.199616 |
| BP | GO:0002230~positive regulation of defense response to virus by host | 3 | 1.578947 | 0.046143 | IL4, HSP90AA1, STAT1 | 0.199616 |
| BP | GO:0070059~intrinsic apoptotic signaling pathway in response to endoplasmic reticulum stress | 3 | 1.578947 | 0.046143 | BCL2, BAX, TP53 | 0.199616 |
| BP | GO:0030324~lung development | 4 | 2.105263 | 0.045498 | COL3A1, NOS3, ELK1, EGFR | 0.198502 |
| CC | GO:0035976~transcription factor AP-1 complex | 2 | 1.052632 | 0.045214 | JUN, FOS | 0.199068 |
| MF | GO:0008289~lipid binding | 6 | 3.157895 | 0.044836 | FABP5, PTEN, BAX, APOD, PPARA, PPARD | 0.202661 |
| BP | GO:0021987~cerebral cortex development | 4 | 2.105263 | 0.044121 | NCOA1, COL3A1, BAX, HIF1A | 0.192822 |
| BP | GO:0002027~regulation of heart rate | 3 | 1.578947 | 0.043783 | MDM2, CALM3, SCN5A | 0.191671 |
| BP | GO:0001662~behavioral fear response | 3 | 1.578947 | 0.043783 | DPP4, BCL2, DRD1 | 0.191671 |
| BP | GO:0051276~chromosome organization | 3 | 1.578947 | 0.043783 | RB1, MYC, TP53 | 0.191671 |
| BP | GO:0045648~positive regulation of erythrocyte differentiation | 3 | 1.578947 | 0.043783 | STAT1, MAPK14, HIF1A | 0.191671 |
| BP | GO:0005977~glycogen metabolic process | 3 | 1.578947 | 0.043783 | GSK3B, AKT1, PYGM | 0.191671 |
| BP | GO:0032720~negative regulation of tumor necrosis factor production | 4 | 2.105263 | 0.042765 | IL10, IL4, CHRNA7, LBP | 0.188829 |
| BP | GO:0048469~cell maturation | 3 | 1.578947 | 0.041469 | CTNNB1, PPARG, RUNX2 | 0.183424 |
| BP | GO:0051973~positive regulation of telomerase activity | 3 | 1.578947 | 0.041469 | HSP90AA1, MYC, MAPK1 | 0.183424 |
| BP | GO:0048873~homeostasis of number of cells within a tissue | 3 | 1.578947 | 0.041469 | NOS3, BCL2, BAX | 0.183424 |
| BP | GO:0071902~positive regulation of protein serine/threonine kinase activity | 3 | 1.578947 | 0.041469 | IFNG, CALM3, ADRB2 | 0.183424 |
| BP | GO:0008210~estrogen metabolic process | 3 | 1.578947 | 0.041469 | CYP1A1, CYP1B1, CYP3A4 | 0.183424 |
| BP | GO:0035633~maintenance of permeability of blood-brain barrier | 3 | 1.578947 | 0.041469 | IL6, GJA1, PTGS2 | 0.183424 |
| BP | GO:0071310~cellular response to organic substance | 3 | 1.578947 | 0.041469 | STAT1, IL1B, BCL2 | 0.183424 |
| BP | GO:0032729~positive regulation of interferon-gamma production | 4 | 2.105263 | 0.04143 | IL1B, CD14, TNF, IL2 | 0.183424 |
| BP | GO:0045669~positive regulation of osteoblast differentiation | 4 | 2.105263 | 0.04143 | PPP3CA, IL6, CTNNB1, RUNX2 | 0.183424 |
| CC | GO:0016604~nuclear body | 8 | 4.210526 | 0.041109 | CDKN1A, PCNA, PARP1, NR1I2, MDM2, NFATC1, HIF1A, TP53 | 0.183545 |
| MF | GO:0071889~14-3-3 protein binding | 3 | 1.578947 | 0.040614 | AKT1, ESR1, TP53 | 0.185057 |
| BP | GO:0048511~rhythmic process | 4 | 2.105263 | 0.040117 | MAPK8, PPARG, AHR, TP53 | 0.180246 |
| BP | GO:0009267~cellular response to starvation | 4 | 2.105263 | 0.040117 | CAV1, ALB, PPARA, SIRT1 | 0.180246 |
| BP | GO:0043433~negative regulation of sequence-specific DNA binding transcription factor activity | 4 | 2.105263 | 0.040117 | RB1, EGLN1, ESR1, SIRT1 | 0.180246 |
| BP | GO:0031398~positive regulation of protein ubiquitination | 4 | 2.105263 | 0.040117 | GSK3B, HSPA5, CAV1, XIAP | 0.180246 |
| MF | GO:0004052~arachidonate 12-lipoxygenase activity | 2 | 1.052632 | 0.039442 | ALOX5, ALOX12 | 0.181177 |
| MF | GO:0051380~norepinephrine binding | 2 | 1.052632 | 0.039442 | ADRB2, ADRA2A | 0.181177 |
| MF | GO:0035033~histone deacetylase regulator activity | 2 | 1.052632 | 0.039442 | MAPK8, TP53 | 0.181177 |
| MF | GO:0050692~DBD domain binding | 2 | 1.052632 | 0.039442 | RXRA, PPARG | 0.181177 |
| MF | GO:0034617~tetrahydrobiopterin binding | 2 | 1.052632 | 0.039442 | NOS2, NOS3 | 0.181177 |
| BP | GO:0043122~regulation of I-kappaB kinase/NF-kappaB signaling | 3 | 1.578947 | 0.039204 | IL1B, HSPB1, TNF | 0.177386 |
| BP | GO:0045191~regulation of isotype switching | 2 | 1.052632 | 0.038689 | IL10, IL4 | 0.175368 |
| BP | GO:0072717~cellular response to actinomycin D | 2 | 1.052632 | 0.038689 | MDM2, TP53 | 0.175368 |
| BP | GO:0065008~regulation of biological quality | 2 | 1.052632 | 0.038689 | MDM2, TNF | 0.175368 |
| BP | GO:0060742~epithelial cell differentiation involved in prostate gland development | 2 | 1.052632 | 0.038689 | AR, CTNNB1 | 0.175368 |
| BP | GO:1904783~positive regulation of NMDA glutamate receptor activity | 2 | 1.052632 | 0.038689 | IFNG, CCL2 | 0.175368 |
| BP | GO:0031649~heat generation | 2 | 1.052632 | 0.038689 | ADRB1, ADRB2 | 0.175368 |
| BP | GO:0046666~retinal cell programmed cell death | 2 | 1.052632 | 0.038689 | BCL2, BAX | 0.175368 |
| BP | GO:0072331~signal transduction by p53 class mediator | 2 | 1.052632 | 0.038689 | CDKN1A, TP53 | 0.175368 |
| BP | GO:1990911~response to psychosocial stress | 2 | 1.052632 | 0.038689 | HSF1, ADRB2 | 0.175368 |
| BP | GO:0035624~receptor transactivation | 2 | 1.052632 | 0.038689 | ADRA2C, ADRA2A | 0.175368 |
| BP | GO:0038020~insulin receptor recycling | 2 | 1.052632 | 0.038689 | PTPN1, CTSD | 0.175368 |
| BP | GO:0060571~morphogenesis of an epithelial fold | 2 | 1.052632 | 0.038689 | AR, EGFR | 0.175368 |
| BP | GO:0036005~response to macrophage colony-stimulating factor | 2 | 1.052632 | 0.038689 | SPP1, TNF | 0.175368 |
| BP | GO:0030730~sequestering of triglyceride | 2 | 1.052632 | 0.038689 | IL1B, TNF | 0.175368 |
| BP | GO:0033590~response to cobalamin | 2 | 1.052632 | 0.038689 | RELA, EGFR | 0.175368 |
| MF | GO:0005230~extracellular ligand-gated ion channel activity | 3 | 1.578947 | 0.038323 | GABRA1, CHRNA2, CHRNA7 | 0.181177 |
| MF | GO:0004497~monooxygenase activity | 4 | 2.105263 | 0.038123 | CYP2B6, CYP1A1, CYP1B1, CYP3A4 | 0.181177 |
| MF | GO:0043621~protein self-association | 4 | 2.105263 | 0.038123 | ACHE, HSF1, PPARG, TP53 | 0.181177 |
| BP | GO:0070372~regulation of ERK1 and ERK2 cascade | 3 | 1.578947 | 0.036987 | IL1B, ERBB2, EGFR | 0.172227 |
| BP | GO:0008015~blood circulation | 3 | 1.578947 | 0.036987 | CXCL10, STAT1, OLR1 | 0.172227 |
| BP | GO:0045930~negative regulation of mitotic cell cycle | 3 | 1.578947 | 0.036987 | IL10, TNF, EGFR | 0.172227 |
| BP | GO:0042220~response to cocaine | 3 | 1.578947 | 0.036987 | HSP90AA1, MDM2, SLC6A3 | 0.172227 |
| BP | GO:0042327~positive regulation of phosphorylation | 3 | 1.578947 | 0.036987 | AR, KDR, EGFR | 0.172227 |
| BP | GO:0001935~endothelial cell proliferation | 3 | 1.578947 | 0.036987 | CAV1, HMOX1, NOX5 | 0.172227 |
| BP | GO:1900745~positive regulation of p38MAPK cascade | 3 | 1.578947 | 0.036987 | NCF1, IL1B, XDH | 0.172227 |
| BP | GO:1900017~positive regulation of cytokine production involved in inflammatory response | 3 | 1.578947 | 0.036987 | IL6, HIF1A, TNF | 0.172227 |
| BP | GO:0030878~thyroid gland development | 3 | 1.578947 | 0.036987 | MAPK1, RAF1, DUOX2 | 0.172227 |
| MF | GO:0004175~endopeptidase activity | 4 | 2.105263 | 0.036841 | MMP1, MMP2, MMP3, MMP9 | 0.181001 |
| BP | GO:0030512~negative regulation of transforming growth factor beta receptor signaling pathway | 5 | 2.631579 | 0.036609 | HSPA5, PPARG, PPARA, SIRT1, TP53 | 0.172227 |
| CC | GO:0035189~Rb-E2F complex | 2 | 1.052632 | 0.036337 | RB1, E2F1 | 0.164555 |
| MF | GO:0008237~metallopeptidase activity | 4 | 2.105263 | 0.035581 | MMP2, MMP3, LTA4H, MMP9 | 0.176344 |
| BP | GO:1990000~amyloid fibril formation | 3 | 1.578947 | 0.034822 | APP, CDKN2A, MDM2 | 0.165145 |
| BP | GO:0051602~response to electrical stimulus | 3 | 1.578947 | 0.034822 | NQO1, MMP2, CD14 | 0.165145 |
| BP | GO:0009409~response to cold | 3 | 1.578947 | 0.032707 | HSP90AA1, ADRB1, ADRB2 | 0.155695 |
| BP | GO:0045742~positive regulation of epidermal growth factor receptor signaling pathway | 3 | 1.578947 | 0.032707 | TGFB1, MMP9, ADRA2A | 0.155695 |
| BP | GO:0042104~positive regulation of activated T cell proliferation | 3 | 1.578947 | 0.032707 | PPP3CA, IGF2, IL2 | 0.155695 |
| BP | GO:0001678~cellular glucose homeostasis | 3 | 1.578947 | 0.032707 | HIF1A, SIRT1, HK2 | 0.155695 |
| BP | GO:0035458~cellular response to interferon-beta | 3 | 1.578947 | 0.032707 | STAT1, IRF1, CALM3 | 0.155695 |
| BP | GO:0032922~circadian regulation of gene expression | 4 | 2.105263 | 0.032695 | AHR, TOP1, PPARA, SIRT1 | 0.155695 |
| MF | GO:0004364~glutathione transferase activity | 3 | 1.578947 | 0.031764 | GSTM1, GSTA2, PTGES | 0.158822 |
| BP | GO:0071300~cellular response to retinoic acid | 4 | 2.105263 | 0.031535 | GSK3B, PPARG, TNF, SLC6A4 | 0.151806 |
| BP | GO:0006260~DNA replication | 5 | 2.631579 | 0.031465 | PCNA, CHEK1, CDK2, TOP1, ADRA2A | 0.151755 |
| CC | GO:0005657~replication fork | 3 | 1.578947 | 0.031 | PCNA, CHEK1, TP53 | 0.142421 |
| CC | GO:0030666~endocytic vesicle membrane | 4 | 2.105263 | 0.03094 | GRIA2, NOS3, CAV1, MDM2 | 0.142421 |
| BP | GO:0010800~positive regulation of peptidyl-threonine phosphorylation | 3 | 1.578947 | 0.030646 | APP, MAPK1, CALM3 | 0.148084 |
| BP | GO:0033137~negative regulation of peptidyl-serine phosphorylation | 3 | 1.578947 | 0.030646 | CAV1, PTEN, BAX | 0.148084 |
| BP | GO:0045776~negative regulation of blood pressure | 3 | 1.578947 | 0.030646 | NOS2, NOS3, PPARA | 0.148084 |
| BP | GO:0006898~receptor-mediated endocytosis | 4 | 2.105263 | 0.030397 | INSR, CD14, ADRB2, EGFR | 0.147715 |
| BP | GO:0031175~neuron projection development | 5 | 2.631579 | 0.029851 | RB1, GSK3B, APP, IL6, MAP2 | 0.14534 |
| MF | GO:0008384~IkappaB kinase activity | 2 | 1.052632 | 0.029729 | IKBKB, CHUK | 0.149972 |
| MF | GO:0005334~norepinephrine:sodium symporter activity | 2 | 1.052632 | 0.029729 | SLC6A2, SLC6A3 | 0.149972 |
| MF | GO:0005330~dopamine:sodium symporter activity | 2 | 1.052632 | 0.029729 | SLC6A2, SLC6A3 | 0.149972 |
| MF | GO:0052596~phenethylamine:oxygen oxidoreductase (deaminating) activity | 2 | 1.052632 | 0.029729 | MAOB, MAOA | 0.149972 |
| MF | GO:0097621~monoamine oxidase activity | 2 | 1.052632 | 0.029729 | MAOB, MAOA | 0.149972 |
| MF | GO:0051425~PTB domain binding | 2 | 1.052632 | 0.029729 | APP, INSR | 0.149972 |
| MF | GO:0004938~alpha2-adrenergic receptor activity | 2 | 1.052632 | 0.029729 | ADRA2C, ADRA2A | 0.149972 |
| MF | GO:0004517~nitric-oxide synthase activity | 2 | 1.052632 | 0.029729 | NOS2, NOS3 | 0.149972 |
| MF | GO:0016712~oxidoreductase activity, acting on paired donors, with incorporation or reduction of molecular oxygen, reduced flavin or flavoprotein as one donor, and incorporation of one atom of oxygen | 3 | 1.578947 | 0.029688 | CYP2B6, CYP1B1, CYP3A4 | 0.149972 |
| BP | GO:1904999~positive regulation of leukocyte adhesion to arterial endothelial cell | 2 | 1.052632 | 0.029159 | ALOX5, TNF | 0.142239 |
| BP | GO:0010641~positive regulation of platelet-derived growth factor receptor signaling pathway | 2 | 1.052632 | 0.029159 | F7, F3 | 0.142239 |
| BP | GO:0031399~regulation of protein modification process | 2 | 1.052632 | 0.029159 | IKBKB, RAF1 | 0.142239 |
| BP | GO:0060769~positive regulation of epithelial cell proliferation involved in prostate gland development | 2 | 1.052632 | 0.029159 | AR, CTNNB1 | 0.142239 |
| BP | GO:0051097~negative regulation of helicase activity | 2 | 1.052632 | 0.029159 | SIRT1, TP53 | 0.142239 |
| BP | GO:1900100~positive regulation of plasma cell differentiation | 2 | 1.052632 | 0.029159 | IL10, IL2 | 0.142239 |
| BP | GO:2000111~positive regulation of macrophage apoptotic process | 2 | 1.052632 | 0.029159 | CDKN2A, SIRT1 | 0.142239 |
| BP | GO:0002025~norepinephrine-epinephrine-mediated vasodilation involved in regulation of systemic arterial blood pressure | 2 | 1.052632 | 0.029159 | ADRB1, ADRB2 | 0.142239 |
| BP | GO:0002679~respiratory burst involved in defense response | 2 | 1.052632 | 0.029159 | MPO, PIK3CG | 0.142239 |
| BP | GO:1902948~negative regulation of tau-protein kinase activity | 2 | 1.052632 | 0.029159 | RB1, IFNG | 0.142239 |
| BP | GO:0001661~conditioned taste aversion | 2 | 1.052632 | 0.029159 | FOS, DRD1 | 0.142239 |
| BP | GO:0001660~fever generation | 2 | 1.052632 | 0.029159 | IL1A, IL1B | 0.142239 |
| BP | GO:1903131~mononuclear cell differentiation | 2 | 1.052632 | 0.029159 | NFATC1, FOS | 0.142239 |
| BP | GO:0060748~tertiary branching involved in mammary gland duct morphogenesis | 2 | 1.052632 | 0.029159 | AR, PGR | 0.142239 |
| BP | GO:0045986~negative regulation of smooth muscle contraction | 2 | 1.052632 | 0.029159 | ADRB2, PTGS2 | 0.142239 |
| BP | GO:0002904~positive regulation of B cell apoptotic process | 2 | 1.052632 | 0.029159 | IL10, BAX | 0.142239 |
| BP | GO:0031281~positive regulation of cyclase activity | 2 | 1.052632 | 0.029159 | MAPK8, MAPK14 | 0.142239 |
| BP | GO:0048597~post-embryonic camera-type eye morphogenesis | 2 | 1.052632 | 0.029159 | KDR, BAX | 0.142239 |
| BP | GO:0001996~positive regulation of heart rate by epinephrine-norepinephrine | 2 | 1.052632 | 0.029159 | ADRB1, ADRA1B | 0.142239 |
| CC | GO:0032809~neuronal cell body membrane | 3 | 1.578947 | 0.029107 | INSR, SLC6A2, SLC6A3 | 0.137715 |
| BP | GO:0051607~defense response to virus | 7 | 3.684211 | 0.028714 | IL6, IFNG, STAT1, IRF1, BCL2, RELA, BCL2L1 | 0.142239 |
| BP | GO:0007190~activation of adenylate cyclase activity | 3 | 1.578947 | 0.028639 | DRD1, ADRB2, RAF1 | 0.142239 |
| BP | GO:0072332~intrinsic apoptotic signaling pathway by p53 class mediator | 3 | 1.578947 | 0.028639 | E2F1, BAX, TP53 | 0.142239 |
| BP | GO:0042026~protein refolding | 3 | 1.578947 | 0.028639 | HSP90AA1, HSPA5, HSPB1 | 0.142239 |
| BP | GO:0002639~positive regulation of immunoglobulin production | 3 | 1.578947 | 0.028639 | IL10, IL6, IL2 | 0.142239 |
| BP | GO:0010033~response to organic substance | 3 | 1.578947 | 0.028639 | CDK2, RELA, SOD1 | 0.142239 |
| MF | GO:0030971~receptor tyrosine kinase binding | 4 | 2.105263 | 0.028502 | PTPN1, PCNA, ERBB2, TP53 | 0.149972 |
| MF | GO:1990841~promoter-specific chromatin binding | 4 | 2.105263 | 0.028502 | STAT1, HSF1, SIRT1, TP53 | 0.149972 |
| BP | GO:0042752~regulation of circadian rhythm | 4 | 2.105263 | 0.028187 | GSK3B, MAPK8, PPARG, PPARA | 0.142239 |
| BP | GO:0051898~negative regulation of protein kinase B signaling | 4 | 2.105263 | 0.028187 | PTEN, PPARA, SIRT1, XDH | 0.142239 |
| CC | GO:0043293~apoptosome | 2 | 1.052632 | 0.027378 | CASP9, CYCS | 0.131497 |
| CC | GO:0097124~cyclin A2-CDK2 complex | 2 | 1.052632 | 0.027378 | CCNA2, CDK2 | 0.131497 |
| CC | GO:0000228~nuclear chromosome | 3 | 1.578947 | 0.027262 | JUN, BIRC5, TOP1 | 0.131497 |
| BP | GO:0010595~positive regulation of endothelial cell migration | 4 | 2.105263 | 0.027116 | KDR, AKT1, PRKCA, PIK3CG | 0.139443 |
| MF | GO:0004713~protein tyrosine kinase activity | 5 | 2.631579 | 0.026933 | INSR, ERBB2, KDR, MET, EGFR | 0.149972 |
| BP | GO:0001764~neuron migration | 5 | 2.631579 | 0.026774 | COL3A1, HSP90AA1, GJA1, BAX, CTNNB1 | 0.137966 |
| BP | GO:2000353~positive regulation of endothelial cell apoptotic process | 3 | 1.578947 | 0.026687 | TGFB1, CD40LG, CCL2 | 0.137793 |
| BP | GO:0032740~positive regulation of interleukin-17 production | 3 | 1.578947 | 0.026687 | IL6, TGFB1, IL2 | 0.137793 |
| BP | GO:0001782~B cell homeostasis | 3 | 1.578947 | 0.026687 | CASP3, BCL2, BAX | 0.137793 |
| BP | GO:0007202~activation of phospholipase C activity | 3 | 1.578947 | 0.026687 | HTR2A, SELE, EGFR | 0.137793 |
| BP | GO:0071280~cellular response to copper ion | 3 | 1.578947 | 0.026687 | HSF1, CYP1A1, NFE2L2 | 0.137793 |
| BP | GO:0003009~skeletal muscle contraction | 3 | 1.578947 | 0.026687 | HSP90AA1, CHUK, TNF | 0.137793 |
| BP | GO:0007271~synaptic transmission, cholinergic | 3 | 1.578947 | 0.026687 | NQO1, CHRNA2, CHRNA7 | 0.137793 |
| CC | GO:0016020~membrane | 65 | 34.21053 | 0.026571 | MT-ND6, CHRM2, ACHE, APP, GSK3B, CHRM1, NCF1, HTR2A, SLC2A4, NR3C1, SLC6A2, SLC6A3, HK2, PIK3CG, SLC6A4, ICAM1, THBD, MYC, CYP1B1, AKT1, OLR1, SCN5A, LBP, KCNH2, HSP90AA1, PARP1, DIO1, PRKCA, OPRM1, F3, AR, KCNMA1, MET, TP53, PTGES, ABCG2, OPRD1, GRIA2, CHRNA2, MAOB, CHRNA7, PTGER3, ALOX12, ADRB2, CYP3A4, EGFR, DPP4, ERBB2, HMOX1, DUOX2, PTPN1, NQO1, GABRA1, MGAM, VCAM1, HSPA5, INSR, CAV1, ESR1, CD40LG, PDE3A, BCL2, BAX, CTNNB1, NOX5 | 0.131497 |
| CC | GO:0005769~early endosome | 8 | 4.210526 | 0.025874 | PTPN1, APP, VCAM1, ERBB2, KDR, MAPK1, ADRB1, ADRB2 | 0.131497 |
| CC | GO:0032590~dendrite membrane | 3 | 1.578947 | 0.025467 | OPRD1, GABRA1, INSR | 0.131497 |
| MF | GO:0016705~oxidoreductase activity, acting on paired donors, with incorporation or reduction of molecular oxygen | 4 | 2.105263 | 0.025274 | EGLN1, CYP2B6, CYP1B1, CYP3A4 | 0.1428 |
| CC | GO:0005768~endosome | 8 | 4.210526 | 0.025053 | APP, CAV1, CDK2, KDR, ADRB2, OPRM1, ADRA2C, EGFR | 0.131497 |
| BP | GO:0030325~adrenal gland development | 3 | 1.578947 | 0.024792 | INSR, CYP1B1, NR3C1 | 0.12984 |
| BP | GO:0001894~tissue homeostasis | 3 | 1.578947 | 0.024792 | RB1, COL3A1, ACACA | 0.12984 |
| BP | GO:0095500~acetylcholine receptor signaling pathway | 3 | 1.578947 | 0.024792 | ACHE, CHRNA2, CHRNA7 | 0.12984 |
| BP | GO:0042742~defense response to bacterium | 6 | 3.157895 | 0.024638 | IL10, CASP7, NOS2, XIAP, LYZ, MPO | 0.12983 |
| BP | GO:0042552~myelination | 4 | 2.105263 | 0.024035 | TGFB1, ERBB2, MAPK1, RAF1 | 0.126915 |
| BP | GO:0007169~transmembrane receptor protein tyrosine kinase signaling pathway | 5 | 2.631579 | 0.023896 | INSR, ERBB2, KDR, MET, EGFR | 0.126444 |
| BP | GO:0006874~cellular calcium ion homeostasis | 5 | 2.631579 | 0.023208 | TGFB1, PRKCB, CHRNA7, CAV1, HTR2A | 0.123054 |
| MF | GO:0000977~RNA polymerase II regulatory region sequence-specific DNA binding | 9 | 4.736842 | 0.023143 | RB1, NCOA1, RXRA, STAT1, IRF1, NR1I2, NR3C1, HIF1A, RELA | 0.13208 |
| BP | GO:0050768~negative regulation of neurogenesis | 3 | 1.578947 | 0.022954 | IL6, IL1B, TNF | 0.121963 |
| BP | GO:0090200~positive regulation of release of cytochrome c from mitochondria | 3 | 1.578947 | 0.022954 | BAX, TP53, MMP9 | 0.121963 |
| BP | GO:0032094~response to food | 3 | 1.578947 | 0.022954 | CYP1A1, AKT1, MPO | 0.121963 |
| BP | GO:0045736~negative regulation of cyclin-dependent protein serine/threonine kinase activity | 3 | 1.578947 | 0.022954 | CDKN1A, CDKN2A, PTEN | 0.121963 |
| MF | GO:0009055~electron carrier activity | 4 | 2.105263 | 0.022256 | MAOB, NCF1, AKR1B1, CYCS | 0.128311 |
| MF | GO:0000987~core promoter proximal region sequence-specific DNA binding | 4 | 2.105263 | 0.022256 | E2F1, AHR, HIF1A, TP53 | 0.128311 |
| BP | GO:0045785~positive regulation of cell adhesion | 4 | 2.105263 | 0.022093 | PPP3CA, ERBB2, PRKCA, TNF | 0.118369 |
| MF | GO:0005507~copper ion binding | 4 | 2.105263 | 0.021296 | IL1A, ALB, TP53, SOD1 | 0.125336 |
| MF | GO:0070888~E-box binding | 4 | 2.105263 | 0.021296 | MYC, PPARG, AHR, HIF1A | 0.125336 |
| MF | GO:0016491~oxidoreductase activity | 7 | 3.684211 | 0.020922 | MAOA, NOS3, CYP1A1, AKR1B1, CYP3A4, XDH, DUOX2 | 0.125336 |
| BP | GO:0007155~cell adhesion | 12 | 6.315789 | 0.020431 | DPP4, ACHE, APP, VCAM1, SPP1, CYP1B1, CCL2, CTNNB1, PRKCA, SELE, IL2, ICAM1 | 0.109695 |
| BP | GO:0050853~B cell receptor signaling pathway | 4 | 2.105263 | 0.020241 | NFKBIA, PRKCB, BCL2, MAPK1 | 0.1089 |
| MF | GO:0004993~G-protein coupled serotonin receptor activity | 3 | 1.578947 | 0.020184 | CHRM2, CHRM1, HTR2A | 0.122624 |
| MF | GO:0035403~histone kinase activity (H3-T6 specific) | 2 | 1.052632 | 0.019918 | PRKCB, PRKCA | 0.122325 |
| MF | GO:0052595~aliphatic-amine oxidase activity | 2 | 1.052632 | 0.019918 | MAOB, MAOA | 0.122325 |
| MF | GO:0070576~vitamin D 24-hydroxylase activity | 2 | 1.052632 | 0.019918 | CYP1A1, CYP3A4 | 0.122325 |
| MF | GO:0004666~prostaglandin-endoperoxide synthase activity | 2 | 1.052632 | 0.019918 | PTGS2, PTGS1 | 0.122325 |
| BP | GO:0097048~dendritic cell apoptotic process | 2 | 1.052632 | 0.019534 | BCL2, BCL2L1 | 0.105321 |
| BP | GO:1990268~response to gold nanoparticle | 2 | 1.052632 | 0.019534 | MPO, TNF | 0.105321 |
| BP | GO:0032227~negative regulation of synaptic transmission, dopaminergic | 2 | 1.052632 | 0.019534 | PTGS2, SLC6A4 | 0.105321 |
| BP | GO:2000098~negative regulation of smooth muscle cell-matrix adhesion | 2 | 1.052632 | 0.019534 | SERPINE1, APOD | 0.105321 |
| BP | GO:0014806~smooth muscle hyperplasia | 2 | 1.052632 | 0.019534 | NOS3, HMOX1 | 0.105321 |
| BP | GO:0006808~regulation of nitrogen utilization | 2 | 1.052632 | 0.019534 | BCL2, BAX | 0.105321 |
| BP | GO:0002439~chronic inflammatory response to antigenic stimulus | 2 | 1.052632 | 0.019534 | IL10, TNF | 0.105321 |
| BP | GO:0003057~regulation of the force of heart contraction by chemical signal | 2 | 1.052632 | 0.019534 | NOS3, CAV1 | 0.105321 |
| BP | GO:0002069~columnar/cuboidal epithelial cell maturation | 2 | 1.052632 | 0.019534 | GJA1, TGFB1 | 0.105321 |
| BP | GO:0003340~negative regulation of mesenchymal to epithelial transition involved in metanephros morphogenesis | 2 | 1.052632 | 0.019534 | STAT1, CTNNB1 | 0.105321 |
| BP | GO:1904681~response to 3-methylcholanthrene | 2 | 1.052632 | 0.019534 | CYP1A1, CYP1B1 | 0.105321 |
| BP | GO:0060557~positive regulation of vitamin D biosynthetic process | 2 | 1.052632 | 0.019534 | IFNG, TNF | 0.105321 |
| BP | GO:0034465~response to carbon monoxide | 2 | 1.052632 | 0.019534 | IL10, KCNMA1 | 0.105321 |
| BP | GO:0071887~leukocyte apoptotic process | 2 | 1.052632 | 0.019534 | CASP9, CASP3 | 0.105321 |
| BP | GO:0030520~intracellular estrogen receptor signaling pathway | 3 | 1.578947 | 0.019459 | AR, ESR1, ESR2 | 0.105321 |
| BP | GO:0006693~prostaglandin metabolic process | 3 | 1.578947 | 0.019459 | GSTM1, AKR1B1, PTGES | 0.105321 |
| BP | GO:0032148~activation of protein kinase B activity | 3 | 1.578947 | 0.019459 | INSR, ADRA2C, ADRA2A | 0.105321 |
| BP | GO:0060307~regulation of ventricular cardiac muscle cell membrane repolarization | 3 | 1.578947 | 0.019459 | KCNH2, GJA1, SCN5A | 0.105321 |
| BP | GO:0030282~bone mineralization | 4 | 2.105263 | 0.019348 | PTGS2, HIF1A, DUOX2, RUNX2 | 0.105321 |
| BP | GO:0060070~canonical Wnt signaling pathway | 5 | 2.631579 | 0.019331 | GSK3B, TGFB1, CAV1, PTEN, CTNNB1 | 0.105321 |
| MF | GO:0001227~transcriptional repressor activity, RNA polymerase II transcription regulatory region sequence-specific binding | 9 | 4.736842 | 0.019213 | JUN, MYC, HSF1, PPARG, PPARA, NR3C1, TP53, RELA, PPARD | 0.122325 |
| CC | GO:0009898~cytoplasmic side of plasma membrane | 5 | 2.631579 | 0.018896 | IKBKB, PPP3CA, NCF1, CHUK, PTEN | 0.101528 |
| CC | GO:0031904~endosome lumen | 3 | 1.578947 | 0.018802 | PTPN1, APP, CTSD | 0.101528 |
| BP | GO:0046718~viral entry into host cell | 5 | 2.631579 | 0.018728 | DPP4, INSR, HTR2A, EGFR, ICAM1 | 0.105321 |
| BP | GO:0071346~cellular response to interferon-gamma | 5 | 2.631579 | 0.018728 | NOS2, STAT1, CCL2, CALM3, TNF | 0.105321 |
| BP | GO:1903078~positive regulation of protein localization to plasma membrane | 4 | 2.105263 | 0.018478 | IFNG, AKT1, TNF, EGFR | 0.104463 |
| MF | GO:0000979~RNA polymerase II core promoter sequence-specific DNA binding | 3 | 1.578947 | 0.018469 | STAT1, FOS, RELA | 0.119944 |
| CC | GO:1905286~serine-type peptidase complex | 2 | 1.052632 | 0.018336 | F7, F3 | 0.101528 |
| CC | GO:0070557~PCNA-p21 complex | 2 | 1.052632 | 0.018336 | CDKN1A, PCNA | 0.101528 |
| BP | GO:0006338~chromatin remodeling | 9 | 4.736842 | 0.017828 | RB1, NCOA1, PRKCB, MYC, CHEK1, CDK2, PRKCA, TOP1, ESR1 | 0.10101 |
| BP | GO:0046677~response to antibiotic | 3 | 1.578947 | 0.017804 | HSP90AA1, MDM2, TP53 | 0.10101 |
| BP | GO:0014911~positive regulation of smooth muscle cell migration | 3 | 1.578947 | 0.017804 | BCL2, CYP1B1, HAS2 | 0.10101 |
| BP | GO:0006469~negative regulation of protein kinase activity | 4 | 2.105263 | 0.01763 | RB1, CDKN2A, HSPB1, AKT1 | 0.100557 |
| BP | GO:0045600~positive regulation of fat cell differentiation | 4 | 2.105263 | 0.01763 | AKT1, PPARG, HTR2A, PPARD | 0.100557 |
| CC | GO:0005796~Golgi lumen | 5 | 2.631579 | 0.017251 | F7, APP, TGFB1, F10, F2 | 0.099428 |
| MF | GO:0001216~bacterial-type RNA polymerase transcriptional activator activity, sequence-specific DNA binding | 3 | 1.578947 | 0.01682 | E2F1, PPARA, HIF1A | 0.110502 |
| BP | GO:0010718~positive regulation of epithelial to mesenchymal transition | 4 | 2.105263 | 0.016804 | IL6, TGFB1, IL1B, CTNNB1 | 0.096277 |
| BP | GO:0050680~negative regulation of epithelial cell proliferation | 4 | 2.105263 | 0.016804 | RB1, AR, TGFB1, PPARD | 0.096277 |
| BP | GO:0034220~ion transmembrane transport | 6 | 3.157895 | 0.016741 | KCNH2, GRIA2, GJA1, CHRNA2, CHRNA7, SCN5A | 0.096277 |
| BP | GO:0050901~leukocyte tethering or rolling | 3 | 1.578947 | 0.016211 | VCAM1, SELE, TNF | 0.093508 |
| BP | GO:0046827~positive regulation of protein export from nucleus | 3 | 1.578947 | 0.016211 | GSK3B, IL1B, MDM2 | 0.093508 |
| BP | GO:0042759~long-chain fatty acid biosynthetic process | 3 | 1.578947 | 0.016211 | ALOX5, CYP1A1, CYP3A4 | 0.093508 |
| BP | GO:0001516~prostaglandin biosynthetic process | 3 | 1.578947 | 0.016211 | PTGS2, PTGES, PTGS1 | 0.093508 |
| BP | GO:0006801~superoxide metabolic process | 3 | 1.578947 | 0.016211 | NCF1, NOS2, SOD1 | 0.093508 |
| BP | GO:0019373~epoxygenase P450 pathway | 3 | 1.578947 | 0.016211 | CYP2B6, CYP1A1, CYP1B1 | 0.093508 |
| BP | GO:0001892~embryonic placenta development | 3 | 1.578947 | 0.016211 | IGF2, HIF1A, EGFR | 0.093508 |
| BP | GO:0019229~regulation of vasoconstriction | 3 | 1.578947 | 0.016211 | ADRA1B, ADRA2C, ADRA2A | 0.093508 |
| BP | GO:0045780~positive regulation of bone resorption | 3 | 1.578947 | 0.016211 | SPP1, PRKCA, EGFR | 0.093508 |
| MF | GO:0043539~protein serine/threonine kinase activator activity | 4 | 2.105263 | 0.016026 | TGFB1, CD40LG, IGF2, CALM3 | 0.106527 |
| BP | GO:2000045~regulation of G1/S transition of mitotic cell cycle | 4 | 2.105263 | 0.016001 | CDKN1A, CCND1, CDKN2A, E2F1 | 0.093508 |
| BP | GO:0001837~epithelial to mesenchymal transition | 4 | 2.105263 | 0.016001 | GSK3B, TGFB1, CTNNB1, HIF1A | 0.093508 |
| BP | GO:0042572~retinol metabolic process | 4 | 2.105263 | 0.016001 | CYP1A1, AKR1B1, CYP1B1, CYP3A4 | 0.093508 |
| BP | GO:0046777~protein autophosphorylation | 6 | 3.157895 | 0.015891 | GSK3B, CHEK2, INSR, KDR, AKT1, EGFR | 0.093508 |
| BP | GO:0034599~cellular response to oxidative stress | 5 | 2.631579 | 0.015884 | NQO1, MAPK8, PARP1, HIF1A, NFE2L2 | 0.093508 |
| CC | GO:0098992~neuronal dense core vesicle | 3 | 1.578947 | 0.015795 | OPRD1, ADRB1, ADRB2 | 0.09272 |
| MF | GO:0005326~neurotransmitter transporter activity | 3 | 1.578947 | 0.015237 | SLC6A2, SLC6A3, SLC6A4 | 0.102487 |
| CC | GO:1904724~tertiary granule lumen | 4 | 2.105263 | 0.014909 | LTA4H, LYZ, CTSD, MMP9 | 0.089175 |
| BP | GO:0048514~blood vessel morphogenesis | 3 | 1.578947 | 0.014684 | GJA1, RASA1, CYP1B1 | 0.087456 |
| BP | GO:2000811~negative regulation of anoikis | 3 | 1.578947 | 0.014684 | CAV1, BCL2, BCL2L1 | 0.087456 |
| BP | GO:0042310~vasoconstriction | 3 | 1.578947 | 0.014684 | CRP, CAV1, SLC6A4 | 0.087456 |
| BP | GO:0050995~negative regulation of lipid catabolic process | 3 | 1.578947 | 0.014684 | IL1B, TNF, ADRA2A | 0.087456 |
| BP | GO:0030949~positive regulation of vascular endothelial growth factor receptor signaling pathway | 3 | 1.578947 | 0.014684 | PRKCB, IL1B, HIF1A | 0.087456 |
| BP | GO:0009416~response to light stimulus | 3 | 1.578947 | 0.014684 | MAPK8, FOS, ELK1 | 0.087456 |
| BP | GO:0031623~receptor internalization | 4 | 2.105263 | 0.01446 | ACHE, CXCL8, CAV1, INSR | 0.087343 |
| BP | GO:1900087~positive regulation of G1/S transition of mitotic cell cycle | 4 | 2.105263 | 0.01446 | CCND1, CYP1A1, AKT1, EGFR | 0.087343 |
| BP | GO:0010467~gene expression | 6 | 3.157895 | 0.013516 | TGFB1, AKT1, NR3C1, ELK1, RUNX2, SOD1 | 0.082028 |
| BP | GO:0051000~positive regulation of nitric-oxide synthase activity | 3 | 1.578947 | 0.013223 | AKT1, HIF1A, ESR1 | 0.080437 |
| BP | GO:0045019~negative regulation of nitric oxide biosynthetic process | 3 | 1.578947 | 0.013223 | IL10, CAV1, OPRM1 | 0.080437 |
| BP | GO:0030857~negative regulation of epithelial cell differentiation | 3 | 1.578947 | 0.013223 | IFNG, CCND1, CAV1 | 0.080437 |
| BP | GO:0060749~mammary gland alveolus development | 3 | 1.578947 | 0.013223 | AR, CCND1, ESR1 | 0.080437 |
| BP | GO:0071380~cellular response to prostaglandin E stimulus | 3 | 1.578947 | 0.013223 | AKT1, PPARG, ACACA | 0.080437 |
| BP | GO:0002028~regulation of sodium ion transport | 3 | 1.578947 | 0.013223 | TGFB1, NOS3, ADRB2 | 0.080437 |
| BP | GO:0030889~negative regulation of B cell proliferation | 3 | 1.578947 | 0.013223 | IL10, CDKN2A, CASP3 | 0.080437 |
| BP | GO:0018108~peptidyl-tyrosine phosphorylation | 4 | 2.105263 | 0.013008 | INSR, ERBB2, KDR, EGFR | 0.080437 |
| MF | GO:0004888~transmembrane signaling receptor activity | 7 | 3.684211 | 0.01295 | THBD, CHRNA2, CHRNA7, ERBB2, SELE, EGFR, ICAM1 | 0.088154 |
| CC | GO:0098685~Schaffer collateral - CA1 synapse | 5 | 2.631579 | 0.012864 | PPP3CA, CHRM1, CTNNB1, ADRB1, PLAT | 0.078422 |
| BP | GO:0051781~positive regulation of cell division | 4 | 2.105263 | 0.012315 | IL1A, TGFB1, IL1B, IGF2 | 0.076366 |
| BP | GO:0055093~response to hyperoxia | 3 | 1.578947 | 0.011829 | CDKN1A, MMP2, CYP1A1 | 0.073532 |
| BP | GO:1904385~cellular response to angiotensin | 3 | 1.578947 | 0.011829 | HSF1, RELA, NFE2L2 | 0.073532 |
| BP | GO:0042326~negative regulation of phosphorylation | 3 | 1.578947 | 0.011829 | CDKN1A, CDKN2A, SIRT1 | 0.073532 |
| BP | GO:0034620~cellular response to unfolded protein | 3 | 1.578947 | 0.011829 | PTPN1, HSF1, BAX | 0.073532 |
| CC | GO:0043020~NADPH oxidase complex | 3 | 1.578947 | 0.011717 | NCF1, NOX5, DUOX2 | 0.07283 |
| BP | GO:0000079~regulation of cyclin-dependent protein serine/threonine kinase activity | 4 | 2.105263 | 0.011644 | CCNA2, CDKN1A, CCND1, CDKN2A | 0.073088 |
| BP | GO:0030968~endoplasmic reticulum unfolded protein response | 4 | 2.105263 | 0.011644 | PTPN1, CCND1, HSPA5, NFE2L2 | 0.073088 |
| BP | GO:0032880~regulation of protein localization | 5 | 2.631579 | 0.011487 | HSP90AA1, MAPK8, PARP1, MAP2, BCL2 | 0.072459 |
| MF | GO:0001540~beta-amyloid binding | 5 | 2.631579 | 0.011355 | ACHE, GRIA2, CHRNA7, INSR, ADRB2 | 0.078236 |
| MF | GO:0005524~ATP binding | 26 | 13.68421 | 0.011112 | GSK3B, HK2, ACACA, EGFR, PIK3CG, IKBKB, MAPK8, CHEK2, ERBB2, CHEK1, KDR, AKT1, MAPK1, HSP90AA1, HSPA5, CHUK, PRKCB, INSR, PRKCA, MAPK14, RUNX2, CDK2, TOP1, RAF1, MET, ABCG2 | 0.077512 |
| BP | GO:0031647~regulation of protein stability | 5 | 2.631579 | 0.011054 | CDKN2A, CASP3, PTEN, BCL2, MAPK1 | 0.069896 |
| MF | GO:0004707~MAP kinase activity | 3 | 1.578947 | 0.010905 | MAPK8, MAPK1, MAPK14 | 0.077014 |
| MF | GO:0001046~core promoter sequence-specific DNA binding | 3 | 1.578947 | 0.010905 | MYC, NR3C1, TP53 | 0.077014 |
| BP | GO:0016485~protein processing | 5 | 2.631579 | 0.010631 | CASP9, F7, CASP7, CASP3, F3 | 0.067389 |
| BP | GO:0006816~calcium ion transport | 5 | 2.631579 | 0.010631 | PPP3CA, PRKCB, NOS3, CHRNA7, CAV1 | 0.067389 |
| BP | GO:0050731~positive regulation of peptidyl-tyrosine phosphorylation | 5 | 2.631579 | 0.010631 | IL6, TGFB1, IGF2, HTR2A, TP53 | 0.067389 |
| BP | GO:0046323~glucose import | 3 | 1.578947 | 0.010505 | DRD1, SLC2A4, MAPK14 | 0.067091 |
| BP | GO:0098664~G-protein coupled serotonin receptor signaling pathway | 3 | 1.578947 | 0.010505 | CHRM2, CHRM1, HTR2A | 0.067091 |
| BP | GO:0042176~regulation of protein catabolic process | 3 | 1.578947 | 0.010505 | CHEK2, ODC1, MDM2 | 0.067091 |
| BP | GO:0030890~positive regulation of B cell proliferation | 4 | 2.105263 | 0.010366 | IL4, CDKN1A, BCL2, IL2 | 0.066701 |
| BP | GO:0022617~extracellular matrix disassembly | 4 | 2.105263 | 0.010366 | MMP1, MMP2, MMP3, MMP9 | 0.066701 |
| BP | GO:0050829~defense response to Gram-negative bacterium | 5 | 2.631579 | 0.010219 | IL6, NOS2, SERPINE1, LBP, LYZ | 0.066085 |
| BP | GO:0008203~cholesterol metabolic process | 5 | 2.631579 | 0.010219 | IL4, APP, PON1, CYP3A4, PPARD | 0.066085 |
| BP | GO:0042177~negative regulation of protein catabolic process | 4 | 2.105263 | 0.009759 | NQO1, NOS2, RELA, EGFR | 0.063435 |
| BP | GO:0007189~adenylate cyclase-activating G-protein coupled receptor signaling pathway | 6 | 3.157895 | 0.009509 | CXCL10, CXCL11, PTGER3, DRD1, ADRB2, ADRA2A | 0.061969 |
| BP | GO:0001503~ossification | 5 | 2.631579 | 0.009426 | BCL2, SPP1, IGF2, EGFR, RUNX2 | 0.061579 |
| MF | GO:0008201~heparin binding | 7 | 3.684211 | 0.009357 | CXCL10, APP, CXCL11, CXCL8, PCOLCE, F2, MPO | 0.067779 |
| BP | GO:0045725~positive regulation of glycogen biosynthetic process | 3 | 1.578947 | 0.009251 | INSR, IGF2, AKT1 | 0.060594 |
| BP | GO:0014044~Schwann cell development | 3 | 1.578947 | 0.009251 | ERBB2, MAPK1, RAF1 | 0.060594 |
| BP | GO:0019395~fatty acid oxidation | 3 | 1.578947 | 0.009251 | PPARG, ALOX12, MAPK14 | 0.060594 |
| BP | GO:1900034~regulation of cellular response to heat | 3 | 1.578947 | 0.009251 | GSK3B, HSF1, SIRT1 | 0.060594 |
| BP | GO:0051412~response to corticosterone | 3 | 1.578947 | 0.009251 | CDKN1A, MAOB, FOS | 0.060594 |
| BP | GO:0030278~regulation of ossification | 3 | 1.578947 | 0.009251 | MAPK1, MAPK14, RUNX2 | 0.060594 |
| BP | GO:0071732~cellular response to nitric oxide | 3 | 1.578947 | 0.009251 | CCNA2, CDK2, MMP3 | 0.060594 |
| BP | GO:0048546~digestive tract morphogenesis | 3 | 1.578947 | 0.009251 | BCL2, HIF1A, EGFR | 0.060594 |
| BP | GO:0071364~cellular response to epidermal growth factor stimulus | 4 | 2.105263 | 0.009174 | ERBB2, AKT1, FOS, EGFR | 0.060594 |
| CC | GO:0098794~postsynapse | 6 | 3.157895 | 0.009097 | GSK3B, PPP3CA, GABRA1, GRIA2, CHRNA7, AKT1 | 0.057676 |
| BP | GO:0016525~negative regulation of angiogenesis | 6 | 3.157895 | 0.008933 | CXCL10, STAT1, ALOX5, CTNNB1, PPARG, RELA | 0.059887 |
| BP | GO:0061844~antimicrobial humoral immune response mediated by antimicrobial peptide | 5 | 2.631579 | 0.008674 | CXCL10, CXCL11, CXCL8, F2, CXCL2 | 0.058304 |
| BP | GO:0006952~defense response | 5 | 2.631579 | 0.008674 | STAT1, HSF1, NOX5, MPO, DUOX2 | 0.058304 |
| BP | GO:0050830~defense response to Gram-positive bacterium | 6 | 3.157895 | 0.008654 | CRP, IL6, IL1B, LBP, LYZ, TNF | 0.058304 |
| BP | GO:1904706~negative regulation of vascular smooth muscle cell proliferation | 4 | 2.105263 | 0.00861 | IL10, CDKN1A, PTEN, PPARG | 0.058304 |
| BP | GO:0031295~T cell costimulation | 4 | 2.105263 | 0.00861 | DPP4, CD40LG, CAV1, AKT1 | 0.058304 |
| BP | GO:0032088~negative regulation of NF-kappaB transcription factor activity | 5 | 2.631579 | 0.008313 | NFKBIA, CHUK, CDKN2A, CYP1B1, SIRT1 | 0.056622 |
| BP | GO:0002523~leukocyte migration involved in inflammatory response | 3 | 1.578947 | 0.008069 | ALOX5, SELE, TNF | 0.055109 |
| BP | GO:0006978~DNA damage response, signal transduction by p53 class mediator resulting in transcription of p21 class mediator | 3 | 1.578947 | 0.008069 | CDKN1A, CHEK2, TP53 | 0.055109 |
| BP | GO:0097284~hepatocyte apoptotic process | 3 | 1.578947 | 0.008069 | RB1, PIK3CG, BCL2L1 | 0.055109 |
| BP | GO:0031571~mitotic G1 DNA damage checkpoint | 3 | 1.578947 | 0.008069 | CCND1, CDK2, TP53 | 0.055109 |
| BP | GO:0034116~positive regulation of heterotypic cell-cell adhesion | 3 | 1.578947 | 0.008069 | IL10, IL1B, TNF | 0.055109 |
| BP | GO:0060134~prepulse inhibition | 3 | 1.578947 | 0.008069 | PTEN, DRD1, SLC6A3 | 0.055109 |
| BP | GO:1904996~positive regulation of leukocyte adhesion to vascular endothelial cell | 3 | 1.578947 | 0.008069 | IL6, TNF, RELA | 0.055109 |
| BP | GO:0045737~positive regulation of cyclin-dependent protein serine/threonine kinase activity | 3 | 1.578947 | 0.008069 | CCND1, AKT1, EGFR | 0.055109 |
| BP | GO:0021854~hypothalamus development | 3 | 1.578947 | 0.008069 | NCOA1, BAX, CTNNB1 | 0.055109 |
| BP | GO:0002281~macrophage activation involved in immune response | 3 | 1.578947 | 0.008069 | IFNG, LBP, TNF | 0.055109 |
| BP | GO:0009755~hormone-mediated signaling pathway | 4 | 2.105263 | 0.008066 | RXRA, PPARG, PPARA, PPARD | 0.055109 |
| BP | GO:0080090~regulation of primary metabolic process | 4 | 2.105263 | 0.008066 | CDKN1A, HSP90AA1, KDR, PRKCA | 0.055109 |
| BP | GO:0007565~female pregnancy | 5 | 2.631579 | 0.007962 | THBD, TGFB1, BCL2, FOS, ADRA2A | 0.055109 |
| BP | GO:0007275~multicellular organism development | 5 | 2.631579 | 0.007622 | INSR, ERBB2, KDR, MET, EGFR | 0.053919 |
| BP | GO:0072593~reactive oxygen species metabolic process | 4 | 2.105263 | 0.007544 | BCL2, NOX5, TP53, SOD1 | 0.053517 |
| BP | GO:0030574~collagen catabolic process | 4 | 2.105263 | 0.007544 | MMP1, MMP2, MMP3, MMP9 | 0.053517 |
| BP | GO:0045454~cell redox homeostasis | 4 | 2.105263 | 0.007544 | NQO1, NOS2, NOS3, NFE2L2 | 0.053517 |
| BP | GO:0006357~regulation of transcription from RNA polymerase II promoter | 27 | 14.21053 | 0.007433 | RB1, AHR, NR3C1, ELK1, HIF1A, RELA, NR3C2, MYC, HSF1, E2F1, HMOX1, MAPK1, JUN, PRKCB, STAT1, NFATC1, FOS, MAPK14, ESR1, RUNX2, ESR2, IRF1, PPARG, PGR, TP53, PPARD, NFE2L2 | 0.053173 |
| BP | GO:0007268~chemical synaptic transmission | 8 | 4.210526 | 0.007403 | CHRM2, GRIA2, CHRM1, CHRNA7, MAPK1, CTNNB1, HTR2A, SLC6A2 | 0.053107 |
| BP | GO:0008217~regulation of blood pressure | 5 | 2.631579 | 0.00729 | NOS3, PPARG, PTGS2, SOD1, PTGS1 | 0.052446 |
| BP | GO:0006953~acute-phase response | 4 | 2.105263 | 0.007042 | CRP, IL6, LBP, F2 | 0.050802 |
| BP | GO:0010389~regulation of G2/M transition of mitotic cell cycle | 3 | 1.578947 | 0.006961 | CDKN1A, CDKN2A, CDK2 | 0.050362 |
| BP | GO:0032495~response to muramyl dipeptide | 3 | 1.578947 | 0.006961 | NFKBIA, MAPK14, RELA | 0.050362 |
| BP | GO:1903377~negative regulation of oxidative stress-induced neuron intrinsic apoptotic signaling pathway | 3 | 1.578947 | 0.006961 | IL10, CTNNB1, HIF1A | 0.050362 |
| BP | GO:0010818~T cell chemotaxis | 3 | 1.578947 | 0.006961 | CXCL10, CXCL11, PIK3CG | 0.050362 |
| BP | GO:0046685~response to arsenic-containing substance | 3 | 1.578947 | 0.006961 | CDKN1A, CYP1A1, CYP1B1 | 0.050362 |
| MF | GO:0005515~protein binding | 177 | 93.15789 | 8.04E-18 | RB1, APP, CHRM1, NCF1, SERPINE1, PLAT, AHR, NR3C1, DCAF5, MPO, ELK1, TNF, ICAM1, NR3C2, GJA1, CCND1, PLAU, MYC, CHEK2, CHEK1, KDR, CYP1B1, AKT1, KCNH2, PRKCB, IGFBP3, PRKCA, PCOLCE, RUNX2, AR, SLPI, PGR, RAF1, TP53, PTGES, ABCG2, GRIA2, MAOB, MAOA, CHRNA7, ADRB1, ALOX12, ADRB2, HIF1A, ACACA, DPP4, MAP2, HMOX1, APOD, DRD1, CD14, LTA4H, MGAM, JUN, TGFB1, HSPA5, INSR, CAV1, IGF2, NFATC1, SELE, ESR1, ESR2, IL2, NFKBIA, IL4, CXCL10, CXCL11, IL6, ALB, CDK2, CYP1A1, PDE3A, BCL2, MDM2, CYCS, CALM3, BCL2L1, NFE2L2, ACHE, GSK3B, CDKN1A, CXCL8, PTEN, AKR1B1, HSPB1, PYGM, HTR2A, SLC2A4, ADRA1B, CXCL2, SLC6A2, HK2, SLC6A3, PIK3CG, SLC6A4, CASP9, IKBKB, THBD, PPP3CA, CASP7, CASP8, CA2, CASP3, OLR1, LBP, SCN5A, CTSD, IL10, NCOA1, HSP90AA1, CHUK, PARP1, MMP2, MMP3, AHSA1, FOS, OPRM1, F2, ADRA2C, F3, SIRT1, MMP9, ADRA2A, CCNA2, F7, IL1A, IFNG, RASA1, IRF1, IL1B, KCNMA1, BIRC5, PPARG, TOP1, PPARA, MET, PPARD, CRP, OPRD1, PCNA, ODC1, NR1I2, XIAP, CYP3A4, PTGS2, RELA, EGFR, PTGS1, MAPK8, RXRA, ALOX5, ERBB2, HSF1, SPP1, E2F1, CCL2, MAPK1, HAS2, XDH, DUOX2, EGLN1, PTPN1, NQO1, F10, NOS2, CDKN2A, STAT1, NOS3, MAPK14, SOD1, COL3A1, CD40LG, FABP5, GSTA2, BAX, CTNNB1 | 1.51E-15 |
| CC | GO:0005829~cytosol | 91 | 47.89474 | 9.52E-10 | RB1, APP, GSK3B, CDKN1A, NCF1, PTEN, AKR1B1, HSPB1, PYGM, AHR, SLC2A4, NR3C1, HK2, PIK3CG, NR3C2, CASP9, IKBKB, PPP3CA, GJA1, CASP7, CASP8, CCND1, CA2, CASP3, CHEK1, AKT1, NCOA1, HSP90AA1, PARP1, CHUK, PRKCB, AHSA1, PRKCA, FOS, SIRT1, RUNX2, CCNA2, IL1A, AR, RASA1, IRF1, IL1B, BIRC5, PPARG, PGR, RAF1, TP53, MAOA, ODC1, XIAP, ALOX12, HIF1A, ACACA, RELA, MAPK8, RXRA, MAP2, ALOX5, ERBB2, HSF1, HMOX1, MAPK1, LTA4H, XDH, DUOX2, EGLN1, PTPN1, NQO1, GSTM1, NOS2, HSPA5, CDKN2A, NOS3, STAT1, NFATC1, MAPK14, ESR1, SOD1, NFKBIA, FABP5, GSTA2, CDK2, PDE3A, BCL2, MDM2, CYCS, BAX, CTNNB1, CALM3, NFE2L2, BCL2L1 | 5.03E-08 |
| CC | GO:0005634~nucleus | 90 | 47.36842 | 1.76E-07 | RB1, ACHE, APP, GSK3B, CDKN1A, PTEN, HSPB1, AHR, ADRA1B, NR3C1, DCAF5, MPO, ELK1, NR3C2, CASP9, IKBKB, PPP3CA, GJA1, CASP7, CCND1, MYC, CHEK2, CASP3, CHEK1, KDR, AKT1, NCOA1, HSP90AA1, PARP1, CHUK, PRKCB, IGFBP3, MMP2, MMP3, PRKCA, FOS, SIRT1, RUNX2, CCNA2, IL1A, AR, IRF1, BIRC5, PPARG, PGR, TOP1, RAF1, PPARA, TP53, PPARD, PCNA, XIAP, ADRB2, HIF1A, EGFR, RELA, MAPK8, RXRA, ERBB2, HSF1, E2F1, HMOX1, MAPK1, DRD1, LTA4H, EGLN1, NQO1, JUN, TGFB1, NOS2, HSPA5, CDKN2A, NOS3, STAT1, NFATC1, MAPK14, ESR1, ESR2, SOD1, NFKBIA, FABP5, ALB, CDK2, BCL2, MDM2, CYCS, BAX, CTNNB1, CALM3, NFE2L2 | 5.07E-06 |
| CC | GO:0005737~cytoplasm | 85 | 44.73684 | 3.80E-07 | APP, GSK3B, NCF1, PTEN, HSPB1, PYGM, PLAT, AHR, ADRA1B, NR3C1, DCAF5, SLC6A3, PIK3CG, CASP9, IKBKB, PPP3CA, GJA1, CASP7, CASP8, CCND1, CA2, CHEK2, CASP3, CHEK1, AKT1, HSP90AA1, CHUK, PRKCB, MMP3, PRKCA, ADRA2C, SIRT1, ADRA2A, RUNX2, CCNA2, AR, RASA1, IRF1, BIRC5, PPARG, PGR, RAF1, TP53, ODC1, XIAP, ALOX12, CYP3A4, PTGS2, HIF1A, EGFR, RELA, PTGS1, MAPK8, CYP2B6, MAP2, HSF1, E2F1, MAPK1, APOD, LTA4H, EGLN1, PTPN1, NQO1, GSTM1, TGFB1, NOS2, HSPA5, CDKN2A, NOS3, STAT1, NFATC1, MAPK14, ESR1, SOD1, NFKBIA, FABP5, ALB, CDK2, BCL2, MDM2, BAX, CTNNB1, CALM3, NFE2L2, BCL2L1 | 1.00E-05 |
| CC | GO:0005886~plasma membrane | 84 | 44.21053 | 1.11E-07 | CHRM2, ACHE, APP, GSK3B, CHRM1, NCF1, SERPINE1, PTEN, HTR2A, SLC2A4, ADRA1B, SLC6A2, TNF, SLC6A3, PIK3CG, SLC6A4, ICAM1, THBD, PPP3CA, GJA1, PLAU, CA2, KDR, AKT1, OLR1, SCN5A, KCNH2, NCOA1, HSP90AA1, PRKCB, MMP2, DIO1, PRKCA, OPRM1, ADRA2C, F2, F3, ADRA2A, F7, AR, RASA1, KCNMA1, PGR, RAF1, MET, ABCG2, OPRD1, GRIA2, CHRNA2, CHRNA7, PTGER3, ADRB1, ADRB2, EGFR, DPP4, MAPK8, ERBB2, MAPK1, HAS2, DRD1, CD14, DUOX2, PTPN1, GABRA1, MGAM, TGFB1, VCAM1, NOS2, HSPA5, F10, NOS3, INSR, CAV1, SELE, ESR1, SOD1, NFKBIA, CD40LG, FABP5, MDM2, CTNNB1, CALM3, NOX5, NFE2L2 | 3.91E-06 |
| CC | GO:0005654~nucleoplasm | 74 | 38.94737 | 5.46E-10 | RB1, GSK3B, CDKN1A, PTEN, AKR1B1, AHR, NR3C1, DCAF5, MPO, ELK1, NR3C2, PPP3CA, GJA1, CASP7, CASP8, CCND1, MYC, CHEK2, CASP3, CHEK1, AKT1, OLR1, SCN5A, NCOA1, HSP90AA1, PARP1, CHUK, PRKCB, PRKCA, FOS, SIRT1, RUNX2, CCNA2, AR, IRF1, BIRC5, PPARG, PGR, TOP1, PPARA, TP53, ABCG2, PPARD, CHRNA2, PCNA, NR1I2, XIAP, HIF1A, RELA, MAPK8, RXRA, ALOX5, ERBB2, HSF1, E2F1, HMOX1, MAPK1, LTA4H, JUN, NOS2, CDKN2A, STAT1, NFATC1, MAPK14, ESR1, ESR2, SOD1, NFKBIA, FABP5, CDK2, MDM2, CTNNB1, CALM3, NFE2L2 | 3.46E-08 |
| MF | GO:0042802~identical protein binding | 63 | 33.15789 | 7.08E-20 | RB1, APP, PTEN, HSPB1, HTR2A, NR3C1, TNF, PIK3CG, SLC6A4, CASP9, IKBKB, CASP8, MYC, CHEK2, KDR, AKT1, OLR1, KCNH2, HSP90AA1, PARP1, FOS, MMP9, SIRT1, KCNMA1, BIRC5, PPARG, PGR, RAF1, MET, TP53, ABCG2, CRP, PCNA, MAOB, XIAP, ADRB2, ACACA, EGFR, RELA, DPP4, RXRA, ERBB2, HSF1, HMOX1, MAPK1, HAS2, NQO1, JUN, GSTM1, TGFB1, STAT1, INSR, CAV1, LYZ, ESR1, SOD1, NFKBIA, FABP5, ALB, BCL2, MDM2, BAX, BCL2L1 | 2.00E-17 |
| CC | GO:0005615~extracellular space | 57 | 30 | 2.74E-15 | ACHE, APP, CXCL8, SERPINE1, AKR1B1, HSPB1, PLAT, MPO, TNF, CXCL2, ICAM1, THBD, CASP7, PLAU, CHEK1, LBP, CTSD, IL10, MMP1, IGFBP3, MMP2, MMP3, PCOLCE, F2, MMP9, F3, IL1A, F7, IFNG, SLPI, IL1B, CRP, PON1, EGFR, ALOX5, SPP1, CCL2, HMOX1, APOD, CD14, XDH, TGFB1, VCAM1, F10, IGF2, LYZ, SELE, IL2, SOD1, IL4, CXCL10, COL3A1, CXCL11, IL6, CD40LG, FABP5, ALB | 8.69E-13 |
| CC | GO:0005576~extracellular region | 55 | 28.94737 | 5.07E-12 | ACHE, APP, CXCL8, SERPINE1, PTEN, PLAT, MPO, TNF, CXCL2, PLAU, KDR, OLR1, LBP, CTSD, IL10, HSP90AA1, MMP1, IGFBP3, MMP2, MMP3, PCOLCE, F2, MMP9, IL1A, F7, IFNG, SLPI, IL1B, MET, CRP, PON1, DPP4, ALOX5, SPP1, CCL2, MAPK1, APOD, CD14, LTA4H, MGAM, TGFB1, F10, IGF2, MAPK14, LYZ, IL2, SOD1, IL4, CXCL10, COL3A1, CXCL11, IL6, FABP5, ALB, CALM3 | 5.36E-10 |
| BP | GO:0045944~positive regulation of transcription from RNA polymerase II promoter | 54 | 28.42105 | 9.63E-21 | RB1, APP, AHR, NR3C1, ELK1, TNF, IKBKB, PPP3CA, MYC, AKT1, IL10, NCOA1, PARP1, CHUK, FOS, SIRT1, RUNX2, IL1A, AR, IRF1, IL1B, PPARG, PGR, RAF1, PPARA, MET, TP53, PPARD, NR1I2, ADRB2, HIF1A, EGFR, RELA, RXRA, HSF1, E2F1, EGLN1, JUN, TGFB1, HSPA5, CDKN2A, STAT1, IGF2, NFATC1, MAPK14, ESR1, ESR2, IL2, NFKBIA, IL4, CXCL10, IL6, CTNNB1, NFE2L2 | 8.22E-18 |
| CC | GO:0070062~extracellular exosome | 42 | 22.10526 | 1.27E-05 | APP, PCNA, PON1, SERPINE1, AKR1B1, HSPB1, PYGM, PLAT, ALOX12, SLC2A4, MPO, PTGS1, ICAM1, DPP4, PLAU, CA2, SPP1, APOD, LTA4H, LBP, CD14, CTSD, DUOX2, MGAM, HSP90AA1, VCAM1, HSPA5, PRKCB, INSR, AHSA1, PRKCA, PCOLCE, F2, LYZ, MMP9, SOD1, FABP5, SLPI, GSTA2, ALB, BAX, CTNNB1 | 1.92E-04 |
| BP | GO:0045893~positive regulation of transcription, DNA-templated | 42 | 22.10526 | 2.56E-20 | NCF1, NR1I2, AHR, ELK1, HIF1A, TNF, RELA, EGFR, IKBKB, RXRA, CHEK2, MYC, E2F1, SPP1, AKT1, IL10, NCOA1, KCNH2, JUN, TGFB1, CHUK, CDKN2A, PRKCB, STAT1, INSR, NFATC1, FOS, ESR1, RUNX2, ESR2, IL4, CCNA2, AR, IL6, IRF1, IL1B, CDK2, CTNNB1, PPARG, PPARA, TP53, PPARD | 1.64E-17 |
| MF | GO:0019899~enzyme binding | 40 | 21.05263 | 1.47E-28 | APP, PCNA, PTEN, CYP3A4, PTGS2, HIF1A, RELA, EGFR, PPP3CA, MAPK8, RXRA, CCND1, AKT1, HMOX1, SCN5A, EGLN1, PTPN1, JUN, GSTM1, TGFB1, PARP1, HSPA5, STAT1, CAV1, PRKCA, MAPK14, ESR1, SIRT1, ESR2, NFKBIA, AR, SLPI, CYP1A1, MDM2, BIRC5, CTNNB1, PPARG, PGR, RAF1, TP53 | 8.32E-26 |
| BP | GO:0010628~positive regulation of gene expression | 37 | 19.47368 | 5.24E-21 | CRP, APP, GSK3B, CXCL8, NR1I2, HIF1A, TNF, RELA, SLC6A4, PPP3CA, GJA1, MAPK8, MYC, ERBB2, HSF1, E2F1, AKT1, TGFB1, CDKN2A, NOS3, CAV1, MAPK14, F3, RUNX2, IL4, IL1A, AR, IL6, IFNG, IL1B, MDM2, CTNNB1, PPARG, PGR, TP53, PPARD, NFE2L2 | 6.71E-18 |
| BP | GO:0007165~signal transduction | 35 | 18.42105 | 5.45E-08 | GRIA2, CHRM1, CHRNA2, CXCL8, CHRNA7, NR1I2, NR3C1, HIF1A, EGFR, NR3C2, GJA1, MAPK8, PLAU, ERBB2, SPP1, CCL2, AKT1, MAPK1, IL10, PRKCB, STAT1, MAPK14, ESR1, ESR2, CXCL10, AR, CXCL11, RASA1, IL1B, CDK2, PPARG, PGR, RAF1, MET, PTGES | 3.10E-06 |
| BP | GO:0006915~apoptotic process | 33 | 17.36842 | 3.60E-15 | APP, PTEN, AHR, NR3C1, CASP9, CASP7, CASP8, CASP3, CHEK1, MAPK1, CD14, PARP1, CDKN2A, PRKCB, IGFBP3, MAPK14, MMP9, SOD1, NFKBIA, IL1A, IFNG, IRF1, IL1B, BCL2, MDM2, BAX, BIRC5, CYCS, NOX5, RAF1, TP53, BCL2L1, PPARD | 1.15E-12 |
| CC | GO:0005739~mitochondrion | 32 | 16.84211 | 6.78E-06 | MT-ND6, GSK3B, MAOB, MAOA, NR3C1, HK2, ACACA, CASP9, PPP3CA, GJA1, MAPK8, CASP8, RXRA, CYP1B1, AKT1, MAPK1, HSP90AA1, PARP1, HSPA5, CDKN2A, MMP2, PRKCA, MAPK14, SIRT1, ESR2, SOD1, BCL2, BAX, CYCS, RAF1, TP53, BCL2L1 | 1.07E-04 |
| CC | GO:0000785~chromatin | 32 | 16.84211 | 2.36E-08 | RB1, PCNA, NR1I2, AHR, NR3C1, ELK1, HIF1A, RELA, NR3C2, RXRA, MYC, CHEK1, HSF1, E2F1, NCOA1, JUN, PARP1, STAT1, NFATC1, FOS, ESR1, SIRT1, RUNX2, ESR2, AR, IRF1, PPARG, PGR, PPARA, TP53, PPARD, NFE2L2 | 1.07E-06 |
| CC | GO:0032991~macromolecular complex | 32 | 16.84211 | 2.64E-13 | CDKN1A, AHR, NR3C1, PTGS2, HIF1A, EGFR, CASP9, CASP7, CASP8, MYC, CASP3, CHEK1, E2F1, AKT1, NCOA1, PTPN1, HSP90AA1, PARP1, HSPA5, CDKN2A, STAT1, CAV1, ESR1, SOD1, AR, ALB, BCL2, MDM2, BIRC5, CTNNB1, CALM3, TP53 | 4.19E-11 |
| BP | GO:0043066~negative regulation of apoptotic process | 32 | 16.84211 | 2.57E-16 | GSK3B, CDKN1A, XIAP, AKR1B1, HSPB1, MPO, TNF, RELA, EGFR, MAPK8, MYC, ERBB2, KDR, AKT1, IL10, NQO1, HSPA5, MMP9, SIRT1, IL2, IL4, IL6, CD40LG, RASA1, PDE3A, BCL2, MDM2, BIRC5, CTNNB1, RAF1, TP53, BCL2L1 | 1.32E-13 |
| BP | GO:0000122~negative regulation of transcription from RNA polymerase II promoter | 30 | 15.78947 | 1.96E-07 | RB1, PCNA, NR1I2, NR3C1, TNF, RELA, RXRA, CCND1, MYC, HSF1, E2F1, JUN, TGFB1, PARP1, STAT1, CAV1, IGF2, ESR1, SIRT1, ESR2, IL4, AR, IFNG, CDK2, MDM2, CTNNB1, PPARG, PPARA, TP53, PPARD | 1.05E-05 |
| BP | GO:0008284~positive regulation of cell proliferation | 30 | 15.78947 | 1.49E-14 | CHRNA7, ODC1, PTEN, HTR2A, RELA, EGFR, DPP4, MYC, ERBB2, KDR, AKT1, HAS2, TGFB1, INSR, IGF2, F2, SIRT1, ADRA2A, IL2, IL4, CXCL10, AR, IL6, IFNG, IL1B, CDK2, BCL2, MDM2, BIRC5, CTNNB1 | 3.83E-12 |
| BP | GO:0009410~response to xenobiotic stimulus | 30 | 15.78947 | 6.98E-23 | CDKN1A, MAOB, AHR, HTR2A, PTGS2, SLC6A2, TNF, SLC6A3, RELA, SLC6A4, CCND1, MYC, CASP3, DRD1, IL10, HSP90AA1, TGFB1, CHUK, PRKCB, STAT1, MMP2, FOS, SOD1, PDE3A, BCL2, MDM2, CTNNB1, PPARG, TOP1, TP53 | 1.79E-19 |
| MF | GO:0003677~DNA binding | 29 | 15.26316 | 1.88E-04 | APP, PCNA, AHR, NR3C1, RELA, MYC, HSF1, E2F1, MAPK1, JUN, PARP1, CDKN2A, STAT1, NFATC1, FOS, ESR1, RUNX2, ESR2, AR, SLPI, IRF1, ALB, PPARG, PGR, TOP1, PPARA, TP53, PPARD, NFE2L2 | 0.002616 |
| MF | GO:0042803~protein homodimerization activity | 29 | 15.26316 | 1.55E-09 | ACHE, CHRNA7, PON1, ODC1, HSPB1, AHR, ADRB2, PTGS2, RELA, DPP4, IKBKB, CHEK2, AKT1, HMOX1, XDH, KCNH2, HSP90AA1, GSTM1, PARP1, NOS2, CHUK, STAT1, ADRA2C, ADRA2A, BCL2, BAX, BIRC5, ABCG2, BCL2L1 | 9.73E-08 |
| MF | GO:0000978~RNA polymerase II core promoter proximal region sequence-specific DNA binding | 27 | 14.21053 | 1.10E-04 | APP, NR1I2, NR3C1, ELK1, HIF1A, RELA, RXRA, MYC, HSF1, E2F1, JUN, STAT1, NFATC1, FOS, ESR1, SIRT1, RUNX2, ESR2, AR, IRF1, PPARG, PGR, TOP1, PPARA, TP53, PPARD, NFE2L2 | 0.001633 |
| MF | GO:0000981~RNA polymerase II transcription factor activity, sequence-specific DNA binding | 26 | 13.68421 | 7.68E-04 | NR1I2, AHR, NR3C1, ELK1, HIF1A, RELA, NR3C2, RXRA, MYC, HSF1, E2F1, JUN, STAT1, NFATC1, FOS, ESR1, RUNX2, ESR2, AR, IRF1, PPARG, PGR, PPARA, TP53, PPARD, NFE2L2 | 0.008682 |
| MF | GO:0003700~transcription factor activity, sequence-specific DNA binding | 26 | 13.68421 | 2.24E-09 | NR1I2, AHR, NR3C1, ELK1, HIF1A, RELA, NR3C2, RXRA, MYC, HSF1, E2F1, JUN, STAT1, NFATC1, FOS, ESR1, RUNX2, ESR2, AR, IRF1, PPARG, PGR, PPARA, TP53, PPARD, NFE2L2 | 1.27E-07 |
| BP | GO:0006954~inflammatory response | 26 | 13.68421 | 4.63E-13 | CRP, CXCL8, PTGER3, TNF, CXCL2, RELA, PIK3CG, IKBKB, CCL2, AKT1, OLR1, CD14, TGFB1, VCAM1, NOS2, CHUK, FOS, LYZ, SELE, IL1A, CXCL10, CXCL11, IL6, CD40LG, IL1B, NFE2L2 | 8.91E-11 |
| MF | GO:0008270~zinc ion binding | 25 | 13.15789 | 9.60E-06 | NR1I2, NR3C1, NR3C2, RXRA, CA2, LTA4H, PTPN1, PARP1, PRKCB, MMP1, MMP2, MMP3, PRKCA, MMP9, ESR1, ESR2, SOD1, AR, MDM2, BIRC5, PPARG, PGR, PPARA, TP53, PPARD | 1.87E-04 |
| BP | GO:0043065~positive regulation of apoptotic process | 25 | 13.15789 | 1.15E-14 | PTGS2, TNF, CASP9, MAPK8, CASP8, CASP3, E2F1, CYP1B1, CTSD, NCOA1, JUN, TGFB1, CDKN2A, MMP2, IGFBP3, MMP9, SIRT1, SOD1, IL6, KCNMA1, BCL2, BAX, CTNNB1, PPARG, TP53 | 3.26E-12 |
| MF | GO:0019901~protein kinase binding | 24 | 12.63158 | 6.17E-10 | PTPN1, GSK3B, CDKN1A, PARP1, CDKN2A, CAV1, HSPB1, NR3C1, HIF1A, ESR1, EGFR, RELA, ADRA2A, CASP9, CCNA2, IKBKB, CCND1, CHEK2, HSF1, AKT1, CTNNB1, CALM3, SCN5A, BCL2L1 | 4.36E-08 |
| CC | GO:0005783~endoplasmic reticulum | 23 | 12.10526 | 9.08E-04 | PTPN1, APP, VCAM1, HSPA5, F10, CAV1, AHSA1, PRKCA, FOS, OPRM1, PTGS2, GJA1, ALB, KDR, BCL2, BAX, HMOX1, NOX5, APOD, SCN5A, TP53, DUOX2, BCL2L1 | 0.007778 |
| CC | GO:0048471~perinuclear region of cytoplasm | 23 | 12.10526 | 1.53E-06 | KCNH2, ACHE, APP, CDKN1A, HSP90AA1, NOS2, STAT1, CAV1, NFATC1, PRKCA, SLC2A4, SELE, EGFR, ALOX5, HSF1, ERBB2, SPP1, HMOX1, CTNNB1, APOD, PPARG, SCN5A, PTGES | 3.23E-05 |
| CC | GO:0009986~cell surface | 23 | 12.10526 | 1.32E-07 | KCNH2, ACHE, APP, HSP90AA1, TGFB1, VCAM1, HSPA5, PLAT, SLC6A2, F3, TNF, SLC6A3, EGFR, ICAM1, DPP4, IL1A, THBD, CD40LG, PLAU, LBP, SCN5A, MET, DUOX2 | 4.19E-06 |
| BP | GO:0016310~phosphorylation | 22 | 11.57895 | 8.98E-07 | GSK3B, CDKN1A, CDKN2A, INSR, PRKCA, MAPK14, EGFR, PIK3CG, IKBKB, MAPK8, PLAU, CHEK2, ERBB2, CHEK1, CDK2, KDR, AKT1, MAPK1, CALM3, TOP1, RAF1, MET | 3.71E-05 |
| BP | GO:0045892~negative regulation of transcription, DNA-templated | 22 | 11.57895 | 2.24E-07 | RB1, JUN, TGFB1, PARP1, CDKN2A, NR1I2, AHR, NR3C1, SIRT1, TNF, RELA, RUNX2, IL4, IFNG, IRF1, MDM2, E2F1, BIRC5, CTNNB1, PPARG, TP53, PPARD | 1.10E-05 |
| BP | GO:0010629~negative regulation of gene expression | 22 | 11.57895 | 4.37E-12 | RB1, OPRD1, GSK3B, APP, CDKN1A, TGFB1, CXCL8, NOS2, HIF1A, ESR1, SIRT1, TNF, PPP3CA, GJA1, IFNG, HSF1, KDR, AKT1, CTNNB1, PGR, PPARG, XDH | 6.59E-10 |
| CC | GO:0005789~endoplasmic reticulum membrane | 21 | 11.05263 | 0.00424 | GRIA2, HSPA5, CAV1, PON1, DIO1, CYP3A4, PTGS2, EGFR, PTGS1, NR3C2, GJA1, CYP2B6, CYP1A1, BCL2, BAX, CYP1B1, HMOX1, HAS2, NOX5, DRD1, PTGES | 0.0286 |
| CC | GO:0005794~Golgi apparatus | 21 | 11.05263 | 0.002753 | ACHE, APP, VCAM1, NOS3, CAV1, ADRB2, OPRM1, ESR1, PTGS1, GJA1, CD40LG, SLPI, CHEK2, ALB, SPP1, KDR, MAPK1, HAS2, CD14, RAF1, NFE2L2 | 0.02182 |
| BP | GO:0008285~negative regulation of cell proliferation | 21 | 11.05263 | 9.77E-09 | IL10, APP, CDKN1A, TGFB1, CXCL8, CDKN2A, NOS3, IGFBP3, PTEN, OPRM1, PTGS2, IL1A, AR, IL6, IL1B, IRF1, CYP1B1, CTNNB1, RAF1, TP53, PTGES | 7.36E-07 |
| BP | GO:0071222~cellular response to lipopolysaccharide | 21 | 11.05263 | 3.20E-15 | IL10, CXCL8, NOS2, MMP3, SERPINE1, MAPK14, CXCL2, TNF, MMP9, RELA, IL1A, CXCL10, CXCL11, CASP7, IL6, MAPK8, IL1B, HSF1, CCL2, CD14, LBP | 1.15E-12 |
| BP | GO:0001666~response to hypoxia | 21 | 11.05263 | 6.71E-16 | EGLN1, TGFB1, VCAM1, NOS2, CHRNA7, CAV1, MMP2, PLAT, HIF1A, TNF, HK2, SLC6A4, CASP9, DPP4, IL1A, F7, PLAU, CASP3, KCNMA1, CYP1A1, PPARA | 2.86E-13 |
| BP | GO:0006508~proteolysis | 20 | 10.52632 | 6.36E-06 | APP, F10, MMP1, MMP2, MMP3, PLAT, PCOLCE, F2, MMP9, CASP9, DPP4, F7, THBD, CASP7, CASP8, PLAU, CASP3, OLR1, LTA4H, CTSD | 1.89E-04 |
| MF | GO:0031625~ubiquitin protein ligase binding | 20 | 10.52632 | 3.69E-10 | RB1, KCNH2, GSK3B, CDKN1A, JUN, HSP90AA1, HSPA5, PARP1, HIF1A, EGFR, RELA, NFKBIA, CASP8, CHEK2, MDM2, BCL2, CTNNB1, SCN5A, TP53, NFE2L2 | 3.48E-08 |
| BP | GO:0007186~G-protein coupled receptor signaling pathway | 19 | 10 | 0.006039 | CHRM2, OPRD1, CHRM1, CXCL8, INSR, PTGER3, F2, ADRA1B, ADRA2C, PIK3CG, ADRA2A, CXCL10, AR, PDE3A, CCL2, AKT1, CALM3, PGR, PPARG | 0.044826 |
| MF | GO:0001228~transcriptional activator activity, RNA polymerase II transcription regulatory region sequence-specific binding | 19 | 10 | 2.44E-06 | JUN, NR1I2, NFATC1, FOS, NR3C1, ELK1, HIF1A, ESR1, RELA, RUNX2, AR, MYC, IRF1, HSF1, PGR, PPARG, PPARA, TP53, NFE2L2 | 6.00E-05 |
| BP | GO:0030335~positive regulation of cell migration | 19 | 10 | 8.35E-11 | TGFB1, HSPA5, F10, CAV1, MMP2, INSR, PRKCA, F3, EGFR, ADRA2A, IL4, F7, PPP3CA, CASP8, PLAU, IL1B, KDR, HAS2, DRD1 | 9.29E-09 |
| MF | GO:0004672~protein kinase activity | 18 | 9.473684 | 3.53E-07 | GSK3B, CHUK, PRKCB, PRKCA, MAPK14, EGFR, PIK3CG, IKBKB, MAPK8, CCND1, CHEK2, ERBB2, CHEK1, CDK2, CCL2, AKT1, RAF1, MET | 1.33E-05 |
| BP | GO:0006468~protein phosphorylation | 18 | 9.473684 | 2.15E-07 | GSK3B, APP, CHUK, PRKCB, INSR, IGFBP3, PRKCA, IKBKB, MAPK8, CHEK2, ERBB2, CHEK1, CDK2, CCL2, BIRC5, AKT1, MAPK1, RAF1 | 1.08E-05 |
| BP | GO:0001525~angiogenesis | 18 | 9.473684 | 5.11E-10 | CXCL8, NOS3, CAV1, MMP2, SERPINE1, PRKCA, PTGS2, MAPK14, HIF1A, SIRT1, PIK3CG, CASP8, KDR, CYP1B1, CCL2, HMOX1, NOX5, APOD | 4.85E-08 |
| BP | GO:0032496~response to lipopolysaccharide | 18 | 9.473684 | 3.40E-14 | NQO1, VCAM1, MAOB, NOS2, CHUK, NOS3, FOS, PTGS2, SELE, MPO, CASP9, THBD, CASP8, SLPI, IL1B, CASP3, CYP1A1, LBP | 7.91E-12 |
| CC | GO:0005813~centrosome | 17 | 8.947368 | 3.60E-04 | GSK3B, PCNA, PRKCB, NR3C1, HK2, CCNA2, CCND1, HSF1, CHEK1, CDK2, E2F1, MAPK1, CTNNB1, CALM3, TP53, NFE2L2, BCL2L1 | 0.003805 |
| MF | GO:0005102~receptor binding | 17 | 8.947368 | 1.47E-06 | APP, CAV1, SERPINE1, PLAT, F2, SLC6A3, DPP4, CXCL10, F7, AR, GJA1, RASA1, ERBB2, CCL2, CTNNB1, PGR, LBP | 4.38E-05 |
| MF | GO:0043565~sequence-specific DNA binding | 17 | 8.947368 | 2.67E-07 | NR1I2, NR3C1, ELK1, HIF1A, ESR1, ESR2, NR3C2, AR, RXRA, HSF1, E2F1, BCL2, PGR, PPARG, PPARA, NFE2L2, PPARD | 1.08E-05 |
| BP | GO:0001934~positive regulation of protein phosphorylation | 17 | 8.947368 | 3.49E-10 | APP, CDKN1A, HSP90AA1, CHRNA7, INSR, F2, SIRT1, TNF, MMP9, EGFR, CCND1, IL1B, ERBB2, KDR, BIRC5, AKT1, RAF1 | 3.57E-08 |
| CC | GO:0045121~membrane raft | 17 | 8.947368 | 1.06E-10 | APP, CAV1, SLC2A4, SELE, TNF, SLC6A3, EGFR, SLC6A4, ICAM1, DPP4, IKBKB, GJA1, KDR, OLR1, CD14, CTSD, ABCG2 | 8.37E-09 |
| BP | GO:0043410~positive regulation of MAPK cascade | 17 | 8.947368 | 8.95E-12 | TGFB1, CHRNA7, INSR, IGFBP3, IGF2, ADRB2, ADRA1B, ADRA2C, TNF, ADRA2A, SOD1, AR, IL6, ERBB2, KDR, CTNNB1, RAF1 | 1.21E-09 |
| BP | GO:0045766~positive regulation of angiogenesis | 17 | 8.947368 | 6.17E-12 | CXCL8, PRKCB, NOS3, CHRNA7, SERPINE1, HSPB1, PRKCA, F3, HIF1A, SIRT1, HK2, IL1A, IL1B, KDR, CYP1B1, HMOX1, NFE2L2 | 8.78E-10 |
| BP | GO:0071456~cellular response to hypoxia | 17 | 8.947368 | 5.16E-13 | EGLN1, OPRD1, TGFB1, FOS, SLC2A4, PTGS2, HIF1A, SIRT1, CCNA2, MYC, MDM2, BCL2, HMOX1, PPARG, TP53, NFE2L2, PPARD | 8.91E-11 |
| BP | GO:0071356~cellular response to tumor necrosis factor | 17 | 8.947368 | 4.11E-13 | VCAM1, CXCL8, CHUK, NFATC1, FOS, SLC2A4, MAPK14, SIRT1, RELA, NFKBIA, IKBKB, CYP1B1, CCL2, AKT1, MAPK1, HAS2, NFE2L2 | 8.77E-11 |
| MF | GO:0046982~protein heterodimerization activity | 16 | 8.421053 | 5.90E-06 | CHUK, CAV1, ADRB1, AHR, ADRA1B, ADRA2C, HIF1A, ADRA2A, IKBKB, HSF1, ERBB2, BCL2, BAX, BIRC5, TP53, BCL2L1 | 1.24E-04 |
| CC | GO:0009897~external side of plasma membrane | 16 | 8.421053 | 5.89E-06 | GRIA2, VCAM1, F10, INSR, SLC2A4, F2, SELE, F3, TNF, ICAM1, CXCL10, THBD, CD40LG, PLAU, KDR, CD14 | 9.83E-05 |
| CC | GO:0043025~neuronal cell body | 16 | 8.421053 | 1.51E-06 | CHRM2, NQO1, GRIA2, HSP90AA1, TGFB1, MAOB, NCF1, HTR2A, ELK1, TNF, SLC6A3, ADRA2A, SOD1, MAP2, CASP3, APOD | 3.23E-05 |
| MF | GO:0061629~RNA polymerase II sequence-specific DNA binding transcription factor binding | 16 | 8.421053 | 5.71E-10 | RB1, GSK3B, JUN, PARP1, CDKN2A, HSPB1, NFATC1, AHR, FOS, ELK1, HIF1A, AR, CTNNB1, PPARA, TP53, NFE2L2 | 4.36E-08 |
| MF | GO:0003682~chromatin binding | 15 | 7.894737 | 4.07E-04 | NCOA1, PCNA, PARP1, PRKCB, FOS, MPO, ELK1, ESR1, EGFR, RELA, AR, CTNNB1, PPARG, TOP1, TP53 | 0.00516 |
| CC | GO:0045202~synapse | 15 | 7.894737 | 2.61E-04 | CHRM2, NQO1, ACHE, GABRA1, APP, CHRM1, CHRNA2, CHRNA7, OPRM1, NR3C1, SLC6A4, MAPK8, FABP5, MAPK1, CTNNB1 | 0.002906 |
| BP | GO:0035556~intracellular signal transduction | 15 | 7.894737 | 8.01E-05 | GSK3B, CXCL8, PRKCB, HSPB1, NFATC1, PRKCA, ADRA1B, MAPK14, RELA, RASA1, ERBB2, CHEK1, AKT1, HMOX1, MAPK1 | 0.001496 |
| MF | GO:0004674~protein serine/threonine kinase activity | 15 | 7.894737 | 3.70E-05 | GSK3B, CHUK, PRKCB, PRKCA, MAPK14, PIK3CG, IKBKB, MAPK8, CHEK2, CHEK1, CDK2, AKT1, MAPK1, TOP1, RAF1 | 5.97E-04 |
| BP | GO:0043123~positive regulation of I-kappaB kinase/NF-kappaB signaling | 15 | 7.894737 | 2.69E-08 | TGFB1, PARP1, CHUK, PRKCB, CAV1, XIAP, TNF, RELA, IKBKB, IL1A, GJA1, CASP8, IL1B, HMOX1, CTNNB1 | 1.77E-06 |
| MF | GO:1990837~sequence-specific double-stranded DNA binding | 14 | 7.368421 | 0.004228 | JUN, NR1I2, NFATC1, AHR, FOS, NR3C1, ELK1, ESR1, RUNX2, NR3C2, RXRA, HSF1, E2F1, PPARD | 0.034831 |
| BP | GO:0006955~immune response | 14 | 7.368421 | 0.001272 | IL10, OPRD1, CXCL8, CHUK, TNF, IL2, PIK3CG, IL4, IL1A, CD40LG, IFNG, SLPI, IL1B, CCL2 | 0.01324 |
| MF | GO:0044877~macromolecular complex binding | 14 | 7.368421 | 3.13E-05 | CDKN1A, TGFB1, PCNA, CHUK, CAV1, INSR, ADRB2, HTR2A, SLC6A3, PPP3CA, CASP8, MYC, CASP3, PPARA | 5.21E-04 |
| MF | GO:0000976~transcription regulatory region sequence-specific DNA binding | 14 | 7.368421 | 7.75E-07 | KCNH2, JUN, AHR, FOS, ELK1, TNF, RELA, AR, RXRA, IRF1, HSF1, PPARG, TP53, NFE2L2 | 2.43E-05 |
| BP | GO:0007507~heart development | 14 | 7.368421 | 1.63E-07 | CDKN1A, TGFB1, PCNA, MMP2, PTEN, IL1A, COL3A1, CASP7, GJA1, CASP8, CASP3, ERBB2, PPARG, PPARA | 9.10E-06 |
| BP | GO:0042127~regulation of cell proliferation | 14 | 7.368421 | 2.11E-09 | JUN, TGFB1, NOS2, STAT1, XIAP, SIRT1, EGFR, PTGS1, NFKBIA, CXCL10, CXCL11, PLAU, ERBB2, CHEK1 | 1.86E-07 |
| BP | GO:0045087~innate immune response | 13 | 6.842105 | 0.00607 | CRP, NQO1, PARP1, NCF1, CHUK, MMP3, PIK3CG, RELA, IKBKB, SLPI, PPARG, CD14, LBP | 0.044929 |
| CC | GO:0030425~dendrite | 13 | 6.842105 | 8.38E-04 | CHRM2, NQO1, GRIA2, GSK3B, CHRM1, MAOB, NCF1, STAT1, HTR2A, OPRM1, ELK1, MAP2, APOD | 0.007383 |
| CC | GO:0098978~glutamatergic synapse | 13 | 6.842105 | 4.87E-04 | CHRM2, EGLN1, GSK3B, CHRM1, PLAT, HTR2A, MAPK14, RELA, ADRA2A, PPP3CA, AKT1, CTNNB1, DRD1 | 0.004869 |
| BP | GO:0006974~cellular response to DNA damage stimulus | 13 | 6.842105 | 3.76E-05 | CDKN1A, PARP1, XIAP, SIRT1, CASP9, CCND1, CHEK2, MYC, CASP3, CHEK1, BCL2, MAPK1, TP53 | 7.90E-04 |
| BP | GO:0042981~regulation of apoptotic process | 13 | 6.842105 | 3.44E-06 | HSP90AA1, STAT1, XIAP, SIRT1, CASP9, CXCL10, CASP8, BAX, BIRC5, AKT1, RAF1, TP53, BCL2L1 | 1.21E-04 |
| MF | GO:0019904~protein domain specific binding | 13 | 6.842105 | 2.00E-06 | HSPA5, INSR, HIF1A, RUNX2, CCNA2, IL1B, CHEK1, CDK2, MDM2, TOP1, SCN5A, PPARA, NFE2L2 | 5.22E-05 |
| CC | GO:0005667~transcription factor complex | 13 | 6.842105 | 1.19E-06 | NCOA1, JUN, PARP1, NR1I2, NFATC1, AHR, ESR1, RELA, RUNX2, RXRA, CDK2, CTNNB1, TP53 | 2.89E-05 |
| MF | GO:0020037~heme binding | 13 | 6.842105 | 5.31E-08 | NOS2, NOS3, CYP3A4, PTGS2, MPO, PTGS1, CYP2B6, CYP1A1, CYP1B1, HMOX1, CYCS, NOX5, DUOX2 | 2.50E-06 |
| BP | GO:0045471~response to ethanol | 13 | 6.842105 | 3.95E-10 | NQO1, VCAM1, MAOB, PRKCB, FOS, SLC2A4, TNF, SLC6A3, IL2, SOD1, CASP8, CD14, PPARA | 3.89E-08 |
| BP | GO:0032355~response to estradiol | 13 | 6.842105 | 2.56E-11 | NCOA1, NQO1, TGFB1, PCNA, PTGS2, ESR1, SLC6A4, CASP9, F7, CASP8, CASP3, CYP1B1, CTNNB1 | 3.28E-09 |
| BP | GO:1902895~positive regulation of pri-miRNA transcription from RNA polymerase II promoter | 13 | 6.842105 | 5.22E-13 | IL10, JUN, TGFB1, FOS, NR3C1, HIF1A, TNF, EGFR, RELA, AR, MYC, PPARG, TP53 | 8.91E-11 |
| CC | GO:0016324~apical plasma membrane | 12 | 6.315789 | 0.001309 | DPP4, HSP90AA1, GJA1, MGAM, CAV1, ERBB2, KCNMA1, PTEN, ADRB2, EGFR, DUOX2, ABCG2 | 0.010637 |
| BP | GO:0007166~cell surface receptor signaling pathway | 12 | 6.315789 | 3.72E-04 | CXCL10, IFNG, STAT1, ERBB2, MAPK1, CCL2, CD14, ADRB2, F2, MAPK14, MET, EGFR | 0.005123 |
| CC | GO:0005788~endoplasmic reticulum lumen | 12 | 6.315789 | 1.48E-04 | F7, APP, COL3A1, IL6, HSPA5, F10, IGFBP3, ALB, SPP1, MAPK1, F2, PTGS2 | 0.001878 |
| CC | GO:0043005~neuron projection | 12 | 6.315789 | 1.41E-04 | OPRD1, GABRA1, CHRNA2, MAP2, CHRNA7, PTEN, OPRM1, PTGS2, SLC6A2, SLC6A3, SLC6A4, PTGS1 | 0.001865 |
| BP | GO:0070374~positive regulation of ERK1 and ERK2 cascade | 12 | 6.315789 | 1.38E-05 | IL1A, APP, TGFB1, CHRNA7, KDR, CCL2, PRKCA, HTR2A, OPRM1, TNF, EGFR, ICAM1 | 3.61E-04 |
| CC | GO:0043235~receptor complex | 12 | 6.315789 | 4.34E-06 | APP, RXRA, INSR, ERBB2, KDR, OLR1, PPARG, ADRB2, MET, EGFR, ADRA2A, NR3C2 | 8.00E-05 |
| CC | GO:0045211~postsynaptic membrane | 12 | 6.315789 | 2.72E-06 | CHRM2, GABRA1, GRIA2, CHRM1, CHRNA2, CHRNA7, KCNMA1, CTNNB1, DRD1, HTR2A, SLC6A3, SLC6A4 | 5.38E-05 |
| BP | GO:0006979~response to oxidative stress | 12 | 6.315789 | 3.61E-08 | NQO1, APP, MAPK8, HMOX1, AKT1, PTGS2, MPO, SIRT1, TP53, DUOX2, NFE2L2, PTGS1 | 2.20E-06 |
| BP | GO:0009636~response to toxic substance | 12 | 6.315789 | 2.67E-10 | NQO1, CDKN1A, MAOB, CHUK, PON1, MDM2, BCL2, BAX, CYP1B1, FOS, AHR, SLC6A4 | 2.85E-08 |
| BP | GO:0043525~positive regulation of neuron apoptotic process | 12 | 6.315789 | 3.43E-11 | CASP9, NQO1, GSK3B, CASP7, CASP8, CASP3, BAX, CTNNB1, HTR2A, NR3C1, TNF, TP53 | 4.19E-09 |
| MF | GO:0004879~RNA polymerase II transcription factor activity, ligand-activated sequence-specific DNA binding | 12 | 6.315789 | 4.96E-12 | AR, RXRA, NR1I2, PGR, PPARG, AHR, PPARA, NR3C1, ESR1, ESR2, NR3C2, PPARD | 7.01E-10 |
| CC | GO:0005925~focal adhesion | 11 | 5.789474 | 0.00649 | DPP4, GJA1, HSPA5, PLAU, CAV1, HSPB1, MAPK1, CTNNB1, EGFR, SLC6A4, ICAM1 | 0.041989 |
| MF | GO:0016301~kinase activity | 11 | 5.789474 | 1.47E-04 | GSK3B, CDKN1A, PLAU, CDKN2A, CHEK2, ERBB2, AKT1, CALM3, PRKCA, EGFR, PIK3CG | 0.002134 |
| MF | GO:0005125~cytokine activity | 11 | 5.789474 | 2.55E-05 | IL10, IL4, IL1A, IL6, TGFB1, CD40LG, IFNG, IL1B, SPP1, TNF, IL2 | 4.36E-04 |
| MF | GO:0004252~serine-type endopeptidase activity | 11 | 5.789474 | 1.94E-05 | DPP4, F7, F10, PLAU, MMP1, MMP2, MMP3, PLAT, F2, F3, MMP9 | 3.54E-04 |
| BP | GO:0051897~positive regulation of protein kinase B signaling | 11 | 5.789474 | 1.57E-05 | TGFB1, NCF1, INSR, IGF2, KDR, F2, SIRT1, TNF, EGFR, PIK3CG, PPARD | 3.94E-04 |
| BP | GO:0018105~peptidyl-serine phosphorylation | 11 | 5.789474 | 4.17E-06 | IKBKB, GSK3B, MAPK8, CHUK, PRKCB, CDK2, MAPK1, AKT1, PRKCA, TOP1, MAPK14 | 1.37E-04 |
| MF | GO:0005178~integrin binding | 11 | 5.789474 | 4.15E-06 | COL3A1, VCAM1, CD40LG, IL1B, SPP1, IGF2, KDR, PRKCA, EGFR, SLC6A4, ICAM1 | 9.40E-05 |
| BP | GO:0043524~negative regulation of neuron apoptotic process | 11 | 5.789474 | 2.06E-06 | CCND1, RASA1, BCL2, KDR, BAX, CCL2, BIRC5, HIF1A, SIRT1, BCL2L1, SOD1 | 7.66E-05 |
| BP | GO:0051091~positive regulation of sequence-specific DNA binding transcription factor activity | 11 | 5.789474 | 4.33E-08 | IL10, APP, IL6, IL1B, HSF1, PTEN, AKT1, PPARG, ESR1, TNF, ESR2 | 2.58E-06 |
| BP | GO:0051402~neuron apoptotic process | 11 | 5.789474 | 7.58E-09 | RB1, CASP9, APP, CASP7, TGFB1, CASP3, BCL2, XIAP, HIF1A, TP53, BCL2L1 | 6.06E-07 |
| BP | GO:0071466~cellular response to xenobiotic stimulus | 11 | 5.789474 | 1.16E-09 | RB1, KCNH2, PCNA, NOS2, MYC, IL1B, HSF1, E2F1, TP53, EGFR, NFE2L2 | 1.06E-07 |
| BP | GO:0034614~cellular response to reactive oxygen species | 11 | 5.789474 | 7.23E-11 | JUN, MAPK8, NCF1, CHUK, MMP2, MMP3, MAPK1, AKT1, FOS, MMP9, EGFR | 8.42E-09 |
| MF | GO:0001223~transcription coactivator binding | 11 | 5.789474 | 3.74E-11 | AR, STAT1, NFATC1, PGR, PPARG, AHR, PPARA, HIF1A, ESR1, RELA, PPARD | 4.22E-09 |
| BP | GO:0071276~cellular response to cadmium ion | 11 | 5.789474 | 6.98E-13 | JUN, MAPK8, NCF1, CHUK, HSF1, HMOX1, MAPK1, AKT1, FOS, MMP9, EGFR | 1.12E-10 |
| CC | GO:0030424~axon | 10 | 5.263158 | 0.005573 | GSK3B, APP, MAPK8, TGFB1, STAT1, INSR, HTR2A, OPRM1, SLC6A2, SLC6A3 | 0.036807 |
| BP | GO:0006366~transcription from RNA polymerase II promoter | 10 | 5.263158 | 6.45E-04 | RB1, AR, PARP1, IRF1, CTNNB1, FOS, ADRB2, MAPK14, ESR1, IL2 | 0.007616 |
| BP | GO:0006629~lipid metabolic process | 10 | 5.263158 | 4.91E-04 | CYP2B6, FABP5, PTEN, PDE3A, APOD, PPARG, ALOX12, LTA4H, CYP3A4, PPARD | 0.006251 |
| BP | GO:0007267~cell-cell signaling | 10 | 5.263158 | 4.76E-04 | CXCL10, AR, CXCL11, GJA1, IL1B, PGR, ADRA1B, ADRA2C, ESR2, IL2 | 0.0061 |
| CC | GO:0005741~mitochondrial outer membrane | 10 | 5.263158 | 2.03E-04 | CASP8, MAOB, MAOA, BCL2, BAX, HMOX1, PGR, RAF1, HK2, BCL2L1 | 0.002474 |
| BP | GO:0050728~negative regulation of inflammatory response | 10 | 5.263158 | 1.42E-05 | IL10, RB1, IL4, ALOX5, PPARG, AHR, PPARA, IL2, SOD1, PPARD | 3.66E-04 |
| CC | GO:0042734~presynaptic membrane | 10 | 5.263158 | 4.54E-06 | CHRM2, OPRD1, CHRM1, ERBB2, CTNNB1, DRD1, HTR2A, SLC6A2, SLC6A3, SLC6A4 | 8.00E-05 |
| MF | GO:0002020~protease binding | 10 | 5.263158 | 2.03E-06 | DPP4, GSK3B, COL3A1, CASP3, SERPINE1, BCL2, TNF, TP53, F3, SLC6A3 | 5.22E-05 |
| BP | GO:0032755~positive regulation of interleukin-6 production | 10 | 5.263158 | 7.30E-07 | IL1A, APP, IL6, TGFB1, IFNG, NOS2, IL1B, LBP, TNF, RELA | 3.11E-05 |
| MF | GO:0008233~peptidase activity | 10 | 5.263158 | 6.09E-07 | CASP9, APP, CASP7, CASP8, MMP1, CASP3, MMP3, LTA4H, CTSD, MMP9 | 2.02E-05 |
| BP | GO:0033138~positive regulation of peptidyl-serine phosphorylation | 10 | 5.263158 | 5.02E-08 | OPRD1, APP, IL6, CAV1, BCL2, AKT1, RAF1, PTGS2, TNF, EGFR | 2.92E-06 |
| CC | GO:0005901~caveola | 10 | 5.263158 | 2.93E-08 | NOS3, CAV1, INSR, KCNMA1, MAPK1, SCN5A, HTR2A, ADRA1B, PTGS2, SELE | 1.16E-06 |
| BP | GO:0001819~positive regulation of cytokine production | 10 | 5.263158 | 2.09E-08 | IL10, IL1A, IFNG, IL1B, NOX5, CD14, TNF, ADRA2A, PIK3CG, SOD1 | 1.49E-06 |
| BP | GO:0051726~regulation of cell cycle | 9 | 4.736842 | 0.002847 | RB1, CDKN1A, JUN, CDKN2A, IRF1, MDM2, BAX, XIAP, TP53 | 0.024884 |
| BP | GO:0001701~in utero embryonic development | 9 | 4.736842 | 0.001563 | AR, COL3A1, CDKN1A, GJA1, NOS3, IGF2, CTNNB1, TP53, BCL2L1 | 0.015579 |
| BP | GO:0019221~cytokine-mediated signaling pathway | 9 | 4.736842 | 1.04E-04 | IL1A, IL6, IL1B, CAV1, CCL2, AKT1, F3, RELA, DUOX2 | 0.001841 |
| BP | GO:0030308~negative regulation of cell growth | 9 | 4.736842 | 2.76E-05 | RB1, CDKN1A, GJA1, TGFB1, CDKN2A, BCL2, PPARG, TP53, ESR2 | 6.24E-04 |
| CC | GO:0090575~RNA polymerase II transcription factor complex | 9 | 4.736842 | 2.10E-05 | NCOA1, JUN, RXRA, STAT1, E2F1, PPARG, FOS, HIF1A, NFE2L2 | 3.02E-04 |
| BP | GO:0009615~response to virus | 9 | 4.736842 | 1.13E-05 | CHRM2, IKBKB, IFNG, CHUK, ODC1, HSPB1, TNF, DUOX2, BCL2L1 | 3.05E-04 |
| BP | GO:0098586~cellular response to virus | 9 | 4.736842 | 4.02E-06 | CXCL10, IL6, HSP90AA1, TGFB1, IFNG, CHUK, BAX, MAPK14, HIF1A | 1.36E-04 |
| BP | GO:0070301~cellular response to hydrogen peroxide | 9 | 4.736842 | 2.95E-07 | NQO1, IL6, PCNA, HSF1, MDM2, CYP1B1, SIRT1, RELA, NFE2L2 | 1.35E-05 |
| BP | GO:0000082~G1/S transition of mitotic cell cycle | 9 | 4.736842 | 2.95E-07 | RB1, CCNA2, PPP3CA, CDKN1A, CCND1, MYC, CDK2, BCL2, E2F1 | 1.35E-05 |
| BP | GO:0032757~positive regulation of interleukin-8 production | 9 | 4.736842 | 2.06E-07 | IL6, NOS2, IL1B, SERPINE1, CD14, LBP, TNF, F3, RELA | 1.05E-05 |
| BP | GO:0043536~positive regulation of blood vessel endothelial cell migration | 9 | 4.736842 | 2.37E-08 | TGFB1, NOS3, KDR, HSPB1, AKT1, PRKCA, HIF1A, SIRT1, NFE2L2 | 1.60E-06 |
| BP | GO:0045429~positive regulation of nitric oxide biosynthetic process | 9 | 4.736842 | 1.17E-08 | HSP90AA1, IFNG, IL1B, INSR, AKT1, OPRM1, PTGS2, ESR1, TNF | 8.59E-07 |
| BP | GO:0035094~response to nicotine | 9 | 4.736842 | 3.52E-09 | VCAM1, CHRNA2, CHRNA7, CASP3, MMP2, BCL2, HMOX1, MAPK1, SLC6A3 | 3.01E-07 |
| BP | GO:0010468~regulation of gene expression | 8 | 4.210526 | 0.006142 | IL10, APP, MYC, CDK2, MDM2, BCL2, AHR, HIF1A | 0.04533 |
| MF | GO:0005516~calmodulin binding | 8 | 4.210526 | 0.00491 | PPP3CA, NOS2, MAP2, NOS3, AKT1, SCN5A, ESR1, EGFR | 0.038527 |
| CC | GO:0005635~nuclear envelope | 8 | 4.210526 | 0.003214 | PARP1, ALOX5, MYC, INSR, PTGER3, CDK2, BAX, SIRT1 | 0.02369 |
| MF | GO:0046983~protein dimerization activity | 8 | 4.210526 | 0.002646 | IL10, NCOA1, PPP3CA, MYC, E2F1, AHR, HIF1A, ABCG2 | 0.024439 |
| MF | GO:0005506~iron ion binding | 8 | 4.210526 | 6.24E-04 | EGLN1, CYP2B6, ALOX5, CYP1A1, CYP1B1, ALOX12, CYP3A4, XDH | 0.00734 |
| BP | GO:0006935~chemotaxis | 8 | 4.210526 | 2.73E-04 | CXCL10, CXCL11, CXCL8, PLAU, MAPK1, CCL2, MAPK14, CXCL2 | 0.004115 |
| BP | GO:0001649~osteoblast differentiation | 8 | 4.210526 | 2.60E-04 | GJA1, IGFBP3, SPP1, IGF2, CTNNB1, AKT1, MAPK14, RUNX2 | 0.003951 |
| BP | GO:0006805~xenobiotic metabolic process | 8 | 4.210526 | 1.49E-04 | NQO1, CYP2B6, GSTA2, NR1I2, CYP1A1, CYP1B1, AHR, CYP3A4 | 0.002473 |
| BP | GO:0000165~MAPK cascade | 8 | 4.210526 | 1.49E-04 | AR, MYC, CAV1, IGFBP3, HSF1, CTNNB1, RAF1, EGFR | 0.002473 |
| BP | GO:0042593~glucose homeostasis | 8 | 4.210526 | 1.26E-04 | IL6, FABP5, ALOX5, INSR, AKT1, PPARG, SLC2A4, ADRA2A | 0.00217 |
| BP | GO:0032760~positive regulation of tumor necrosis factor production | 8 | 4.210526 | 1.13E-04 | IL1A, APP, IL6, TGFB1, IFNG, HSPB1, CD14, LBP | 0.001966 |
| BP | GO:0050729~positive regulation of inflammatory response | 8 | 4.210526 | 1.01E-04 | NFKBIA, APP, TGFB1, IFNG, IL1B, SERPINE1, TNF, IL2 | 0.001803 |
| BP | GO:0008584~male gonad development | 8 | 4.210526 | 8.95E-05 | NCOA1, AR, GJA1, INSR, BCL2, CYP1B1, ESR1, BCL2L1 | 0.00165 |
| BP | GO:0030593~neutrophil chemotaxis | 8 | 4.210526 | 1.74E-05 | CXCL10, CXCL11, CXCL8, IL1B, CCL2, LBP, CXCL2, PIK3CG | 4.28E-04 |
| BP | GO:0042060~wound healing | 8 | 4.210526 | 1.06E-05 | PPP3CA, COL3A1, CDKN1A, ERBB2, NFATC1, RAF1, PPARA, PPARD | 2.88E-04 |
| BP | GO:0071260~cellular response to mechanical stimulus | 8 | 4.210526 | 9.69E-06 | MAPK8, TGFB1, CASP8, IL1B, IRF1, CHEK1, PTGS2, EGFR | 2.70E-04 |
| BP | GO:0006919~activation of cysteine-type endopeptidase activity involved in apoptotic process | 8 | 4.210526 | 7.40E-06 | CASP9, CASP8, CDKN2A, BAX, PPARG, TNF, F3, XDH | 2.16E-04 |
| MF | GO:0097110~scaffold protein binding | 8 | 4.210526 | 6.34E-06 | KCNH2, IKBKB, HSP90AA1, GJA1, CASP8, CHUK, NOS3, SCN5A | 1.28E-04 |
| BP | GO:0007249~I-kappaB kinase/NF-kappaB signaling | 8 | 4.210526 | 5.07E-06 | NFKBIA, IKBKB, CHUK, IL1B, CTNNB1, AKT1, TNF, RELA | 1.58E-04 |
| BP | GO:0097190~apoptotic signaling pathway | 8 | 4.210526 | 4.59E-06 | CASP8, CASP3, CAV1, PDE3A, BAX, CTNNB1, PRKCA, PPARD | 1.47E-04 |
| BP | GO:0002931~response to ischemia | 8 | 4.210526 | 4.59E-06 | CASP9, NQO1, CAV1, BCL2, TP53, RELA, HK2, NFE2L2 | 1.47E-04 |
| BP | GO:0006006~glucose metabolic process | 8 | 4.210526 | 3.38E-06 | FABP5, IGF2, AKT1, APOD, MAPK14, TNF, HK2, PPARD | 1.20E-04 |
| BP | GO:0007584~response to nutrient | 8 | 4.210526 | 2.19E-06 | NQO1, VCAM1, STAT1, HSF1, CYP1B1, PPARG, PPARA, SLC6A4 | 8.01E-05 |
| BP | GO:0048661~positive regulation of smooth muscle cell proliferation | 8 | 4.210526 | 9.42E-07 | IL6, TGFB1, STAT1, HMOX1, AKT1, PTGS2, TNF, EGFR | 3.83E-05 |
| BP | GO:1904646~cellular response to beta-amyloid | 8 | 4.210526 | 2.60E-07 | GSK3B, APP, GJA1, VCAM1, PARP1, ADRB2, TNF, ICAM1 | 1.23E-05 |
| BP | GO:2000379~positive regulation of reactive oxygen species metabolic process | 8 | 4.210526 | 3.38E-08 | CDKN1A, CYP1B1, NOX5, F2, MAPK14, TP53, XDH, NFE2L2 | 2.11E-06 |
| BP | GO:0097192~extrinsic apoptotic signaling pathway in absence of ligand | 8 | 4.210526 | 3.38E-08 | IL4, IL1A, GSK3B, IL1B, BCL2, BAX, IL2, BCL2L1 | 2.11E-06 |
| BP | GO:0010575~positive regulation of vascular endothelial growth factor production | 8 | 4.210526 | 2.15E-08 | IL1A, IL6, TGFB1, IL1B, CYP1B1, PTGS2, HIF1A, RELA | 1.49E-06 |
| BP | GO:1902894~negative regulation of pri-miRNA transcription from RNA polymerase II promoter | 8 | 4.210526 | 7.81E-09 | TGFB1, PPARG, PPARA, HIF1A, ESR1, TNF, RELA, PPARD | 6.06E-07 |
| MF | GO:0008083~growth factor activity | 7 | 3.684211 | 0.006212 | IL10, IL4, IL6, TGFB1, IGF2, F2, IL2 | 0.04558 |
| BP | GO:0030198~extracellular matrix organization | 7 | 3.684211 | 0.004594 | APP, COL3A1, MMP1, MMP2, MMP3, TNF, MMP9 | 0.035979 |
| BP | GO:0007204~positive regulation of cytosolic calcium ion concentration | 7 | 3.684211 | 0.003114 | PTGER3, HTR2A, OPRM1, ADRA1B, ESR1, IL2, PIK3CG | 0.026854 |
| BP | GO:0051092~positive regulation of NF-kappaB transcription factor activity | 7 | 3.684211 | 0.002709 | IKBKB, AR, CD40LG, CHUK, IL1B, TNF, RELA | 0.023927 |
| MF | GO:0042826~histone deacetylase binding | 7 | 3.684211 | 0.00191 | HSP90AA1, MAPK8, CCND1, PARP1, HIF1A, TP53, RELA | 0.018481 |
| CC | GO:1904813~ficolin-1-rich granule lumen | 7 | 3.684211 | 0.001068 | HSP90AA1, ALOX5, MAPK1, LTA4H, MAPK14, CTSD, MMP9 | 0.008911 |
| BP | GO:0007179~transforming growth factor beta receptor signaling pathway | 7 | 3.684211 | 4.58E-04 | COL3A1, JUN, TGFB1, PARP1, FOS, SIRT1, TP53 | 0.0061 |
| BP | GO:0050727~regulation of inflammatory response | 7 | 3.684211 | 3.48E-04 | ALOX5, XIAP, PTGS2, SELE, ESR1, RELA, PTGES | 0.004844 |
| BP | GO:0032869~cellular response to insulin stimulus | 7 | 3.684211 | 3.10E-04 | RB1, PARP1, STAT1, INSR, AKT1, PPARG, SLC2A4 | 0.00445 |
| MF | GO:0019903~protein phosphatase binding | 7 | 3.684211 | 2.71E-04 | HSP90AA1, MAPK8, CTNNB1, PPARG, MAPK14, MET, EGFR | 0.003562 |
| BP | GO:0007596~blood coagulation | 7 | 3.684211 | 2.02E-04 | THBD, F7, F10, PLAU, PLAT, F2, F3 | 0.003221 |
| BP | GO:0043491~protein kinase B signaling | 7 | 3.684211 | 2.02E-04 | ERBB2, PTEN, AKT1, HTR2A, TNF, PIK3CG, PPARD | 0.003221 |
| BP | GO:0007265~Ras protein signal transduction | 7 | 3.684211 | 1.78E-04 | RB1, CCNA2, CDKN1A, CDKN2A, CDK2, TP53, ADRA2A | 0.002921 |
| BP | GO:0001938~positive regulation of endothelial cell proliferation | 7 | 3.684211 | 7.54E-05 | IL10, KDR, AKT1, PRKCA, HIF1A, SIRT1, F3 | 0.001419 |
| BP | GO:0008286~insulin receptor signaling pathway | 7 | 3.684211 | 5.93E-05 | GSK3B, PTPN1, INSR, IGF2, MAPK1, AKT1, RAF1 | 0.00115 |
| BP | GO:0007611~learning or memory | 7 | 3.684211 | 5.45E-05 | APP, CHRNA7, CASP3, PTEN, MAPK1, SLC2A4, EGFR | 0.001087 |
| BP | GO:0007623~circadian rhythm | 7 | 3.684211 | 5.45E-05 | GSK3B, F7, NOS2, TOP1, TNF, EGFR, SLC6A4 | 0.001087 |
| BP | GO:0032731~positive regulation of interleukin-1 beta production | 7 | 3.684211 | 4.22E-05 | APP, IL6, CASP8, IFNG, HSPB1, TNF, RELA | 8.71E-04 |
| BP | GO:0030168~platelet activation | 7 | 3.684211 | 3.86E-05 | COL3A1, IL6, CD40LG, F2, MAPK14, ADRA2C, ADRA2A | 8.04E-04 |
| BP | GO:0033209~tumor necrosis factor-mediated signaling pathway | 7 | 3.684211 | 2.66E-05 | NFKBIA, IKBKB, CHUK, STAT1, TNF, TP53, RELA | 6.09E-04 |
| BP | GO:0002062~chondrocyte differentiation | 7 | 3.684211 | 2.19E-05 | RB1, COL3A1, TGFB1, CTNNB1, MAPK14, HIF1A, RUNX2 | 5.24E-04 |
| BP | GO:0032091~negative regulation of protein binding | 7 | 3.684211 | 1.98E-05 | GSK3B, CDKN1A, MAPK8, SLPI, CAV1, BAX, AKT1 | 4.78E-04 |
| BP | GO:0043406~positive regulation of MAP kinase activity | 7 | 3.684211 | 1.78E-05 | IL1B, INSR, ERBB2, TNF, EGFR, ADRA2A, PIK3CG | 4.35E-04 |
| BP | GO:0034605~cellular response to heat | 7 | 3.684211 | 1.61E-05 | IL1A, CXCL10, CDKN1A, HSP90AA1, HSF1, HMOX1, PTGS2 | 3.99E-04 |
| BP | GO:0007187~G-protein coupled receptor signaling pathway, coupled to cyclic nucleotide second messenger | 7 | 3.684211 | 1.44E-05 | CHRM2, OPRD1, CHRM1, CCL2, DRD1, HTR2A, OPRM1 | 3.66E-04 |
| BP | GO:0048146~positive regulation of fibroblast proliferation | 7 | 3.684211 | 1.44E-05 | CCNA2, CDKN1A, TGFB1, MYC, E2F1, ESR1, EGFR | 3.66E-04 |
| BP | GO:1904707~positive regulation of vascular smooth muscle cell proliferation | 7 | 3.684211 | 1.29E-05 | IL10, GJA1, JUN, MMP2, MDM2, TNF, MMP9 | 3.41E-04 |
| BP | GO:0031334~positive regulation of protein complex assembly | 7 | 3.684211 | 1.29E-05 | GSK3B, TGFB1, IFNG, MMP1, MMP3, BAX, TNF | 3.41E-04 |
| BP | GO:0000086~G2/M transition of mitotic cell cycle | 7 | 3.684211 | 9.17E-06 | CCNA2, APP, CHEK2, CHEK1, CDK2, BIRC5, CALM3 | 2.58E-04 |
| BP | GO:0050673~epithelial cell proliferation | 7 | 3.684211 | 9.17E-06 | RB1, AR, TGFB1, KDR, EGFR, RUNX2, BCL2L1 | 2.58E-04 |
| BP | GO:0008630~intrinsic apoptotic signaling pathway in response to DNA damage | 7 | 3.684211 | 7.19E-06 | CASP9, CHEK2, BCL2, E2F1, BAX, TNF, BCL2L1 | 2.12E-04 |
| MF | GO:0051879~Hsp90 protein binding | 7 | 3.684211 | 5.47E-06 | HSF1, CYP1A1, KDR, AHSA1, AHR, NR3C1, HIF1A | 1.19E-04 |
| MF | GO:0016922~ligand-dependent nuclear receptor binding | 7 | 3.684211 | 4.16E-06 | NCOA1, RXRA, STAT1, NR1I2, CTNNB1, HIF1A, SIRT1 | 9.40E-05 |
| BP | GO:0007566~embryo implantation | 7 | 3.684211 | 3.72E-06 | IL1B, MMP2, SPP1, PTGS2, MMP9, SOD1, PPARD | 1.27E-04 |
| BP | GO:0032722~positive regulation of chemokine production | 7 | 3.684211 | 3.72E-06 | APP, IL6, IFNG, HMOX1, LBP, HIF1A, TNF | 1.27E-04 |
| BP | GO:0007595~lactation | 7 | 3.684211 | 2.79E-06 | NCOA1, CCND1, CAV1, HIF1A, XDH, SLC6A3, HK2 | 1.01E-04 |
| BP | GO:0042542~response to hydrogen peroxide | 7 | 3.684211 | 2.06E-06 | STAT1, CASP3, MMP2, BCL2, HMOX1, SIRT1, SOD1 | 7.66E-05 |
| MF | GO:0001221~transcription cofactor binding | 7 | 3.684211 | 1.96E-06 | RXRA, MYC, CTNNB1, PPARG, FOS, ESR1, NFE2L2 | 5.22E-05 |
| BP | GO:0071392~cellular response to estradiol stimulus | 7 | 3.684211 | 1.76E-06 | IL10, CCNA2, MMP2, HSF1, ESR1, ESR2, EGFR | 6.92E-05 |
| BP | GO:0031663~lipopolysaccharide-mediated signaling pathway | 7 | 3.684211 | 8.84E-07 | NFKBIA, NOS3, MAPK1, AKT1, CD14, LBP, MAPK14 | 3.71E-05 |
| BP | GO:0097421~liver regeneration | 7 | 3.684211 | 4.96E-07 | IL10, IL6, TGFB1, PCNA, CCND1, TNF, EGFR | 2.19E-05 |
| MF | GO:0005496~steroid binding | 7 | 3.684211 | 4.52E-07 | AR, PGR, CYP3A4, NR3C1, ESR1, ESR2, NR3C2 | 1.59E-05 |
| BP | GO:1902042~negative regulation of extrinsic apoptotic signaling pathway via death domain receptors | 7 | 3.684211 | 2.60E-07 | GSK3B, NOS3, SERPINE1, HMOX1, RAF1, BCL2L1, ICAM1 | 1.23E-05 |
| BP | GO:0071480~cellular response to gamma radiation | 7 | 3.684211 | 2.06E-07 | CDKN1A, CHEK2, HSF1, MDM2, ELK1, TP53, BCL2L1 | 1.05E-05 |
| BP | GO:0010332~response to gamma radiation | 7 | 3.684211 | 2.06E-07 | IL1A, CXCL10, PARP1, MYC, BCL2, BAX, TP53 | 1.05E-05 |
| MF | GO:0003707~steroid hormone receptor activity | 7 | 3.684211 | 8.14E-08 | RXRA, PGR, PPARA, ESR1, ESR2, NR3C2, PPARD | 3.54E-06 |
| BP | GO:0051146~striated muscle cell differentiation | 7 | 3.684211 | 6.09E-09 | RB1, CASP7, CHUK, CASP3, IGF2, AKT1, MAPK14 | 5.03E-07 |
| MF | GO:0003690~double-stranded DNA binding | 6 | 3.157895 | 0.004998 | JUN, RXRA, STAT1, PPARG, TOP1, EGFR | 0.038687 |
| MF | GO:0051087~chaperone binding | 6 | 3.157895 | 0.004104 | HSPA5, ALB, AHSA1, BIRC5, TP53, SOD1 | 0.034831 |
| CC | GO:0016605~PML body | 6 | 3.157895 | 0.003527 | RB1, CHEK2, HSF1, PTEN, SIRT1, TP53 | 0.024925 |
| BP | GO:0006606~protein import into nucleus | 6 | 3.157895 | 0.003199 | NFKBIA, PPP3CA, CDKN1A, AKT1, DRD1, TP53 | 0.027495 |
| CC | GO:0042383~sarcolemma | 6 | 3.157895 | 0.003131 | PPP3CA, VCAM1, CAV1, ALOX12, SLC2A4, SCN5A | 0.023635 |
| BP | GO:0046330~positive regulation of JNK cascade | 6 | 3.157895 | 0.002686 | IL1A, APP, NCF1, IL1B, XIAP, TNF | 0.023803 |
| BP | GO:0007200~phospholipase C-activating G-protein coupled receptor signaling pathway | 6 | 3.157895 | 0.002568 | OPRD1, PTGER3, HTR2A, OPRM1, ADRA1B, ESR1 | 0.023111 |
| BP | GO:0030307~positive regulation of cell growth | 6 | 3.157895 | 0.001752 | ERBB2, BCL2, AKT1, F2, EGFR, IL2 | 0.017395 |
| MF | GO:0051117~ATPase binding | 6 | 3.157895 | 0.001552 | PPP3CA, AR, CAV1, PGR, ESR1, EGFR | 0.015946 |
| BP | GO:0007613~memory | 6 | 3.157895 | 0.001502 | CHRNA7, INSR, DRD1, HTR2A, PTGS2, SLC6A4 | 0.015029 |
| BP | GO:0001933~negative regulation of protein phosphorylation | 6 | 3.157895 | 0.001211 | CDKN1A, TGFB1, IGFBP3, PTEN, XDH, IL2 | 0.012816 |
| BP | GO:0071347~cellular response to interleukin-1 | 6 | 3.157895 | 0.001145 | CXCL8, MMP2, CCL2, HAS2, HIF1A, RELA | 0.01217 |
| BP | GO:0032868~response to insulin | 6 | 3.157895 | 8.55E-04 | IL10, FOS, MAPK14, PPARA, SIRT1, RELA | 0.009607 |
| CC | GO:0045177~apical part of cell | 6 | 3.157895 | 8.16E-04 | APP, VCAM1, CA2, CTNNB1, PLAT, DUOX2 | 0.007383 |
| MF | GO:0002039~p53 binding | 6 | 3.157895 | 7.27E-04 | GSK3B, CDKN2A, MDM2, HIF1A, SIRT1, TP53 | 0.00838 |
| BP | GO:0030163~protein catabolic process | 6 | 3.157895 | 6.24E-04 | NQO1, CASP7, CHEK2, CASP3, MMP2, RELA | 0.007397 |
| MF | GO:0050660~flavin adenine dinucleotide binding | 6 | 3.157895 | 5.96E-04 | MAOB, NOS2, MAOA, NOS3, NOX5, XDH | 0.007165 |
| BP | GO:0071560~cellular response to transforming growth factor beta stimulus | 6 | 3.157895 | 5.84E-04 | TGFB1, CAV1, PDE3A, NFATC1, FOS, NR3C1 | 0.007121 |
| BP | GO:0050679~positive regulation of epithelial cell proliferation | 6 | 3.157895 | 5.84E-04 | TGFB1, MYC, ERBB2, SCN5A, EGFR, RUNX2 | 0.007121 |
| BP | GO:1901224~positive regulation of NIK/NF-kappaB signaling | 6 | 3.157895 | 5.10E-04 | APP, IL1B, TNF, RELA, EGFR, NR3C2 | 0.006463 |
| BP | GO:0042531~positive regulation of tyrosine phosphorylation of STAT protein | 6 | 3.157895 | 4.75E-04 | IL4, IL6, IFNG, HSF1, TNF, IL2 | 0.0061 |
| BP | GO:0006959~humoral immune response | 6 | 3.157895 | 3.83E-04 | IL6, IFNG, ALOX5, BCL2, CCL2, TNF | 0.005244 |
| CC | GO:0000791~euchromatin | 6 | 3.157895 | 2.66E-04 | JUN, HSF1, CTNNB1, HIF1A, ESR1, SIRT1 | 0.002906 |
| CC | GO:0043679~axon terminus | 6 | 3.157895 | 2.46E-04 | CHRM2, OPRD1, CHRM1, ELK1, SLC6A3, ADRA2A | 0.00289 |
| BP | GO:0034644~cellular response to UV | 6 | 3.157895 | 2.39E-04 | CASP9, PCNA, PARP1, MYC, BAX, TP53 | 0.003713 |
| BP | GO:0090398~cellular senescence | 6 | 3.157895 | 2.39E-04 | CDKN1A, MAPK8, CDKN2A, CDK2, MAPK14, TP53 | 0.003713 |
| BP | GO:0043627~response to estrogen | 6 | 3.157895 | 2.02E-04 | F7, HSP90AA1, CAV1, MMP2, PPARG, ESR1 | 0.003221 |
| BP | GO:0018107~peptidyl-threonine phosphorylation | 6 | 3.157895 | 2.02E-04 | GSK3B, MAPK8, CHEK1, MAPK1, AKT1, PRKCA | 0.003221 |
| BP | GO:0097191~extrinsic apoptotic signaling pathway | 6 | 3.157895 | 1.69E-04 | GSK3B, TGFB1, CASP8, IFNG, BAX, TNF | 0.002796 |
| BP | GO:0051384~response to glucocorticoid | 6 | 3.157895 | 1.16E-04 | IL10, IL6, CASP3, BCL2, PTGS2, TNF | 0.002008 |
| BP | GO:0014823~response to activity | 6 | 3.157895 | 9.49E-05 | IL10, IL6, MMP2, HSF1, FOS, TNF | 0.001723 |
| BP | GO:0034097~response to cytokine | 6 | 3.157895 | 9.49E-05 | COL3A1, STAT1, BCL2, SELE, RELA, BCL2L1 | 0.001723 |
| BP | GO:0042311~vasodilation | 6 | 3.157895 | 6.88E-05 | NOS3, KCNMA1, DRD1, TNF, EGFR, ADRA2A | 0.001314 |
| BP | GO:0050796~regulation of insulin secretion | 6 | 3.157895 | 6.14E-05 | IL6, IFNG, NOS2, ALOX5, IL1B, TNF | 0.001183 |
| BP | GO:0046427~positive regulation of JAK-STAT cascade | 6 | 3.157895 | 5.47E-05 | IL10, IL6, CYP1B1, CALM3, F2, TNF | 0.001087 |
| MF | GO:0030331~estrogen receptor binding | 6 | 3.157895 | 5.33E-05 | NCOA1, PCNA, PARP1, CTNNB1, PPARG, ESR1 | 8.14E-04 |
| BP | GO:0001541~ovarian follicle development | 6 | 3.157895 | 3.33E-05 | MMP2, BCL2, KDR, BAX, BCL2L1, SOD1 | 7.06E-04 |
| BP | GO:0051591~response to cAMP | 6 | 3.157895 | 2.92E-05 | THBD, STAT1, FOS, RELA, SLC6A3, DUOX2 | 6.39E-04 |
| BP | GO:2001240~negative regulation of extrinsic apoptotic signaling pathway in absence of ligand | 6 | 3.157895 | 2.92E-05 | IL1A, IL1B, BCL2, AKT1, TNF, BCL2L1 | 6.39E-04 |
| BP | GO:2000352~negative regulation of endothelial cell apoptotic process | 6 | 3.157895 | 2.92E-05 | IL10, IL4, SERPINE1, KDR, ICAM1, NFE2L2 | 6.39E-04 |
| BP | GO:0097193~intrinsic apoptotic signaling pathway | 6 | 3.157895 | 2.92E-05 | CASP9, CDKN1A, CASP3, BAX, CYCS, TP53 | 6.39E-04 |
| BP | GO:0045765~regulation of angiogenesis | 6 | 3.157895 | 2.54E-05 | EGLN1, IL6, ERBB2, HMOX1, CTNNB1, PIK3CG | 5.87E-04 |
| MF | GO:0097718~disordered domain specific binding | 6 | 3.157895 | 2.09E-05 | RB1, HSP90AA1, CDKN2A, MDM2, CTNNB1, TP53 | 3.70E-04 |
| BP | GO:0048662~negative regulation of smooth muscle cell proliferation | 6 | 3.157895 | 1.01E-05 | IFNG, NOS3, IGFBP3, HMOX1, APOD, PPARG | 2.78E-04 |
| BP | GO:0010875~positive regulation of cholesterol efflux | 6 | 3.157895 | 5.85E-06 | NFKBIA, RXRA, CAV1, PON1, PPARG, SIRT1 | 1.81E-04 |
| BP | GO:0051247~positive regulation of protein metabolic process | 6 | 3.157895 | 4.81E-06 | NFKBIA, APP, MAPK8, TGFB1, CHRNA7, AKT1 | 1.52E-04 |
| BP | GO:0006809~nitric oxide biosynthetic process | 6 | 3.157895 | 1.99E-06 | NQO1, NOS2, NOS3, CAV1, CYP1B1, AKT1 | 7.59E-05 |
| BP | GO:0043401~steroid hormone mediated signaling pathway | 6 | 3.157895 | 1.19E-06 | RXRA, PDE3A, MAPK1, PPARA, ESR1, PPARD | 4.76E-05 |
| BP | GO:0071880~adenylate cyclase-activating adrenergic receptor signaling pathway | 6 | 3.157895 | 6.67E-07 | ADRB1, DRD1, ADRB2, ADRA1B, ADRA2C, ADRA2A | 2.90E-05 |
| BP | GO:0090399~replicative senescence | 6 | 3.157895 | 3.46E-07 | CDKN1A, CDKN2A, CHEK2, CHEK1, SERPINE1, TP53 | 1.55E-05 |
| MF | GO:0034056~estrogen response element binding | 6 | 3.157895 | 4.19E-08 | AR, PGR, NR3C1, ESR1, ESR2, NR3C2 | 2.15E-06 |
| BP | GO:0098869~cellular oxidant detoxification | 5 | 2.631579 | 0.006657 | ALB, PTGS2, DUOX2, PTGES, PTGS1 | 0.04885 |
| BP | GO:0060079~excitatory postsynaptic potential | 5 | 2.631579 | 0.004978 | GSK3B, PPP3CA, CHRNA2, CHRNA7, AKT1 | 0.037832 |
| BP | GO:0070098~chemokine-mediated signaling pathway | 5 | 2.631579 | 0.004978 | CXCL10, CXCL11, CXCL8, CCL2, CXCL2 | 0.037832 |
| MF | GO:0004197~cysteine-type endopeptidase activity | 5 | 2.631579 | 0.00456 | CASP9, CASP7, CASP8, CASP3, CTSD | 0.036808 |
| BP | GO:0043124~negative regulation of I-kappaB kinase/NF-kappaB signaling | 5 | 2.631579 | 0.004257 | NFKBIA, CASP8, STAT1, ESR1, SIRT1 | 0.03365 |
| BP | GO:0007346~regulation of mitotic cell cycle | 5 | 2.631579 | 0.004257 | RB1, CDKN1A, CDK2, BIRC5, SIRT1 | 0.03365 |
| BP | GO:0006879~cellular iron ion homeostasis | 5 | 2.631579 | 0.003818 | EGLN1, MYC, HMOX1, HIF1A, SOD1 | 0.031039 |
| CC | GO:0031093~platelet alpha granule lumen | 5 | 2.631579 | 0.003586 | APP, TGFB1, ALB, SERPINE1, IGF2 | 0.024925 |
| BP | GO:0001570~vasculogenesis | 5 | 2.631579 | 0.00341 | TGFB1, RASA1, CAV1, KDR, HAS2 | 0.02805 |
| BP | GO:0071230~cellular response to amino acid stimulus | 5 | 2.631579 | 0.00341 | COL3A1, MMP2, TNF, EGFR, BCL2L1 | 0.02805 |
| BP | GO:0010507~negative regulation of autophagy | 5 | 2.631579 | 0.003033 | IL10, BCL2, AKT1, MET, BCL2L1 | 0.026333 |
| BP | GO:0042102~positive regulation of T cell proliferation | 5 | 2.631579 | 0.003033 | IL4, IL6, VCAM1, CD40LG, IL1B | 0.026333 |
| BP | GO:0042110~T cell activation | 5 | 2.631579 | 0.002686 | DPP4, IL4, PPP3CA, CASP8, PIK3CG | 0.023803 |
| BP | GO:0042475~odontogenesis of dentin-containing tooth | 5 | 2.631579 | 0.002686 | TGFB1, BAX, CTNNB1, SCN5A, RUNX2 | 0.023803 |
| BP | GO:2000134~negative regulation of G1/S transition of mitotic cell cycle | 5 | 2.631579 | 0.002523 | RB1, CDKN1A, PTEN, BCL2, CCL2 | 0.023111 |
| BP | GO:0071363~cellular response to growth factor stimulus | 5 | 2.631579 | 0.002523 | OPRD1, INSR, ERBB2, MDM2, CTNNB1 | 0.023111 |
| BP | GO:0045599~negative regulation of fat cell differentiation | 5 | 2.631579 | 0.002367 | IL6, TGFB1, E2F1, SIRT1, TNF | 0.022203 |
| BP | GO:0050890~cognition | 5 | 2.631579 | 0.002075 | APP, CHRM1, CHRNA7, TNF, SLC6A3 | 0.019608 |
| BP | GO:0045786~negative regulation of cell cycle | 5 | 2.631579 | 0.002075 | RB1, TGFB1, CASP3, PTGS2, SIRT1 | 0.019608 |
| BP | GO:0033674~positive regulation of kinase activity | 5 | 2.631579 | 0.002075 | INSR, ERBB2, KDR, MET, EGFR | 0.019608 |
| BP | GO:0009612~response to mechanical stimulus | 5 | 2.631579 | 0.002075 | MAPK8, STAT1, MMP2, PPARG, MPO | 0.019608 |
| BP | GO:0042149~cellular response to glucose starvation | 5 | 2.631579 | 0.001809 | HSPA5, BCL2, SIRT1, TP53, NFE2L2 | 0.017777 |
| BP | GO:0051592~response to calcium ion | 5 | 2.631579 | 0.001809 | PPP3CA, CAV1, KCNMA1, CALM3, EGFR | 0.017777 |
| MF | GO:0008009~chemokine activity | 5 | 2.631579 | 0.001683 | CXCL10, CXCL11, CXCL8, CCL2, CXCL2 | 0.016979 |
| BP | GO:0072089~stem cell proliferation | 5 | 2.631579 | 0.001349 | TGFB1, KDR, CTNNB1, TP53, RUNX2 | 0.013989 |
| BP | GO:0048538~thymus development | 5 | 2.631579 | 0.001249 | BCL2, MAPK1, CTNNB1, RAF1, SOD1 | 0.013057 |
| BP | GO:0008202~steroid metabolic process | 5 | 2.631579 | 0.001249 | CYP2B6, NR1I2, CYP1A1, CYP1B1, CYP3A4 | 0.013057 |
| BP | GO:0043280~positive regulation of cysteine-type endopeptidase activity involved in apoptotic process | 5 | 2.631579 | 9.78E-04 | MYC, CYCS, SIRT1, TNF, CTSD | 0.010614 |
| BP | GO:0030316~osteoclast differentiation | 5 | 2.631579 | 9.78E-04 | TGFB1, CTNNB1, FOS, MAPK14, TNF | 0.010614 |
| BP | GO:0032735~positive regulation of interleukin-12 production | 5 | 2.631579 | 8.98E-04 | CD40LG, IFNG, IRF1, MAPK14, RELA | 0.010043 |
| BP | GO:0046627~negative regulation of insulin receptor signaling pathway | 5 | 2.631579 | 8.23E-04 | GSK3B, PTPN1, PRKCB, IL1B, RELA | 0.009281 |
| MF | GO:0004714~transmembrane receptor protein tyrosine kinase activity | 5 | 2.631579 | 8.08E-04 | INSR, ERBB2, KDR, MET, EGFR | 0.008955 |
| BP | GO:2000648~positive regulation of stem cell proliferation | 5 | 2.631579 | 7.52E-04 | GJA1, TGFB1, KDR, CTNNB1, RUNX2 | 0.008634 |
| BP | GO:0042307~positive regulation of protein import into nucleus | 5 | 2.631579 | 7.52E-04 | HSP90AA1, TGFB1, IFNG, MAPK14, PTGS2 | 0.008634 |
| BP | GO:0051604~protein maturation | 5 | 2.631579 | 7.52E-04 | CASP9, IKBKB, CASP7, CASP8, CASP3 | 0.008634 |
| BP | GO:0043154~negative regulation of cysteine-type endopeptidase activity involved in apoptotic process | 5 | 2.631579 | 7.52E-04 | XIAP, BIRC5, AKT1, RAF1, TNF | 0.008634 |
| CC | GO:0000307~cyclin-dependent protein kinase holoenzyme complex | 5 | 2.631579 | 6.50E-04 | CCNA2, CDKN1A, PCNA, CCND1, CDK2 | 0.00624 |
| BP | GO:0008625~extrinsic apoptotic signaling pathway via death domain receptors | 5 | 2.631579 | 6.23E-04 | CASP8, BCL2, BAX, RAF1, TNF | 0.007397 |
| BP | GO:0071479~cellular response to ionizing radiation | 5 | 2.631579 | 6.23E-04 | CDKN1A, TGFB1, MAPK14, SIRT1, TP53 | 0.007397 |
| BP | GO:1900182~positive regulation of protein localization to nucleus | 5 | 2.631579 | 6.23E-04 | TGFB1, PARP1, CDKN2A, AKT1, F2 | 0.007397 |
| BP | GO:0009408~response to heat | 5 | 2.631579 | 6.23E-04 | HSP90AA1, NOS3, HSPB1, AKT1, SOD1 | 0.007397 |
| BP | GO:0048144~fibroblast proliferation | 5 | 2.631579 | 6.23E-04 | COL3A1, CDKN1A, CAV1, ESR1, TP53 | 0.007397 |
| BP | GO:0001937~negative regulation of endothelial cell proliferation | 5 | 2.631579 | 5.65E-04 | STAT1, ALOX5, CAV1, TNF, XDH | 0.00704 |
| CC | GO:0032993~protein-DNA complex | 5 | 2.631579 | 4.92E-04 | PARP1, CTNNB1, FOS, TOP1, NFE2L2 | 0.004869 |
| BP | GO:0070555~response to interleukin-1 | 5 | 2.631579 | 4.61E-04 | APP, IL1B, PRKCA, SELE, RELA | 0.0061 |
| BP | GO:0031333~negative regulation of protein complex assembly | 5 | 2.631579 | 4.61E-04 | OPRD1, GSK3B, HSPA5, HSF1, RAF1 | 0.0061 |
| BP | GO:0045931~positive regulation of mitotic cell cycle | 5 | 2.631579 | 3.71E-04 | APP, HSF1, MDM2, BIRC5, PRKCA | 0.005123 |
| BP | GO:0032570~response to progesterone | 5 | 2.631579 | 3.31E-04 | NCOA1, TGFB1, CAV1, FOS, RELA | 0.004634 |
| BP | GO:0030522~intracellular receptor signaling pathway | 5 | 2.631579 | 3.31E-04 | AR, NR1I2, AHR, PPARA, PPARD | 0.004634 |
| BP | GO:0051881~regulation of mitochondrial membrane potential | 5 | 2.631579 | 3.31E-04 | OPRD1, BCL2, BAX, BCL2L1, SOD1 | 0.004634 |
| BP | GO:0030225~macrophage differentiation | 5 | 2.631579 | 2.94E-04 | CASP8, IFNG, PARP1, SIRT1, MMP9 | 0.004309 |
| BP | GO:0035924~cellular response to vascular endothelial growth factor stimulus | 5 | 2.631579 | 2.94E-04 | VCAM1, KDR, HSPB1, AKT1, MAPK14 | 0.004309 |
| BP | GO:0007159~leukocyte cell-cell adhesion | 5 | 2.631579 | 2.61E-04 | VCAM1, CD40LG, OLR1, SELE, ICAM1 | 0.003951 |
| BP | GO:0016239~positive regulation of macroautophagy | 5 | 2.631579 | 2.61E-04 | IL4, KDR, HMOX1, HIF1A, SIRT1 | 0.003951 |
| BP | GO:0048009~insulin-like growth factor receptor signaling pathway | 5 | 2.631579 | 2.61E-04 | AR, IGF2, MAPK1, AKT1, RAF1 | 0.003951 |
| BP | GO:0034612~response to tumor necrosis factor | 5 | 2.631579 | 2.30E-04 | CASP8, CASP3, CD14, PTGS2, SELE | 0.003612 |
| BP | GO:0051281~positive regulation of release of sequestered calcium ion into cytosol | 5 | 2.631579 | 2.30E-04 | CXCL10, CXCL11, BAX, DRD1, F2 | 0.003612 |
| BP | GO:0045840~positive regulation of mitotic nuclear division | 5 | 2.631579 | 2.02E-04 | IL1A, IL1B, INSR, IGF2, TNF | 0.003221 |
| MF | GO:0051721~protein phosphatase 2A binding | 5 | 2.631579 | 1.90E-04 | PTPN1, STAT1, BCL2, TP53, SLC6A3 | 0.002616 |
| CC | GO:0043209~myelin sheath | 5 | 2.631579 | 1.20E-04 | HSP90AA1, CA2, ERBB2, BCL2, CALM3 | 0.001654 |
| BP | GO:0042554~superoxide anion generation | 5 | 2.631579 | 9.68E-05 | NCF1, NOX5, ALOX12, DUOX2, SOD1 | 0.001745 |
| BP | GO:2000378~negative regulation of reactive oxygen species metabolic process | 5 | 2.631579 | 8.19E-05 | MMP3, BCL2, HIF1A, TP53, HK2 | 0.00152 |
| BP | GO:0045821~positive regulation of glycolytic process | 5 | 2.631579 | 5.73E-05 | APP, IFNG, INSR, HTR2A, HIF1A | 0.001119 |
| BP | GO:1902004~positive regulation of beta-amyloid formation | 5 | 2.631579 | 5.73E-05 | IFNG, CHRNA7, CASP3, TNF, RELA | 0.001119 |
| BP | GO:1904019~epithelial cell apoptotic process | 5 | 2.631579 | 4.72E-05 | CASP9, CASP3, BCL2, BAX, HMOX1 | 9.67E-04 |
| BP | GO:0033280~response to vitamin D | 5 | 2.631579 | 3.10E-05 | CXCL10, TGFB1, PRKCB, SPP1, PTGS2 | 6.68E-04 |
| BP | GO:0035994~response to muscle stretch | 5 | 2.631579 | 3.10E-05 | NFKBIA, FOS, RAF1, MAPK14, RELA | 6.68E-04 |
| BP | GO:0008637~apoptotic mitochondrial changes | 5 | 2.631579 | 2.47E-05 | CDKN2A, BAX, AKT1, HK2, BCL2L1 | 5.75E-04 |
| BP | GO:0035234~ectopic germ cell programmed cell death | 5 | 2.631579 | 2.47E-05 | IL1A, IL1B, BAX, BCL2L1, SOD1 | 5.75E-04 |
| BP | GO:0048143~astrocyte activation | 5 | 2.631579 | 2.47E-05 | APP, IFNG, IL1B, TNF, EGFR | 5.75E-04 |
| BP | GO:0051918~negative regulation of fibrinolysis | 5 | 2.631579 | 6.00E-06 | THBD, PLAU, SERPINE1, PLAT, F2 | 1.81E-04 |
| BP | GO:0060333~interferon-gamma-mediated signaling pathway | 5 | 2.631579 | 6.00E-06 | IFNG, STAT1, IRF1, RAF1, TP53 | 1.81E-04 |
| BP | GO:0030518~intracellular steroid hormone receptor signaling pathway | 5 | 2.631579 | 4.18E-06 | AR, PGR, NR3C1, ESR1, NR3C2 | 1.37E-04 |
| BP | GO:0010888~negative regulation of lipid storage | 5 | 2.631579 | 1.80E-06 | NFKBIA, CRP, IL6, PPARG, TNF | 7.00E-05 |
| BP | GO:0000077~DNA damage checkpoint | 4 | 2.105263 | 0.006561 | CHEK2, CHEK1, E2F1, MAPK14 | 0.048281 |
| BP | GO:0046326~positive regulation of glucose import | 4 | 2.105263 | 0.005658 | INSR, AKT1, MAPK14, NFE2L2 | 0.042495 |
| BP | GO:0031100~animal organ regeneration | 4 | 2.105263 | 0.005658 | CCNA2, F7, CDKN1A, PPARG | 0.042495 |
| BP | GO:0048147~negative regulation of fibroblast proliferation | 4 | 2.105263 | 0.005658 | MYC, CAV1, BAX, TP53 | 0.042495 |
| BP | GO:0003180~aortic valve morphogenesis | 4 | 2.105263 | 0.005237 | RB1, TGFB1, NOS3, NFATC1 | 0.039679 |
| BP | GO:0050873~brown fat cell differentiation | 4 | 2.105263 | 0.004835 | ADRB1, SLC2A4, ADRB2, PTGS2 | 0.037636 |
| BP | GO:0043687~post-translational protein modification | 4 | 2.105263 | 0.004835 | CCNA2, PRKCB, CDK2, PRKCA | 0.037636 |
| MF | GO:0051059~NF-kappaB binding | 4 | 2.105263 | 0.004701 | NFKBIA, GSK3B, CDKN2A, RELA | 0.037407 |
| BP | GO:0060078~regulation of postsynaptic membrane potential | 4 | 2.105263 | 0.004452 | GABRA1, GRIA2, CHRM1, ADRB1 | 0.034977 |
| BP | GO:0071407~cellular response to organic cyclic compound | 4 | 2.105263 | 0.004452 | CASP8, STAT1, CYP1A1, CYP1B1 | 0.034977 |
| BP | GO:0042771~intrinsic apoptotic signaling pathway in response to DNA damage by p53 class mediator | 4 | 2.105263 | 0.004089 | CDKN1A, CHEK2, SIRT1, TP53 | 0.032566 |
| BP | GO:0051968~positive regulation of synaptic transmission, glutamatergic | 4 | 2.105263 | 0.004089 | CCL2, DRD1, PTGS2, EGFR | 0.032566 |
| MF | GO:0005164~tumor necrosis factor receptor binding | 4 | 2.105263 | 0.003954 | CASP8, CD40LG, STAT1, TNF | 0.034367 |
| BP | GO:0070050~neuron cellular homeostasis | 4 | 2.105263 | 0.003744 | APP, IL6, CA2, SLC6A2 | 0.030534 |
| BP | GO:2001234~negative regulation of apoptotic signaling pathway | 4 | 2.105263 | 0.003744 | RB1, BCL2, BAX, TNF | 0.030534 |
| MF | GO:0070330~aromatase activity | 4 | 2.105263 | 0.003609 | CYP2B6, CYP1A1, CYP1B1, CYP3A4 | 0.031864 |
| BP | GO:0051899~membrane depolarization | 4 | 2.105263 | 0.003417 | CHRNA2, CAV1, SCN5A, SLC6A4 | 0.02805 |
| BP | GO:0045740~positive regulation of DNA replication | 4 | 2.105263 | 0.003417 | CDKN1A, PCNA, CDK2, EGFR | 0.02805 |
| BP | GO:0045907~positive regulation of vasoconstriction | 4 | 2.105263 | 0.003417 | CAV1, HTR2A, PTGS2, EGFR | 0.02805 |
| MF | GO:0004601~peroxidase activity | 4 | 2.105263 | 0.003284 | PTGS2, MPO, DUOX2, PTGS1 | 0.029452 |
| BP | GO:0034142~toll-like receptor 4 signaling pathway | 4 | 2.105263 | 0.003109 | NFKBIA, CHUK, CD14, RELA | 0.026854 |
| BP | GO:0001774~microglial cell activation | 4 | 2.105263 | 0.002818 | IL4, APP, IFNG, TNF | 0.024716 |
| BP | GO:0045672~positive regulation of osteoclast differentiation | 4 | 2.105263 | 0.002818 | PPP3CA, IFNG, FOS, TNF | 0.024716 |
| BP | GO:0045662~negative regulation of myoblast differentiation | 4 | 2.105263 | 0.002545 | CXCL10, TGFB1, TNF, PPARD | 0.023111 |
| BP | GO:0071549~cellular response to dexamethasone stimulus | 4 | 2.105263 | 0.002545 | CASP9, TGFB1, NR3C1, EGFR | 0.023111 |
| BP | GO:0038061~NIK/NF-kappaB signaling | 4 | 2.105263 | 0.002545 | NFKBIA, CHUK, AKT1, RELA | 0.023111 |
| BP | GO:0048565~digestive tract development | 4 | 2.105263 | 0.002289 | RB1, COL3A1, TGFB1, CYP1A1 | 0.021547 |
| BP | GO:0035902~response to immobilization stress | 4 | 2.105263 | 0.002049 | TGFB1, CYP1A1, PPARG, FOS | 0.019608 |
| BP | GO:0046716~muscle cell cellular homeostasis | 4 | 2.105263 | 0.002049 | TGFB1, CAV1, HIF1A, SOD1 | 0.019608 |
| BP | GO:2001243~negative regulation of intrinsic apoptotic signaling pathway | 4 | 2.105263 | 0.002049 | BCL2, AKT1, MMP9, BCL2L1 | 0.019608 |
| MF | GO:0070412~R-SMAD binding | 4 | 2.105263 | 0.00193 | JUN, PARP1, PPARG, FOS | 0.018481 |
| MF | GO:0017025~TBP-class protein binding | 4 | 2.105263 | 0.00193 | AHR, NR3C1, ESR1, NR3C2 | 0.018481 |
| BP | GO:0002053~positive regulation of mesenchymal cell proliferation | 4 | 2.105263 | 0.001826 | STAT1, MYC, KDR, CTNNB1 | 0.017777 |
| BP | GO:0046697~decidualization | 4 | 2.105263 | 0.001826 | PARP1, SPP1, PTGS2, PPARD | 0.017777 |
| BP | GO:0045861~negative regulation of proteolysis | 4 | 2.105263 | 0.001826 | AKT1, PLAT, F2, TP53 | 0.017777 |
| BP | GO:0001836~release of cytochrome c from mitochondria | 4 | 2.105263 | 0.001426 | BCL2, BAX, TP53, BCL2L1 | 0.014323 |
| BP | GO:0097194~execution phase of apoptosis | 4 | 2.105263 | 0.001426 | CASP7, CASP8, CASP3, AKT1 | 0.014323 |
| BP | GO:0042178~xenobiotic catabolic process | 4 | 2.105263 | 0.001426 | GSTM1, CYP2B6, NR1I2, CYP3A4 | 0.014323 |
| BP | GO:0043200~response to amino acid | 4 | 2.105263 | 0.001426 | CHUK, CASP3, BCL2, RELA | 0.014323 |
| BP | GO:0070498~interleukin-1-mediated signaling pathway | 4 | 2.105263 | 0.001426 | NFKBIA, IKBKB, IL1B, RELA | 0.014323 |
| BP | GO:0006977~DNA damage response, signal transduction by p53 class mediator resulting in cell cycle arrest | 4 | 2.105263 | 0.001249 | CDKN1A, CHEK2, MDM2, TP53 | 0.013057 |
| BP | GO:0045088~regulation of innate immune response | 4 | 2.105263 | 0.001087 | CASP8, IRF1, XIAP, NFE2L2 | 0.011594 |
| BP | GO:0071391~cellular response to estrogen stimulus | 4 | 2.105263 | 0.001087 | AR, MDM2, ESR1, ESR2 | 0.011594 |
| BP | GO:0060252~positive regulation of glial cell proliferation | 4 | 2.105263 | 0.001087 | IL6, IL1B, TNF, EGFR | 0.011594 |
| BP | GO:0019538~protein metabolic process | 4 | 2.105263 | 0.001087 | MMP1, MMP2, LTA4H, ACACA | 0.011594 |
| BP | GO:1902176~negative regulation of oxidative stress-induced intrinsic apoptotic signaling pathway | 4 | 2.105263 | 8.03E-04 | HSPB1, AKT1, SIRT1, NFE2L2 | 0.009101 |
| BP | GO:0042730~fibrinolysis | 4 | 2.105263 | 8.03E-04 | PLAU, SERPINE1, PLAT, F2 | 0.009101 |
| BP | GO:0051403~stress-activated MAPK cascade | 4 | 2.105263 | 8.03E-04 | IKBKB, MAPK8, MAPK1, MAPK14 | 0.009101 |
| BP | GO:0010165~response to X-ray | 4 | 2.105263 | 6.81E-04 | THBD, CDKN1A, CASP3, TP53 | 0.007966 |
| BP | GO:0032930~positive regulation of superoxide anion generation | 4 | 2.105263 | 6.81E-04 | CRP, TGFB1, EGFR, SOD1 | 0.007966 |
| BP | GO:0051044~positive regulation of membrane protein ectodomain proteolysis | 4 | 2.105263 | 5.72E-04 | IFNG, IL1B, TNF, ADRA2A | 0.00704 |
| BP | GO:0042770~signal transduction in response to DNA damage | 4 | 2.105263 | 5.72E-04 | CASP9, CHEK2, CHEK1, MAPK14 | 0.00704 |
| BP | GO:0030194~positive regulation of blood coagulation | 4 | 2.105263 | 5.72E-04 | F7, SERPINE1, F2, NFE2L2 | 0.00704 |
| BP | GO:0044849~estrous cycle | 4 | 2.105263 | 4.74E-04 | NCOA1, PCNA, CYP1B1, HAS2 | 0.0061 |
| BP | GO:2000573~positive regulation of DNA biosynthetic process | 4 | 2.105263 | 4.74E-04 | CCNA2, CYP1B1, NFATC1, TNF | 0.0061 |
| BP | GO:0051770~positive regulation of nitric-oxide synthase biosynthetic process | 4 | 2.105263 | 4.74E-04 | IFNG, STAT1, KDR, CCL2 | 0.0061 |
| BP | GO:0006940~regulation of smooth muscle contraction | 4 | 2.105263 | 4.74E-04 | CHRM2, CAV1, ADRB2, ADRA2C | 0.0061 |
| BP | GO:1904645~response to beta-amyloid | 4 | 2.105263 | 4.74E-04 | CHRNA7, MMP2, MMP3, MMP9 | 0.0061 |
| BP | GO:0045651~positive regulation of macrophage differentiation | 4 | 2.105263 | 4.74E-04 | RB1, CASP8, HSF1, PRKCA | 0.0061 |
| MF | GO:0045236~CXCR chemokine receptor binding | 4 | 2.105263 | 4.11E-04 | CXCL10, CXCL11, CXCL8, CXCL2 | 0.00516 |
| BP | GO:0019430~removal of superoxide radicals | 4 | 2.105263 | 3.88E-04 | NQO1, NOS3, MPO, SOD1 | 0.00526 |
| BP | GO:0010039~response to iron ion | 4 | 2.105263 | 3.88E-04 | MDM2, BCL2, HIF1A, SLC6A3 | 0.00526 |
| BP | GO:0071498~cellular response to fluid shear stress | 4 | 2.105263 | 3.13E-04 | MMP2, HAS2, PTGS2, NFE2L2 | 0.00445 |
| BP | GO:0045348~positive regulation of MHC class II biosynthetic process | 4 | 2.105263 | 3.13E-04 | IL10, IL4, IFNG, SIRT1 | 0.00445 |
| BP | GO:0051173~positive regulation of nitrogen compound metabolic process | 4 | 2.105263 | 3.13E-04 | IKBKB, IFNG, RAF1, TNF | 0.00445 |
| BP | GO:0010745~negative regulation of macrophage derived foam cell differentiation | 4 | 2.105263 | 3.13E-04 | NFKBIA, CRP, PPARG, PPARA | 0.00445 |
| MF | GO:0016702~oxidoreductase activity, acting on single donors with incorporation of molecular oxygen, incorporation of two atoms of oxygen | 4 | 2.105263 | 2.03E-04 | ALOX5, ALOX12, PTGS2, PTGS1 | 0.002733 |
| BP | GO:0044346~fibroblast apoptotic process | 4 | 2.105263 | 1.45E-04 | CASP9, CASP7, MYC, CASP3 | 0.002441 |
| BP | GO:0071492~cellular response to UV-A | 4 | 2.105263 | 1.45E-04 | MMP1, MMP2, MMP3, MMP9 | 0.002441 |
| BP | GO:0050665~hydrogen peroxide biosynthetic process | 4 | 2.105263 | 1.45E-04 | MAOB, CYP1A1, DUOX2, SOD1 | 0.002441 |
| BP | GO:0071394~cellular response to testosterone stimulus | 4 | 2.105263 | 1.06E-04 | AR, NCF1, SPP1, ELK1 | 0.001862 |
| BP | GO:0071223~cellular response to lipoteichoic acid | 4 | 2.105263 | 1.06E-04 | CD14, LBP, MAPK14, RELA | 0.001862 |
| BP | GO:0009651~response to salt stress | 4 | 2.105263 | 7.48E-05 | HSP90AA1, BAX, TNF, TP53 | 0.001419 |
| MF | GO:0097153~cysteine-type endopeptidase activity involved in apoptotic process | 4 | 2.105263 | 5.33E-05 | CASP9, CASP7, CASP8, CASP3 | 8.14E-04 |
| BP | GO:0001542~ovulation from ovarian follicle | 4 | 2.105263 | 5.03E-05 | NOS3, MMP2, PGR, SIRT1 | 0.001022 |
| BP | GO:0010573~vascular endothelial growth factor production | 4 | 2.105263 | 3.16E-05 | IL6, IL1B, HIF1A, TNF | 6.75E-04 |
| MF | GO:0030235~nitric-oxide synthase regulator activity | 4 | 2.105263 | 1.93E-05 | HSP90AA1, AKT1, ESR1, EGFR | 3.54E-04 |
| BP | GO:0031622~positive regulation of fever generation | 4 | 2.105263 | 9.17E-06 | IL1B, PTGER3, PTGS2, TNF | 2.58E-04 |
| MF | GO:0097371~MDM2/MDM4 family protein binding | 3 | 1.578947 | 0.006157 | CDKN2A, PPARA, TP53 | 0.04558 |
| MF | GO:0004861~cyclin-dependent protein serine/threonine kinase inhibitor activity | 3 | 1.578947 | 0.006157 | CDKN1A, CDKN2A, CASP3 | 0.04558 |
| BP | GO:0042420~dopamine catabolic process | 3 | 1.578947 | 0.005928 | MAOB, MAOA, SLC6A3 | 0.044136 |
| BP | GO:0051926~negative regulation of calcium ion transport | 3 | 1.578947 | 0.005928 | NOS3, PTGS2, ADRA2A | 0.044136 |
| BP | GO:0050927~positive regulation of positive chemotaxis | 3 | 1.578947 | 0.005928 | F7, KDR, F3 | 0.044136 |
| MF | GO:0050998~nitric-oxide synthase binding | 3 | 1.578947 | 0.005165 | CAV1, SCN5A, SLC6A4 | 0.039434 |
| BP | GO:0006983~ER overload response | 3 | 1.578947 | 0.004972 | GSK3B, HSPA5, TP53 | 0.037832 |
| BP | GO:1900015~regulation of cytokine production involved in inflammatory response | 3 | 1.578947 | 0.004972 | NOS2, ALOX5, MAPK14 | 0.037832 |
| BP | GO:0070242~thymocyte apoptotic process | 3 | 1.578947 | 0.004972 | CHEK2, BAX, TP53 | 0.037832 |
| BP | GO:0006950~response to stress | 3 | 1.578947 | 0.004972 | BCL2, BAX, BCL2L1 | 0.037832 |
| BP | GO:0010887~negative regulation of cholesterol storage | 3 | 1.578947 | 0.004972 | PPARG, PPARA, PPARD | 0.037832 |
| BP | GO:0032308~positive regulation of prostaglandin secretion | 3 | 1.578947 | 0.004972 | IL1A, IL1B, PTGES | 0.037832 |
| MF | GO:0016175~superoxide-generating NADPH oxidase activity | 3 | 1.578947 | 0.004254 | NCF1, NOX5, DUOX2 | 0.034831 |
| MF | GO:0008504~monoamine transmembrane transporter activity | 3 | 1.578947 | 0.004254 | SLC6A2, SLC6A3, SLC6A4 | 0.034831 |
| BP | GO:0051093~negative regulation of developmental process | 3 | 1.578947 | 0.004095 | GSK3B, BCL2L1, SOD1 | 0.032566 |
| BP | GO:0070431~nucleotide-binding oligomerization domain containing 2 signaling pathway | 3 | 1.578947 | 0.004095 | NFKBIA, XIAP, RELA | 0.032566 |
| BP | GO:2000273~positive regulation of receptor activity | 3 | 1.578947 | 0.004095 | IL10, IFNG, HIF1A | 0.032566 |
| BP | GO:1904179~positive regulation of adipose tissue development | 3 | 1.578947 | 0.004095 | NCOA1, PPARG, SIRT1 | 0.032566 |
| BP | GO:0071875~adrenergic receptor signaling pathway | 3 | 1.578947 | 0.004095 | ADRB2, ADRA2C, ADRA2A | 0.032566 |
| CC | GO:0005641~nuclear envelope lumen | 3 | 1.578947 | 0.003617 | APP, ALOX5, PTGES | 0.024925 |
| BP | GO:1903140~regulation of establishment of endothelial barrier | 3 | 1.578947 | 0.003297 | IKBKB, IL1B, TNF | 0.027503 |
| BP | GO:0097237~cellular response to toxic substance | 3 | 1.578947 | 0.003297 | OPRD1, CYP1B1, TNF | 0.027503 |
| BP | GO:0051122~hepoxilin biosynthetic process | 3 | 1.578947 | 0.003297 | GSTM1, ALOX5, ALOX12 | 0.027503 |
| BP | GO:0035357~peroxisome proliferator activated receptor signaling pathway | 3 | 1.578947 | 0.003297 | NCOA1, RXRA, PPARG | 0.027503 |
| BP | GO:0071316~cellular response to nicotine | 3 | 1.578947 | 0.003297 | CHRNA2, TNF, RELA | 0.027503 |
| BP | GO:0015844~monoamine transport | 3 | 1.578947 | 0.003297 | SLC6A2, SLC6A3, SLC6A4 | 0.027503 |
| BP | GO:0038133~ERBB2-ERBB3 signaling pathway | 3 | 1.578947 | 0.003297 | ERBB2, MAPK1, RAF1 | 0.027503 |
| BP | GO:0002237~response to molecule of bacterial origin | 3 | 1.578947 | 0.003297 | IL10, CXCL8, CXCL2 | 0.027503 |
| BP | GO:0014909~smooth muscle cell migration | 3 | 1.578947 | 0.003297 | PLAU, BCL2, PLAT | 0.027503 |
| CC | GO:0097136~Bcl-2 family protein complex | 3 | 1.578947 | 0.002911 | BCL2, BAX, BCL2L1 | 0.022508 |
| MF | GO:0101020~estrogen 16-alpha-hydroxylase activity | 3 | 1.578947 | 0.002682 | CYP1A1, CYP1B1, CYP3A4 | 0.024439 |
| BP | GO:0010544~negative regulation of platelet activation | 3 | 1.578947 | 0.002581 | THBD, NOS3, F2 | 0.023111 |
| BP | GO:0007197~adenylate cyclase-inhibiting G-protein coupled acetylcholine receptor signaling pathway | 3 | 1.578947 | 0.002581 | CHRM2, CHRM1, OPRM1 | 0.023111 |
| BP | GO:0010742~macrophage derived foam cell differentiation | 3 | 1.578947 | 0.002581 | TGFB1, STAT1, PPARG | 0.023111 |
| BP | GO:2000272~negative regulation of receptor activity | 3 | 1.578947 | 0.002581 | PPARG, PPARA, TNF | 0.023111 |
| BP | GO:0061044~negative regulation of vascular wound healing | 3 | 1.578947 | 0.002581 | ALOX5, SERPINE1, TNF | 0.023111 |
| BP | GO:0007207~phospholipase C-activating G-protein coupled acetylcholine receptor signaling pathway | 3 | 1.578947 | 0.002581 | CHRM2, CHRM1, PRKCB | 0.023111 |
| BP | GO:0010757~negative regulation of plasminogen activation | 3 | 1.578947 | 0.002581 | PLAU, SERPINE1, PLAT | 0.023111 |
| MF | GO:0008131~primary amine oxidase activity | 3 | 1.578947 | 0.002025 | VCAM1, MAOB, MAOA | 0.019066 |
| BP | GO:2001233~regulation of apoptotic signaling pathway | 3 | 1.578947 | 0.001948 | BCL2, BAX, BCL2L1 | 0.0189 |
| MF | GO:0097199~cysteine-type endopeptidase activity involved in apoptotic signaling pathway | 3 | 1.578947 | 0.001456 | CASP9, CASP8, CASP3 | 0.015232 |
| MF | GO:0051434~BH3 domain binding | 3 | 1.578947 | 0.001456 | BCL2, BAX, BCL2L1 | 0.015232 |
| BP | GO:0008635~activation of cysteine-type endopeptidase activity involved in apoptotic process by cytochrome c | 3 | 1.578947 | 0.001401 | CASP9, BAX, CYCS | 0.014323 |
| BP | GO:0038127~ERBB signaling pathway | 3 | 1.578947 | 0.001401 | ERBB2, MAPK1, EGFR | 0.014323 |
| BP | GO:0032025~response to cobalt ion | 3 | 1.578947 | 0.001401 | CASP9, CASP8, CASP3 | 0.014323 |
| MF | GO:0101021~estrogen 2-hydroxylase activity | 3 | 1.578947 | 9.77E-04 | CYP2B6, CYP1A1, CYP3A4 | 0.010615 |
| BP | GO:0045923~positive regulation of fatty acid metabolic process | 3 | 1.578947 | 9.40E-04 | PPARG, PPARA, PPARD | 0.010286 |
| BP | GO:0010829~negative regulation of glucose transport | 3 | 1.578947 | 9.40E-04 | FABP5, PRKCB, IL1B | 0.010286 |
| BP | GO:0002248~connective tissue replacement involved in inflammatory response wound healing | 3 | 1.578947 | 9.40E-04 | IL1A, TGFB1, HIF1A | 0.010286 |
| BP | GO:0071677~positive regulation of mononuclear cell migration | 3 | 1.578947 | 9.40E-04 | IL4, TGFB1, TNF | 0.010286 |
| BP | GO:0050999~regulation of nitric-oxide synthase activity | 3 | 1.578947 | 9.40E-04 | IL1A, IL1B, EGFR | 0.010286 |
| CC | GO:0097180~serine protease inhibitor complex | 3 | 1.578947 | 8.29E-04 | PLAU, SERPINE1, PLAT | 0.007383 |
| MF | GO:0030284~estrogen receptor activity | 3 | 1.578947 | 5.90E-04 | PDE3A, ESR1, ESR2 | 0.007165 |
| BP | GO:0090400~stress-induced premature senescence | 3 | 1.578947 | 5.68E-04 | CDKN1A, MAPK14, SIRT1 | 0.00704 |
| BP | GO:1902512~positive regulation of apoptotic DNA fragmentation | 3 | 1.578947 | 5.68E-04 | IL6, HSF1, BAX | 0.00704 |
| BP | GO:0060559~positive regulation of calcidiol 1-monooxygenase activity | 3 | 1.578947 | 2.86E-04 | IFNG, IL1B, TNF | 0.004228 |
| BP | GO:0070141~response to UV-A | 3 | 1.578947 | 2.86E-04 | CCND1, AKT1, EGFR | 0.004228 |
| BP | GO:0034349~glial cell apoptotic process | 3 | 1.578947 | 2.86E-04 | RB1, CASP9, CASP3 | 0.004228 |
