# Supplementary material for: Elucidating the mechanism of Buyang Huanwu Decoction in the treatment of ischemic stroke: A network pharmacology and molecular docking study
Source: Medicine (Baltimore). 2026 Jul 17;105(29):e49736. doi: 10.1097/MD.0000000000049736 (PMC13384647; doi:10.1097/MD.0000000000049736)
Supplement: Supplementary file 10 [file medi-105-e49736-s010.docx]

**S 10.** Functional enrichment analysis of KEGG pathways using DAVID: Raw Data.

| **Term** | **Count** | **Gene ratio** | **PValue** | **Genes** | **FDR** |
| --- | --- | --- | --- | --- | --- |
| hsa05200:Pathways in cancer | 70 | 36.84211 | 6.87E-39 | RB1, GSK3B, CDKN1A, CXCL8, PTEN, ELK1, CASP9, IKBKB, CASP7, CASP8, CCND1, MYC, CASP3, AKT1, NCOA1, HSP90AA1, CHUK, PRKCB, MMP1, MMP2, PRKCA, FOS, F2, MMP9, CCNA2, AR, IFNG, BIRC5, PPARG, RAF1, MET, TP53, PPARD, PTGER3, XIAP, PTGS2, HIF1A, EGFR, RELA, MAPK8, RXRA, ERBB2, E2F1, HMOX1, MAPK1, EGLN1, NQO1, JUN, GSTM1, TGFB1, NOS2, CDKN2A, STAT1, IGF2, ESR1, ESR2, IL2, NFKBIA, IL4, IL6, GSTA2, CDK2, BCL2, MDM2, CYCS, BAX, CTNNB1, CALM3, NFE2L2, BCL2L1 | 7.00E-37 |
| hsa05417:Lipid and atherosclerosis | 49 | 25.78947 | 1.15E-37 | GSK3B, CXCL8, NCF1, TNF, CXCL2, ICAM1, CASP9, IKBKB, PPP3CA, CASP7, CASP8, CASP3, AKT1, OLR1, LBP, HSP90AA1, CHUK, MMP1, MMP3, PRKCA, FOS, MMP9, IL1B, PPARG, TP53, RELA, MAPK8, CYP2B6, RXRA, CCL2, MAPK1, CD14, JUN, VCAM1, HSPA5, NOS3, NFATC1, MAPK14, SELE, NFKBIA, IL6, CD40LG, CYP1A1, BCL2, CYCS, BAX, CALM3, NFE2L2, BCL2L1 | 5.86E-36 |
| hsa05163:Human cytomegalovirus infection | 37 | 19.47368 | 8.11E-23 | RB1, GSK3B, CDKN1A, CXCL8, PTGER3, PTGS2, ELK1, TNF, RELA, EGFR, CASP9, IKBKB, PPP3CA, CASP8, CCND1, MYC, CASP3, E2F1, CCL2, AKT1, MAPK1, CHUK, CDKN2A, PRKCB, PRKCA, NFATC1, MAPK14, NFKBIA, IL6, IL1B, MDM2, BAX, CTNNB1, CYCS, CALM3, RAF1, TP53 | 1.18E-21 |
| hsa05161:Hepatitis B | 36 | 18.94737 | 8.17E-27 | RB1, CDKN1A, PCNA, CXCL8, ELK1, TNF, RELA, CASP9, IKBKB, MAPK8, CASP8, MYC, CASP3, E2F1, AKT1, MAPK1, JUN, TGFB1, CHUK, PRKCB, STAT1, PRKCA, NFATC1, FOS, MAPK14, MMP9, NFKBIA, CCNA2, IL6, CDK2, BCL2, BAX, BIRC5, CYCS, RAF1, TP53 | 2.59E-25 |
| hsa05167:Kaposi sarcoma-associated herpesvirus infection | 35 | 18.42105 | 6.78E-23 | RB1, GSK3B, CDKN1A, CXCL8, PTGS2, HIF1A, CXCL2, RELA, PIK3CG, ICAM1, CASP9, IKBKB, PPP3CA, MAPK8, CASP8, CCND1, MYC, CASP3, E2F1, AKT1, MAPK1, JUN, CHUK, STAT1, NFATC1, FOS, MAPK14, NFKBIA, IL6, BAX, CTNNB1, CYCS, CALM3, RAF1, TP53 | 1.15E-21 |
| hsa05418:Fluid shear stress and atherosclerosis | 34 | 17.89474 | 1.02E-26 | NCF1, PLAT, TNF, RELA, ICAM1, IKBKB, THBD, MAPK8, KDR, CCL2, AKT1, HMOX1, NQO1, JUN, HSP90AA1, GSTM1, VCAM1, CHUK, NOS3, CAV1, MMP2, FOS, MAPK14, SELE, MMP9, IL1A, IFNG, IL1B, GSTA2, BCL2, CTNNB1, CALM3, TP53, NFE2L2 | 2.59E-25 |
| hsa04151:PI3K-Akt signaling pathway | 34 | 17.89474 | 1.53E-13 | CHRM2, GSK3B, CDKN1A, CHRM1, PTEN, RELA, EGFR, PIK3CG, CASP9, IKBKB, RXRA, CCND1, MYC, ERBB2, KDR, SPP1, AKT1, MAPK1, HSP90AA1, CHUK, NOS3, INSR, IGF2, PRKCA, IL2, IL4, IL6, CDK2, BCL2, MDM2, RAF1, MET, TP53, BCL2L1 | 4.32E-13 |
| hsa05207:Chemical carcinogenesis - receptor activation | 33 | 17.36842 | 1.57E-19 | RB1, CHRNA7, XIAP, ADRB1, AHR, ADRB2, CYP3A4, RELA, EGFR, CYP2B6, RXRA, CCND1, MYC, E2F1, CYP1B1, AKT1, MAPK1, JUN, HSP90AA1, GSTM1, PRKCB, PRKCA, FOS, ESR1, ESR2, AR, GSTA2, CYP1A1, BCL2, BIRC5, PGR, RAF1, PPARA | 1.46E-18 |
| hsa05022:Pathways of neurodegeneration - multiple diseases | 33 | 17.36842 | 1.45E-09 | MT-ND6, GRIA2, APP, GSK3B, CHRM1, CHRNA7, PTGS2, TNF, SLC6A3, RELA, CASP9, PPP3CA, CASP7, MAPK8, CASP8, CASP3, MAPK1, NOS2, HSPA5, PRKCB, PRKCA, MAPK14, SOD1, IL1A, IL6, IL1B, BCL2, BAX, CTNNB1, CYCS, CALM3, RAF1, BCL2L1 | 2.38E-09 |
| hsa05166:Human T-cell leukemia virus 1 infection | 32 | 16.84211 | 6.23E-18 | RB1, CDKN1A, PTEN, XIAP, ELK1, TNF, RELA, ICAM1, IKBKB, PPP3CA, MAPK8, CCND1, CHEK2, MYC, CHEK1, E2F1, AKT1, MAPK1, JUN, TGFB1, CHUK, CDKN2A, NFATC1, FOS, IL2, NFKBIA, CCNA2, IL6, CDK2, BAX, TP53, BCL2L1 | 4.23E-17 |
| hsa04010:MAPK signaling pathway | 31 | 16.31579 | 2.73E-13 | HSPB1, ELK1, TNF, RELA, EGFR, IKBKB, PPP3CA, MAPK8, MYC, CASP3, ERBB2, KDR, AKT1, MAPK1, CD14, JUN, TGFB1, CHUK, PRKCB, INSR, IGF2, PRKCA, NFATC1, FOS, MAPK14, IL1A, RASA1, IL1B, RAF1, MET, TP53 | 7.33E-13 |
| hsa04933:AGE-RAGE signaling pathway in diabetic complications | 30 | 15.78947 | 2.82E-26 | CXCL8, SERPINE1, TNF, RELA, ICAM1, THBD, MAPK8, CCND1, CASP3, CCL2, AKT1, MAPK1, JUN, TGFB1, VCAM1, PRKCB, NOS3, STAT1, MMP2, PRKCA, NFATC1, MAPK14, SELE, F3, IL1A, COL3A1, IL6, IL1B, BCL2, BAX | 5.76E-25 |
| hsa04218:Cellular senescence | 28 | 14.73684 | 3.76E-18 | RB1, CDKN1A, CXCL8, SERPINE1, PTEN, RELA, PPP3CA, CCND1, CHEK2, MYC, CHEK1, E2F1, AKT1, MAPK1, TGFB1, CDKN2A, IGFBP3, NFATC1, MAPK14, SIRT1, CCNA2, IL1A, IL6, CDK2, MDM2, CALM3, RAF1, TP53 | 3.18E-17 |
| hsa05160:Hepatitis C | 28 | 14.73684 | 5.28E-18 | RB1, GSK3B, CDKN1A, TNF, RELA, EGFR, CASP9, IKBKB, CASP8, RXRA, CCND1, MYC, CASP3, E2F1, AKT1, MAPK1, CHUK, STAT1, NFKBIA, CXCL10, IFNG, CDK2, BAX, CTNNB1, CYCS, RAF1, PPARA, TP53 | 3.85E-17 |
| hsa05169:Epstein-Barr virus infection | 28 | 14.73684 | 3.60E-15 | RB1, CDKN1A, TNF, RELA, ICAM1, CASP9, IKBKB, MAPK8, CASP8, CCND1, MYC, CASP3, E2F1, AKT1, JUN, CHUK, STAT1, MAPK14, NFKBIA, CCNA2, CXCL10, IL6, CDK2, BCL2, MDM2, BAX, CYCS, TP53 | 1.41E-14 |
| hsa05215:Prostate cancer | 27 | 14.21053 | 1.21E-22 | RB1, GSK3B, CDKN1A, PTEN, PLAT, RELA, EGFR, CASP9, IKBKB, CCND1, PLAU, ERBB2, E2F1, AKT1, MAPK1, HSP90AA1, CHUK, MMP3, MMP9, NFKBIA, AR, CDK2, BCL2, MDM2, CTNNB1, RAF1, TP53 | 1.54E-21 |
| hsa04668:TNF signaling pathway | 27 | 14.21053 | 1.09E-20 | XIAP, PTGS2, TNF, CXCL2, RELA, ICAM1, IKBKB, CASP7, MAPK8, CASP8, CASP3, CCL2, AKT1, MAPK1, JUN, VCAM1, CHUK, MMP3, FOS, MAPK14, SELE, MMP9, NFKBIA, CXCL10, IL6, IRF1, IL1B | 1.11E-19 |
| hsa05225:Hepatocellular carcinoma | 27 | 14.21053 | 2.85E-16 | RB1, GSK3B, CDKN1A, PTEN, ELK1, EGFR, CCND1, MYC, E2F1, AKT1, HMOX1, MAPK1, NQO1, GSTM1, TGFB1, CDKN2A, PRKCB, IGF2, PRKCA, GSTA2, BAX, CTNNB1, RAF1, MET, TP53, BCL2L1, NFE2L2 | 1.53E-15 |
| hsa05205:Proteoglycans in cancer | 27 | 14.21053 | 4.32E-14 | CDKN1A, ELK1, HIF1A, TNF, EGFR, CCND1, PLAU, MYC, CASP3, ERBB2, KDR, AKT1, MAPK1, TGFB1, PRKCB, CAV1, MMP2, IGF2, PRKCA, MAPK14, MMP9, ESR1, MDM2, CTNNB1, RAF1, MET, TP53 | 1.42E-13 |
| hsa05208:Chemical carcinogenesis - reactive oxygen species | 27 | 14.21053 | 3.27E-13 | MT-ND6, NCF1, PTEN, AHR, HIF1A, RELA, EGFR, IKBKB, MAPK8, CYP1B1, AKT1, HMOX1, MAPK1, NQO1, PTPN1, JUN, GSTM1, CHUK, FOS, MAPK14, SOD1, NFKBIA, GSTA2, CYP1A1, RAF1, MET, NFE2L2 | 8.54E-13 |
| hsa05010:Alzheimer disease | 27 | 14.21053 | 5.32E-08 | MT-ND6, APP, GSK3B, CHRM1, CHRNA7, PTGS2, TNF, RELA, CASP9, IKBKB, PPP3CA, CASP7, MAPK8, CASP8, CASP3, AKT1, MAPK1, NOS2, CHUK, INSR, IL1A, IL6, IL1B, CTNNB1, CYCS, CALM3, RAF1 | 6.62E-08 |
| hsa04657:IL-17 signaling pathway | 26 | 13.68421 | 1.04E-21 | GSK3B, CXCL8, PTGS2, TNF, CXCL2, RELA, IKBKB, MAPK8, CASP8, CASP3, CCL2, MAPK1, JUN, HSP90AA1, CHUK, MMP1, MMP3, FOS, MAPK14, MMP9, IL4, NFKBIA, CXCL10, IL6, IFNG, IL1B | 1.18E-20 |
| hsa05152:Tuberculosis | 26 | 13.68421 | 1.65E-14 | TNF, RELA, CASP9, PPP3CA, MAPK8, CASP8, CASP3, AKT1, MAPK1, LBP, CD14, CTSD, IL10, TGFB1, NOS2, STAT1, MAPK14, IL1A, IL6, IFNG, IL1B, BCL2, BAX, CYCS, CALM3, RAF1 | 5.82E-14 |
| hsa05132:Salmonella infection | 26 | 13.68421 | 2.78E-11 | CXCL8, TNF, RELA, PIK3CG, IKBKB, CASP7, MAPK8, CASP8, MYC, CASP3, AKT1, MAPK1, CD14, JUN, HSP90AA1, CHUK, FOS, MAPK14, NFKBIA, IL6, IL1B, BCL2, BAX, CTNNB1, CYCS, RAF1 | 5.44E-11 |
| hsa05165:Human papillomavirus infection | 26 | 13.68421 | 1.13E-08 | RB1, GSK3B, CDKN1A, PTEN, PTGS2, TNF, RELA, EGFR, IKBKB, CASP8, CCND1, CASP3, E2F1, SPP1, AKT1, MAPK1, CHUK, STAT1, CCNA2, IRF1, CDK2, MDM2, BAX, CTNNB1, RAF1, TP53 | 1.61E-08 |
| hsa05170:Human immunodeficiency virus 1 infection | 25 | 13.15789 | 5.53E-12 | TNF, RELA, CASP9, IKBKB, PPP3CA, MAPK8, CASP8, CASP3, CHEK1, AKT1, MAPK1, JUN, CHUK, PRKCB, PRKCA, NFATC1, FOS, MAPK14, NFKBIA, BCL2, BAX, CYCS, CALM3, RAF1, BCL2L1 | 1.23E-11 |
| hsa05145:Toxoplasmosis | 24 | 12.63158 | 2.09E-17 | IL10, TGFB1, NOS2, CHUK, STAT1, XIAP, MAPK14, TNF, PIK3CG, RELA, CASP9, NFKBIA, IKBKB, MAPK8, CASP8, CD40LG, IFNG, CASP3, ALOX5, BCL2, AKT1, MAPK1, CYCS, BCL2L1 | 1.33E-16 |
| hsa04210:Apoptosis | 24 | 12.63158 | 2.52E-15 | JUN, PARP1, CHUK, XIAP, FOS, TNF, RELA, CASP9, NFKBIA, IKBKB, CASP7, MAPK8, CASP8, CASP3, BCL2, BAX, BIRC5, AKT1, MAPK1, CYCS, RAF1, CTSD, TP53, BCL2L1 | 1.07E-14 |
| hsa05162:Measles | 24 | 12.63158 | 3.53E-15 | GSK3B, JUN, CHUK, STAT1, FOS, IL2, RELA, CASP9, NFKBIA, IKBKB, IL1A, IL6, MAPK8, CASP8, CCND1, IL1B, CASP3, CDK2, BCL2, BAX, AKT1, CYCS, TP53, BCL2L1 | 1.41E-14 |
| hsa05164:Influenza A | 24 | 12.63158 | 4.06E-13 | CXCL8, CHUK, PRKCB, STAT1, PRKCA, TNF, RELA, ICAM1, CASP9, NFKBIA, IKBKB, IL1A, CXCL10, IL6, CASP8, IFNG, IL1B, CASP3, BAX, CCL2, AKT1, MAPK1, CYCS, RAF1 | 1.04E-12 |
| hsa05206:MicroRNAs in cancer | 24 | 12.63158 | 6.45E-08 | CDKN1A, CDKN2A, PRKCB, PTEN, PRKCA, PTGS2, SIRT1, MMP9, EGFR, IKBKB, CCND1, PLAU, MYC, CASP3, ERBB2, MDM2, E2F1, BCL2, CYP1B1, HMOX1, MAPK1, RAF1, MET, TP53 | 7.93E-08 |
| hsa05222:Small cell lung cancer | 23 | 12.10526 | 4.05E-18 | RB1, CDKN1A, NOS2, CHUK, PTEN, XIAP, PTGS2, RELA, CASP9, NFKBIA, IKBKB, RXRA, CCND1, MYC, CASP3, CDK2, E2F1, BCL2, BAX, AKT1, CYCS, TP53, BCL2L1 | 3.18E-17 |
| hsa04625:C-type lectin receptor signaling pathway | 23 | 12.10526 | 6.79E-17 | IL10, JUN, CHUK, STAT1, NFATC1, PTGS2, MAPK14, TNF, IL2, RELA, NFKBIA, IKBKB, PPP3CA, IL6, MAPK8, CASP8, IL1B, IRF1, MDM2, AKT1, MAPK1, CALM3, RAF1 | 4.08E-16 |
| hsa04932:Non-alcoholic fatty liver disease | 23 | 12.10526 | 4.46E-13 | GSK3B, JUN, TGFB1, CXCL8, INSR, FOS, MAPK14, TNF, RELA, IKBKB, IL1A, CASP7, IL6, MAPK8, CASP8, RXRA, IL1B, CASP3, BAX, AKT1, CYCS, PPARG, PPARA | 1.11E-12 |
| hsa05171:Coronavirus disease - COVID-19 | 23 | 12.10526 | 1.54E-09 | JUN, CXCL8, CHUK, PRKCB, STAT1, MMP1, MMP3, PRKCA, FOS, F2, MAPK14, TNF, EGFR, IL2, RELA, NFKBIA, IKBKB, CXCL10, IL6, MAPK8, IL1B, CCL2, MAPK1 | 2.50E-09 |
| hsa01522:Endocrine resistance | 22 | 11.57895 | 2.71E-16 | RB1, CDKN1A, JUN, CDKN2A, MMP2, FOS, MAPK14, ESR1, MMP9, EGFR, ESR2, MAPK8, CCND1, ERBB2, MDM2, E2F1, BCL2, BAX, AKT1, MAPK1, RAF1, TP53 | 1.53E-15 |
| hsa05142:Chagas disease | 22 | 11.57895 | 6.43E-16 | IL10, JUN, TGFB1, CXCL8, NOS2, CHUK, SERPINE1, FOS, MAPK14, TNF, IL2, RELA, NFKBIA, IKBKB, IL6, MAPK8, CASP8, IFNG, IL1B, CCL2, AKT1, MAPK1 | 3.12E-15 |
| hsa04659:Th17 cell differentiation | 22 | 11.57895 | 2.41E-15 | JUN, HSP90AA1, TGFB1, CHUK, STAT1, NFATC1, AHR, FOS, MAPK14, HIF1A, IL2, RELA, IL4, NFKBIA, IKBKB, PPP3CA, IL6, MAPK8, RXRA, IFNG, IL1B, MAPK1 | 1.07E-14 |
| hsa04936:Alcoholic liver disease | 22 | 11.57895 | 6.91E-13 | GSK3B, CXCL8, CHUK, MAPK14, CXCL2, SIRT1, TNF, RELA, ACACA, NFKBIA, IKBKB, IL6, MAPK8, CASP8, CCND1, IL1B, CASP3, AKT1, CTNNB1, CD14, LBP, PPARA | 1.64E-12 |
| hsa05131:Shigellosis | 22 | 11.57895 | 2.44E-08 | GSK3B, JUN, CXCL8, CHUK, MAPK14, TNF, EGFR, RELA, HK2, NFKBIA, IKBKB, MAPK8, IL1B, MDM2, BCL2, BAX, AKT1, MAPK1, CYCS, CD14, TP53, BCL2L1 | 3.23E-08 |
| hsa04620:Toll-like receptor signaling pathway | 21 | 11.05263 | 3.04E-14 | JUN, CXCL8, CHUK, STAT1, FOS, MAPK14, TNF, RELA, NFKBIA, IKBKB, CXCL10, CXCL11, IL6, MAPK8, CASP8, IL1B, SPP1, AKT1, MAPK1, CD14, LBP | 1.03E-13 |
| hsa05224:Breast cancer | 21 | 11.05263 | 1.24E-11 | RB1, NCOA1, GSK3B, CDKN1A, JUN, PTEN, FOS, ESR1, EGFR, ESR2, CCND1, MYC, ERBB2, E2F1, BAX, AKT1, MAPK1, CTNNB1, PGR, RAF1, TP53 | 2.63E-11 |
| hsa05202:Transcriptional misregulation in cancer | 21 | 11.05263 | 1.78E-09 | CDKN1A, CXCL8, IGFBP3, MMP3, PLAT, MPO, MMP9, RELA, RUNX2, CCNA2, IL6, RXRA, PLAU, MYC, MDM2, BAX, PPARG, CD14, MET, TP53, BCL2L1 | 2.75E-09 |
| hsa05212:Pancreatic cancer | 20 | 10.52632 | 3.53E-16 | RB1, CDKN1A, TGFB1, CHUK, CDKN2A, STAT1, EGFR, RELA, CASP9, IKBKB, MAPK8, CCND1, ERBB2, E2F1, BAX, AKT1, MAPK1, RAF1, TP53, BCL2L1 | 1.80E-15 |
| hsa05210:Colorectal cancer | 20 | 10.52632 | 4.53E-15 | GSK3B, CDKN1A, JUN, TGFB1, FOS, EGFR, CASP9, MAPK8, CCND1, MYC, CASP3, BCL2, BAX, BIRC5, AKT1, MAPK1, CTNNB1, CYCS, RAF1, TP53 | 1.71E-14 |
| hsa04064:NF-kappa B signaling pathway | 20 | 10.52632 | 1.80E-13 | VCAM1, CXCL8, PARP1, CHUK, PRKCB, XIAP, PTGS2, CXCL2, TNF, RELA, ICAM1, NFKBIA, IKBKB, CD40LG, PLAU, IL1B, BCL2, CD14, LBP, BCL2L1 | 4.95E-13 |
| hsa04660:T cell receptor signaling pathway | 20 | 10.52632 | 3.07E-12 | IL10, GSK3B, JUN, CHUK, NFATC1, FOS, MAPK14, TNF, IL2, RELA, IL4, NFKBIA, IKBKB, PPP3CA, MAPK8, CD40LG, IFNG, AKT1, MAPK1, RAF1 | 7.12E-12 |
| hsa04380:Osteoclast differentiation | 20 | 10.52632 | 2.26E-11 | JUN, TGFB1, NCF1, CHUK, STAT1, NFATC1, FOS, MAPK14, TNF, RELA, NFKBIA, IKBKB, IL1A, PPP3CA, MAPK8, IFNG, IL1B, AKT1, MAPK1, PPARG | 4.53E-11 |
| hsa04621:NOD-like receptor signaling pathway | 20 | 10.52632 | 5.95E-09 | JUN, HSP90AA1, CXCL8, CHUK, STAT1, XIAP, MAPK14, CXCL2, TNF, RELA, NFKBIA, IKBKB, IL6, MAPK8, CASP8, IL1B, BCL2, CCL2, MAPK1, BCL2L1 | 8.55E-09 |
| hsa05130:Pathogenic Escherichia coli infection | 20 | 10.52632 | 1.68E-08 | JUN, CXCL8, CHUK, FOS, F2, MAPK14, TNF, RELA, CASP9, NFKBIA, IKBKB, CASP7, IL6, MAPK8, CASP8, IL1B, CASP3, BAX, MAPK1, CYCS | 2.31E-08 |
| hsa05415:Diabetic cardiomyopathy | 20 | 10.52632 | 2.52E-08 | MT-ND6, GSK3B, TGFB1, PARP1, NCF1, PRKCB, NOS3, MMP2, INSR, PTEN, PRKCA, SLC2A4, MAPK14, MMP9, RELA, COL3A1, MAPK8, AKT1, PPARA, CTSD | 3.26E-08 |
| hsa04510:Focal adhesion | 20 | 10.52632 | 2.52E-08 | GSK3B, JUN, PRKCB, CAV1, PTEN, XIAP, PRKCA, ELK1, EGFR, MAPK8, CCND1, ERBB2, SPP1, KDR, BCL2, AKT1, MAPK1, CTNNB1, RAF1, MET | 3.26E-08 |
| hsa04020:Calcium signaling pathway | 20 | 10.52632 | 8.18E-07 | CHRM2, CHRM1, NOS2, PRKCB, NOS3, CHRNA7, PTGER3, NFATC1, PRKCA, ADRB1, ADRB2, HTR2A, ADRA1B, EGFR, PPP3CA, ERBB2, KDR, CALM3, DRD1, MET | 8.78E-07 |
| hsa05140:Leishmaniasis | 19 | 10 | 8.36E-15 | IL10, JUN, TGFB1, NOS2, NCF1, PRKCB, STAT1, FOS, PTGS2, MAPK14, ELK1, TNF, RELA, IL4, NFKBIA, IL1A, IFNG, IL1B, MAPK1 | 3.04E-14 |
| hsa04066:HIF-1 signaling pathway | 19 | 10 | 4.89E-12 | EGLN1, CDKN1A, NOS2, PRKCB, NOS3, INSR, SERPINE1, PRKCA, HIF1A, EGFR, RELA, HK2, IL6, IFNG, ERBB2, BCL2, AKT1, HMOX1, MAPK1 | 1.11E-11 |
| hsa04068:FoxO signaling pathway | 19 | 10 | 1.18E-10 | IL10, CDKN1A, TGFB1, CHUK, INSR, PTEN, SLC2A4, MAPK14, SIRT1, EGFR, IKBKB, IL6, MAPK8, CCND1, CDK2, MDM2, AKT1, MAPK1, RAF1 | 2.23E-10 |
| hsa05135:Yersinia infection | 19 | 10 | 2.52E-10 | IL10, GSK3B, JUN, CXCL8, CHUK, NFATC1, FOS, MAPK14, TNF, IL2, RELA, NFKBIA, IKBKB, IL6, MAPK8, IL1B, CCL2, AKT1, MAPK1 | 4.58E-10 |
| hsa05226:Gastric cancer | 19 | 10 | 1.02E-09 | RB1, GSK3B, CDKN1A, TGFB1, EGFR, RXRA, CCND1, MYC, ERBB2, CDK2, E2F1, BCL2, BAX, AKT1, MAPK1, CTNNB1, RAF1, MET, TP53 | 1.70E-09 |
| hsa04024:cAMP signaling pathway | 19 | 10 | 6.45E-07 | CHRM2, GRIA2, JUN, CHRM1, PTGER3, NFATC1, ADRB1, FOS, ADRB2, RELA, NFKBIA, MAPK8, PDE3A, AKT1, MAPK1, CALM3, DRD1, RAF1, PPARA | 7.08E-07 |
| hsa05168:Herpes simplex virus 1 infection | 19 | 10 | 0.015991 | CHUK, STAT1, TNF, RELA, CASP9, NFKBIA, IKBKB, IL6, CASP8, IFNG, IL1B, CASP3, BCL2, BAX, CCL2, AKT1, CYCS, TP53, BCL2L1 | 0.015991 |
| hsa01524:Platinum drug resistance | 18 | 9.473684 | 5.18E-14 | CDKN1A, GSTM1, CDKN2A, XIAP, CASP9, CASP8, CASP3, GSTA2, ERBB2, MDM2, BCL2, BAX, BIRC5, AKT1, MAPK1, CYCS, TP53, BCL2L1 | 1.65E-13 |
| hsa04115:p53 signaling pathway | 18 | 9.473684 | 6.61E-14 | CDKN1A, CDKN2A, IGFBP3, SERPINE1, PTEN, CASP9, CASP8, CCND1, CHEK2, CASP3, CHEK1, CDK2, MDM2, BCL2, BAX, CYCS, TP53, BCL2L1 | 2.04E-13 |
| hsa05133:Pertussis | 18 | 9.473684 | 1.06E-13 | IL10, JUN, CXCL8, NOS2, FOS, MAPK14, TNF, RELA, IL1A, CASP7, IL6, MAPK8, IL1B, CASP3, IRF1, MAPK1, CALM3, CD14 | 3.10E-13 |
| hsa05220:Chronic myeloid leukemia | 18 | 9.473684 | 1.06E-13 | RB1, CDKN1A, TGFB1, CHUK, CDKN2A, RELA, NFKBIA, IKBKB, CCND1, MYC, MDM2, E2F1, BAX, AKT1, MAPK1, RAF1, TP53, BCL2L1 | 3.10E-13 |
| hsa04919:Thyroid hormone signaling pathway | 18 | 9.473684 | 2.79E-10 | NCOA1, GSK3B, PRKCB, STAT1, DIO1, PRKCA, HIF1A, ESR1, CASP9, RXRA, CCND1, MYC, MDM2, AKT1, MAPK1, CTNNB1, RAF1, TP53 | 4.99E-10 |
| hsa04926:Relaxin signaling pathway | 18 | 9.473684 | 7.76E-10 | JUN, TGFB1, NOS2, NOS3, MMP1, MMP2, PRKCA, FOS, MAPK14, MMP9, EGFR, RELA, NFKBIA, COL3A1, MAPK8, AKT1, MAPK1, RAF1 | 1.32E-09 |
| hsa04915:Estrogen signaling pathway | 18 | 9.473684 | 2.00E-09 | NCOA1, JUN, HSP90AA1, NOS3, MMP2, FOS, OPRM1, ESR1, MMP9, EGFR, ESR2, BCL2, AKT1, MAPK1, CALM3, PGR, RAF1, CTSD | 3.05E-09 |
| hsa04014:Ras signaling pathway | 18 | 9.473684 | 5.64E-06 | CHUK, PRKCB, INSR, IGF2, PRKCA, ELK1, EGFR, RELA, IKBKB, MAPK8, RASA1, KDR, AKT1, MAPK1, CALM3, RAF1, MET, BCL2L1 | 5.64E-06 |
| hsa05020:Prion disease | 18 | 9.473684 | 3.61E-05 | MT-ND6, GSK3B, HSPA5, NCF1, CAV1, MAPK14, TNF, SOD1, CASP9, IL1A, PPP3CA, IL6, MAPK8, IL1B, CASP3, BAX, MAPK1, CYCS | 3.61E-05 |
| hsa04080:Neuroactive ligand-receptor interaction | 18 | 9.473684 | 0.001252 | CHRM2, OPRD1, GRIA2, GABRA1, CHRM1, CHRNA2, CHRNA7, PTGER3, ADRB1, ADRB2, HTR2A, OPRM1, F2, ADRA1B, ADRA2C, NR3C1, ADRA2A, DRD1 | 0.001252 |
| hsa05223:Non-small cell lung cancer | 17 | 8.947368 | 6.51E-13 | RB1, CDKN1A, CDKN2A, PRKCB, PRKCA, EGFR, CASP9, RXRA, CCND1, ERBB2, E2F1, BAX, AKT1, MAPK1, RAF1, MET, TP53 | 1.58E-12 |
| hsa05235:PD-L1 expression and PD-1 checkpoint pathway in cancer | 17 | 8.947368 | 2.07E-11 | JUN, CHUK, STAT1, PTEN, NFATC1, FOS, MAPK14, HIF1A, EGFR, RELA, NFKBIA, IKBKB, PPP3CA, IFNG, AKT1, MAPK1, RAF1 | 4.22E-11 |
| hsa04022:cGMP-PKG signaling pathway | 17 | 8.947368 | 2.27E-07 | OPRD1, NOS3, INSR, NFATC1, ADRB1, ADRB2, ADRA1B, ADRA2C, PIK3CG, ADRA2A, PPP3CA, KCNMA1, PDE3A, AKT1, MAPK1, CALM3, RAF1 | 2.60E-07 |
| hsa04062:Chemokine signaling pathway | 17 | 8.947368 | 1.63E-06 | GSK3B, CXCL8, NCF1, CHUK, PRKCB, STAT1, CXCL2, PIK3CG, RELA, NFKBIA, IKBKB, CXCL10, CXCL11, CCL2, AKT1, MAPK1, RAF1 | 1.72E-06 |
| hsa05219:Bladder cancer | 16 | 8.421053 | 8.85E-16 | RB1, CDKN1A, CXCL8, CDKN2A, MMP1, MMP2, MMP9, EGFR, CCND1, MYC, ERBB2, MDM2, E2F1, MAPK1, RAF1, TP53 | 4.10E-15 |
| hsa05214:Glioma | 16 | 8.421053 | 1.80E-11 | RB1, CDKN1A, CDKN2A, PRKCB, PTEN, PRKCA, EGFR, CCND1, MDM2, E2F1, BAX, AKT1, MAPK1, CALM3, RAF1, TP53 | 3.75E-11 |
| hsa05146:Amoebiasis | 16 | 8.421053 | 1.71E-09 | IL10, TGFB1, CXCL8, NOS2, PRKCB, HSPB1, PRKCA, CXCL2, TNF, RELA, COL3A1, IL6, IFNG, IL1B, CASP3, CD14 | 2.69E-09 |
| hsa04931:Insulin resistance | 16 | 8.421053 | 3.86E-09 | PTPN1, GSK3B, PRKCB, NOS3, INSR, PTEN, PYGM, SLC2A4, TNF, RELA, NFKBIA, IKBKB, IL6, MAPK8, AKT1, PPARA | 5.71E-09 |
| hsa04071:Sphingolipid signaling pathway | 16 | 8.421053 | 1.89E-08 | OPRD1, PRKCB, NOS3, PTEN, PRKCA, MAPK14, TNF, RELA, MAPK8, BCL2, BAX, AKT1, MAPK1, RAF1, CTSD, TP53 | 2.58E-08 |
| hsa04921:Oxytocin signaling pathway | 16 | 8.421053 | 4.81E-07 | CDKN1A, JUN, PRKCB, NOS3, NFATC1, PRKCA, FOS, PTGS2, ELK1, EGFR, PIK3CG, PPP3CA, CCND1, MAPK1, CALM3, RAF1 | 5.45E-07 |
| hsa05203:Viral carcinogenesis | 16 | 8.421053 | 1.61E-05 | RB1, CDKN1A, JUN, CDKN2A, RELA, NFKBIA, CCNA2, CASP8, CCND1, CASP3, CHEK1, CDK2, MDM2, BAX, MAPK1, TP53 | 1.61E-05 |
| hsa05012:Parkinson disease | 16 | 8.421053 | 3.27E-04 | MT-ND6, MAOB, HSPA5, MAOA, SLC6A3, SOD1, CASP9, MAPK8, CASP3, BAX, CYCS, CALM3, DRD1, TP53, NFE2L2, BCL2L1 | 3.27E-04 |
| hsa05213:Endometrial cancer | 15 | 7.894737 | 5.67E-12 | GSK3B, CDKN1A, PTEN, ELK1, EGFR, CASP9, CCND1, MYC, ERBB2, BAX, AKT1, MAPK1, CTNNB1, RAF1, TP53 | 1.23E-11 |
| hsa01521:EGFR tyrosine kinase inhibitor resistance | 15 | 7.894737 | 4.80E-10 | GSK3B, PRKCB, PTEN, PRKCA, EGFR, IL6, ERBB2, KDR, BCL2, BAX, AKT1, MAPK1, RAF1, MET, BCL2L1 | 8.30E-10 |
| hsa04658:Th1 and Th2 cell differentiation | 15 | 7.894737 | 3.86E-09 | JUN, CHUK, STAT1, NFATC1, FOS, MAPK14, IL2, RELA, IL4, NFKBIA, IKBKB, PPP3CA, MAPK8, IFNG, MAPK1 | 5.71E-09 |
| hsa04148:Efferocytosis | 15 | 7.894737 | 3.15E-06 | IL10, TGFB1, ODC1, NFATC1, PTGS2, MAPK14, HIF1A, SIRT1, CASP7, RXRA, CASP3, ALOX5, MAPK1, PPARG, PPARD | 3.21E-06 |
| hsa04110:Cell cycle | 15 | 7.894737 | 3.39E-06 | RB1, GSK3B, CDKN1A, TGFB1, PCNA, CDKN2A, CCNA2, CCND1, CHEK2, MYC, CHEK1, CDK2, MDM2, E2F1, TP53 | 3.43E-06 |
| hsa04060:Cytokine-cytokine receptor interaction | 15 | 7.894737 | 0.002868 | IL10, TGFB1, CXCL8, CXCL2, TNF, IL2, IL4, IL1A, CXCL10, CXCL11, IL6, CD40LG, IFNG, IL1B, CCL2 | 0.002868 |
| hsa05014:Amyotrophic lateral sclerosis | 15 | 7.894737 | 0.016156 | MT-ND6, GRIA2, NOS2, HSPA5, MAPK14, TNF, SOD1, CASP9, PPP3CA, CASP3, BCL2, BAX, CYCS, TP53, BCL2L1 | 0.016156 |
| hsa05134:Legionellosis | 14 | 7.368421 | 5.70E-11 | CXCL8, CXCL2, TNF, RELA, CASP9, NFKBIA, CASP7, IL6, CASP8, IL1B, CASP3, HSF1, CYCS, CD14 | 1.10E-10 |
| hsa05218:Melanoma | 14 | 7.368421 | 1.61E-09 | RB1, CDKN1A, CDKN2A, PTEN, EGFR, CCND1, MDM2, E2F1, BAX, AKT1, MAPK1, RAF1, MET, TP53 | 2.57E-09 |
| hsa05323:Rheumatoid arthritis | 14 | 7.368421 | 4.07E-08 | JUN, TGFB1, CXCL8, MMP1, MMP3, FOS, CXCL2, TNF, ICAM1, IL1A, IL6, IFNG, IL1B, CCL2 | 5.13E-08 |
| hsa04726:Serotonergic synapse | 14 | 7.368421 | 5.20E-07 | APP, MAOB, MAOA, PRKCB, PRKCA, ALOX12, HTR2A, PTGS2, SLC6A4, PTGS1, CASP3, ALOX5, MAPK1, RAF1 | 5.83E-07 |
| hsa04722:Neurotrophin signaling pathway | 14 | 7.368421 | 7.74E-07 | GSK3B, JUN, MAPK14, RELA, NFKBIA, IKBKB, MAPK8, BCL2, BAX, AKT1, MAPK1, CALM3, RAF1, TP53 | 8.40E-07 |
| hsa04728:Dopaminergic synapse | 14 | 7.368421 | 2.54E-06 | GRIA2, GSK3B, MAOB, MAOA, PRKCB, PRKCA, FOS, MAPK14, SLC6A3, PPP3CA, MAPK8, AKT1, CALM3, DRD1 | 2.62E-06 |
| hsa04910:Insulin signaling pathway | 14 | 7.368421 | 3.86E-06 | PTPN1, GSK3B, INSR, PYGM, SLC2A4, ELK1, HK2, ACACA, IKBKB, MAPK8, AKT1, MAPK1, CALM3, RAF1 | 3.86E-06 |
| hsa04630:JAK-STAT signaling pathway | 14 | 7.368421 | 3.12E-05 | IL10, CDKN1A, STAT1, EGFR, IL2, IL4, IL6, IFNG, CCND1, MYC, BCL2, AKT1, RAF1, BCL2L1 | 3.12E-05 |
| hsa05144:Malaria | 13 | 6.842105 | 2.11E-10 | IL10, TGFB1, VCAM1, CXCL8, SELE, TNF, ICAM1, IL6, CD40LG, IFNG, IL1B, CCL2, MET | 3.91E-10 |
| hsa05321:Inflammatory bowel disease | 13 | 6.842105 | 5.37E-09 | IL10, JUN, TGFB1, STAT1, NFATC1, TNF, IL2, RELA, IL4, IL1A, IL6, IFNG, IL1B | 7.83E-09 |
| hsa04917:Prolactin signaling pathway | 13 | 6.842105 | 1.30E-08 | GSK3B, STAT1, FOS, MAPK14, ESR1, RELA, ESR2, MAPK8, CCND1, IRF1, AKT1, MAPK1, RAF1 | 1.82E-08 |
| hsa04662:B cell receptor signaling pathway | 13 | 6.842105 | 1.08E-07 | GSK3B, JUN, CHUK, PRKCB, NFATC1, FOS, RELA, NFKBIA, IKBKB, PPP3CA, AKT1, MAPK1, RAF1 | 1.29E-07 |
| hsa04012:ErbB signaling pathway | 13 | 6.842105 | 1.23E-07 | GSK3B, CDKN1A, JUN, PRKCB, PRKCA, ELK1, EGFR, MAPK8, MYC, ERBB2, AKT1, MAPK1, RAF1 | 1.45E-07 |
| hsa04217:Necroptosis | 13 | 6.842105 | 9.62E-05 | HSP90AA1, PARP1, STAT1, XIAP, PYGM, TNF, IL1A, MAPK8, CASP8, IFNG, IL1B, BCL2, BAX | 9.62E-05 |
| hsa04310:Wnt signaling pathway | 13 | 6.842105 | 2.14E-04 | GSK3B, JUN, PRKCB, NFATC1, PRKCA, SIRT1, PPP3CA, MAPK8, CCND1, MYC, CTNNB1, TP53, PPARD | 2.14E-04 |
| hsa04370:VEGF signaling pathway | 12 | 6.315789 | 2.14E-08 | CASP9, PPP3CA, PRKCB, NOS3, KDR, HSPB1, MAPK1, AKT1, PRKCA, RAF1, MAPK14, PTGS2 | 2.87E-08 |
| hsa05221:Acute myeloid leukemia | 12 | 6.315789 | 8.54E-08 | CCNA2, IKBKB, CCND1, CHUK, MYC, MAPK1, AKT1, CD14, RAF1, MPO, RELA, PPARD | 1.04E-07 |
| hsa05031:Amphetamine addiction | 12 | 6.315789 | 1.17E-07 | PPP3CA, GRIA2, JUN, MAOB, MAOA, PRKCB, CALM3, PRKCA, FOS, DRD1, SIRT1, SLC6A3 | 1.39E-07 |
| hsa05120:Epithelial cell signaling in Helicobacter pylori infection | 12 | 6.315789 | 1.36E-07 | NFKBIA, IKBKB, JUN, MAPK8, CXCL8, CHUK, CASP3, MAPK14, CXCL2, MET, RELA, EGFR | 1.58E-07 |
| hsa04015:Rap1 signaling pathway | 12 | 6.315789 | 0.003639 | PRKCB, INSR, KDR, MAPK1, CTNNB1, AKT1, CALM3, PRKCA, RAF1, MAPK14, MET, EGFR | 0.003639 |
| hsa04215:Apoptosis - multiple species | 11 | 5.789474 | 4.07E-10 | CASP9, CASP7, MAPK8, CASP8, CASP3, BCL2, BAX, XIAP, BIRC5, CYCS, BCL2L1 | 7.16E-10 |
| hsa05230:Central carbon metabolism in cancer | 11 | 5.789474 | 1.28E-06 | MYC, ERBB2, PTEN, MAPK1, AKT1, RAF1, HIF1A, TP53, MET, EGFR, HK2 | 1.36E-06 |
| hsa04912:GnRH signaling pathway | 11 | 5.789474 | 1.75E-05 | JUN, MAPK8, PRKCB, MMP2, MAPK1, CALM3, PRKCA, RAF1, MAPK14, ELK1, EGFR | 1.75E-05 |
| hsa04725:Cholinergic synapse | 11 | 5.789474 | 9.47E-05 | CHRM2, ACHE, CHRM1, PRKCB, CHRNA7, BCL2, MAPK1, AKT1, PRKCA, FOS, PIK3CG | 9.47E-05 |
| hsa04935:Growth hormone synthesis, secretion and action | 11 | 5.789474 | 1.57E-04 | GSK3B, MAPK8, STAT1, PRKCB, IGFBP3, MAPK1, AKT1, PRKCA, FOS, RAF1, MAPK14 | 1.57E-04 |
| hsa04371:Apelin signaling pathway | 11 | 5.789474 | 5.16E-04 | CCND1, NOS2, NOS3, SERPINE1, SPP1, MAPK1, AKT1, CALM3, PLAT, RAF1, PIK3CG | 5.16E-04 |
| hsa04261:Adrenergic signaling in cardiomyocytes | 11 | 5.789474 | 0.001145 | BCL2, MAPK1, AKT1, CALM3, ADRB1, PRKCA, ADRB2, SCN5A, ADRA1B, MAPK14, PIK3CG | 0.001145 |
| hsa04934:Cushing syndrome | 11 | 5.789474 | 0.001203 | RB1, GSK3B, CDKN1A, CCND1, CDKN2A, CDK2, E2F1, MAPK1, CTNNB1, AHR, EGFR | 0.001203 |
| hsa04150:mTOR signaling pathway | 11 | 5.789474 | 0.001263 | IKBKB, GSK3B, CHUK, PRKCB, INSR, PTEN, MAPK1, AKT1, PRKCA, RAF1, TNF | 0.001263 |
| hsa05143:African trypanosomiasis | 10 | 5.263158 | 3.67E-08 | IL10, IL6, VCAM1, IFNG, PRKCB, IL1B, PRKCA, SELE, TNF, ICAM1 | 4.68E-08 |
| hsa04920:Adipocytokine signaling pathway | 10 | 5.263158 | 1.08E-05 | NFKBIA, IKBKB, MAPK8, RXRA, CHUK, AKT1, SLC2A4, PPARA, TNF, RELA | 1.08E-05 |
| hsa04622:RIG-I-like receptor signaling pathway | 10 | 5.263158 | 1.21E-05 | NFKBIA, IKBKB, CXCL10, MAPK8, CXCL8, CASP8, CHUK, MAPK14, TNF, RELA | 1.21E-05 |
| hsa04623:Cytosolic DNA-sensing pathway | 10 | 5.263158 | 4.35E-05 | NFKBIA, IKBKB, CXCL10, IL6, CASP7, CASP8, CHUK, IL1B, CASP3, RELA | 4.35E-05 |
| hsa05231:Choline metabolism in cancer | 10 | 5.263158 | 1.60E-04 | JUN, MAPK8, PRKCB, MAPK1, AKT1, PRKCA, FOS, RAF1, HIF1A, EGFR | 1.60E-04 |
| hsa04928:Parathyroid hormone synthesis, secretion and action | 10 | 5.263158 | 2.91E-04 | CDKN1A, RXRA, PRKCB, BCL2, MAPK1, PRKCA, FOS, RAF1, EGFR, RUNX2 | 2.91E-04 |
| hsa04650:Natural killer cell mediated cytotoxicity | 10 | 5.263158 | 0.001092 | PPP3CA, IFNG, PRKCB, CASP3, MAPK1, NFATC1, PRKCA, RAF1, TNF, ICAM1 | 0.001092 |
| hsa05216:Thyroid cancer | 9 | 4.736842 | 5.78E-07 | CDKN1A, RXRA, CCND1, MYC, BAX, MAPK1, CTNNB1, PPARG, TP53 | 6.41E-07 |
| hsa04664:Fc epsilon RI signaling pathway | 9 | 4.736842 | 6.48E-05 | IL4, MAPK8, ALOX5, MAPK1, AKT1, PRKCA, RAF1, MAPK14, TNF | 6.48E-05 |
| hsa05211:Renal cell carcinoma | 9 | 4.736842 | 7.20E-05 | EGLN1, CDKN1A, JUN, TGFB1, MAPK1, AKT1, RAF1, HIF1A, MET | 7.20E-05 |
| hsa04540:Gap junction | 9 | 4.736842 | 3.99E-04 | GJA1, PRKCB, MAPK1, ADRB1, PRKCA, DRD1, HTR2A, RAF1, EGFR | 3.99E-04 |
| hsa04061:Viral protein interaction with cytokine and cytokine receptor | 9 | 4.736842 | 9.40E-04 | IL10, CXCL10, IL6, CXCL11, CXCL8, CCL2, TNF, CXCL2, IL2 | 9.40E-04 |
| hsa04914:Progesterone-mediated oocyte maturation | 9 | 4.736842 | 0.00107 | CCNA2, HSP90AA1, MAPK8, CDK2, MAPK1, AKT1, PGR, RAF1, MAPK14 | 0.00107 |
| hsa04670:Leukocyte transendothelial migration | 9 | 4.736842 | 0.002311 | VCAM1, NCF1, PRKCB, MMP2, CTNNB1, PRKCA, MAPK14, MMP9, ICAM1 | 0.002311 |
| hsa04723:Retrograde endocannabinoid signaling | 9 | 4.736842 | 0.010482 | MT-ND6, GABRA1, GRIA2, MAPK8, PRKCB, MAPK1, PRKCA, MAPK14, PTGS2 | 0.010482 |
| hsa04072:Phospholipase D signaling pathway | 9 | 4.736842 | 0.010482 | CXCL8, INSR, MAPK1, AKT1, PRKCA, F2, RAF1, EGFR, PIK3CG | 0.010482 |
| hsa04140:Autophagy - animal | 9 | 4.736842 | 0.019299 | MAPK8, PTEN, BCL2, MAPK1, AKT1, RAF1, HIF1A, CTSD, BCL2L1 | 0.019299 |
| hsa04613:Neutrophil extracellular trap formation | 9 | 4.736842 | 0.040827 | NCF1, PRKCB, MAPK1, AKT1, PRKCA, RAF1, MAPK14, MPO, RELA | 0.040827 |
| hsa01523:Antifolate resistance | 8 | 4.210526 | 1.74E-06 | IKBKB, IL6, CHUK, IL1B, ALOX12, TNF, RELA, ABCG2 | 1.81E-06 |
| hsa05416:Viral myocarditis | 8 | 4.210526 | 3.95E-04 | CASP9, CASP8, CD40LG, CCND1, CASP3, CAV1, CYCS, ICAM1 | 3.95E-04 |
| hsa04610:Complement and coagulation cascades | 8 | 4.210526 | 0.001768 | THBD, F7, F10, PLAU, SERPINE1, PLAT, F2, F3 | 0.001768 |
| hsa04970:Salivary secretion | 8 | 4.210526 | 0.002769 | PRKCB, KCNMA1, CALM3, ADRB1, PRKCA, ADRB2, ADRA1B, LYZ | 0.002769 |
| hsa04750:Inflammatory mediator regulation of TRP channels | 8 | 4.210526 | 0.003716 | MAPK8, PRKCB, IL1B, CALM3, PRKCA, ALOX12, HTR2A, MAPK14 | 0.003716 |
| hsa04152:AMPK signaling pathway | 8 | 4.210526 | 0.011516 | CCNA2, CCND1, INSR, AKT1, PPARG, SLC2A4, SIRT1, ACACA | 0.011516 |
| hsa04611:Platelet activation | 8 | 4.210526 | 0.013056 | COL3A1, NOS3, MAPK1, AKT1, F2, MAPK14, PIK3CG, PTGS1 | 0.013056 |
| hsa04930:Type II diabetes mellitus | 7 | 3.684211 | 3.08E-04 | IKBKB, MAPK8, INSR, MAPK1, SLC2A4, TNF, HK2 | 3.08E-04 |
| hsa05030:Cocaine addiction | 7 | 3.684211 | 4.38E-04 | GRIA2, JUN, MAOB, MAOA, DRD1, RELA, SLC6A3 | 4.38E-04 |
| hsa04923:Regulation of lipolysis in adipocytes | 7 | 3.684211 | 0.00109 | INSR, PTGER3, AKT1, ADRB1, ADRB2, PTGS2, PTGS1 | 0.00109 |
| hsa00590:Arachidonic acid metabolism | 7 | 3.684211 | 0.001422 | CYP2B6, ALOX5, ALOX12, LTA4H, PTGS2, PTGES, PTGS1 | 0.001422 |
| hsa04929:GnRH secretion | 7 | 3.684211 | 0.001826 | PRKCB, SPP1, MAPK1, AKT1, PRKCA, RAF1, ESR2 | 0.001826 |
| hsa04720:Long-term potentiation | 7 | 3.684211 | 0.002312 | PPP3CA, GRIA2, PRKCB, MAPK1, CALM3, PRKCA, RAF1 | 0.002312 |
| hsa03320:PPAR signaling pathway | 7 | 3.684211 | 0.004083 | RXRA, FABP5, MMP1, OLR1, PPARG, PPARA, PPARD | 0.004083 |
| hsa04211:Longevity regulating pathway | 7 | 3.684211 | 0.009342 | INSR, BAX, AKT1, PPARG, SIRT1, TP53, RELA | 0.009342 |
| hsa04520:Adherens junction | 7 | 3.684211 | 0.01147 | PTPN1, INSR, ERBB2, MAPK1, CTNNB1, MET, EGFR | 0.01147 |
| hsa04916:Melanogenesis | 7 | 3.684211 | 0.016715 | GSK3B, PRKCB, MAPK1, CTNNB1, CALM3, PRKCA, RAF1 | 0.016715 |
| hsa04137:Mitophagy - animal | 7 | 3.684211 | 0.018248 | JUN, MAPK8, E2F1, HIF1A, TP53, RELA, BCL2L1 | 0.018248 |
| hsa04922:Glucagon signaling pathway | 7 | 3.684211 | 0.021598 | PPP3CA, AKT1, CALM3, PYGM, PPARA, SIRT1, ACACA | 0.021598 |
| hsa05330:Allograft rejection | 6 | 3.157895 | 9.54E-04 | IL10, IL4, CD40LG, IFNG, TNF, IL2 | 9.54E-04 |
| hsa05332:Graft-versus-host disease | 6 | 3.157895 | 0.001514 | IL1A, IL6, IFNG, IL1B, TNF, IL2 | 0.001514 |
| hsa04672:Intestinal immune network for IgA production | 6 | 3.157895 | 0.003027 | IL10, IL4, IL6, TGFB1, CD40LG, IL2 | 0.003027 |
| hsa04924:Renin secretion | 6 | 3.157895 | 0.0129 | PPP3CA, KCNMA1, PDE3A, CALM3, ADRB1, ADRB2 | 0.0129 |
| hsa05204:Chemical carcinogenesis - DNA adducts | 6 | 3.157895 | 0.013671 | GSTM1, GSTA2, CYP1A1, CYP1B1, CYP3A4, PTGS2 | 0.013671 |
| hsa00982:Drug metabolism - cytochrome P450 | 6 | 3.157895 | 0.015303 | GSTM1, CYP2B6, MAOB, MAOA, GSTA2, CYP3A4 | 0.015303 |
| hsa00980:Metabolism of xenobiotics by cytochrome P450 | 6 | 3.157895 | 0.020966 | GSTM1, CYP2B6, GSTA2, CYP1A1, CYP1B1, CYP3A4 | 0.020966 |
| hsa05032:Morphine addiction | 6 | 3.157895 | 0.037514 | GABRA1, PRKCB, PDE3A, PRKCA, DRD1, OPRM1 | 0.037514 |
| hsa04666:Fc gamma R-mediated phagocytosis | 6 | 3.157895 | 0.047255 | NCF1, PRKCB, MAPK1, AKT1, PRKCA, RAF1 | 0.047255 |
| hsa04713:Circadian entrainment | 6 | 3.157895 | 0.047255 | GRIA2, PRKCB, MAPK1, CALM3, PRKCA, FOS | 0.047255 |
| hsa04960:Aldosterone-regulated sodium reabsorption | 5 | 2.631579 | 0.006448 | PRKCB, INSR, MAPK1, PRKCA, NR3C2 | 0.006448 |
| hsa04940:Type I diabetes mellitus | 5 | 2.631579 | 0.010969 | IL1A, IFNG, IL1B, TNF, IL2 | 0.010969 |
| hsa00330:Arginine and proline metabolism | 5 | 2.631579 | 0.018352 | MAOB, NOS2, MAOA, NOS3, ODC1 | 0.018352 |
| hsa04913:Ovarian steroidogenesis | 5 | 2.631579 | 0.019607 | ALOX5, INSR, CYP1A1, CYP1B1, PTGS2 | 0.019607 |
| hsa04730:Long-term depression | 5 | 2.631579 | 0.033282 | GRIA2, PRKCB, MAPK1, PRKCA, RAF1 | 0.033282 |
| hsa05217:Basal cell carcinoma | 5 | 2.631579 | 0.038817 | GSK3B, CDKN1A, BAX, CTNNB1, TP53 | 0.038817 |
| hsa05310:Asthma | 4 | 2.105263 | 0.02443 | IL10, IL4, CD40LG, TNF | 0.02443 |
