# Supplementary material for: Elucidating the mechanism of Buyang Huanwu Decoction in the treatment of ischemic stroke: A network pharmacology and molecular docking study
Source: Medicine (Baltimore). 2026 Jul 17;105(29):e49736. doi: 10.1097/MD.0000000000049736 (PMC13384647; doi:10.1097/MD.0000000000049736)
Supplement: Supplementary file 11 [file medi-105-e49736-s011.docx]

**S 11.** The KEGG functionally-enriched pathway map for the top ten pathways

| **1.Pathways in cancer** |
| --- |
| 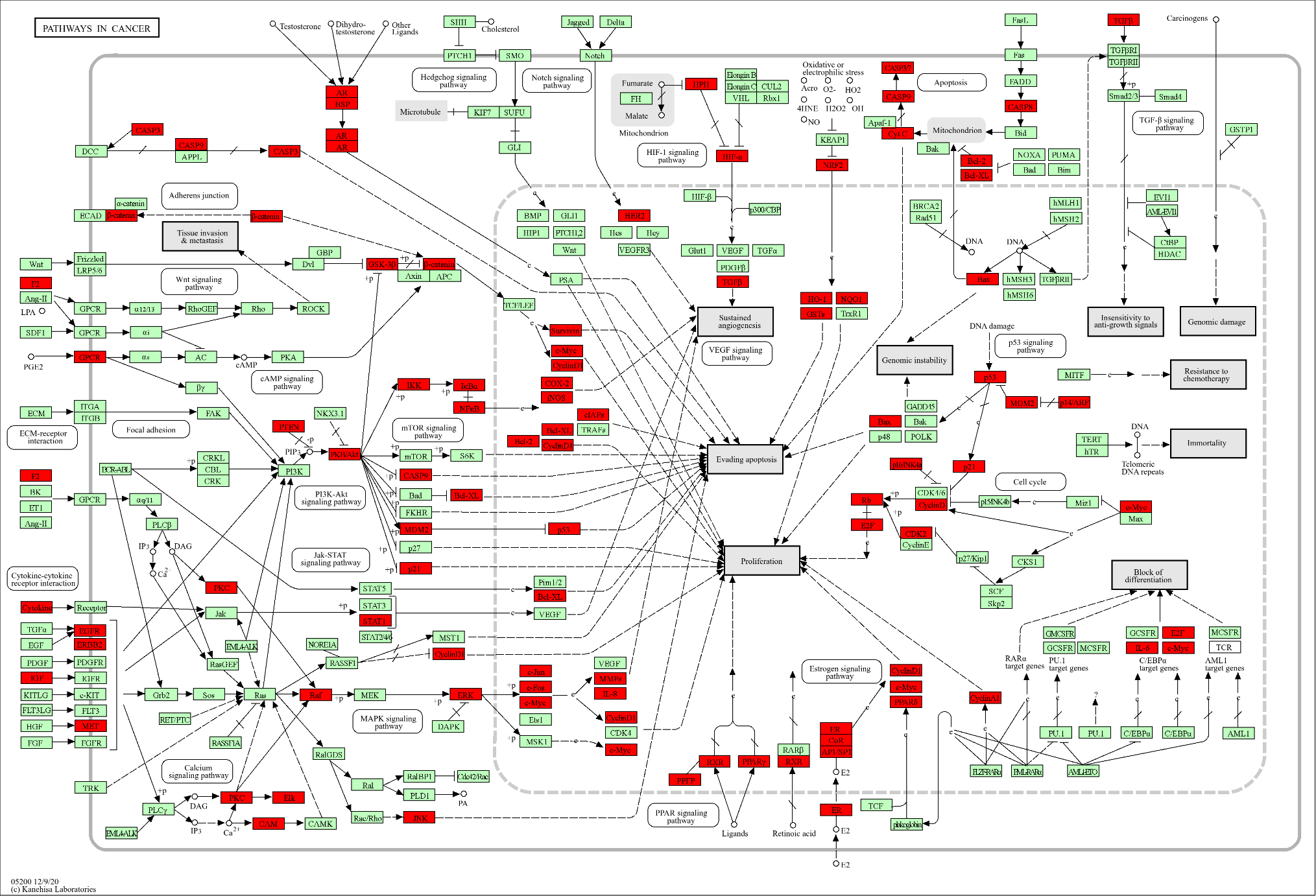 |
| 2.Lipid and atherosclerosis |
| 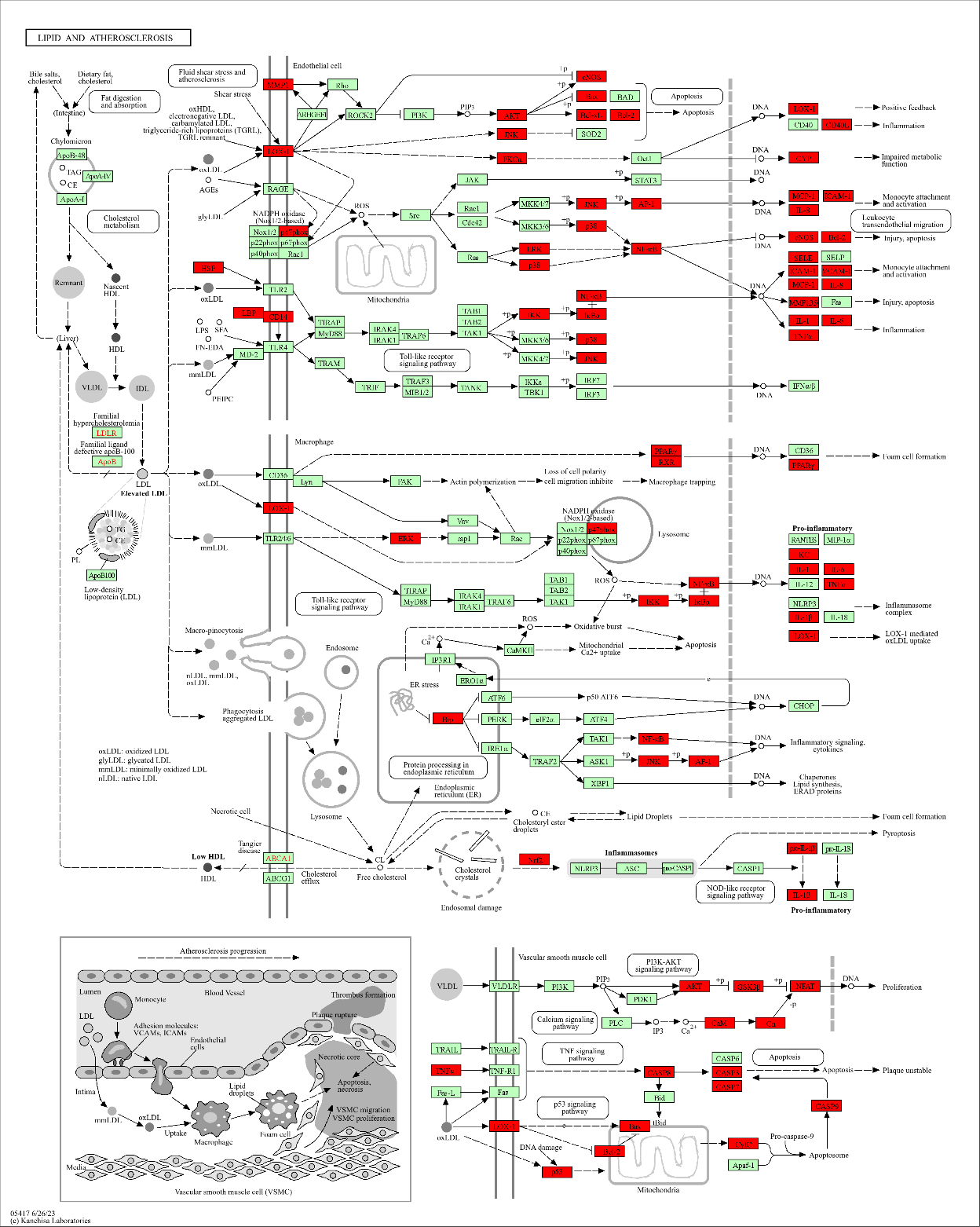 |
| **3. Human cytomegalovirus infection** |
| 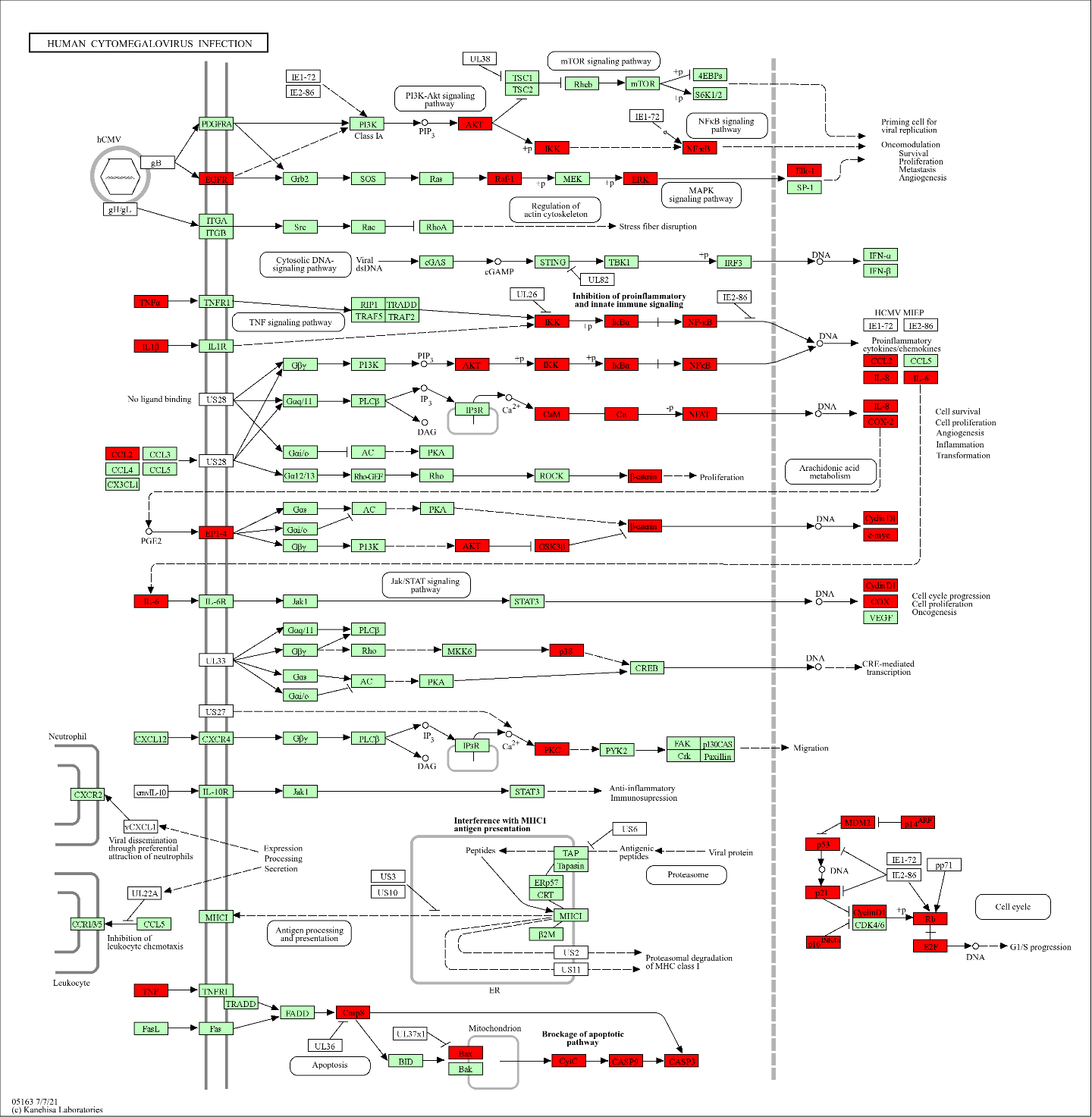 |
| **4. Hepatitis B** |
| 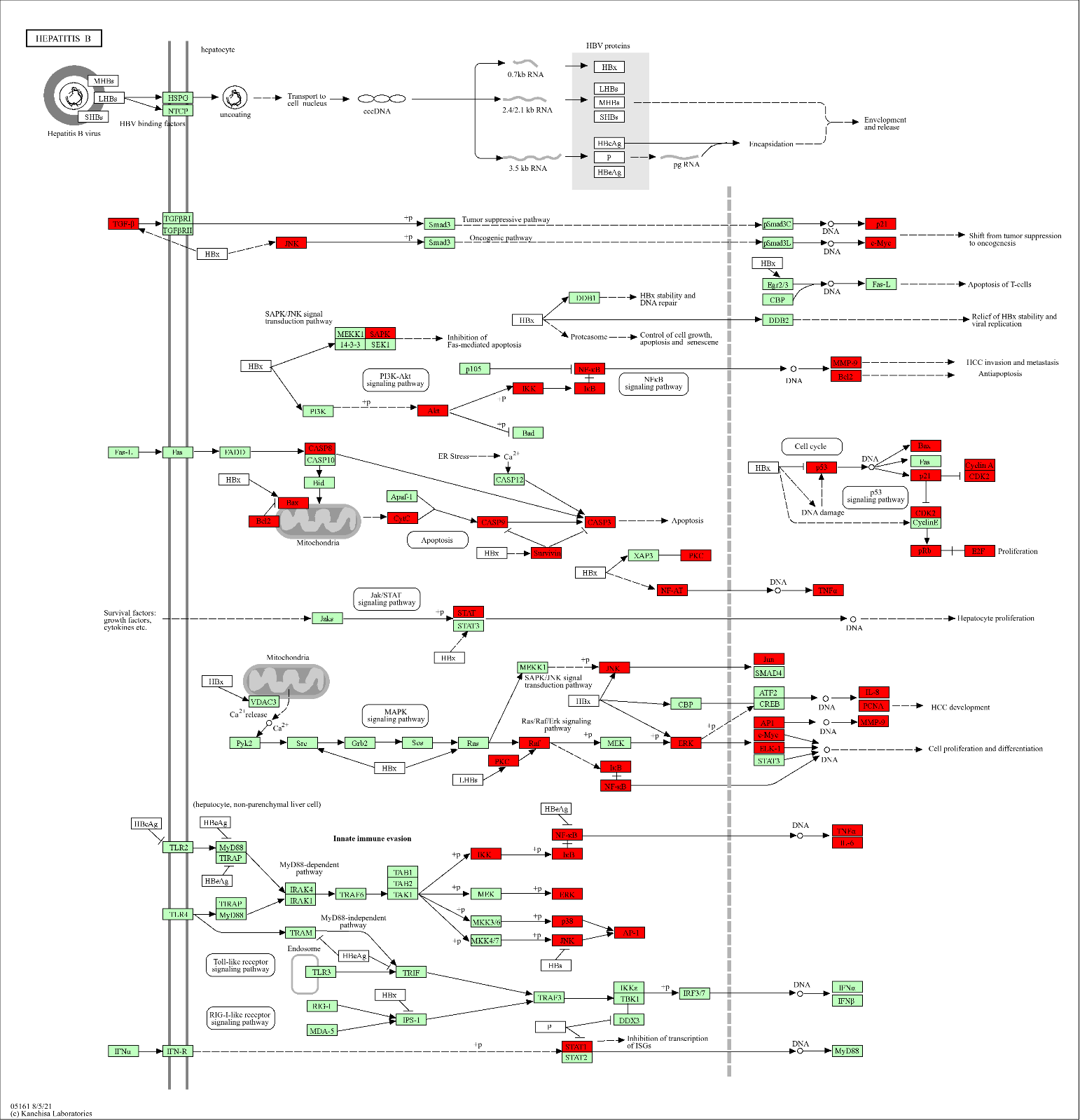 |
| **5. Kaposi sarcoma-associated herpesvirus infection** |
| 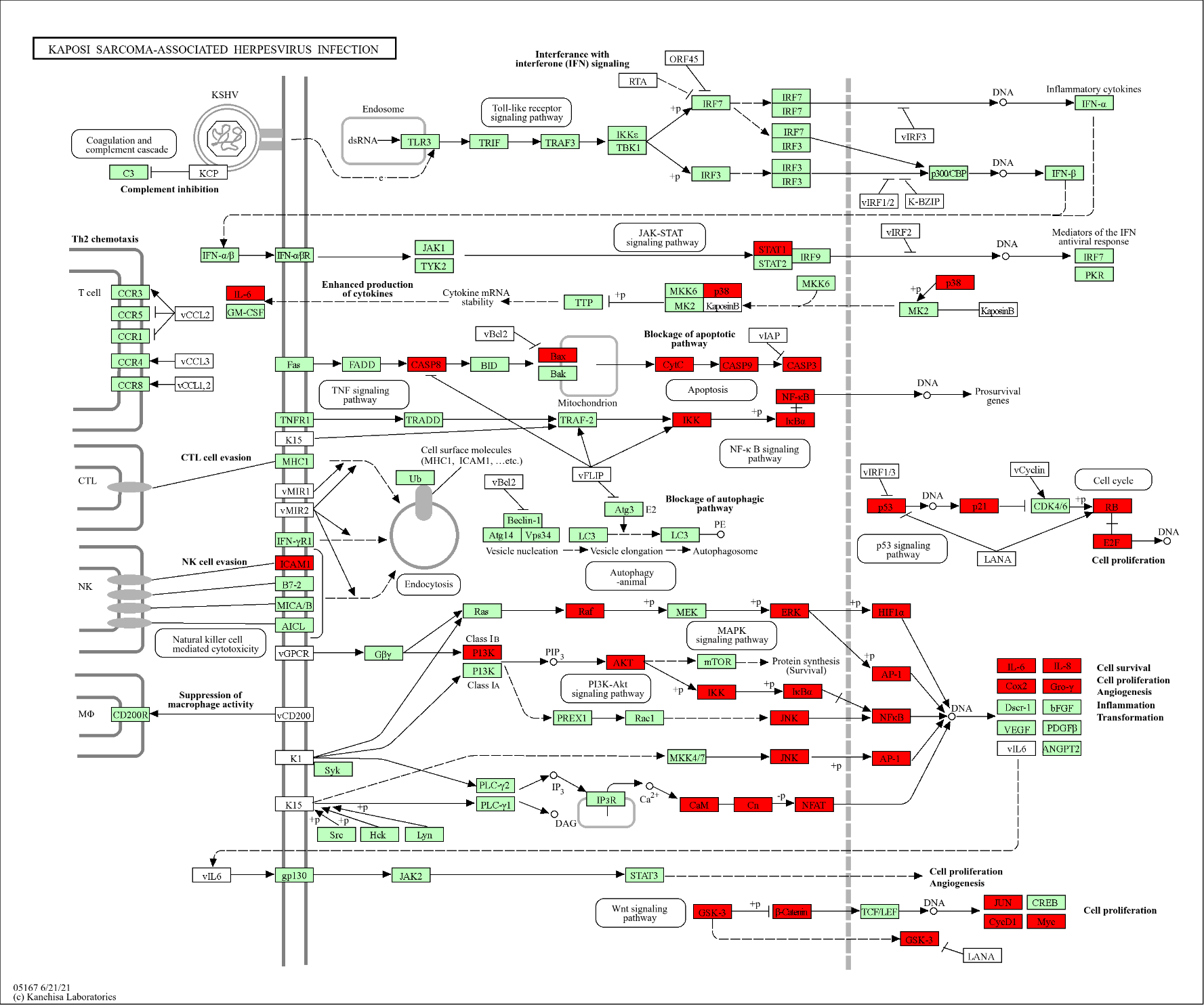 |
| **6.Fluid shear stress and atherosclerosis** |
| 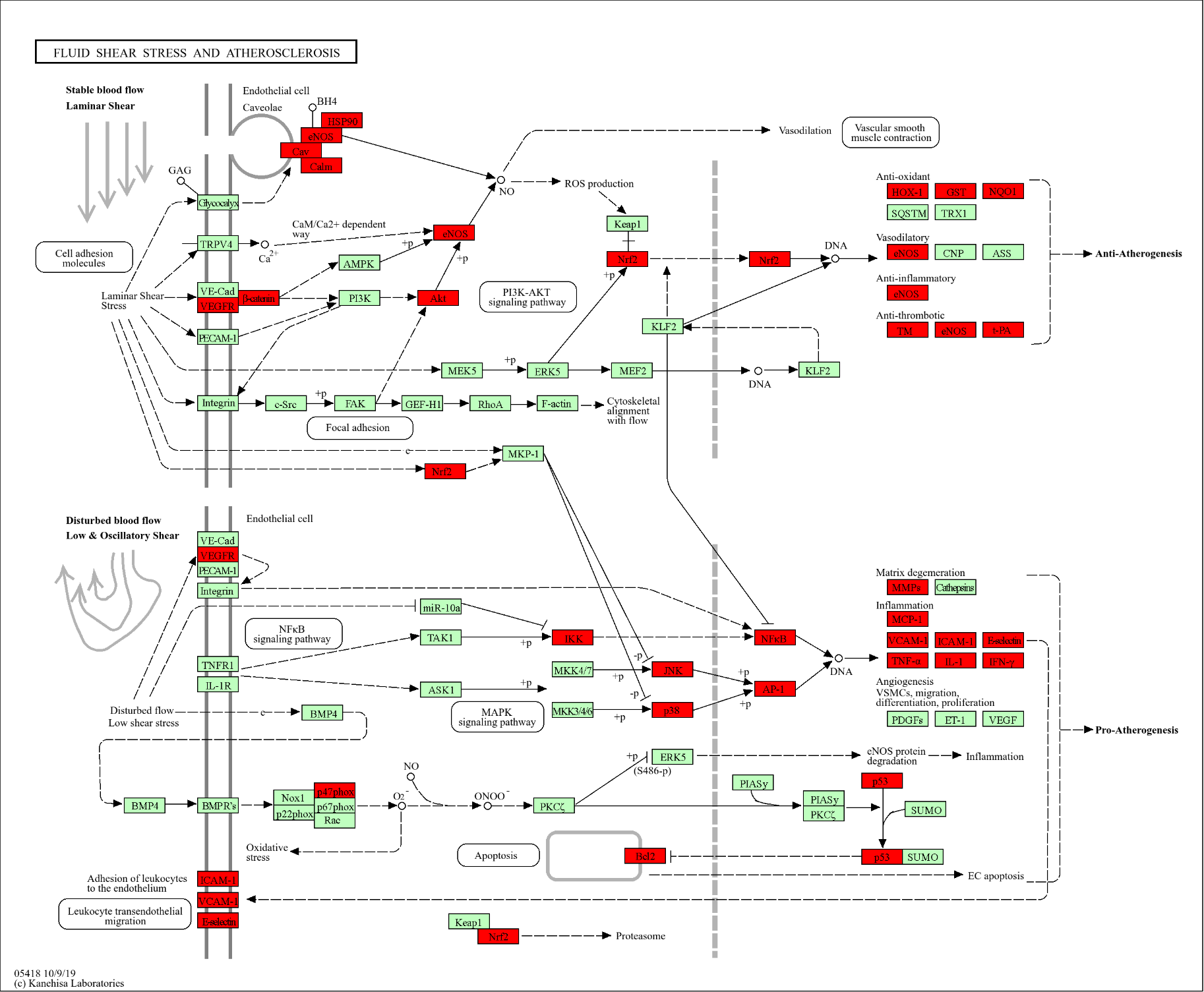 |
| **7. PI3K-Akt signaling pathway** |
| 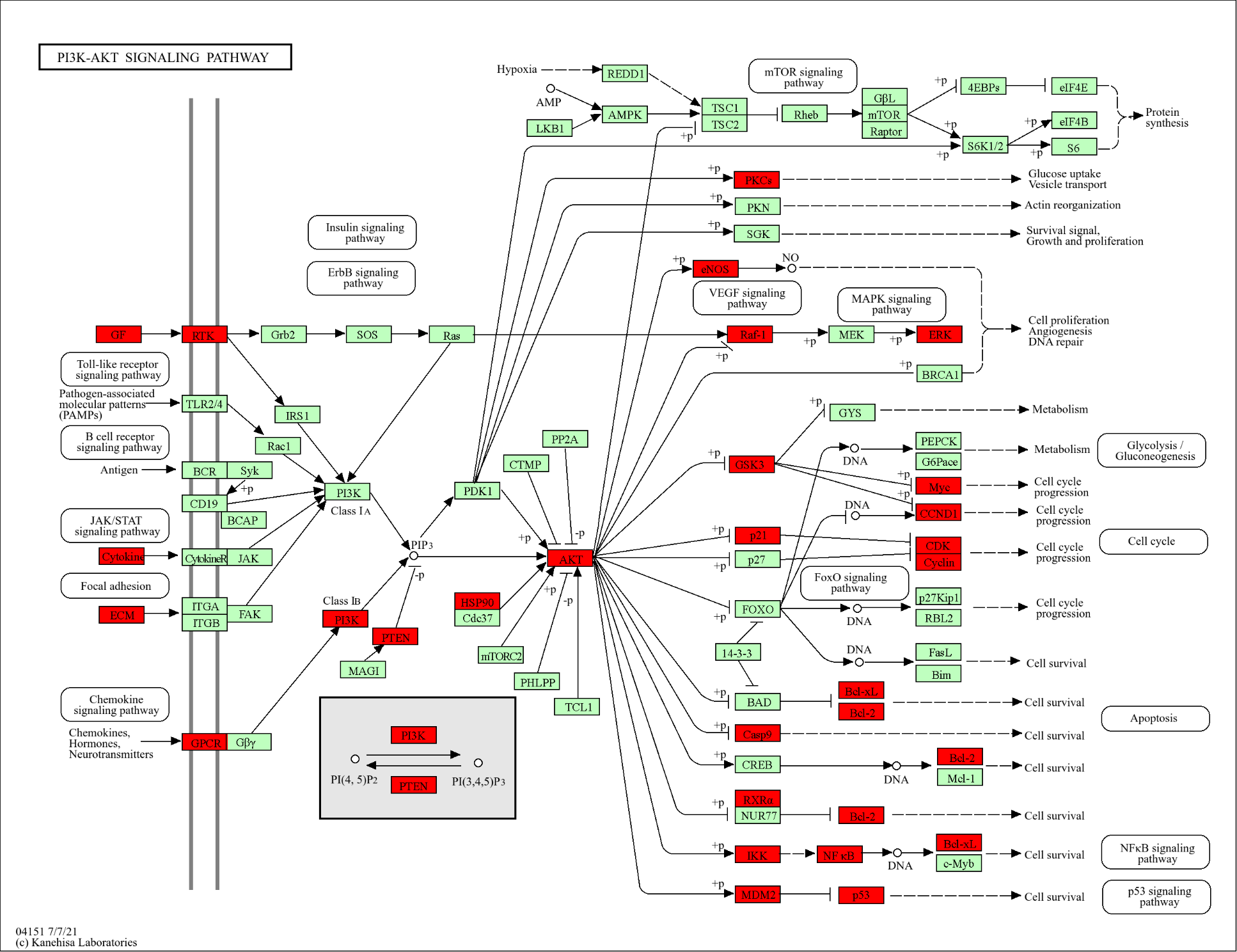 |
| **8. Chemical carcinogenesis - receptor activation** |
| 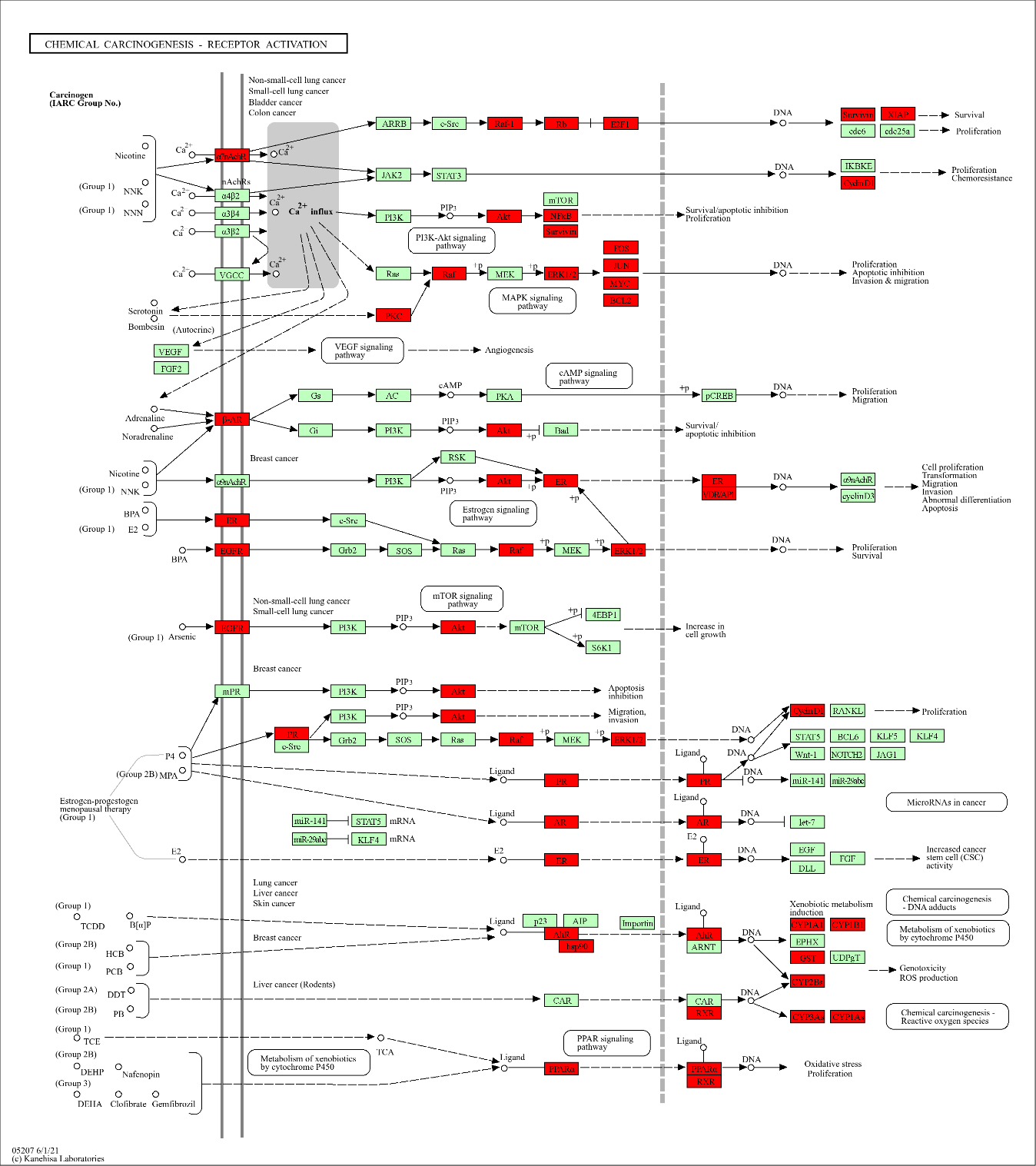 |
| **9. Pathways of neurodegeneration - multiple diseases** |
| 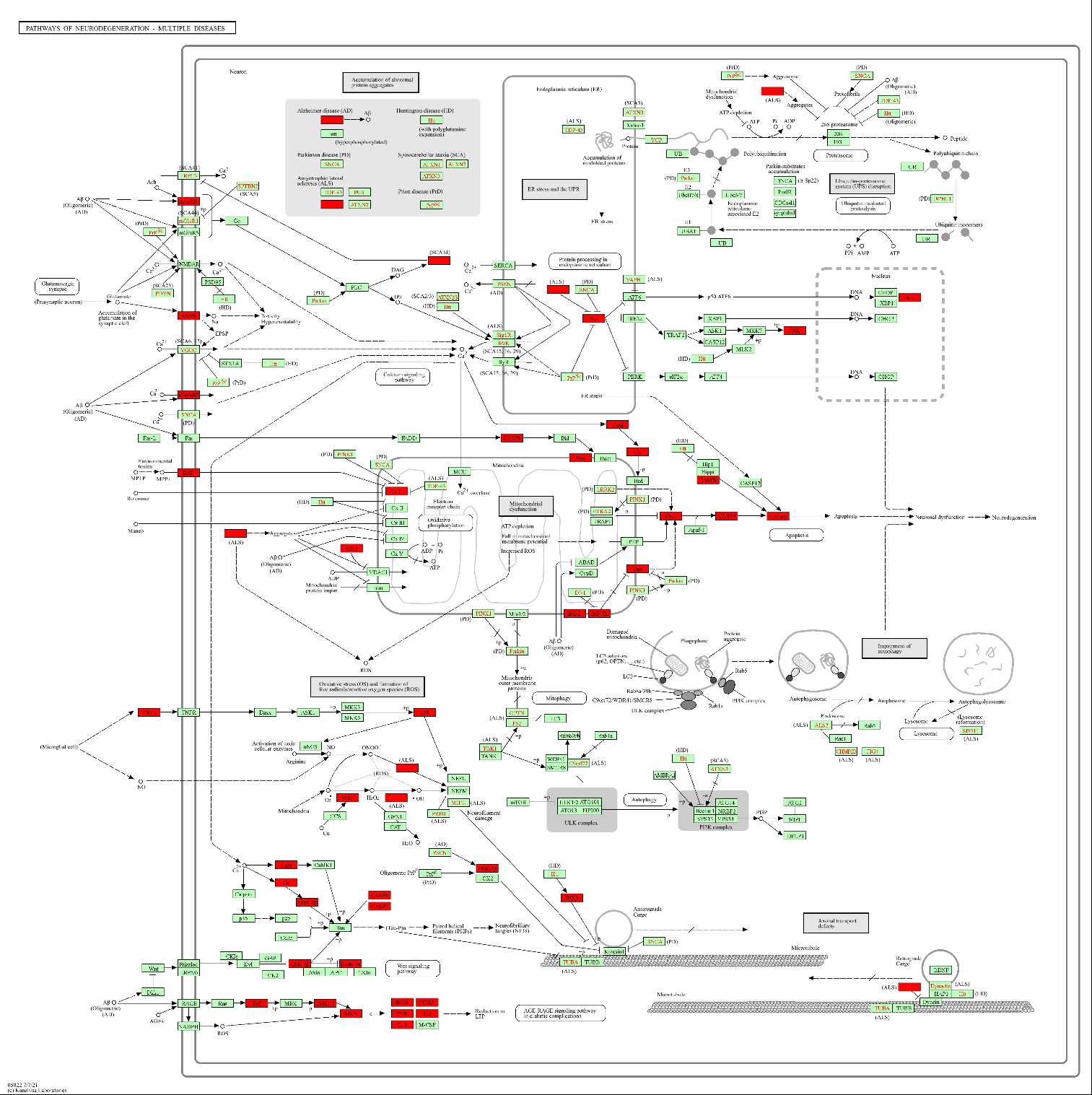 |
| **10. Human T-cell leukemia virus 1 infection** |
| 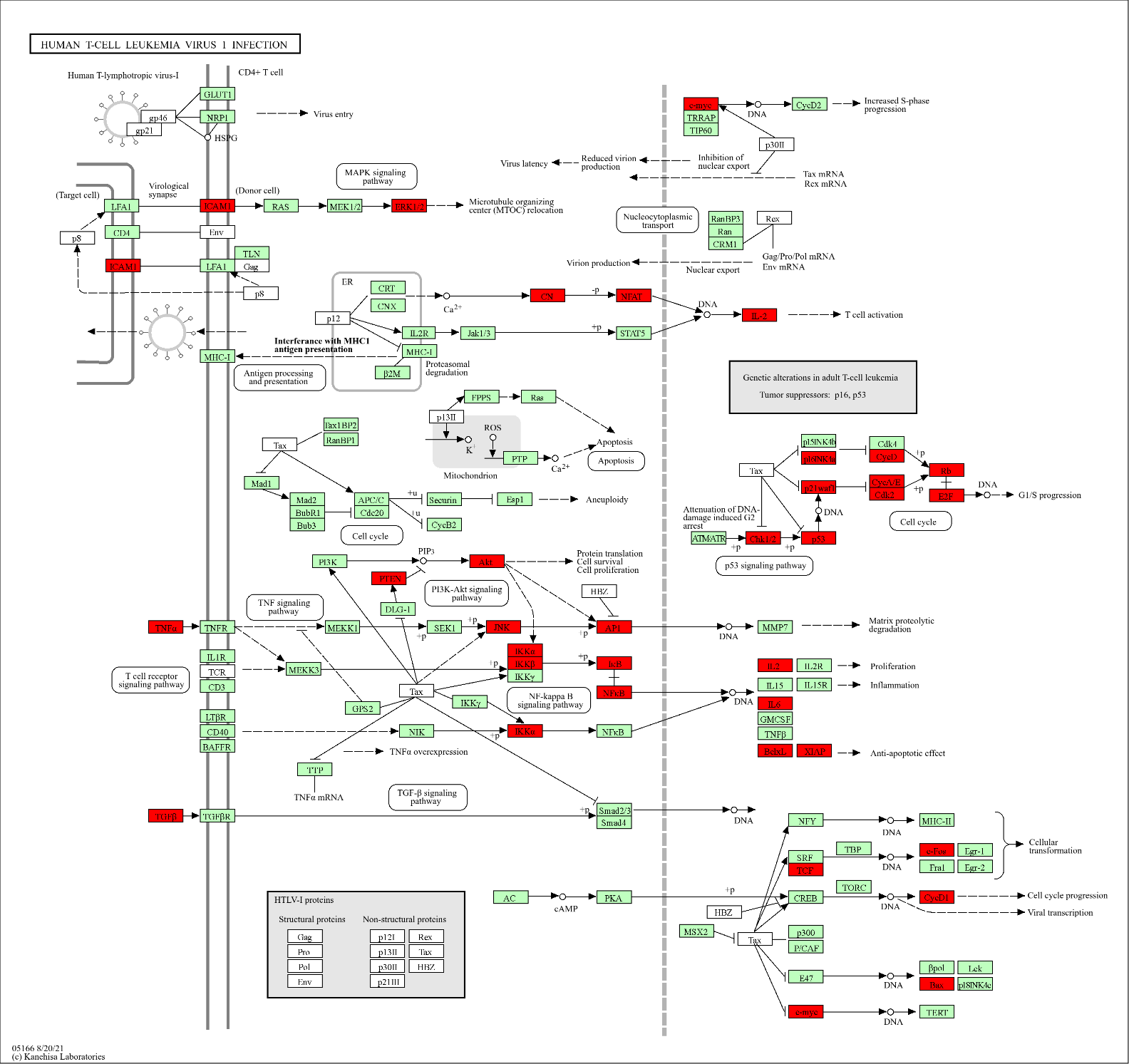 |
