# Supplementary material for: Elucidating the mechanism of Buyang Huanwu Decoction in the treatment of ischemic stroke: A network pharmacology and molecular docking study
Source: Medicine (Baltimore). 2026 Jul 17;105(29):e49736. doi: 10.1097/MD.0000000000049736 (PMC13384647; doi:10.1097/MD.0000000000049736)
Supplement: Supplementary file 12 [file medi-105-e49736-s012.docx]

**S 12.** The identification information for ligand and receptor molecules across each platform and the actual grid box dimensions and center coordinates of receptors.

| **receptor proteins** | | |  |  |
| --- | --- | --- | --- | --- |
| **core targets** | **Gene Ids in Uniport** | **PDB ID** | **center coordinates** | **grid box dimension** |
| TP53 | [P04637](https://www.uniprot.org/uniprotkb/P04637/entry) | [7BWN](https://www.rcsb.org/structure/1C26) | center_x=25.97, center_y=107.885, center_z=-22.605 | size_x=40, size_y=40, size_z=40 |
| JUN | [P05412](https://www.uniprot.org/uniprotkb/P05412/entry) | [1JNM](https://www.rcsb.org/structure/1JNM) | center_x=24.686, center_y=99.145,  center_z=-19.178 | size_x=40, size_y=40, size_z=40 |
| AKT1 | P31749 | [1H10](https://www.rcsb.org/structure/1H10) | center_x=24.562, center_y=95.578, center_z=-17.946 | size_x=40, size_y=40, size_z=40 |
| MAPK1 | [P28482](https://www.uniprot.org/uniprotkb/P28482/entry) | 1TVO | center_x=23.581, center_y=85.347, center_z= -15.004 | size_x=40, size_y=40, size_z=40 |
| MYC | P01106 | 1EE4 | center_x=12.166, center_y=43.209, center_z=74.67 | size_x=40, size_y=40, size_z=40 |
| ESR1 | [P03372](https://www.uniprot.org/uniprotkb/P03372/entry) | 2BJ4 | center_x=21.765, center_y=80.544, center_z=-10.911 | size_x=40, size_y=40, size_z=40 |
| **ligand moleculs** | | |  |  |
| **active ingredients** | **TCMSP IDs** | **Pubchem IDs** |  |  |
| quercetin | MOL000098 | 5280343 |  |  |
| kaempferol | MOL000422 | 5280863 |  |  |
| luteolin | MOL000006 | 5280445 |  |  |
| 7-O-methylisomucronulatol | MOL000378 | 15689652 |  |  |
| isorhamnetin | MOL000354 | 5281654 |  |  |
| formononetin | MOL000392 | 5280378 |  |  |
| beta-sitosterol | MOL000358 | 222284 |  |  |
| baicalein | MOL002714 | 5281605 |  |  |
| Myricanone | MOL002135 | 161748 |  |  |
| Stigmasterol | MOL000449 | 5280794 |  |  |
