# Supplementary material for: Elucidating the mechanism of Buyang Huanwu Decoction in the treatment of ischemic stroke: A network pharmacology and molecular docking study
Source: Medicine (Baltimore). 2026 Jul 17;105(29):e49736. doi: 10.1097/MD.0000000000049736 (PMC13384647; doi:10.1097/MD.0000000000049736)
Supplement: Supplementary file 13 [file medi-105-e49736-s013.docx]

S 13.Results of positive controls in molecular docking experiments.*

| **Hub gene** | **receptor protein** | **positive control** | **PubChem CID** | **binding energy （kcal/mol）** |
| --- | --- | --- | --- | --- |
| TP53 | [7BWN](https://www.rcsb.org/structure/1C26) | APR-246 | 52918385 | -5.2 |
| JUN | [1JNM](https://www.rcsb.org/structure/1JNM) | Leriodenine | 10144 | -8.6 |
| AKT1 | [1H10](https://www.rcsb.org/structure/1H10) | Ipatasertib (GDC-0068) | 24788740 | -8.0 |
| MAPK1 | 1TVO | SCH772984 | 24866313 | -8.8 |
| MYC | 1EE4 | 10058-F4 | 1271002 | -5.2 |
| ESR1 | 2BJ4 | 4-Hydroxytamoxifen | 449459 | -5.8 |

*Binding energy less than -5.0 kcal/mol is used as a screening criterion, indicating a high binding affinity between the ligand and protein.
