# Supplementary material for: Elucidating the mechanism of Buyang Huanwu Decoction in the treatment of ischemic stroke: A network pharmacology and molecular docking study
Source: Medicine (Baltimore). 2026 Jul 17;105(29):e49736. doi: 10.1097/MD.0000000000049736 (PMC13384647; doi:10.1097/MD.0000000000049736)
Supplement: Supplementary file 14 [file medi-105-e49736-s014.docx]

S 14.Hub genes and overlap rates of gene sets were identified and compared by network analysis tools that employ different algorithms.

| **centrality algorithm** | **Number of core targets** | **Target name** |
| --- | --- | --- |
| CytoNCA | 16 | TP53，JUN，AKT1，TNF，IL6，ESR1，RELA，MAPK1，HSP90AA1，BCL2，CASP3，FOS，IL1B，MYC，CTNNB1，BCL2L1， |
| MCODE | 15 | IL1A，RELA，CXCL2，IL6，IL1B，IL4，MAPK1，CXCL10，TNF，IL10，CCL2，CXCL8，IFNG，IL2，HIF1A， |
| CytoHubba | 16 | IL4，IL1B，IL6，CXCL2，IL1A，RELA，IL2，IFNG，CXCL8，CCL2，MYC，TP53，IL10，TNF，JUN，CXCL10， |
| **Overlap rate (CytoNCA vs MCODE) =5/16 *100%=31.25%** | | |
| **Overlap rate (CytoNCA vs CytoHubba) =7/16 *100%=43.75%** | | |
